# Tandem Synthesis of Tetrahydropyrroloquinazolines and Related Polyannular Scaffolds

**DOI:** 10.1021/acs.joc.5c00996

**Published:** 2025-06-18

**Authors:** Haley M. Carlson, R. Adam Mosey

**Affiliations:** 7237 Lake Superior State University, 650 W. Easterday Ave., Sault Sainte Marie, Michigan 49783, United States

## Abstract

A Tf_2_O-mediated tandem synthesis of tetrahydropyrroloquinazolines from amino amides and aldehydes has been developed in which both heterocyclic rings of the scaffold are assembled in a single pot. An alkyl tether linking the amide and amine functionalities acts as a carbon bridge to form saturated rings about the core amidine moiety, thereby facilitating the installation of diverse fused cycloalkyl and bicyclic ring systems. The reaction also extends to the formation of the indoloquinazolinone scaffold.

## Graphical abstract

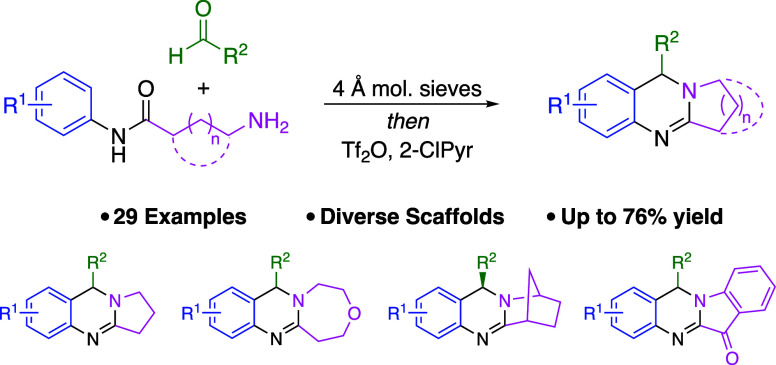

Dihydroquinazolines (DHQs) have garnered significant attention as nitrogen-containing heterocycles with diverse biological activities.^1^ The addition of a pyrrolidine ring about the 2 and 3 carbons of the DHQ scaffold extends the framework into a tetrahydropyrrolo­[2,1-*b*]­quinazoline (THPQ) arrangement (Figure 1). THPQ compounds are found naturally in plants such as (e.g., pegaharmol A and perharmalines F and I), which has been used for its analgesic, anti-inflammatory, antiparasitic, and antitumor properties.^2^ Additionally, the plant, found in many regions throughout Asia, has been used for its bioactive THPQ-type alkaloids including vasicine, vasicinone, vasicol, and vasicinolone.^3^ Vasicine in particular is known for its bronchodilatory properties, and is used in traditional medicine for treating diseases like asthma.^4^

**Figure 1:**
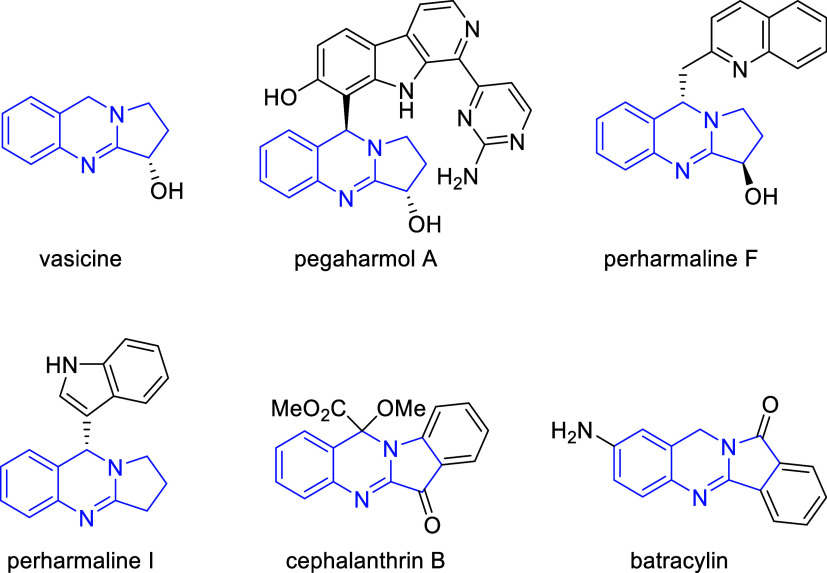
Natural product and synthetic THPQs.

Several methods are known for the synthesis of fused DHQ-pyrrole derivatives about the 2 and 3 positions of the DHQ scaffold.^5^ Most frequently, ring systems of this type have been prepared with the inclusion of a fused pyrrolidinone ring,^6^ like that found in cephalanthrin B and batracylin (Figure 1). Conversely, the synthesis of related molecules bearing fused pyrrolidine rings found in vasicine and pegaharmol A (Figure 1) is less prevalent,^7–9^ with common methods being those which involve carbonyl reduction of fused quinazolinone-pyrrolidine compounds^8^ and oxidation of fused pyrrolidine-tetrahydroquinazoline aminals.^9^ In each of the known syntheses, one or both of the fused heterocyclic rings required preassembly prior to the final synthesis of the scaffold. We previously developed a one-pot method for the construction of DHQs from simple amide, amine, and aldehyde starting materials (Scheme 1).^10^ A similar tandem process, in which the amide and amine functionalities were tethered together through an alkyl linkage, was envisioned to give rise to THPQs and related structural motifs (Scheme 1). Such an approach would permit the assembly of both fused heterocyclic rings of the THPQ scaffold in a single reaction and would allow for the synthesis of diverse compounds bearing the core heterocycle. Herein, we describe the development of a Tf_2_O-mediated one-pot tandem assembly of THPQs and structurally related polyannular compounds from amino amide and aldehyde starting materials.

**Scheme 1:**
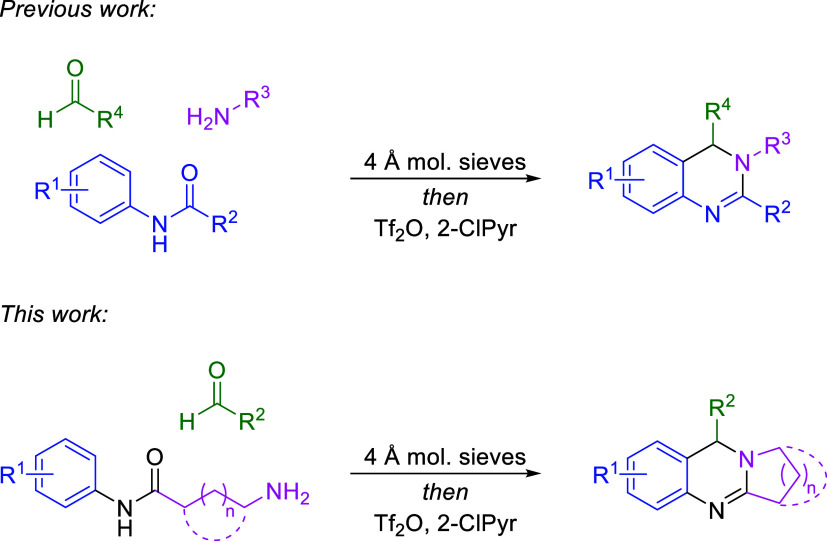
Synthesis of DHQ and THPQ Scaffolds

The proposed synthesis of THPQs through a tandem approach involves initial *in situ* generation of an imine from an amino amide and an aldehyde (Scheme 2). Treatment of imino amide **1a** with Tf_2_O and 2-chloropyridine (2-ClPyr) would give rise to **1b**, which would then undergo intramolecular cyclization to generate iminium intermediate **1c** in which the pyrrolidine ring had been constructed. An ensuing Pictet–Spengler-like cyclization would then generate a second ring to afford the multicyclic THPQ assembly in **2**. To test the reaction, compound **1** was prepared and was stirred with benzaldehyde and molecular sieves for 18 h followed by treatment with Tf_2_O and 2-ClPyr under our previously reported conditions.^10^ To our delight, desired THPQ **2** was generated in 68% yield, as measured by quantitative NMR with 1,3,5-trimethoxybenzene as the internal standard (Table 1, entry 1). Owing to the successive intramolecular cyclizations that occurred during the transformation, the reaction concentration was then varied (entries 2–4). As anticipated, the reaction provided enhanced product yields when performed under more dilute conditions, with the reaction providing optimal results when performed at 0.02 M in DCM (entry 3). Additional solvents were then evaluated in the reaction, but none provided an increase in yield (entries 5–8). Modifications to temperatures or reaction times (entries 9–11) and changes to equivalents of reactants or reagents (entries 12–13) also had no beneficial effect on reaction yield. Lastly, the reaction provided a similar yield of product when 2-fluoropyridine (2-FPyr) replaced 2-ClPyr (entry 14), wherein the reaction likely proceeds through a nitrilium indermediate^11^ rather than **1b** followed by 5-exo-dig cyclization to give the Pictet-Spengler-like annulation precursor **1c**. With the optimized conditions identified (entry 3), the reaction was performed on a 1.0 g scale, resulting in a 69% isolated yield of THPQ **2**.^12^

**Scheme 2:**
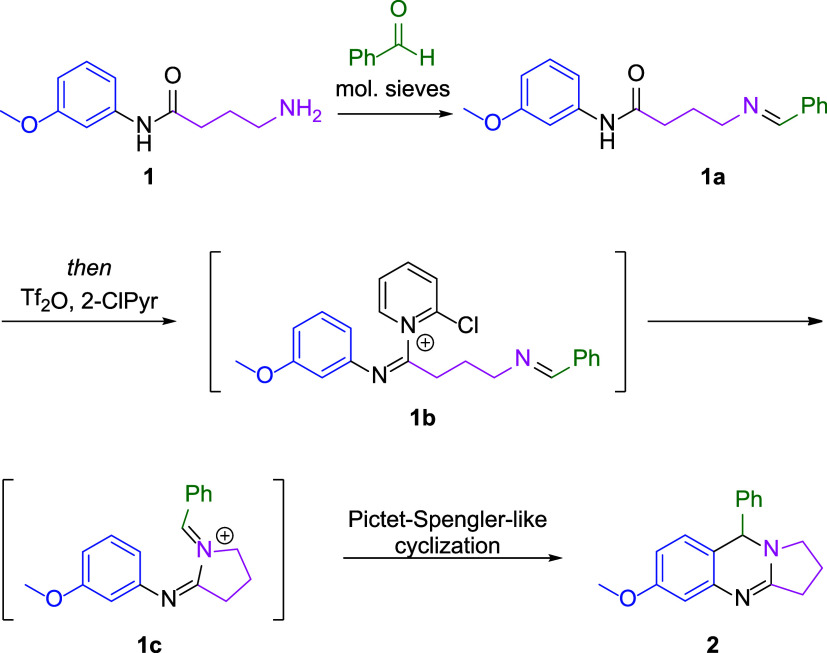
THPQ Tandem Assembly

**Table 1:**
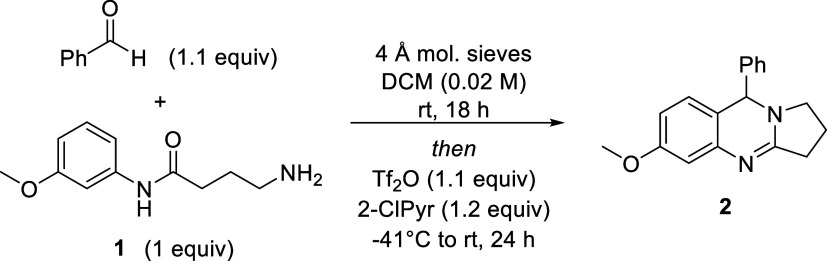
Optimization of Reaction Conditions^a^

entry	variation from the above conditions	yield (%)^b^
1	DCM (0.05 M)	68
2	DCM (0.25 M)	47
**3**	**none**	**74**; **60**^c^; **69**^d^
4	DCM (0.01 M)	74
5	DCE (0.02 M)	42
6	CHCl_3_ (0.02 M)	30
7	PhCl (0.02 M)	50
8	toluene (0.02 M)	24
9	Tf_2_O and 2-ClPyr added at −78 °C	70
10	–41 °C for 1 h before warming to rt	74
11	48 h after addition of Tf_2_O and 2-ClPyr	63
12	1.5 equiv benzaldehyde added	72
13	3.0 equiv 2-ClPyr added	74
14	2-FPyr instead of 2-ClPyr	72

a Conditions: **1** (0.50 mmol), benzaldehyde (0.55 mmol), 4 Å mol. sieves (0.5 g), CH_2_Cl_2_ (25 mL), rt, 18 h; then 2-ClPyr (0.60 mmol), Tf_2_O (0.55 mmol), −41 °C; then rt, 24 h.

b NMR yield with 1,3,5-trimethoxybenzene as internal standard.

c Isolated yield.

d Isolated yield of 1.0 g scale reaction.

Following optimization, the scope of the reaction was explored (Table 2). First, variation of substituents about the anilide portion of the amino amide was investigated (e.g., **4a**–**4g**). The reaction was observed to tolerate electronically and sterically diverse anilides (e.g., **4a**–**4c**), and the use of anilides featuring fused rings resulted in the formation of THPQs with extended ring systems (e.g., **4d**–**4f**). Importantly, the absence of an additional electron-donating character on the starting anilide ring resulted in a very low product yield (e.g., **4g**). Under optimized conditions, THPQ **4g** was observed in only trace amounts. However, the yield was increased to 4% by heating the reaction mixture to 85 °C in DCE following treatment with 2-ClPyr and Tf_2_O. The THPQ scaffold was then decorated with different aromatic and heteroaromatic aldehydes. The reaction showed good functional group tolerance when several substituted benzaldehydes were used (e.g., **4h**–**4p**), wherein lower yields were observed when using ortho-substituted benzaldehydes relative to the para-substituted counterparts (e.g., **4i**–**4j** vs **4o**–**4p**). Additionally, heterocyclic substituents were installed from the use of heteroaromatic aldehydes (e.g., **4q**–**4t**). Notably, the incorporation of 3-indolecarboxaldehyde resulted in the formation of **4t**, the methoxy-substituted derivative of perharmaline I (Figure 1).

**Table 2:**
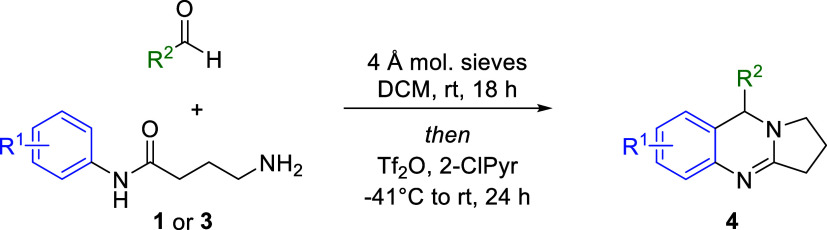

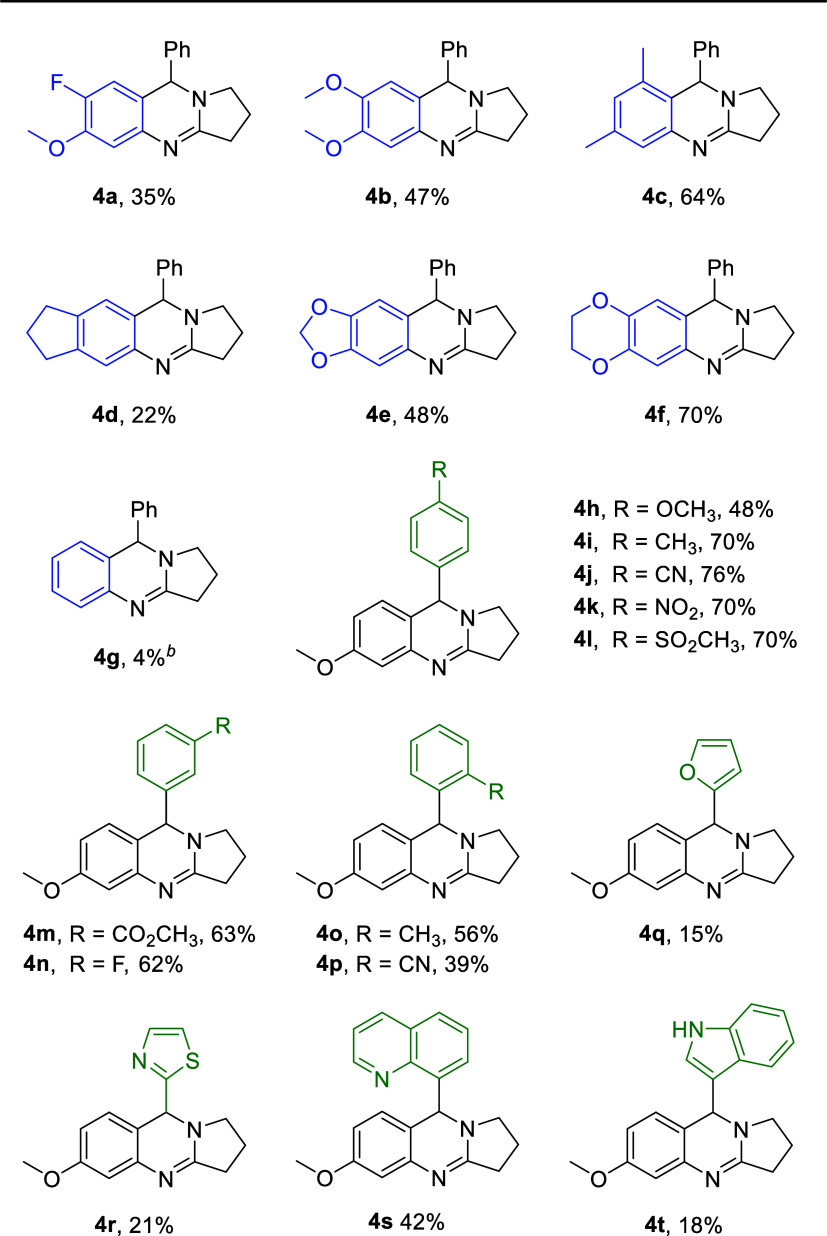
Synthesis of Diverse THPQs^a^

a Conditions: amide **1** or **3** (1.0 mmol), aldehyde (1.1 mmol), 4 Å mol. sieves (1.0 g), CH_2_Cl_2_ (50 mL), rt, 18 h; then 2-ClPyr (1.2 mmol), Tf_2_O (1.1 mmol), −41 °C; then rt, 24 h. Isolated yield.

b DCE used instead of CH_2_Cl_2_; reaction heated to 85 °C after addition of 2-ClPyr and Tf_2_O.

The scope of the reaction was then expanded to include the installation of saturated ring systems of differing sizes and shapes through variation of the alkyl tether bridging the amine and amide functionalities (Table 3). Initially, the alkyl tether was lengthened in an attempt to generate larger 6- and 7-membered saturated rings. Indeed, amino amide **5a**, which features a four-carbon tether between the functional groups, was transformed into piperidine-containing **6a**, the core of which is common to mackinazoline alkaloids.^13^ Similarly, fused azepane analogue **6b** was prepared from **5b**, in which a 5-carbon chain separated the amine and amide. When an ether-containing tether was instead used, oxazepane derivative **6c** was generated. The saturated ring portion of the scaffold was further elaborated to include bicyclic arrangements through the use of cycloalkyl tethers. When subjected to reaction conditions, single enantiomer **5d** was transformed diastereoselectively (d.r. = 5:1) into **6d**, in which the cyclopentyl tether was converted into a [2.2.1] bicyclic arrangement about the core heterocyclic ring. The absolute stereochemistry of the major diastereomer of **6d** was determined by NOESY and single-crystal X-ray analysis.^12^ Likewise, the opposite enantiomer **6e** was generated as the major diastereomer from amino amide **5e** in a 5:1 diastereomeric ratio. To synthesize **6f** with a [3.2.1] bicyclic arrangement, racemic *cis*-1,3-disubstituted cyclohexane **5f** was used. NOESY analysis indicated the major diastereomer to have a relative stereochemistry analogous to **6d**, with the smaller bridge situated on the same face of the molecule as the phenyl substituent.^12^ Lastly, the use of amino amide **5g** containing a *cis*-1,4-disubstituted cyclohexane tether resulted in the formation of the [2.2.2] bicyclic system present in **6g**.

**Table 3:**
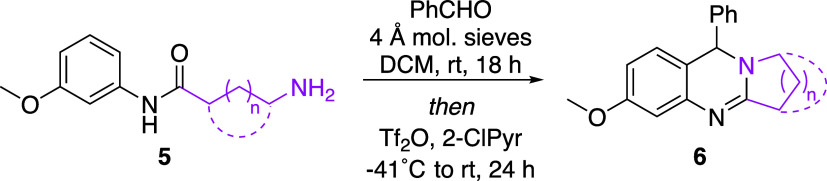

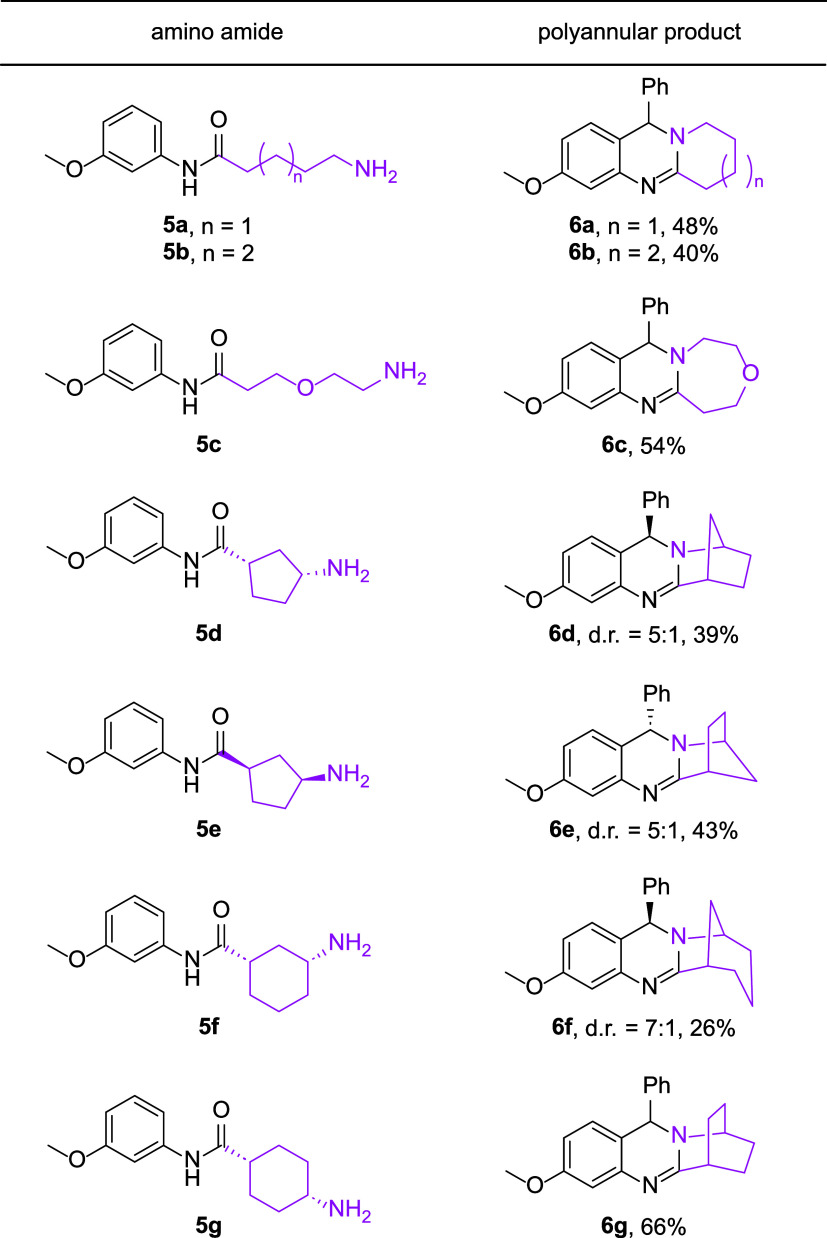
Variation of Saturated Ring Size and Shape

Additional appendage of a fused arene ring to the pyrrolidine ring was then pursued through the use of amino amide **7** (Scheme 3). The benzyl tether separating the functional groups was anticipated to be incorporated into compound **8**, but under optimized reaction conditions, none of the desired product was obtained. Rather, a small amount of oxidized indoloquinazolinone **9**, the polyannular skeleton of which is found in cephalanthrin B (Figure 1), was instead isolated following column chromatography. While compound **9** did not appear to be present in the crude reaction mixture following workup, it was observed to form spontaneously in the presence of air. The formation of **9** was thus promoted by diluting the crude reaction mixture in EtOAc and allowing the reaction to stir open to the air for 24 h.^12^ In this way, indoloquinazolinone **9** was prepared as the major product in 50% yield.

**Scheme 3:**
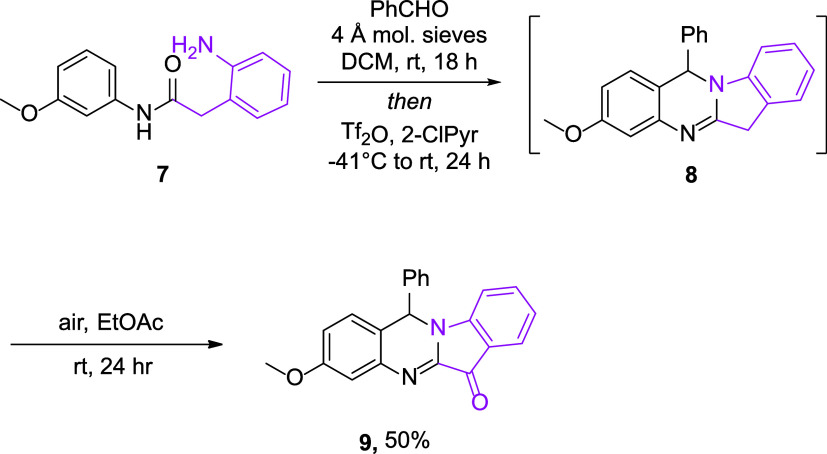
Indoloquinazolinone Synthesis

In conclusion, the syntheses of THPQs and related polyannular analogs are readily afforded from a Tf_2_O-mediated tandem assembly of amino amides and aldehydes. This reaction is unique in that both heteroatom-containing rings of the core multicyclic systems are assembled in a single pot from two simple starting materials. While pyrrolidine rings are installed in the scaffold through the use of a 3-carbon alkyl tether between the amine and amide functional groups, additional alkyl tethers extend the utility of the reaction for the formation of THPQ derivatives bearing fused ring systems of varying size and shape.

## Experimental Section

### General Experimental Information

Reactions were carried out in flame-dried glassware under a nitrogen atmosphere. All reactions were magnetically stirred and monitored by TLC on EMD Millipore silica gel 60F_254_ precoated glass plates using UV light (254 nm) to visualize the compounds. Column chromatography was carried out on a Yamazen AKROS MPLC system using silica gel columns supplied by the Yamazen Corporation (silica gel columns used unless otherwise noted). Proton (^1^H NMR) and carbon (^13^C NMR) nuclear magnetic resonance spectra were recorded on a Bruker Avance III 400 MHz spectrometer. The chemical shifts are given in parts per million (ppm) on the delta (δ) scale. Tetramethylsilane (TMS) or the residual solvent peak was used as a reference value. Infrared spectra were recorded on an Agilent Technologies Cary 630 FT-IR spectrometer. High-resolution mass spectra were recorded on either an Agilent 1290 Ultra-High-Pressure Liquid Chromatograph with a Time of Flight Mass Spectrometer (UHPLC-TOF) at Lake Superior State University or on a Thermo Fisher Scientific Orbitrap Exploris 120 at the Lumigen Instrument Center at Wayne State University. Melting points were obtained using a Mel-Temp capillary melting point apparatus and are uncorrected. Specific rotation data was recorded using a Vernier Chemical polarimeter. HPLC data were recorded on an Agilent 1260 Infinity II system using a Daicel Chiralpak IE-3 chiral column. DCM, DCE, chlorobenzene, toluene, and 2-chloropyridine were dried over 4 Å molecular sieves; all other solvents and chemicals were purchased from commercial vendors and used without additional purification.

### General Procedure for Amino Amide Synthesis (General Procedure A)

To a mixture of a N-Boc-amino acid and 1-(3-(dimethylamino)­propyl)-3-ethylcarbodiimide hydrochloride (EDCI) in DCM was added an amine, and the reaction was stirred for 24 h at room temperature. The reaction mixture was then washed successively with water and saturated aqueous NaHCO_3_ solution before being dried (Na_2_SO_4_) and concentrated. The concentrated residue was subsequently dissolved in DCM and trifluoroacetic acid, and the mixture was stirred for 2 h at room temperature. The reaction mixture was concentrated *in vacuo*, with MeOH additions being used to azeotropically remove trifluoroacetic acid. The crude was then diluted in H_2_O, and the mixture was made basic (pH > 12) with dropwise additions of 8 M NaOH solution. The basic aqueous layer was extracted with DCM (3×), and the organic extracts were dried (Na_2_SO_4_) and concentrated. The crude mixture was then purified via chromatography or crystallization.

### General Procedure for THPQ Synthesis (General Procedure B)

A mixture of amino amide, aldehyde, and 4 Å molecular sieves (∼1 g per mmol of amide) in DCM was prepared and stirred for 18 h at room temperature under a N_2_ atmosphere. The reaction mixture was cooled to −41 °C in an acetonitrile/dry ice bath and was treated with 2-chloropyridine followed by Tf_2_O. The reaction was then allowed to warm to room temperature and was stirred for 24 h. The molecular sieves were filtered from the reaction, and the filtrate was washed with an aqueous NaHCO_3_ solution. The DCM layer was collected and the aqueous layer was extracted with more DCM (×2). The pooled organic extracts were dried (Na_2_SO_4_) and concentrated, and the crude mixture was purified via chromatography.

#### 4-Amino-*N*-(3-methoxyphenyl)­butanamide (**1**)

Prepared according to general procedure A with Boc-y-aminobutyric acid (4.065 g, 20.0 mmol), *m*-anisidine (2.50 mL, 21.2 mmol), EDCI (5.751 g, 30.0 mmol), DCM (40 mL), and for the second step, DCM (24 mL) and trifluoroacetic acid (12 mL). Following workup, the residue was purified by crystallization from EtOAc and hexanes to afford the desired product (2.606 g, 63%) as a white solid (mp = 97–98 °C). ^1^H NMR (400 MHz, CDCl_3_) δ 9.26 (s, 1H), 7.32 (s, 1H), 7.15 (t, *J* = 8.1 Hz, 1H), 7.01 (d, *J* = 7.7 Hz, 1H), 6.61 (dd, *J* = 8.3, 2.5 Hz, 1H), 3.74 (s, 3H), 2.76 (t, *J* = 6.6 Hz, 2H), 2.41 (t, *J* = 7.1 Hz, 2H), 1.81 (p, *J* = 6.9, 6.5 Hz, 2H), 1.66 (s, 2H); ^13^C­{^1^H} NMR (101 MHz, CDCl_3_) δ 171.8, 160.0, 139.7, 129.5, 112.0, 109.5, 105.7, 55.2, 41.4, 35.3, 28.6; IR (neat): 3286, 3198, 3071, 2937, 1664, 1599, 1284, 1156 cm^–1^; HRMS (ESI-TOF) *m*/*z*: [M + H]^+^ Calcd for C_11_H_17_N_2_O_2_ 209.1290; found 209.1292.

#### 6-Methoxy-9-phenyl-1,2,3,9-tetrahydropyrrolo­[2,1-*b*]­quinazoline (**2**)

Prepared according to general procedure B with **1** (0.208 g, 1.0 mmol), benzaldehyde (0.11 mL, 1.1 mmol), 2-chloropyridine (0.11 mL, 1.2 mmol), Tf_2_O (0.19 mL, 1.1 mmol), and DCM (50 mL). Following workup, the residue was purified by MPLC (39–65% MeOH in EtOAc as eluent) to afford the desired product (0.167 g, 60%) as a light yellow solid (mp = 142–144 °C). ^1^H NMR (400 MHz, CDCl_3_) δ 7.37–7.16 (m, 5H), 6.69 (d, *J* = 2.6 Hz, 1H), 6.57 (dd, *J* = 8.4, 0.7 Hz, 1H), 6.45 (dd, *J* = 8.4, 2.6 Hz, 1H), 5.54 (s, 1H), 3.74 (s, 3H), 3.10 (dddd, *J* = 27.3, 9.7, 8.0, 5.9 Hz, 2H), 2.69 (ddd, *J* = 8.5, 7.2, 2.4 Hz, 2H), 2.04–1.83 (m, 2H); ^13^C­{^1^H} NMR (101 MHz, CDCl_3_) δ 161.7, 159.6, 143.6, 142.9, 128.7, 127.9, 127.8, 127.4, 115.6, 110.7, 108.0, 61.3, 55.1, 49.3, 31.8, 18.8; IR (neat): 3061, 2943, 1625, 1595, 1567, 1489, 1284, 1157, 1034 cm^–1^; HRMS (ESI-TOF) *m*/*z*: [M + H]^+^ Calcd for C_18_H_19_N_2_O 279.1497; found 279.1497.

#### Synthesis of Compound **2** on 1.0 g Scale

Prepared according to general procedure B with **1** (1.042 g, 5.00 mmol), benzaldehyde (0.56 mL, 5.5 mmol), 2-chloropyridine (0.56 mL, 6.0 mmol), Tf_2_O (0.93 mL, 5.5 mmol), and DCM (250 mL). Following workup, the residue was purified by MPLC (39–65% MeOH in EtOAc as eluent) to afford the desired product (0.967 g, 69%) as a light yellow solid.

#### 4-Amino-*N*-(4-fluoro-3-methoxyphenyl)­butanamide (**3a**)

Prepared according to general procedure A with Boc-y-aminobutyric acid (1.016 g, 5.0 mmol), 4-fluoro-3-methoxyanaline (0.778 g, 5.50 mmol), EDCI (1.155 g, 6.0 mmol), and DCM (10 mL), and for the second step, DCM (8 mL) and trifluoroacetic acid (4 mL). Following workup, the residue was purified by MPLC (6–35% MeOH in EtOAc as eluent on an amino-functionalized silica gel column) to afford the desired product (1.028 g, 91%) as a light peach solid (mp = 93–96 °C). ^1^H NMR (400 MHz, CDCl_3_) δ 9.75 (s, 1H), 7.47 (dd, *J* = 7.9, 2.3 Hz, 1H), 7.01–6.81 (m, 2H), 3.77 (s, 3H), 2.75 (t, *J* = 6.7 Hz, 2H), 2.42 (t, *J* = 7.2 Hz, 2H), 2.26–1.91 (m, 2H), 1.81 (p, *J* = 7.0 Hz, 2H); ^13^C­{^1^H} NMR (101 MHz, CDCl_3_) δ 171.7, 148.4 (d, ^1^ *J*_*C–F*_ = 242 Hz), 147.0 (d, ^2^ *J*_*C–F*_ = 11 Hz), 134.8 (d, ^3^ *J*_*C–F*_ = 3 Hz), 115.2 (d, ^2^ *J*_*C–F*_ = 19 Hz), 111.7 (d, ^3^ *J*_*C–F*_ = 6 Hz), 106.1, 55.7, 41.1, 34.6, 28.3; IR (neat): 3299, 3164, 3058, 2939, 1664, 1513, 1213, 1116 cm^–1^; HRMS (ESI-TOF) *m*/*z*: [M + H]^+^ Calcd for C_11_H_16_FN_2_O_2_ 227.1196; found 227.1197.

#### 4-Amino-*N*-(3,4-dimethoxyphenyl)­butanamide (**3b**)

Prepared according to general procedure A with Boc-y-aminobutyric acid (1.017 g, 5.0 mmol), 3,4-dimethoxyanaline (0.805 g, 5.26 mmol), EDCI (1.438 g, 7.5 mmol), and DCM (10 mL), and for the second step, DCM (6 mL) and trifluoroacetic acid (3 mL). Following workup, the residue was purified by MPLC (5–35% MeOH in EtOAc as eluent on an amino-functionalized silica gel column) to afford the desired product (0.847 g, 71%) as a purple solid (mp = 98–99 °C). ^1^H NMR (400 MHz, CDCl_3_) δ 9.31 (s, 1H), 7.36 (d, *J* = 2.4 Hz, 1H), 6.93 (dd, *J* = 8.6, 2.4 Hz, 1H), 6.75 (d, *J* = 8.7 Hz, 1H), 3.81 (s, 3H), 3.78 (s, 3H), 2.76 (t, *J* = 6.7 Hz, 2H), 2.40 (t, *J* = 7.2 Hz, 2H), 1.81 (p, *J* = 6.9 Hz, 2H), 1.40 (s, 2H); ^13^C­{^1^H} NMR (101 MHz, CDCl_3_) δ 171.4, 148.6, 145.2, 132.1, 111.7, 111.1, 104.8, 55.8, 55.5, 41.3, 34.7, 28.7; IR (neat): 3351, 3306, 3053, 2937, 1662, 1608, 1513, 1232, 1027 cm^–1^; HRMS (ESI-TOF) *m*/*z*: [M + H]^+^ Calcd for C_12_H_19_N_2_O_3_ 239.1396; found 239.1395.

#### 4-Amino-*N*-(3,5-dimethylphenyl)­butanamide (**3c**)

Prepared according to general procedure A with Boc-y-aminobutyric acid (1.017 g, 5.0 mmol), 3,5-dimethylanaline (0.66 mL, 5.3 mmol), EDCI (1.438 g, 7.5 mmol), and DCM (10 mL), and for the second step, DCM (6 mL) and trifluoroacetic acid (3 mL). Following workup, the residue was purified by MPLC (5–35% MeOH in EtOAc as eluent on an amino-functionalized silica gel column) to afford the desired product (0.668 g, 65%) as a peach solid (mp = 93–94 °C). ^1^H NMR (400 MHz, CDCl_3_) δ 9.29 (s, 1H), 7.16 (s, 2H), 6.68 (s, 1H), 2.72 (t, *J* = 6.7 Hz, 2H), 2.38 (t, *J* = 7.2 Hz, 2H), 2.22 (s, 6H), 1.79 (p, *J* = 7.0 Hz, 2H), 1.41 (s, 2H); ^13^C­{^1^H} NMR (101 MHz, CDCl_3_) δ 171.7, 138.13, 138.11, 125.4, 117.7, 41.3, 34.8, 28.8, 21.1; IR (neat): 3293, 3073, 2920, 1661, 1616, 1560, 1431 cm^–1^; HRMS (ESI-TOF) *m*/*z*: [M + H]^+^ Calcd for C_12_H_19_N_2_O 207.1497; found 207.1494.

#### 4-Amino-*N*-(2,3-dihydro-1*H*-inden-5-yl)­butanamide (**3d**)

Prepared according to general procedure A with Boc-y-aminobutyric acid (1.017 g, 5.0 mmol), 5-indanylamine (0.700 g, 5.25 mmol), EDCI (1.438 g, 7.50 mmol), and DCM (10 mL), and for the second step, DCM (6 mL) and trifluoroacetic acid (3 mL). Following workup, the residue was purified by crystallization from EtOAc and hexanes to afford the desired product (0.631 g, 58%) as a tan solid (mp = 80–83 °C). ^1^H NMR (400 MHz, CDCl_3_) δ 8.58 (s, 1H), 7.46 (s, 1H), 7.18 (dd, *J* = 8.0, 2.0 Hz, 1H), 7.11 (d, *J* = 8.0 Hz, 1H), 2.94–2.74 (m, 6H), 2.43 (t, *J* = 7.1 Hz, 2H), 2.04 (p, *J* = 7.4 Hz, 2H), 1.84 (p, *J* = 6.8 Hz, 2H), 1.52 (s, 2H); ^13^C­{^1^H} NMR (101 MHz, CDCl_3_) δ 171.5, 145.1, 140.0, 136.5, 124.4, 118.1, 116.4, 41.5, 35.4, 33.1, 32.4, 28.8, 25.7; IR (neat): 3288, 3194, 3054, 2939, 1657, 1599, 1543, 1489, 1423 cm^–1^; HRMS (ESI-TOF) *m*/*z*: [M + H]^+^ Calcd for C_13_H_19_N_2_O 219.1497; found 219.1497.

#### 4-Amino-*N*-1,3-benzodioxol-5-ylbutanamide (**3e**)

Prepared according to general procedure A with Boc-y-aminobutyric acid (2.033 g, 10.0 mmol), 3,4-methylenedioxyaniline (1.460 g, 10.6 mmol), EDCI (2.876 g, 15.0 mmol), and DCM (20 mL), and for the second step, DCM (12 mL) and trifluoroacetic acid (6 mL). Following workup, the residue was purified by crystallization from EtOAc and hexanes to afford the desired product (1.168 g, 53%) as a brown solid (mp = 110–112 °C). ^1^H NMR (400 MHz, CDCl_3_) δ 8.73 (s, 1H), 7.24 (s, 1H), 6.81 (d, *J* = 9.2 Hz, 1H), 6.71 (d, *J* = 8.2 Hz, 1H), 5.92 (s, 2H), 2.82 (s, 2H), 2.44 (t, *J* = 6.9 Hz,2), 1.84 (t, *J* = 6.7 Hz, 2H), 1.67 (s, 2H); ^13^C­{^1^H} NMR (101 MHz, CDCl_3_) δ 171.4, 147.8, 144.0, 132.8, 112.9, 108.1, 102.8, 101.3, 41.5, 35.6, 28.5; IR (neat): 3288, 3066, 2937, 1661, 1558, 1489, 1239, 1038 cm^–1^; HRMS (ESI-TOF) *m*/*z*: [M + H]^+^ Calcd for C_11_H_15_N_2_O_3_ 223.1083; found 223.1083.

#### 4-Amino-*N*-(2,3-dihydro-1,4-benzodioxin-6-yl)­butanamide (**3f**)

Prepared according to general procedure A with Boc-y-aminobutyric acid (1.626 g, 8.0 mmol), 3,4-ethylenedioxyaniline (1.270 g, 8.4 mmol), EDCI (2.302 g, 12.0 mmol), and DCM (20 mL), and for the second step, DCM (12 mL) and trifluoroacetic acid (6 mL). Following workup, the residue was purified by MPLC (0–10% MeOH in EtOAc as eluent on an amino-functionalized silica gel column) to afford the desired product (0.941 g, 50%) as a peach solid (mp = 112–113 °C). ^1^H NMR (400 MHz, CDCl_3_) δ 8.82 (s, 1H), 7.15 (d, *J* = 2.4 Hz, 1H), 6.90 (dd, *J* = 8.7, 2.5 Hz, 1H), 6.75 (d, *J* = 8.7 Hz, 1H), 4.20 (s, 4H), 2.78 (t, *J* = 6.6 Hz, 2H), 2.40 (t, *J* = 7.1 Hz, 2H), 2.21 (s, 2H), 1.82 (p, *J* = 6.8 Hz, 2H); ^13^C­{^1^H} NMR (101 MHz, CDCl_3_) δ 171.5, 143.4, 140.2, 132.2, 117.1, 113.6, 109.7, 64.5, 64.3, 41.3, 35.2, 28.6; IR (neat): 3293, 3054, 2937, 1661, 1608, 1506, 1303, 1206, 1068 cm^–1^; HRMS (ESI-TOF) *m*/*z*: [M + H]^+^ Calcd for C_12_H_17_N_2_O_3_ 237.1239; found 237.1239.

#### 4-Amino-*N*-phenylbutanamide (**3g**)

Prepared according to general procedure A with Boc-y-aminobutyric acid (2.033 g, 10.0 mmol), aniline (0.960 mL, 10.5 mmol), EDCI (2.876 g, 15.0 mmol), and DCM (20 mL), and for the second step, DCM (12 mL) and trifluoroacetic acid (6 mL). Following workup, the residue was purified by MPLC (0–20% MeOH in EtOAc as eluent on an amino-functionalized silica gel column) to afford the desired product (0.906 g, 51%) as a beige solid (mp = 136–138 °C). ^1^H NMR (400 MHz, CDCl_3_) δ 9.65 (s, 1H), 7.53 (d, *J* = 7.8 Hz, 2H), 7.23 (t, *J* = 7.9 Hz, 2H), 7.02 (t, *J* = 7.4 Hz, 1H), 2.67 (t, *J* = 6.8 Hz, 2H), 2.37 (t, *J* = 7.3 Hz, 2H), 1.91–1.65 (m, 4H); ^13^C­{^1^H} NMR (101 MHz, CDCl_3_) δ 171.8, 138.3, 128.5, 123.7, 119.9, 41.1, 34.6, 28.6; IR (neat): 3282, 3189, 3055, 2932, 1661, 1597, 1541, 1497, 1441, 1308, 1252 cm^–1^; HRMS (ESI-TOF) *m*/*z*: [M + H]^+^ Calcd for C_10_H_15_N_2_O 179.1184; found 179.1184. The NMR spectral data are consistent with those reported in the literature.^14^

#### 7-Fluoro-6-methoxy-9-phenyl-1,2,3,9-tetrahydropyrrolo­[2,1-*b*]­quinazoline (**4a**)

Prepared according to general procedure B with **3a** (0.227 g, 1.0 mmol), benzaldehyde (0.11 mL, 1.1 mmol), 2-chloropyridine (0.11 mL, 1.2 mmol), Tf_2_O (0.19 mL, 1.1 mmol), and DCM (50 mL). Following workup, the residue was purified by MPLC (80–100% EtOAc in hexanes as an eluent on an amino-functionalized silica gel column) to afford the desired product (0.104 g, 35%) as a tan oil. ^1^H NMR (400 MHz, CDCl_3_) δ 7.39–7.19 (m, 5H), 6.75 (d, *J* = 8.2 Hz, 1H), 6.40 (d, *J* = 11.5 Hz, 1H), 5.55 (s, 1H), 3.85 (s, 3H), 3.17 (ddd, *J* = 9.7, 8.1, 5.5 Hz, 1H), 3.09 (ddd, *J* = 9.7, 7.9, 6.2 Hz, 1H), 2.78–2.61 (m, 2H), 2.09–1.85 (m, 2H); ^13^C­{^1^H} NMR (101 MHz, CDCl_3_) δ 161.4 (d, ^6^ *J*_*C–F*_ = 2 Hz), 149.2 (d, ^1^ *J*_*C–F*_ = 242 Hz), 147.4 (d, ^2^ *J*_*C–F*_ = 12 Hz), 142.4, 139.0 (d, ^4^ *J*_*C–F*_ = 3 Hz), 129.1, 128.4, 127.6, 114.9 (d, ^3^ *J*_*C–F*_ = 6 Hz), 114.1 (d, ^2^ *J*_*C–F*_ = 20 Hz), 108.9 (d, ^4^ *J*_*C–F*_ = 2 Hz), 61.4 (d, ^4^ *J*_*C–F*_ = 2 Hz), 56.1, 49.5, 31.8, 19.0; IR (neat): 3061, 2950, 1608, 1502, 1273, 1161 cm^–1^; HRMS (ESI-TOF) *m*/*z*: [M + H]^+^ Calcd for C_18_H_18_FN_2_O 297.1403; found 297.1401.

#### 6,7-Dimethoxy-9-phenyl-1,2,3,9-tetrahydropyrrolo­[2,1-*b*]­quinazoline (**4b**)

Prepared according to general procedure B with **3b** (0.239 g, 1.0 mmol), benzaldehyde (0.11 mL, 1.1 mmol), 2-chloropyridine (0.11 mL, 1.2 mmol), Tf_2_O (0.19 mL, 1.1 mmol), and DCM (50 mL). Following workup, the residue was purified by MPLC (0–36% MeOH in EtOAc as eluent on an amino-functionalized silica gel column) followed by additional MPLC separation (0–10% MeOH in EtOAc as an eluent on an amino-functionalized silica gel column) to afford the desired product (0.146 g, 47%) as a yellow oil. ^1^H NMR (400 MHz, CDCl_3_) δ 7.39–7.22 (m, 5H), 6.73 (s, 1H), 6.17 (d, *J* = 0.5 Hz, 1H), 5.57 (s, 1H), 3.86 (s, 3H), 3.67 (s, 3H), 3.18 (ddd, *J* = 9.7, 8.1, 5.2 Hz, 1H), 3.13–3.02 (m, 1H), 2.73–2.65 (m, 2H), 2.08–1.84 (m, 2H); ^13^C­{^1^H} NMR (101 MHz, CDCl_3_) δ 160.4, 149.1, 145.9, 142.8, 136.5, 128.9, 128.2, 127.7, 114.4, 110.1, 107.6, 61.8, 56.3, 55.9, 49.4, 31.7, 19.1; IR (neat): 3061, 2935, 1603, 1504, 1261, 1195, 1116 cm^–1^; HRMS (ESI-TOF) *m*/*z*: [M + H]^+^ Calcd for C_19_H_21_N_2_O_2_ 309.1603; found 309.1604.

#### 6,8-Dimethyl-9-phenyl-1,2,3,9-tetrahydropyrrolo­[2,1-*b*]­quinazoline (**4c**)

Prepared according to general procedure B with **3c** (0.206 g, 1.0 mmol), benzaldehyde (0.11 mL, 1.1 mmol), 2-chloropyridine (0.11 mL, 1.2 mmol), Tf_2_O (0.19 mL, 1.1 mmol), and DCM (50 mL). Following workup, the residue was purified by MPLC (0–36% MeOH in EtOAc as eluent on an amino-functionalized silica gel column) to afford the desired product (0.177 g, 64%) as a tan solid (mp = 162–164 °C). ^1^H NMR (400 MHz, CDCl_3_) δ 7.34–7.14 (m, 5H), 6.88 (s, 1H), 6.60 (s, 1H), 5.49 (s, 1H), 3.23 (ddd, *J* = 9.5, 8.3, 3.1 Hz, 1H), 3.09 (q, *J* = 9.0, 8.5 Hz, 1H), 2.67–2.49 (m, 2H), 2.25 (s, 3H), 1.99–1.79 (m, 5H); ^13^C­{^1^H} NMR (101 MHz, CDCl_3_) δ 160.9, 142.9, 141.3, 137.8, 134.3, 128.6, 127.9, 127.8, 127.1, 122.7, 118.9, 59.1, 48.7, 31.7, 21.0, 18.9, 18.6; IR (neat): 3025, 2917, 1603, 1563, 1456, 1267 cm^–1^; HRMS (ESI-TOF) *m*/*z*: [M + H]^+^ Calcd for C_19_H_21_N_2_ 277.1705; found 277.1706.

#### 10-Phenyl-2,3,6,7,8,10-hexahydro-1*H*-cyclopenta­[*g*]­pyrrolo­[2,1-*b*]­quinazoline (**4d**)

Prepared according to general procedure B with **3d** (0.184 g, 0.84 mmol), benzaldehyde (0.090 mL, 0.89 mmol), 2-chloropyridine (0.090 mL, 0.96 mmol), Tf_2_O (0.16 mL, 0.95 mmol), and DCM (50 mL). Following workup, the residue was purified by MPLC (0–10% MeOH in EtOAc as eluent on an amino-functionalized silica gel column) followed by additional MPLC separation (50–80% MeOH in 10% ether/90% DCM as an eluent on an amino-functionalized silica gel column) to afford the desired product (0.053 g, 22%) as a light tan solid (mp = 174–175 °C). ^1^H NMR (400 MHz, CDCl_3_) δ 7.36–7.25 (m, 5H), 6.99 (s, 1H), 6.54 (s, 1H), 5.56 (s, 1H), 3.21–3.01 (m, 2H), 2.82 (t, *J* = 7.4 Hz, 2H), 2.75–2.64 (m, 4H), 2.04–1.89 (m, 4H); ^13^C­{^1^H} NMR (101 MHz, CDCl_3_) δ 160.8, 144.6, 143.3, 140.6, 140.1, 128.9, 128.0, 127.6, 122.7, 121.2, 119.9, 62.1, 49.3, 32.7, 32.4, 31.7, 25.6, 19.2; IR (neat): 3062, 2945, 1599, 1482, 1280 cm^–1^; HRMS (ESI-TOF) *m*/*z*: [M + H]^+^ Calcd for C_20_H_21_N_2_ 289.1705; found 289.1705.

#### 10-Phenyl-6,7,8,10-tetrahydro-[1,3]­dioxolo­[4,5-*g*]­pyrrolo­[2,1-*b*]­quinazoline (**4e**)

Prepared according to general procedure B with **3e** (0.223 g, 1.0 mmol), benzaldehyde (0.11 mL, 1.1 mmol), 2-chloropyridine (0.11 mL, 1.2 mmol), Tf_2_O (0.19 mL, 1.1 mmol), and DCM (50 mL). Following workup, the residue was purified by MPLC (0–20% MeOH in EtOAc as eluent on an amino-functionalized silica gel column) to afford the desired product (0.141 g, 48%) as a tan solid (mp = 170–172 °C). ^1^H NMR (400 MHz, CDCl_3_) δ 7.38–7.19 (m, 5H), 6.65 (s, 1H), 6.14 (s, 1H), 5.82 (d, *J* = 1.4 Hz, 1H), 5.77 (d, *J* = 1.5 Hz, 1H), 5.50 (s, 1H), 3.18–2.99 (m, 2H), 2.70–2.62 (m, 2H), 2.01–1.82 (m, 2H); ^13^C­{^1^H} NMR (101 MHz, CDCl_3_) δ 160.2, 147.3, 144.1, 142.7, 137.3, 128.8, 128.1, 127.4, 115.6, 106.5, 104.8, 100.8, 61.9, 49.2, 31.5, 19.0; IR (neat): 3059, 2974, 1634, 1599, 1478, 1245, 1146, 1036 cm^–1^; HRMS (ESI-TOF) *m*/*z*: [M + H]^+^ Calcd for C_18_H_17_N_2_O_2_ 293.1290; found 293.1290.

#### 11-Phenyl-2,3,7,8,9,11-hexahydro-[1,4]­dioxino­[2,3-*g*]­pyrrolo­[2,1-*b*]­quinazoline (**4f**)

Prepared according to general procedure B with **3f** (0.236 g, 1.0 mmol), benzaldehyde (0.11 mL, 1.1 mmol), 2-chloropyridine (0.11 mL, 1.2 mmol), Tf_2_O (0.19 mL, 1.1 mmol), and DCM (50 mL). Following workup, the residue was purified by MPLC (50–75% EtOAc in 10% ether/90% DCM as eluent on an amino-functionalized silica gel column) to afford the desired product (0.216 g, 70%) as a light yellow solid (mp = 216–218 °C). ^1^H NMR (400 MHz, CDCl_3_) δ 7.35–7.18 (m, 5H), 6.66 (s, 1H), 6.19 (s, 1H), 5.47 (s, 1H), 4.23–3.97 (m, 4H), 3.18–2.97 (m, 2H), 2.74–2.60 (m, 2H), 2.03–1.77 (m, 2H); ^13^C­{^1^H} NMR (101 MHz, CDCl_3_) δ 160.3, 143.1, 142.7, 140.1, 136.5, 128.7, 127.9, 127.3, 116.6, 115.1, 111.9, 64.2, 64.1, 61.2, 49.1, 31.6, 18.9; IR (neat): 3054, 2976, 1608, 1493, 1312, 1159, 1066 cm^–1^; HRMS (ESI-TOF) *m*/*z*: [M + H]^+^ Calcd for C_19_H_19_N_2_O_2_ 307.1447; found 307.1447.

#### 9-Phenyl-1,2,3,9-tetrahydropyrrolo­[2,1-*b*]­quinazoline (**4g**)

Prepared according to general procedure B with **3g** (0.090 g, 0.5 mmol), benzaldehyde (0.06 mL, 0.06 mmol), 2-chloropyridine (0.06 mL, 0.06 mmol), Tf_2_O (0.09 mL, 0.05 mmol), and DCE (25 mL). Prior to treating the reaction mixture with 2-chloropyridine and Tf_2_O, the molecular sieves were filtered, and the filtrate was transferred to a flame-dried pressure vial. The filtered reaction mixture was then treated with 2-chloropyridine and Tf_2_O at −41 °C, and after warming to room temperature, the reaction vial was placed in an aluminum heating block and heated to 85 °C for 24 h prior to workup. Following workup, the residue was purified by MPLC (0–2% MeOH in 10% ether/90% DCM as eluent on an amino-functionalized silica gel column) to afford the desired product (0.005 g, 4%) as a tan solid (mp = 152–155 °C). ^1^H NMR (400 MHz, CDCl_3_) δ 7.37–7.27 (m, 5H), 7.18–7.10 (m, 2H), 6.88 (ddd, *J* = 7.6, 6.5, 2.1 Hz, 1H), 6.70 (d, *J* = 7.6 Hz, 1H), 5.64 (s, 1H), 3.27–3.06 (m, 2H), 2.80–2.66 (m, 2H), 2.07–1.87 (m, 2H); ^13^C­{^1^H} NMR (101 MHz, CDCl_3_) δ 161.6, 142.9, 142.4, 129.0, 128.6, 128.3, 127.7, 127.4, 124.3, 124.2, 123.4, 61.9, 49.5, 32.0, 19.1; IR (neat): 3062, 2924, 1625, 1592, 1569, 1448, 1284 cm^–1^; HRMS (ESI-TOF) *m*/*z*: [M + H]^+^ Calcd for C_17_H_17_N_2_ 249.1392; found 249.1395. The NMR spectral data are consistent with those reported in the literature.^9b^

#### 6-Methoxy-9-(4-methoxyphenyl)-1,2,3,9-tetrahydropyrrolo­[2,1-*b*]­quinazoline (**4h**)

Prepared according to general procedure B with **1** (0.209 g, 1.0 mmol), 4-methoxybenzaldehyde (0.13 mL, 1.1 mmol), 2-chloropyridine (0.11 mL, 1.2 mmol), Tf_2_O (0.19 mL, 1.1 mmol), and DCM (50 mL). Following workup, the residue was purified by MPLC (0–4% MeOH in 10% ether/90% DCM as eluent on an amino-functionalized silica gel column) to afford the desired product (0.148 g, 48%) as a light yellow oil. ^1^H NMR (400 MHz, CDCl_3_) δ 7.17 (d, *J* = 8.7 Hz, 2H), 6.84 (d, *J* = 8.6 Hz, 2H), 6.68 (d, *J* = 2.6 Hz, 1H), 6.57 (dd, *J* = 8.3, 0.7 Hz, 1H), 6.46 (dd, *J* = 8.4, 2.7 Hz, 1H), 5.52 (s, 1H), 3.77 (s, 3H), 3.75 (s, 3H), 3.21–3.04 (m, 2H), 2.74–2.64 (m, 2H), 2.05–1.84 (m, 2H); ^13^C­{^1^H} NMR (101 MHz, CDCl_3_) δ 161.7, 159.7, 159.3, 143.7, 135.4, 128.8, 127.9, 116.0, 114.1, 110.8, 108.0, 60.7, 55.3, 55.2, 49.3, 31.9, 18.9; IR (neat): 3056, 2954, 1595, 1491, 1245, 1157, 1033 cm^–1^; HRMS (ESI-TOF) *m*/*z*: [M + H]^+^ Calcd for C_19_H_21_N_2_O_2_ 309.1603; found 309.1604.

#### 6-Methoxy-9-(4-methylphenyl)-1,2,3,9-tetrahydropyrrolo­[2,1-*b*]­quinazoline (**4i**)

Prepared according to general procedure B with **1** (0.209 g, 1.0 mmol), p-tolualdehyde (0.12 mL, 1.0 mmol), 2-chloropyridine (0.11 mL, 1.2 mmol), Tf_2_O (0.19 mL, 1.1 mmol), and DCM (50 mL). Following workup, the residue was purified by MPLC (85–100% EtOAc in hexanes, followed by 0–10% MeOH in EtOAc as eluent on an amino-functionalized silica gel column) to afford the desired product (0.204 g, 70%) as a yellow oil. ^1^H NMR (400 MHz, CDCl_3_) δ 7.14 (d, *J* = 1.2 Hz, 4H), 6.68 (d, *J* = 2.6 Hz, 1H), 6.58 (dd, *J* = 8.3, 0.7 Hz, 1H), 6.45 (dd, *J* = 8.4, 2.6 Hz, 1H), 5.54 (s, 1H), 3.76 (s, 3H), 3.21–3.05 (m, 2H), 2.74–2.65 (m, 2H), 2.32 (s, 3H), 2.03–1.85 (m, 2H); ^13^C­{^1^H} NMR (101 MHz, CDCl_3_) δ 161.9, 159.7, 143.7, 140.2, 137.8, 129.6, 127.9, 127.6, 116.0, 110.9, 108.0, 61.2, 55.3, 49.4, 32.0, 21.2, 19.0; IR (neat): 3050, 2943, 1595, 1491, 1284, 1157, 1034 cm^–1^; HRMS (ESI-TOF) *m*/*z*: [M + H]^+^ Calcd for C_19_H_21_N_2_O 293.1654; found 293.1638.

#### 4-(6-Methoxy-1,2,3,9-tetrahydropyrrolo­[2,1-*b*]­quinazolin-9-yl)­benzonitrile (**4j**)

Prepared according to general procedure B with **1** (0.209 g, 1.0 mmol), 4-cyanobenzaldehyde (0.144 g, 1.1 mmol), 2-chloropyridine (0.11 mL, 1.2 mmol), Tf_2_O (0.19 mL, 1.1 mmol), and DCM (50 mL). Following workup, the residue was purified by MPLC (0–15% MeOH in EtOAc as eluent on an amino-functionalized silica gel column) to afford the desired product (0.232 g, 76%) as an orange oil. ^1^H NMR (400 MHz, CDCl_3_) δ 7.63 (d, *J* = 8.3 Hz, 2H), 7.39 (d, *J* = 8.3 Hz, 2H), 6.69 (d, *J* = 2.6 Hz, 1H), 6.54 (d, *J* = 8.7 Hz, 1H), 6.47 (dd, *J* = 8.5, 2.6 Hz, 1H), 5.64 (s, 1H), 3.75 (s, 3H), 3.20 (ddd, *J* = 9.7, 8.1, 5.5 Hz, 1H), 3.05 (ddd, *J* = 9.6, 7.9, 6.2 Hz, 1H), 2.70 (ddd, *J* = 8.6, 7.1, 3.5 Hz, 2H), 2.08–1.91 (m, 2H); ^13^C­{^1^H} NMR (101 MHz, CDCl_3_) δ 161.6, 159.9, 147.7, 143.3, 132.7, 128.0, 127.6, 118.3, 114.2, 111.9, 111.0, 108.4, 60.9, 55.1, 49.3, 31.5, 18.8; IR (neat): 3057, 2950, 2227, 1597, 1491, 1286, 1159, 1034 cm^–1^; HRMS (ESI-TOF) *m*/*z*: [M + H]^+^ Calcd for C_19_H_18_N_3_O 304.1450; found 304.1450.

#### 6-Methoxy-9-(4-nitrophenyl)-1,2,3,9-tetrahydropyrrolo­[2,1-*b*]­quinazoline (**4k**)

Prepared according to general procedure B with **1** (0.209 g, 1.0 mmol), 4-nitrobenzaldehyde (0.170 g, 1.1 mmol), 2-chloropyridine (0.11 mL, 1.2 mmol), Tf_2_O (0.19 mL, 1.1 mmol), and DCM (50 mL). Following workup, the residue was purified by MPLC (0–6% MeOH in EtOAc as eluent on an amino-functionalized silica gel column) to afford the desired product (0.226 g, 70%) as an orange solid (mp = 161–162 °C). ^1^H NMR (400 MHz, CDCl_3_) δ 8.20 (d, *J* = 8.7 Hz, 2H), 7.46 (d, *J* = 8.3 Hz, 2H), 6.71 (d, *J* = 2.7 Hz, 1H), 6.56 (d, *J* = 8.5 Hz, 1H), 6.48 (dd, *J* = 8.4, 2.6 Hz, 1H), 5.72 (s, 1H), 3.76 (s, 3H), 3.29–3.19 (m, 1H), 3.14–3.03 (m, 1H), 2.79–2.69 (m, 2H), 2.12–1.91 (m, 2H); ^13^C­{^1^H} NMR (101 MHz, CDCl_3_) δ 161.8, 160.2, 149.7, 147.7, 143.4, 128.3, 127.7, 124.3, 114.2, 111.3, 108.6, 60.9, 55.3, 49.5, 31.6, 19.0; IR (neat): 3075, 2943, 1597, 1521, 1491, 1347, 1288, 1159, 1034 cm^–1^; HRMS (ESI-TOF) *m*/*z*: [M + H]^+^ Calcd for C_18_H_18_N_3_O_3_ 324.1348; found 324.1348.

#### 6-Methoxy-9-(4-methylsulfonyl)-1,2,3,9-tetrahydropyrrolo­[2,1-*b*]­quinazoline (**4l**)

Prepared according to general procedure B with **1** (0.209 g, 1.0 mmol), 4-methylsulfonylbenzaldehyde (0.184 g, 1.1 mmol), 2-chloropyridine (0.11 mL, 1.2 mmol), Tf_2_O (0.19 mL, 1.1 mmol), and DCM (50 mL). Following workup, the residue was purified by MPLC (37–62% MeOH in EtOAc as eluent on a silica gel column) to afford the desired product (0.250 g, 70%) as a light tan solid (mp = 161–164 °C). ^1^H NMR (400 MHz, CDCl_3_) δ 7.91 (d, *J* = 8.4 Hz, 2H), 7.49 (d, *J* = 8.4 Hz, 2H), 6.73 (d, *J* = 2.6 Hz, 1H), 6.58 (d, *J* = 8.5 Hz, 1H), 6.48 (dd, *J* = 8.5, 2.6 Hz, 1H), 5.70 (s, 1H), 3.75 (s, 3H), 3.24 (ddd, *J* = 9.5, 8.1, 5.3 Hz, 1H), 3.12–3.06 (m, 1H), 3.04 (s, 3H), 2.81–2.72 (m, 2H), 2.12–1.89 (m, 2H); ^13^C­{^1^H} NMR (101 MHz, CDCl_3_) δ 161.7, 160.0, 148.6, 143.0, 140.1, 128.2, 128.0, 127.7, 114.2, 111.0, 108.2, 60.7, 55.1, 49.4, 44.2, 31.5, 18.8; IR (neat): 3056, 2928, 1597, 1493, 1306, 1149 cm^–1^; HRMS (ESI-TOF) *m*/*z*: [M + H]^+^ Calcd for C_19_H_21_N_2_O_3_S 357.1273; found 357.1260.

#### Methyl-3-(6-methoxy-1,2,3,9-tetrahydropyrrolo­[2,1-*b*]­quinazolin-9-yl)­benzoate (**4m**)

Prepared according to general procedure B with **1** (0.209 g, 1.0 mmol), methyl-3-formylbenzoate (0.181 g, 1.1 mmol), 2-chloropyridine (0.11 mL, 1.2 mmol), Tf_2_O (0.19 mL, 1.1 mmol), and DCM (50 mL). Following workup, the residue was purified by MPLC (0–2% MeOH in EtOAc as eluent on an amino-functionalized silica gel column) to afford the desired product (0.221 g, 63%) as a light orange oil. ^1^H NMR (400 MHz, CDCl_3_) δ 7.96 (dd, *J* = 7.0, 1.6 Hz, 2H), 7.49–7.38 (m, 2H), 6.70 (d, *J* = 2.6 Hz, 1H), 6.56 (dd, *J* = 8.4, 0.7 Hz, 1H), 6.46 (dd, *J* = 8.4, 2.7 Hz, 1H), 5.65 (s, 1H), 3.90 (s, 3H), 3.75 (s, 3H), 3.17 (ddd, *J* = 9.7, 8.1, 5.6 Hz, 1H), 3.06 (ddd, *J* = 9.7, 7.9, 6.1 Hz, 1H), 2.72 (ddd, *J* = 8.8, 7.1, 5.6 Hz, 2H), 2.05–1.86 (m, 2H); ^13^C­{^1^H} NMR (101 MHz, CDCl_3_) δ 166.6, 161.6, 159.7, 143.5, 143.3, 132.1, 130.5, 129.2, 129.1, 128.2, 127.7, 115.0, 110.8, 108.1, 61.1, 55.1, 52.1, 49.3, 31.7, 18.8; IR (neat): 3057, 2950, 1720, 1595, 1491, 1284, 1159, 1034 cm^–1^; HRMS (ESI-TOF) *m*/*z*: [M + H]^+^ Calcd for C_20_H_21_N_2_O_3_ 337.1552; found 337.1550.

#### 9-(3-Fluorophenyl)-6-methoxy-1,2,3,9-tetrahydropyrrolo­[2,1-*b*]­quinazoline (**4n**)

Prepared according to general procedure B with **1** (0.208 g, 1.0 mmol), 3-fluorobenzaldehyde (0.080 mL, 1.1 mmol), 2-chloropyridine (0.11 mL, 1.2 mmol), Tf_2_O (0.19 mL, 1.1 mmol), and DCM (50 mL). Following workup, the residue was purified by MPLC (0–20% MeOH in EtOAc as eluent on an amino-functionalized silica gel column) to afford the desired product (0.184 g, 62%) as a light yellow oil. ^1^H NMR (400 MHz, CDCl_3_) δ 7.36–7.28 (m, 1H), 7.06 (dt, *J* = 7.7, 1.3 Hz, 1H), 7.02–6.93 (m, 2H), 6.69 (d, *J* = 2.6 Hz, 1H), 6.59 (dd, *J* = 8.4, 0.7 Hz, 1H), 6.48 (dd, *J* = 8.4, 2.7 Hz, 1H), 5.58 (s, 1H), 3.77 (s, 3H), 3.24–3.06 (m, 2H), 2.72 (ddd, *J* = 8.5, 7.2, 3.2 Hz, 2H), 2.08–1.90 (m, 2H); ^13^C­{^1^H} NMR (101 MHz, CDCl_3_) δ 163.4 (d, ^1^ *J*_*C–F*_ = 248 Hz), 161.8, 160.0, 145.7 (d, ^3^ *J*_*C–F*_ = 6 Hz), 143.6, 130.5 (d, ^3^ *J*_*C–F*_ = 8 Hz), 127.9, 123.2 (d, ^4^ *J*_*C–F*_ = 3 Hz), 115.2 (d, ^2^ *J*_*C–F*_ = 22 Hz), 114.5 (d, ^2^ *J*_*C–F*_ = 21 Hz), 111.2, 108.3, 61.2 (d, ^4^ *J*_*C–F*_ = 2 Hz), 55.4, 49.6, 31.9, 19.1; IR (neat): 3057, 2954, 1597, 1491, 1443, 1289, 1161, 1034 cm^–1^; HRMS (ESI-TOF) *m*/*z*: [M + H]^+^ Calcd for C_18_H_18_FN_2_O 297.1403; found 297.1397.

#### 6-Methoxy-9-(2-methylphenyl)-1,2,3,9-tetrahydropyrrolo­[2,1-*b*]­quinazoline (**4o**)

Prepared according to general procedure B with **1** (0.209 g, 1.0 mmol), o-tolualdehyde (0.13 mL, 1.1 mmol), 2-chloropyridine (0.11 mL, 1.2 mmol), Tf_2_O (0.19 mL, 1.1 mmol), and DCM (50 mL). Following workup, the residue was purified by MPLC (0–36% MeOH in EtOAc as eluent on an amino-functionalized silica gel column) followed by additional MPLC separation (0–4% MeOH in 10% ether/DCM as an eluent on an amino-functionalized silica gel column) to afford the desired product (0.166 g, 56%) as an orange oil. ^1^H NMR (400 MHz, CDCl_3_, 330 K) δ 7.25–7.10 (m, 4H), 6.66 (d, *J* = 2.5 Hz, 1H), 6.47 (d, *J* = 8.4 Hz, 1H), 6.42 (dd, *J* = 8.4, 2.5 Hz, 1H), 5.90 (s, 1H), 3.75 (s, 3H), 3.13 (ddd, *J* = 9.7, 8.1, 6.1 Hz, 1H), 3.01 (ddd, *J* = 9.5, 7.9, 5.5 Hz, 1H), 2.75–2.67 (m, 2H), 2.29 (s, 3H), 2.05–1.82 (m, 2H); ^13^C­{^1^H} NMR (101 MHz, CDCl_3_, 330 K) δ 162.0, 160.1, 144.4, 140.5, 136.2, 131.5, 129.7, 128.1, 127.4, 126.7, 115.8, 111.0, 108.5, 59.7, 55.4, 49.6, 31.9, 19.3, 19.1; IR (neat): 3067, 2950, 1597, 1491, 1286, 1157, 1034 cm^–1^; HRMS (ESI-TOF) *m*/*z*: [M + H]^+^ Calcd for C_19_H_21_N_2_O 293.1654; found 293.1648.

#### 2-(6-Methoxy-1,2,3,9-tetrahydropyrrolo­[2,1-*b*]­quinazolin-9-yl)­benzonitrile (**4p**)

Prepared according to general procedure B with **1** (0.209 g, 1.0 mmol), 2-formylbenzonitrile (0.145 g, 1.1 mmol), 2-chloropyridine (0.11 mL, 1.2 mmol), Tf_2_O (0.19 mL, 1.1 mmol), and DCM (10 mL). Following workup, the residue was purified by MPLC (12–38% MeOH in EtOAc as eluent on a silica gel column) to afford the desired product (0.119 g, 39%) as an orange oil. ^1^H NMR (400 MHz, CDCl_3_) δ 7.71–7.63 (m, 1H), 7.57 (ddd, *J* = 8.5, 7.5, 1.4 Hz, 1H), 7.43–7.34 (m, 2H), 6.74 (d, *J* = 2.7 Hz, 1H), 6.66 (dd, *J* = 8.5, 0.7 Hz, 1H), 6.50 (dd, *J* = 8.4, 2.6 Hz, 1H), 6.13 (s, 1H), 3.76 (s, 3H), 3.36 (ddd, *J* = 9.6, 8.2, 5.0 Hz, 1H), 3.13–3.02 (m, 1H), 2.80–2.70 (m, 2H), 2.12–1.92 (m, 2H); ^13^C­{^1^H} NMR (101 MHz, CDCl_3_) δ 161.9, 160.3, 146.5, 142.9, 134.1, 132.8, 129.5, 128.7, 127.7, 117.5, 114.0, 111.7, 110.8, 108.3, 58.8, 55.4, 49.6, 31.4, 19.1; IR (neat): 3056, 2950, 2222, 1593, 1567, 1491, 1286, 1159, 1033 cm^–1^; HRMS (ESI-TOF) *m*/*z*: [M + H]^+^ Calcd for C_19_H_18_N_3_O 304.1450; found 304.1448.

#### 9-(Furan-2-yl)-6-methoxy-1,2,3,9-tetrahydropyrrolo­[2,1-*b*]­quinazoline (**4q**)

Prepared according to general procedure B with **1** (0.209 g, 1.0 mmol), furfural (0.090 mL, 1.1 mmol), 2-chloropyridine (0.11 mL, 1.2 mmol), Tf_2_O (0.19 mL, 1.1 mmol), and DCM (50 mL). Following workup, the residue was purified by MPLC (0–14% MeOH in EtOAc as eluent on an amino-functionalized silica gel column) to afford the desired product (0.041 g, 15%) as a light yellow oil. ^1^H NMR (400 MHz, Acetone-*d*_6_) δ 7.33 (dd, *J* = 1.9, 0.9 Hz, 1H), 6.62 (dd, *J* = 8.4, 0.7 Hz, 1H), 6.42 (d, *J* = 2.6 Hz, 1H), 6.36 (dd, *J* = 8.3, 2.7 Hz, 1H), 6.24 (dd, *J* = 3.2, 1.8 Hz, 1H), 6.18 (dd, *J* = 3.2, 0.8 Hz, 1H), 5.64 (s, 1H), 3.60 (s, 3H), 3.20 (ddd, *J* = 9.5, 7.3, 5.4 Hz, 1H), 3.06 (dtd, *J* = 9.5, 7.4, 0.7 Hz, 1H), 2.50–2.35 (m, 2H), 1.89–1.79 (m, 2H); ^13^C­{^1^H} NMR (101 MHz, Acetone-*d*_6_) δ 161.4, 160.0, 155.0, 144.8, 142.8, 127.3, 113.5, 110.1, 109.6, 108.6, 107.4, 54.5, 53.9, 49.1, 31.1, 18.7; IR (neat): 3072, 2941, 1593, 1567, 1489, 1284, 1157, 1032 cm^–1^; HRMS (ESI-TOF) *m*/*z*: [M + H]^+^ Calcd for C_16_H_17_N_2_O_2_ 269.1290; found 269.1293.

#### 6-Methoxy-9-(1,3-thiazol-2-yl)-1,2,3,9-tetrahydropyrrolo­[2,1-*b*]­quinazoline (**4r**)

Prepared according to general procedure B with **1** (0.209 g, 1.0 mmol), 2-thiazolecarboxaldehyde (0.090 mL, 1.1 mmol), 2-chloropyridine (0.11 mL, 1.2 mmol), Tf_2_O (0.19 mL, 1.1 mmol), and DCM (10 mL). Following workup, the residue was purified by MPLC (80–100% EtOAc in hexane as eluent on an amino-functionalized silica gel column) to afford the desired product (0.060 g, 21%) as a yellow oil. ^1^H NMR (400 MHz, CDCl_3_) δ 7.80 (d, *J* = 3.2 Hz, 1H), 7.41 (dd, *J* = 3.2, 0.8 Hz, 1H), 6.97 (dd, *J* = 8.4, 0.7 Hz, 1H), 6.81 (d, *J* = 2.6 Hz, 1H), 6.65 (dd, *J* = 8.4, 2.7 Hz, 1H), 6.16 (s, 1H), 3.87 (s, 3H), 3.54–3.45 (m, 2H), 2.89–2.72 (m, 2H), 2.21–2.05 (m, 2H); ^13^C­{^1^H} NMR (101 MHz, CDCl_3_) δ 173.1, 161.5, 160.6, 143.4, 142.2, 127.9, 121.1, 113.3, 111.4, 108.6, 58.6, 55.3, 49.8, 31.7, 19.2; IR (neat): 3079, 2939, 1593, 1567, 1491, 1286, 1157, 1033 cm^–1^; HRMS (ESI-TOF) *m*/*z*: [M + H]^+^ Calcd for C_15_H_16_N_3_OS 286.1014; found 286.1021.

#### 6-Methoxy-9-(quinolin-8-yl)-1,2,3,9-tetrahydropyrrolo­[2,1-*b*]­quinazoline (**4s**)

Prepared according to general procedure B with **1** (0.209 g, 1.0 mmol), quinoline-8-carboxaldehyde (0.173 g, 1.1 mmol), 2-chloropyridine (0.11 mL, 1.2 mmol), Tf_2_O (0.19 mL, 1.1 mmol), and DCM (10 mL). Following workup, the residue was purified by MPLC (0–11% MeOH in EtOAc as eluent on an amino-functionalized silica gel column) to afford the desired product (0.140 g, 42%) as a yellow oil. ^1^H NMR (400 MHz, CDCl_3_) δ 8.99 (dd, *J* = 4.2, 1.8 Hz, 1H), 8.15 (dd, *J* = 8.3, 1.8 Hz, 1H), 7.71 (dd, *J* = 8.1, 1.5 Hz, 1H), 7.62 (dd, *J* = 7.3, 1.5 Hz, 1H), 7.48 (dd, *J* = 8.1, 7.2 Hz, 1H), 7.43 (dd, *J* = 8.3, 4.2 Hz, 1H), 7.31 (s, 1H), 6.79 (dd, *J* = 8.4, 0.7 Hz, 1H), 6.72 (d, *J* = 2.6 Hz, 1H), 6.38 (dd, *J* = 8.4, 2.6 Hz, 1H), 3.73 (s, 3H), 3.32 (ddd, *J* = 9.9, 8.3, 4.6 Hz, 1H), 3.08–2.98 (m, 1H), 2.79–2.69 (m, 2H), 2.02–1.82 (m, 2H); ^13^C­{^1^H} NMR (101 MHz, CDCl_3_) δ 162.4, 159.5, 150.1, 145.4, 143.9, 141.5, 136.2, 129.1, 128.0, 127.6, 127.5, 127.0, 121.3, 116.8, 110.6, 108.0, 55.1, 52.9, 49.4, 31.8, 19.1; IR (neat): 3057, 2943, 1593, 1567, 1491, 1288, 1157, 1034 cm^–1^; HRMS (ESI-TOF) *m*/*z*: [M + H]^+^ Calcd for C_21_H_20_N_3_O 330.1606; found 330.1606.

#### 9-(1*H*-Indol-3-yl)-6-methoxy-1,2,3,9-tetrahydropyrrolo­[2,1-*b*]­quinazoline (**4t**)

Prepared according to general procedure B with **1** (0.209 g, 1.0 mmol), indole-3-carboxaldehyde (0.160 g, 1.1 mmol), 2-chloropyridine (0.11 mL, 1.2 mmol), Tf_2_O (0.19 mL, 1.1 mmol), and DCM (50 mL). Following workup, the residue was purified by MPLC (49–100% EtOAc in hexanes as eluent on a silica gel column) to afford the desired product (0.057 g, 18%) as a light yellow solid (mp = 105–108 °C). ^1^H NMR (400 MHz, Acetone-*d*_6_) δ 7.91 (d, *J* = 8.2 Hz, 2H), 7.57–7.46 (m, 2H), 7.35 (ddd, *J* = 8.1, 7.3, 1.0 Hz, 1H), 6.81 (dd, *J* = 8.4, 0.8 Hz, 1H), 6.69 (d, *J* = 2.6 Hz, 1H), 6.48 (dd, *J* = 8.4, 2.6 Hz, 1H), 6.19 (s, 1H), 3.74 (s, 3H), 3.42 (ddd, *J* = 9.7, 8.3, 5.0 Hz, 1H), 3.19–3.09 (m, 1H), 2.74–2.67 (m, 2H), 2.05–2.00 (m, 1H), 1.96–1.86 (m, 1H); ^13^C­{^1^H} (101 MHz, Acetone-*d*_6_) δ 162.0, 160.2, 143.3, 136.4, 128.5, 127.5, 126.6, 126.3, 125.4, 124.3, 121.3, 113.8, 113.1, 110.2, 108.1, 54.5, 53.2, 49.4, 31.1, 18.6; IR (neat): 3062, 2943, 1599, 1493, 1416, 1232, 1202, 1150, 1109, 1034 cm^–1^; HRMS (ESI-TOF) *m*/*z*: [M + H]^+^ Calcd for C_20_H_20_N_3_O 318.1606; found 318.1605.

#### 4-Amino-*N*-(3-methoxyphenyl)­pentanamide (**5a**)

Prepared according to general procedure A with Boc-5-Ava-OH (1.086 g, 5.0 mmol), *m*-anisidine (0.65 mL, 5.5 mmol), EDCI (1.153 g, 6.0 mmol), and DCM (10 mL), and for the second step, DCM (6.0 mL) and trifluoroacetic acid (3.0 mL). Following workup, the residue was purified by MPLC (6–35% MeOH in EtOAc as eluent on an amino-functionalized silica gel column) to afford the desired product (0.810 g, 73%) as a light peach waxy solid (mp = 67–70 °C). ^1^H NMR (400 MHz, CDCl_3_) δ 9.47 (s, 1H), 7.32 (d, *J* = 2.2 Hz, 1H), 7.13 (t, *J* = 8.1 Hz, 1H), 7.05 (d, *J* = 8.0 Hz, 1H), 6.60 (dd, *J* = 8.2, 2.5 Hz, 1H), 3.70 (s, 3H), 2.63 (t, *J* = 7.0 Hz, 2H), 2.32 (t, *J* = 7.5 Hz, 2H), 1.77–1.60 (m, 4H), 1.43 (p, *J* = 7.1 Hz, 2H); ^13^C­{^1^H} NMR (101 MHz, CDCl_3_) δ 171.9, 159.5, 139.4, 129.0, 112.0, 109.0, 105.7, 54.7, 41.3, 36.5, 32.5, 22.5; IR (neat): 3302, 3198, 3062, 2933, 1664, 1597, 1547, 1428, 1284, 1156, 1046 cm^–1^; HRMS (ESI-TOF) *m*/*z*: [M + H]^+^ Calcd for C_12_H_19_N_2_O_2_ 223.1447; found 223.1445.

#### 4-Amino-*N*-(3-methoxyphenyl)­hexanamide (**5b**)

Prepared according to general procedure A with Boc-ε-Acp-OH (1.158 g, 5.0 mmol), *m*-anisidine (0.65 mL, 5.5 mmol), EDCI (1.153 g, 6.0 mmol), and DCM (10 mL), and for the second step, DCM (6.0 mL) and trifluoroacetic acid (3.0 mL). Following workup, the residue was purified by MPLC (11–37% MeOH in EtOAc as eluent on an amino-functionalized silica gel column) to afford the desired product (0.949 g, 80%) as a light peach waxy solid (mp = 49–50 °C). ^1^H NMR (400 MHz, CDCl_3_) δ 9.22 (s, 1H), 7.33 (s, 1H), 7.13 (t, *J* = 8.1 Hz, 1H), 7.06 (d, *J* = 8.0 Hz, 1H), 6.59 (dd, *J* = 8.2, 2.5 Hz, 1H), 3.70 (s, 3H), 2.82 (s, 2H), 2.63 (t, *J* = 7.0 Hz, 2H), 2.31 (t, *J* = 7.5 Hz, 2H), 1.66 (p, *J* = 7.5 Hz, 2H), 1.43 (p, *J* = 7.0 Hz, 2H), 1.32 (td, *J* = 8.5, 4.1 Hz, 2H); ^13^C­{^1^H} NMR (101 MHz, CDCl_3_) δ 172.0, 159.7, 139.6, 129.2, 112.1, 109.3, 105.8, 54.9, 41.3, 36.9, 32.3, 26.1, 25.1; IR (neat): 3288, 3202, 3070, 2930, 1664, 1597, 1545, 1284, 1156, 1047 cm^–1^; HRMS (ESI-TOF) *m*/*z*: [M + H]^+^ Calcd for C_13_H_21_N_2_O_2_ 237.1603; found 237.1610.

#### 3-(2-Aminoethoxy)-*N*-(3-methoxyphenyl)­propanamide (**5c**)

Prepared according to general procedure A with 3-(2-((*tert*-butoxycarbonyl)­amino)­ethoxy)­propanoic acid (1.007 g, 4.3 mmol), *m*-anisidine (0.53 mL, 4.5 mmol), EDCI (1.244 g, 6.5 mmol), and DCM (8 mL), and for the second step, DCM (6.0 mL) and trifluoroacetic acid (3.0 mL). Following workup, the residue was purified by MPLC (8–33% MeOH in EtOAc as eluent on an amino-functionalized silica gel column) to afford the desired product (0.987 g, 96%) as a light peach oil. ^1^H NMR (400 MHz, CDCl_3_) δ 9.15 (s, 1H), 7.32 (t, *J* = 2.2 Hz, 1H), 7.15 (t, *J* = 8.1 Hz, 1H), 7.03 (ddd, *J* = 8.0, 2.0, 1.0 Hz, 1H), 6.61 (ddd, *J* = 8.2, 2.6, 1.0 Hz, 1H), 3.76 (t, *J* = 5.9 Hz, 2H), 3.74 (s, 3H), 3.49 (t, *J* = 5.2 Hz, 2H), 2.86 (t, *J* = 5.2 Hz, 2H), 2.60 (t, *J* = 5.9 Hz, 2H); ^13^C­{^1^H} NMR (101 MHz, CDCl_3_) δ 169.9, 159.8, 139.4, 129.3, 112.0, 109.4, 105.7, 72.8, 66.5, 55.0, 41.4, 37.7; IR (neat): 3297, 3066, 2937, 1666, 1597, 1549, 1491, 1284, 1205, 1113, 1046 cm^–1^; HRMS (ESI-TOF) *m*/*z*: [M + H]^+^ Calcd for C_12_H_19_N_2_O_3_ 239.1396; found 239.1387.

#### (1*S*,3*R*)-3-Amino-*N*-(3-methoxyphenyl)­cyclopentanecarboxamide (**5d**)

Prepared according to general procedure A with (1*S*,3*R*)-3-[(2-methylpropan-2-yl)­oxycarbonylamino]­cyclopentane-1-carboxylic acid (1.074 g, 4.7 mmol), *m*-anisidine (0.58 mL, 4.9 mmol), EDCI (1.353 g, 7.1 mmol), and DCM (10 mL), and for the second step, DCM (8.0 mL) and trifluoroacetic acid (4.0 mL). Following workup, the residue was purified by MPLC (4–36% MeOH in EtOAc as eluent on an amino-functionalized silica gel column) to afford the desired product (0.967 g, 88%) as a light peach waxy solid (mp = 49–50 °C). ^1^H NMR (400 MHz, CDCl_3_) δ 10.71 (s, 1H), 7.36 (d, *J* = 2.2 Hz, 1H), 7.13 (t, *J* = 8.1 Hz, 1H), 7.04 (ddd, *J* = 8.0, 2.0, 1.0 Hz, 1H), 6.57 (ddd, *J* = 8.2, 2.5, 1.0 Hz, 1H), 3.73 (s, 3H), 3.54 (tt, *J* = 5.6, 3.1 Hz, 1H), 2.98–2.80 (m, 1H), 2.12–1.90 (m, 3H), 1.81–1.66 (m, 4H), 1.62–1.53 (m, 1H); ^13^C­{^1^H} NMR (101 MHz, CDCl_3_) δ 176.3, 159.6, 140.3, 129.1, 111.4, 108.6, 104.9, 54.8, 52.8, 46.3, 39.2, 35.4, 29.1; IR (neat): 3354, 3284, 3056, 2939, 1668, 1597, 1552, 1284, 1215, 1046 cm^–1^; HRMS (ESI-TOF) *m*/*z*: [M + H]^+^ Calcd for C_13_H_19_N_2_O_2_ 235.1447; found 235.1448.

#### (1*R*,3*S*)-3-Amino-*N*-(3-methoxyphenyl)­cyclopentanecarboxamide (**5e**)

Prepared according to general procedure A with (1*R*, 3*S*)-3-[(2-methylpropan-2-yl)­oxycarbonylamino]­cyclopentane-1-carboxylic acid (0.918 g, 4.0 mmol), *m*-anisidine (0.49 mL, 4.2 mmol), EDCI (1.151 g, 6.0 mmol), and DCM (10 mL), and for the second step, DCM (7 mL) and trifluoroacetic acid (2.4 mL). Following workup, the residue was purified by MPLC (5–30% MeOH in EtOAc as eluent on an amino-functionalized silica gel column) to afford the desired product (0.706 g, 75%) as a light yellow waxy solid (mp = 50–51 °C). ^1^H NMR (400 MHz, CDCl_3_) δ 10.72 (s, 1H), 7.36 (s, 1H), 7.14 (t, *J* = 8.1 Hz, 1H), 7.03 (ddd, *J* = 8.1, 2.0, 1.0 Hz, 1H), 6.58 (ddd, *J* = 8.2, 2.6, 1.0 Hz, 1H), 3.74 (s, 3H), 3.58 (tt, *J* = 5.5, 2.8 Hz, 1H), 2.88 (dtd, *J* = 9.9, 8.0, 3.8 Hz, 1H), 2.15–1.94 (m, 3H), 1.81–1.69 (m, 2H), 1.66–1.52 (m, 3H); ^13^C­{^1^H} NMR (101 MHz, CDCl_3_) δ 176.4, 159.8, 140.5, 129.2, 111.4, 108.7, 104.9, 55.0, 52.9, 46.6, 39.3, 35.5, 29.3; IR (neat): 3351, 3282, 3060, 2939, 1664, 1597, 1545, 1284, 1213, 1044 cm^–1^; HRMS (ESI-TOF) *m*/*z*: [M + H]^+^ Calcd for C_13_H_19_N_2_O_2_ 235.1447; found 235.1448.

#### *cis*-3-Amino-*N*-(3-methoxyphenyl)­cyclohexanecarboxamide (**5f**)

Prepared according to general procedure A with *cis*-3-((*tert*-butoxycarbonyl)­amino)­cyclohexane carboxylic acid (1.217 g, 5.0 mmol), *m*-anisidine (0.62 mL, 5.3 mmol), EDCI (1.438 g, 7.50 mmol), and DCM (10 mL) for the first step. A white precipitate appeared during the coupling reaction, which was filtered from the reaction mixture instead of an aqueous workup. The solid was diluted in DCM (6 mL) and trifluoroacetic acid (3 mL) for the second step, and the reaction proceeded and was worked up according to the general procedure. The residue was found cleanly to be the desired product (1.196 g, 96%) as a light yellow oil. ^1^H NMR (400 MHz, CDCl_3_) δ 9.04 (s, 1H), 7.35 (s, 1H), 7.14 (t, *J* = 8.1 Hz, 1H), 7.06 (d, *J* = 8.6 Hz, 1H), 6.61 (dd, *J* = 8.2, 2.5 Hz, 1H), 3.70 (s, 3H), 2.57 (ddt, *J* = 11.4, 7.9, 3.7 Hz, 1H), 2.32 (td, *J* = 10.2, 8.5, 6.0 Hz, 1H), 1.99 (d, *J* = 12.6 Hz, 1H), 1.90–1.74 (m, 3H), 1.51–1.16 (m, 5H), 1.08–0.92 (m, 1H); ^13^C­{^1^H} NMR (101 MHz, CDCl_3_) δ 174.2, 159.6, 139.5, 129.1, 112.2, 109.4, 105.7, 54.8, 49.7, 44.8, 39.2, 35.6, 28.4, 23.9; IR (neat): 3306, 3282, 3065, 2932, 1661, 1601, 1545, 1284, 1211, 1046 cm^–1^; HRMS (ESI-TOF) *m*/*z*: [M + H]^+^ Calcd for C_14_H_21_N_2_O_2_ 249.1603; found 249.1603.

#### *cis*-4-Amino-*N*-(3-methoxyphenyl)­cyclohexanecarboxamide (**5g**)

Prepared according to general procedure A with *cis*-4-(*tert*-butoxycarbonylamino)­cyclohexane carboxylic acid (1.217 g, 5.0 mmol), *m*-anisidine (0.62 mL, 5.3 mmol), EDCI (1.438 g, 7.5 mmol), and DCM (10 mL), and for the second step, DCM (6 mL) and trifluoroacetic acid (3 mL). Following workup, the residue was purified by MPLC (2–10% MeOH in EtOAc as eluent on an amino-functionalized silica gel column) followed by crystallization from EtOAc and hexanes to afford the desired product (0.563 g, 45%) as a white solid (mp = 138–139 °C). ^1^H NMR (400 MHz, CDCl_3_) δ 7.72 (s, 1H), 7.35 (t, *J* = 2.3 Hz, 1H), 7.18 (t, *J* = 8.1 Hz, 1H), 6.98 (dd, *J* = 8.0, 0.8 Hz, 1H), 6.64 (ddd, *J* = 8.3, 2.5, 0.9 Hz, 1H), 3.78 (s, 3H), 3.03 (p, *J* = 4.6 Hz, 1H), 2.35 (ddt, *J* = 13.0, 8.7, 3.9 Hz, 1H), 2.04–1.91 (m, 2H), 1.77–1.58 (m, 6H), 1.42 (s, 2H); ^13^C­{^1^H} NMR (101 MHz, CDCl_3_) δ 174.1, 160.2, 139.5, 129.6, 112.0, 110.1, 105.6, 55.3, 46.6, 44.3, 32.6, 24.5; IR (neat): 3293, 3058, 2924, 1662, 1599, 1541, 1211, 1054 cm^–1^; HRMS (ESI-TOF) *m*/*z*: [M + H]^+^ Calcd for C_14_H_21_N_2_O_2_ 249.1603; found 249.1601.

#### 3-Methoxy-11-phenyl-6,8,9,11-tetrahydro-7*H*-pyrido­[2,1-*b*]­quinazoline (**6a**)

Prepared according to general procedure B with **5a** (0.222 g, 1.0 mmol), benzaldehyde (0.11 mL, 1.1 mmol), 2-chloropyridine (0.11 mL, 1.2 mmol), Tf_2_O (0.19 mL, 1.1 mmol), and DCM (10 mL). Following workup, the residue was purified by MPLC (65–85% EtOAc in hexane as eluent on an amino-functionalized silica gel column) to afford the desired product (0.140 g, 48%) as a light yellow solid (mp = 162–165 °C). ^1^H NMR (400 MHz, CDCl_3_) δ 7.34–7.16 (m, 5H), 6.66 (d, *J* = 2.6 Hz, 1H), 6.62 (dd, *J* = 8.3, 0.7 Hz, 1H), 6.45 (dd, *J* = 8.4, 2.6 Hz, 1H), 5.28 (s, 1H), 3.73 (s, 3H), 3.02 (t, *J* = 6.2 Hz, 2H), 2.75–2.64 (m, 1H), 2.63–2.51 (m, 1H), 1.84–1.69 (m, 3H), 1.69–1.55 (m, 1H); ^13^C­{^1^H} NMR (101 MHz, CDCl_3_) δ 159.6, 156.2, 144.1, 142.3, 128.8, 127.8, 127.0, 126.7, 116.7, 110.9, 107.2, 64.9, 55.1, 48.2, 32.1, 23.3, 20.3; IR (neat): 3060, 2945, 1590, 1552, 1493, 1150, 1034 cm^–1^; HRMS (ESI-TOF) *m*/*z*: [M + H]^+^ Calcd for C_19_H_21_N_2_O 293.1654; found 293.1655.

#### 3-Methoxy-12-phenyl-6,7,8,9,10,12-hexahydroazepino­[2,1-*b*]­quinazoline (**6b**)

Prepared according to general procedure B with **5b** (0.237 g, 1.0 mmol), benzaldehyde (0.11 mL, 1.1 mmol), 2-chloropyridine (0.11 mL, 1.2 mmol), Tf_2_O (0.19 mL, 1.1 mmol), and DCM (50 mL). Following workup, the residue was purified by MPLC (0–20% MeOH in EtOAc as eluent on an amino-functionalized silica gel column) to afford the desired product (0.124 g, 40%) as a white solid (mp = 164–166 °C). ^1^H NMR (400 MHz, CDCl_3_) δ 7.33–7.16 (m, 5H), 6.70 (d, *J* = 2.6 Hz, 1H), 6.61 (dd, *J* = 8.3, 0.6 Hz, 1H), 6.46 (dd, *J* = 8.4, 2.6 Hz, 1H), 5.42 (s, 1H), 3.75 (s, 3H), 3.39 (ddd, *J* = 15.1, 8.9, 1.3 Hz, 1H), 3.19 (ddd, *J* = 15.3, 8.5, 1.5 Hz, 1H), 2.73–2.56 (m, 2H), 1.81–1.68 (m, 2H), 1.68–1.42 (m, 3H), 1.30–1.16 (m, 1H); ^13^C­{^1^H} NMR (101 MHz, CDCl_3_) δ 161.9, 159.6, 145.1, 142.6, 128.9, 127.9, 127.1, 126.9, 117.5, 111.4, 107.7, 66.2, 55.2, 52.2, 37.4, 29.8, 28.3, 25.1; IR (neat): 3060, 2928, 1586, 1552, 1497, 1142, 1044 cm^–1^; HRMS (ESI-TOF) *m*/*z*: [M + H]^+^ Calcd for C_20_H_23_N_2_O 307.1810; found 307.1811.

#### 8-Methoxy-11-phenyl-1,2,5,11-tetrahydro-4*H*-[1,4]­oxazepino­[5,4-*b*]­quinazoline (**6c**)

Prepared according to general procedure B with **5c** (0.239 g, 1.0 mmol), benzaldehyde (0.11 mL, 1.1 mmol), 2-chloropyridine (0.11 mL, 1.2 mmol), Tf_2_O (0.19 mL, 1.1 mmol), and DCM (50 mL). Following workup, the residue was purified by MPLC (75–100% EtOAc in DCM as eluent on an amino-functionalized silica gel column) to afford the desired product (0.166 g, 54%) as a light yellow solid (mp = 140–143 °C). ^1^H NMR (400 MHz, CDCl_3_) δ 7.37–7.20 (m, 5H), 6.71 (d, *J* = 2.6 Hz, 1H), 6.62 (dd, *J* = 8.3, 0.6 Hz, 1H), 6.51 (dd, *J* = 8.4, 2.6 Hz, 1H), 5.41 (s, 1H), 3.86–3.79 (m, 2H), 3.76 (s, 3H), 3.63–3.46 (m, 2H), 3.37–3.25 (m, 2H), 2.96–2.79 (m, 2H); ^13^C­{^1^H} NMR (101 MHz, CDCl_3_) δ 160.0, 159.8, 144.3, 142.0, 129.1, 128.3, 127.09, 127.06, 117.1, 112.0, 108.0, 70.1, 67.2, 66.3, 55.3, 54.4, 41.2; IR (neat): 3060, 2956, 1588, 1558, 1497, 1156, 1137, 1034 cm^–1^; HRMS (ESI-TOF) *m*/*z*: [M + H]^+^ Calcd for C_19_H_21_N_2_O_2_ 309.1603; found 309.1592.

#### (6*S*,9*R*,11*R*)-3-Methoxy-11-phenyl-6,8,9,11-tetrahydro-7*H*-6,9-methanopyrido­[2,1-*b*]­quinazoline (**6d**)

Prepared according to general procedure B with **5d** (0.234 g, 1.0 mmol), benzaldehyde (0.11 mL, 1.1 mmol), 2-chloropyridine (0.11 mL, 1.2 mmol), Tf_2_O (0.19 mL, 1.1 mmol), and DCM (50 mL). Following workup, the residue was purified by MPLC (50–100% EtOAc in hexane as eluent on an amino-functionalized silica gel column) to afford a 5:1 mixture of diastereomers. Additional MPLC purification (30–0% hexanes in 10% ether/DCM as eluent on an amino-functionalized silica gel column) afforded the major diastereomer product (0.118 g, 39%) as a white solid (mp = 165–167 °C). [α]_D_^20^ = −58.5 (*c* = 0.01, CH_2_Cl_2_); ^1^H NMR (400 MHz, CDCl_3_) δ 7.44–7.30 (m, 5H), 6.70 (d, *J* = 2.6 Hz, 1H), 6.42 (dd, *J* = 8.5, 2.6 Hz, 1H), 6.38 (d, *J* = 8.5 Hz, 1H), 5.47 (s, 1H), 3.75 (s, 3H), 3.45 (s, 1H), 3.13 (d, *J* = 1.9 Hz, 1H), 1.99–1.82 (m, 2H), 1.79–1.60 (m, 3H), 1.32 (d, *J* = 9.3 Hz, 1H); ^13^C­{^1^H} NMR (101 MHz, CDCl_3_) δ 166.3, 159.8, 143.9, 142.1, 128.8, 128.8, 128.2, 128.1, 117.4, 110.7, 108.3, 59.1, 58.1, 55.3, 45.1, 38.9, 26.1, 24.7; IR (neat): 3063, 2954, 1636, 1601, 1491, 1154, 1032 cm^–1^; HRMS (ESI-TOF) *m*/*z*: [M + H]^+^ Calcd for C_20_H_21_N_2_O 305.1654; found 305.1652.

#### (6*R*,9*S*,11*S)*-3-Methoxy-11-phenyl-6,8,9,11-tetrahydro-7*H*-6,9-methanopyrido­[2,1-*b*]­quinazoline (**6e**)

Prepared according to general procedure B with **5e** (0.235 g, 1.0 mmol), benzaldehyde (0.11 mL, 1.1 mmol), 2-chloropyridine (0.11 mL, 1.2 mmol), Tf_2_O (0.19 mL, 1.1 mmol), and DCM (50 mL). Following workup, the residue was purified by MPLC (50–100% EtOAc in hexanes as eluent on an amino-functionalized silica gel column) to afford a 5:1 mixture of diastereomers. Additional MPLC purification (30–0% hexanes in 10% ether/DCM as eluent on an amino-functionalized silica gel column) afforded the major diastereomer product (0.132 g, 43%) as a white solid (mp = 165–168 °C). [α]_D_^20^ = +52.8 (*c* = 0.01, CH_2_Cl_2_); ^1^H NMR (400 MHz, CDCl_3_) δ 7.43–7.31 (m, 5H), 6.69 (d, *J* = 2.5 Hz, 1H), 6.42 (dd, *J* = 8.5, 2.5 Hz, 1H), 6.38 (dd, *J* = 8.4, 0.8 Hz, 1H), 5.47 (s, 1H), 3.75 (s, 3H), 3.44 (s, 1H), 3.12 (dd, *J* = 3.2, 1.5 Hz, 1H), 1.99–1.83 (m, 2H), 1.77–1.62 (m, 3H), 1.32 (d, *J* = 9.3 Hz, 1H); ^13^C­{^1^H} NMR (101 MHz, CDCl_3_) δ 166.4, 159.8, 144.1, 142.2, 128.9, 128.8, 128.2, 128.1, 117.5, 110.8, 108.4, 59.1, 58.1, 55.3, 45.1, 39.0, 26.1, 24.7; IR (neat): 3061, 2950, 1636, 1597, 1489, 1154, 1034 cm^–1^; HRMS (ESI-TOF) *m*/*z*: [M + H]^+^ Calcd for C_20_H_21_N_2_O 305.1654; found 305.1657.

#### (6R*,10S*,12S*)-3-Methoxy-12-phenyl-6,7,8,9,10,12-hexahydro-6,10-methanoazepino­[2,1-*b*]­quinazoline (**6f**)

Prepared according to general procedure B with **5f** (0.125 g, 0.50 mmol), benzaldehyde (0.06 mL, 0.06 mmol), 2-chloropyridine (0.060 mL, 0.64 mmol), Tf_2_O (0.090 mL, 0.53 mmol), and DCM (25 mL). Following workup, the residue was purified by MPLC (26–80% MeOH in EtOAc as eluent on a silica gel column) to afford the major diastereomer product (0.041 g, 26%) as a light orange solid (mp = 102–104 °C). ^1^H NMR (400 MHz, CDCl_3_) δ 7.41–7.30 (m, 5H), 6.74 (dd, *J* = 2.3, 0.7 Hz, 1H), 6.47–6.39 (m, 2H), 5.66 (s, 1H), 3.77 (s, 3H), 3.29 (t, *J* = 4.8 Hz, 1H), 2.88–2.83 (m, 1H), 2.08–1.96 (m, 2H), 1.92 (d, *J* = 16.9 Hz, 1H), 1.79–1.61 (m, 3H), 1.51 (d, *J* = 10.8 Hz, 1H), 1.45–1.35 (m, 1H); ^13^C­{^1^H} NMR (101 MHz, CDCl_3_) δ 164.8, 159.9, 144.0, 142.5, 128.9, 128.6, 128.24, 128.23, 116.9, 111.1, 108.3, 58.4, 56.0, 55.4, 41.2, 37.0, 28.2, 23.5, 18.6; IR (neat): 3063, 2939, 1653, 1605, 1485, 1286, 1154, 1027 cm^–1^; HRMS (ESI-TOF) *m*/*z*: [M + H]^+^ Calcd for C_21_H_23_N_2_O 319.1810; found 319.1810.

#### 3-Methoxy-11-phenyl-6,8,9,11-tetrahydro-7*H*-6,9-ethanopyrido­[2,1-*b*]­quinazoline (**6g**)

Prepared according to general procedure B with **5g** (0.249 g, 1.0 mmol), benzaldehyde (0.11 mL, 1.1 mmol), 2-chloropyridine (0.11 mL, 1.2 mmol), Tf_2_O (0.19 mL, 1.1 mmol), and DCM (50 mL). Following workup, the residue was purified by MPLC (0–10% MeOH in EtOAc as eluent on an amino-functionalized silica gel column) to afford the desired product (0.210 g, 66%) as a white solid (mp = 140–142 °C). ^1^H NMR (400 MHz, CDCl_3_) δ 7.34–7.21 (m, 5H), 6.71 (d, *J* = 2.6 Hz, 1H), 6.52 (dd, *J* = 8.3, 0.7 Hz, 1H), 6.43 (dd, *J* = 8.4, 2.6 Hz, 1H), 5.55 (s, 1H), 3.75 (s, 3H), 3.27 (s, 1H), 2.72 (s, 1H), 1.92–1.81 (m, 2H), 1.80–1.56 (m, 4H), 1.46–1.34 (m, 1H), 1.17–1.05 (m, 1H); ^13^C­{^1^H} NMR (101 MHz, CDCl_3_) δ 162.6, 159.5, 144.5, 143.4, 128.7, 127.9, 127.8, 127.5, 117.5, 110.7, 107.7, 61.9, 55.1, 51.7, 36.6, 27.2, 26.6, 24.5, 24.4; IR (neat): 3060, 2954, 1592, 1562, 1489, 1448, 1126, 1033 cm^–1^; HRMS (ESI-TOF) *m*/*z*: [M + H]^+^ Calcd for C_21_H_23_N_2_O 319.1810; found 319.1810.

#### *N*-(2′-Aminophenylacetyl)-3-methoxyaniline (**7**)

Prepared according to general procedure A with 2-(2-((*tert*-butoxycarbonyl)­amino)­phenyl)­acetic acid (1.040 g, 4.1 mmol), *m*-anisidine (0.51 mL, 4.3 mmol), EDCI (1.193 g, 6.2 mmol), and DCM (8 mL), and for the second step, DCM (6 mL) and trifluoroacetic acid (3 mL). Following workup, the residue was purified by MPLC (DCM as eluent on an amino-functionalized silica gel column) to afford the desired product (0.614 g, 58%) as a tan solid (mp = 104–106 °C). ^1^H NMR (400 MHz, Acetone-*d*_6_) δ 9.45 (s, 1H), 7.45 (d, *J* = 2.1 Hz, 1H), 7.23–7.13 (m, 3H), 7.05 (td, *J* = 7.7, 1.6 Hz, 1H), 6.80 (dd, *J* = 7.9, 1.2 Hz, 1H), 6.72–6.60 (m, 2H), 4.90 (s, 2H), 3.72 (s, 3H), 3.67 (s, 2H); ^13^C­{^1^H} NMR (101 MHz, Acetone-*d*_6_) δ 170.8, 160.7, 147.5, 140.9, 131.3, 130.1, 128.6, 121.3, 118.4, 116.7, 112.5, 109.9, 106.1, 55.3, 41.8; IR (neat): 3327, 3064, 2958, 1661, 1597, 1543, 1493, 1286, 1157, 1042 cm^–1^; HRMS (ESI-TOF) *m*/*z*: [M + H]^+^ Calcd for C_15_H_17_N_2_O_2_ 257.1290; found 257.1282.

#### 3-Methoxy-12-phenylindolo­[2,1-*b*]­quinazolin-6­(12*H*)-one (**9**)

Prepared according to general procedure B with **7** (0.257 g, 1.0 mmol), benzaldehyde (0.11 mL, 1.1 mmol), 2-chloropyridine (0.11 mL, 1.2 mmol), Tf_2_O (0.19 mL, 1.1 mmol), and DCM (50 mL). Following workup, the residue was diluted in EtOAc (30 mL), and the mixture was stirred open to the air for 24 h. The reaction was then concentrated under vacuum and purified by MPLC (23–43% EtOAc in hexanes as eluent on a silica gel column) to afford the product (0.172 g, 50%) as a brick red solid (mp = 139–142 °C). ^1^H NMR (400 MHz, CDCl_3_) δ 7.71 (ddd, *J* = 7.6, 1.4, 0.6 Hz, 1H), 7.41–7.27 (m, 6H), 7.16 (d, *J* = 2.7 Hz, 1H), 7.00 (td, *J* = 7.5, 0.8 Hz, 1H), 6.89 (dd, *J* = 8.5, 0.7 Hz, 1H), 6.72 (dd, *J* = 8.5, 2.7 Hz, 1H), 6.61 (d, *J* = 8.1 Hz, 1H), 6.12 (s, 1H), 3.80 (s, 3H); ^13^C­{^1^H} NMR (101 MHz, CDCl_3_) δ 184.9, 160.0, 150.8, 146.0, 142.3, 140.4, 137.5, 129.5, 128.8, 128.5, 127.0, 125.4, 122.6, 120.3, 117.9, 115.9, 113.3, 111.1, 58.6, 55.6; IR (neat): 3058, 2971, 1718, 1595, 1493, 1467, 1320, 1202, 1148 cm^–1^; HRMS (ESI-TOF) *m*/*z*: [M + H]^+^ Calcd for C_22_H_17_N_2_O_2_ 341.1290; found 341.1281.

## Supplementary Material



## Data Availability

The data underlying this study are available in the published article and its Supporting Information.

## Notes

^1^H and ^13^C NMR spectral data of all compounds, X-ray crystal data for compound **6d**, and chiral HPLC data for compounds **5d**, **5e**, **6d**, and **6e** (PDF)
