# Supplementary material for: Tandem Synthesis of Tetrahydropyrroloquinazolines and Related Polyannular Scaffolds
Source: J Org Chem. 2025 Jun 18;90(26):9300–12. doi: 10.1021/acs.joc.5c00996 (PMC12235636; doi:10.1021/acs.joc.5c00996)
Supplement: Supplementary file 1 [file jo5c00996_si_001.pdf]

Supporting Information

Tandem Synthesis of Tetrahydropyrroloquinazolines and Related Polyannular Scaffolds

Haley M. Carlson and R. Adam Mosey*

Department of Chemistry, Lake Superior State University, Sault Sainte Marie, MI 49783
*rmosey@lssu.edu

Contents Page #

X-Ray Crystal Data for **6c** S2
Kinetic NMR Data (Synthesis of **9**) S3
Chiral HPLC Data for compounds **5d** and **5e** S4
Chiral HPLC Data for compounds **6d** and **6e** S5
References S5
NMR Spectra S6

(6S,9R,11R)-3-methoxy-11-phenyl-6,8,9,11-tetrahydro-7H-6,9-methanopyrido[2,1-b]quinazoline (6d): Single colourless irregular-shaped crystals of **6d** were used as received. The crystals were grown from benzene and dichloromethane via slow evaporation. A suitable crystal with dimensions 0.17 × 0.15 × 0.09 mm^3 was selected and mounted on a nylon loop with paratone oil on a XtaLAB Synergy, Dualflex, HyPix diffractometer. The crystal was kept at a steady T = 100.00(10) K during data collection. The structure was solved with the ShelXT[1] solution program using dual methods and by using Olex2[2] as the graphical interface. The model was refined with ShelXL[3] using full matrix least squares minimization on F^2.

The crystal structure was deposited into the CCDC database (CCDC No. 2422307).

Table S2. Crystal data and structure refinement for **6d**.

Compound	6d
Formula	$C_{20}H_{20}N_2O$
$D_{calc.}$/ g cm^{-3}	1.301
μ/mm^{-1}	0.634
Formula Weight	304.38
Color	colourless
Shape	irregular-shaped
Size/mm^3	0.17×0.15×0.09
T/K	100.00(10)
Crystal System	orthorhombic
Flack Parameter	-0.09(17)
Hooft Parameter	-0.09(17)
Space Group	$P2_12_12_1$
a/Å	9.3751(2)
b/Å	10.0361(2)
c/Å	16.5108(3)
α/°	90
β/°	90
γ/°	90
V/Å^3	1553.49(5)
Z	4
Z'	1
Wavelength/Å	1.54184
Radiation type	Cu K$_\alpha$
Θ_{min}/°	5.157
Θ_{max}/°	79.772
Measured Refl's.	10137
Indep't Refl's	3304
Refl's I≥2 σ(I)	3105
R_{int}	0.0379
Parameters	209
Restraints	0
Largest Peak	0.194
Deepest Hole	-0.216
GooF	1.058
wR_2 (all data)	0.1002
wR_2	0.0981
R_1 (all data)	0.0393
R_1	0.0370

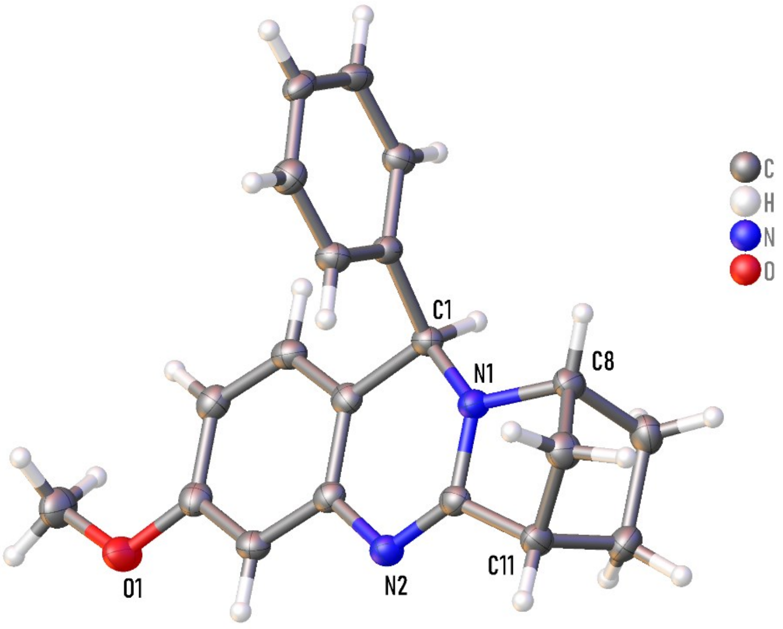

Figure S1. Crystal structure of compound **6d** with drawing at 50% ellipsoids

Kinetic NMR Spectra: Synthesis of Compound 9. See experimental procedure for the synthesis of compound **9**. Initial ^{1}H and ^{13}C NMR spectra were obtained of the crude reaction after being under vacuum for 24 hours before being diluted in EtOAc. The reaction was then sampled 3, 6, and 24 hours later. Each EtOAc sample (~0.25 mL aliquot) was concentrated under vacuum and then diluted in $CDCl_3$ (0.7 mL) prior to obtaining a ^{1}H NMR and/or ^{13}C spectrum.

^{1}H NMR Spectra:

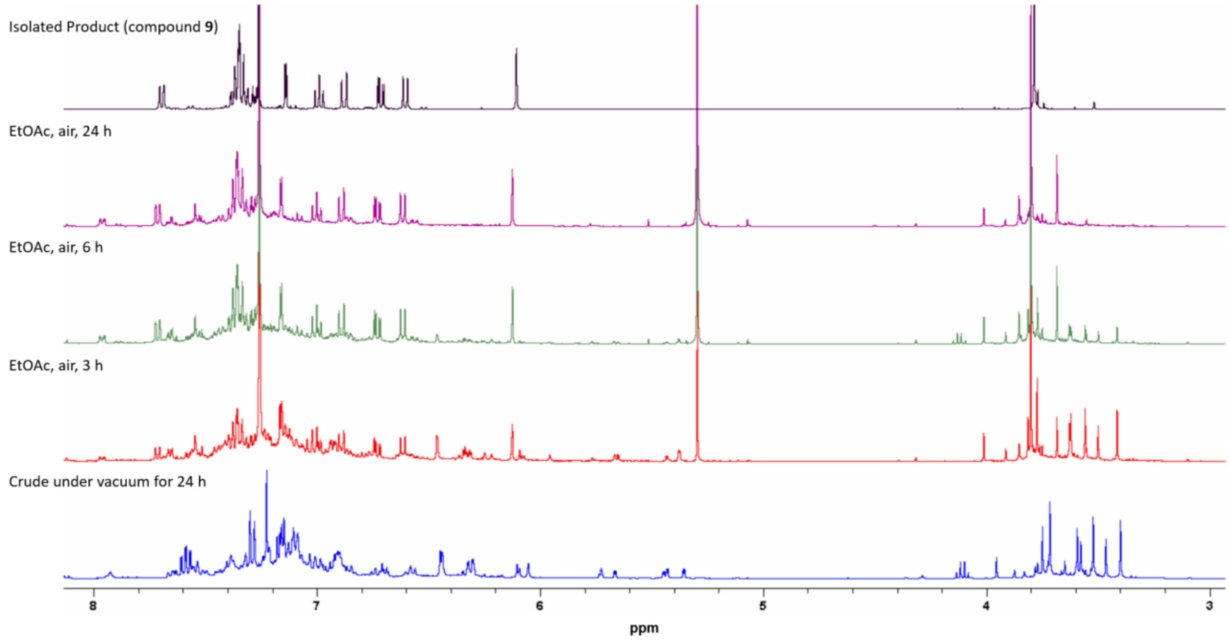

^{13}C NMR Spectra:

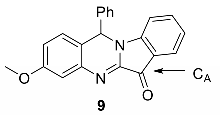

9

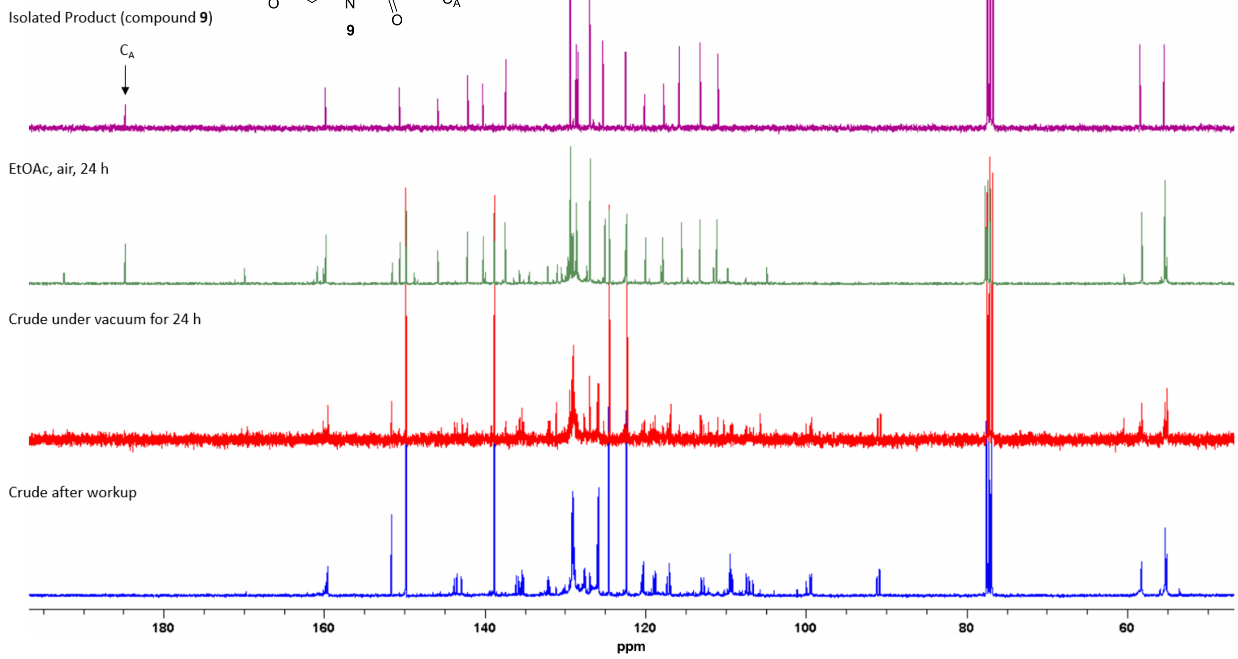

Chiral HPLC Data for Compounds 5d and 5e
HPLC analysis: Chiralpak IE-3, hexanes/*i*-PrOH/(1% DEA in EtOH) = 70:20:10, 1.5 mL/min, 30 °C, detection at 254 nm

Chiral HPLC chromatogram for **5d**

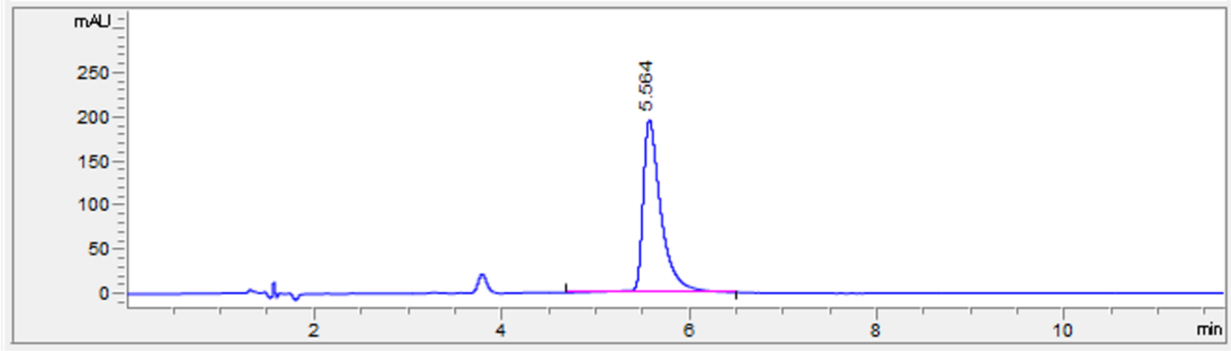

Chiral HPLC chromatogram for **5e**

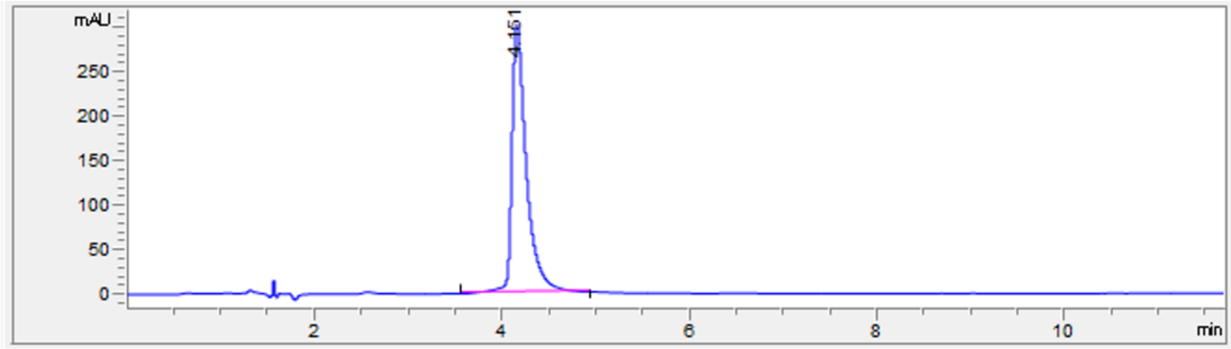

Chiral HPLC Data for Compounds 6d and 6e

HPLC analysis: Chiralpak IE-3, hexanes/*i*-PrOH/(1% DEA in EtOH) = 90:7:3, 1.5 mL/min, 30 °C, detection at 254 nm

Chiral HPLC chromatogram for **6d**

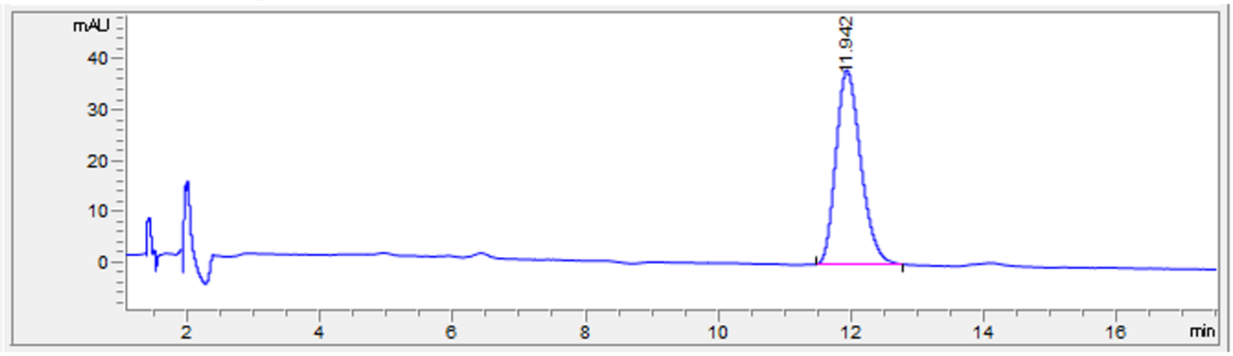

Chiral HPLC chromatogram for **6e**

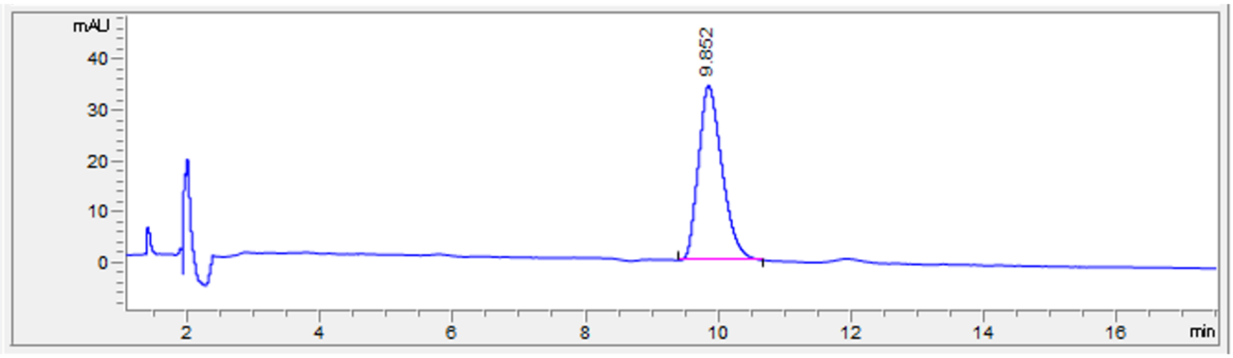

Chiral HPLC chromatogram for racemic **6d**/**6e**

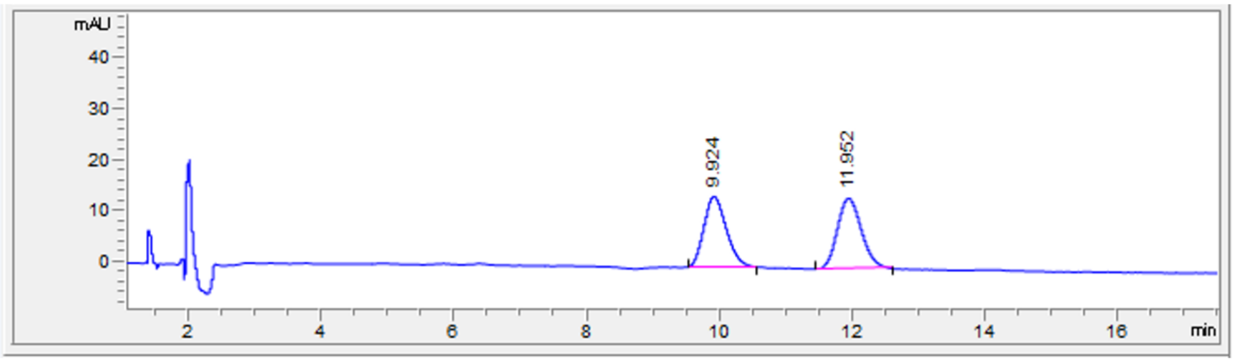

References

1. Sheldrick, G. M. SHELXT– Integrated Space-Group and Crystal-Structure Determination. *Acta Cryst.* **2015**, *A71*, 3–8.
2. Dolomanov, O. V.; Bourhis, L. J.; Gildea, R. J.; Howard, J. A. K.; Puschmann, H. OLEX2: A Complete Structure Solution, Refinement and Analysis Program. *J. Appl. Cryst.* **2009**, *42*, 339–341.
3. Sheldrick, G. M. A short history of SHELX. *Acta Cryst.* **2008**, *A64*, 112-122.

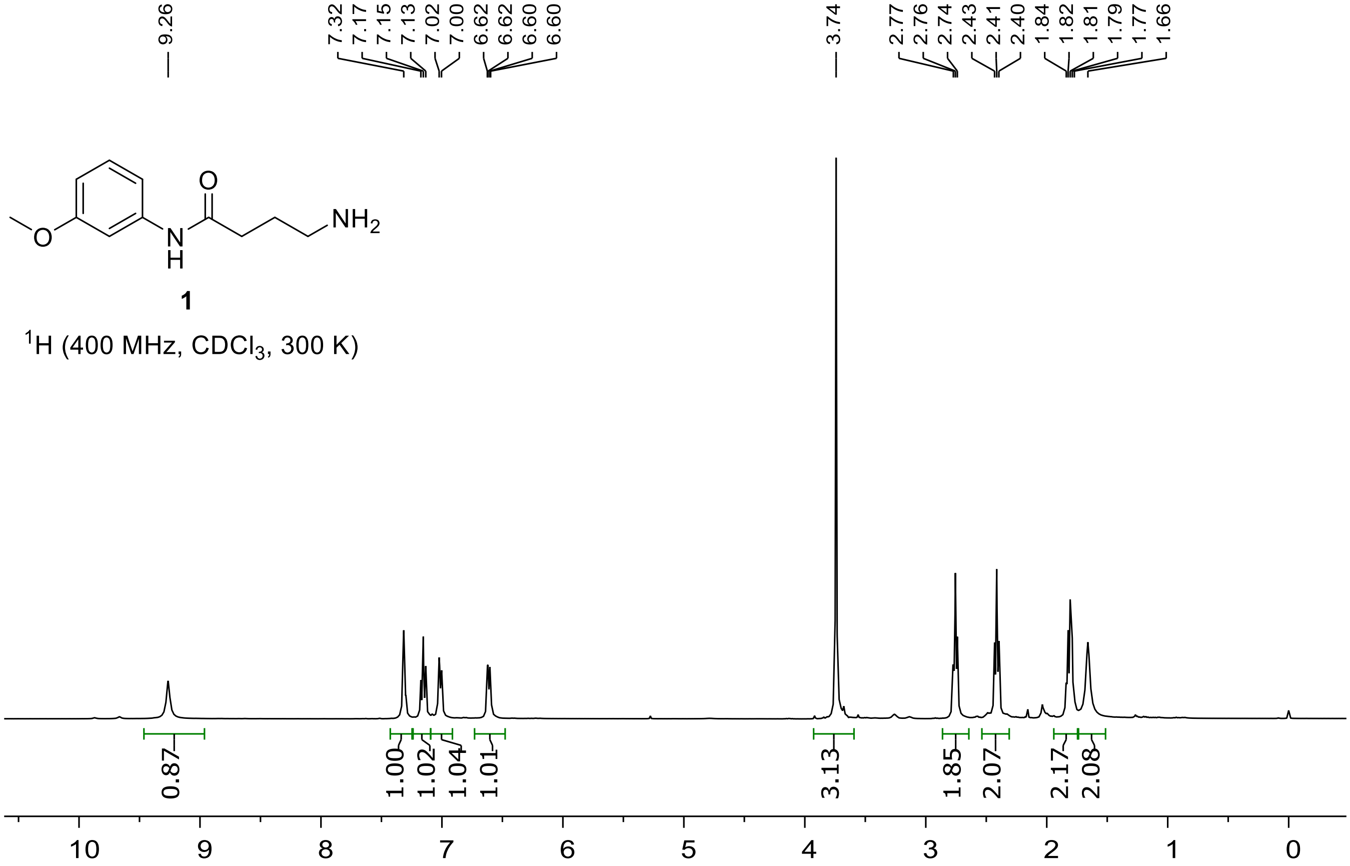
9.26
7.32 7.17 7.15 7.13 7.02 7.00 6.62 6.62 6.60 6.60
3.74
2.77 2.76 2.74 2.43 2.41 2.40 1.84 1.82 1.81 1.79 1.77 1.66
O
O
N
H
NH2
1
1H (400 MHz, CDCl3, 300 K)
0.87
1.00 1.02 1.04 1.01
3.13
1.85 2.07 2.17 2.08
10 9 8 7 6 5 4 3 2 1 0

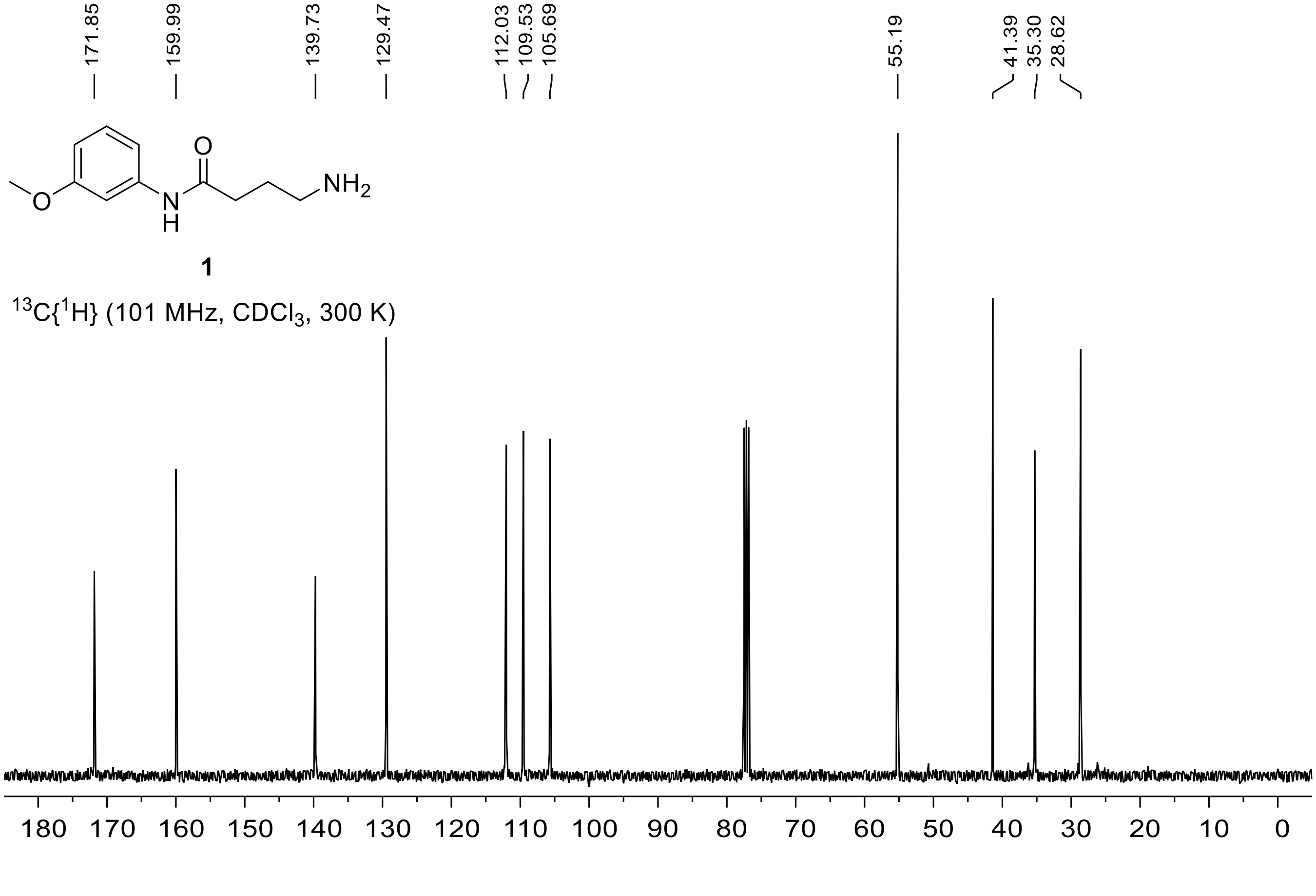
171.85
159.99
139.73
129.47
112.03 109.53 105.69
55.19
41.39 35.30 28.62
O
O
N
H
NH2
1
13C{1H} (101 MHz, CDCl3, 300 K)
180 170 160 150 140 130 120 110 100 90 80 70 60 50 40 30 20 10 0

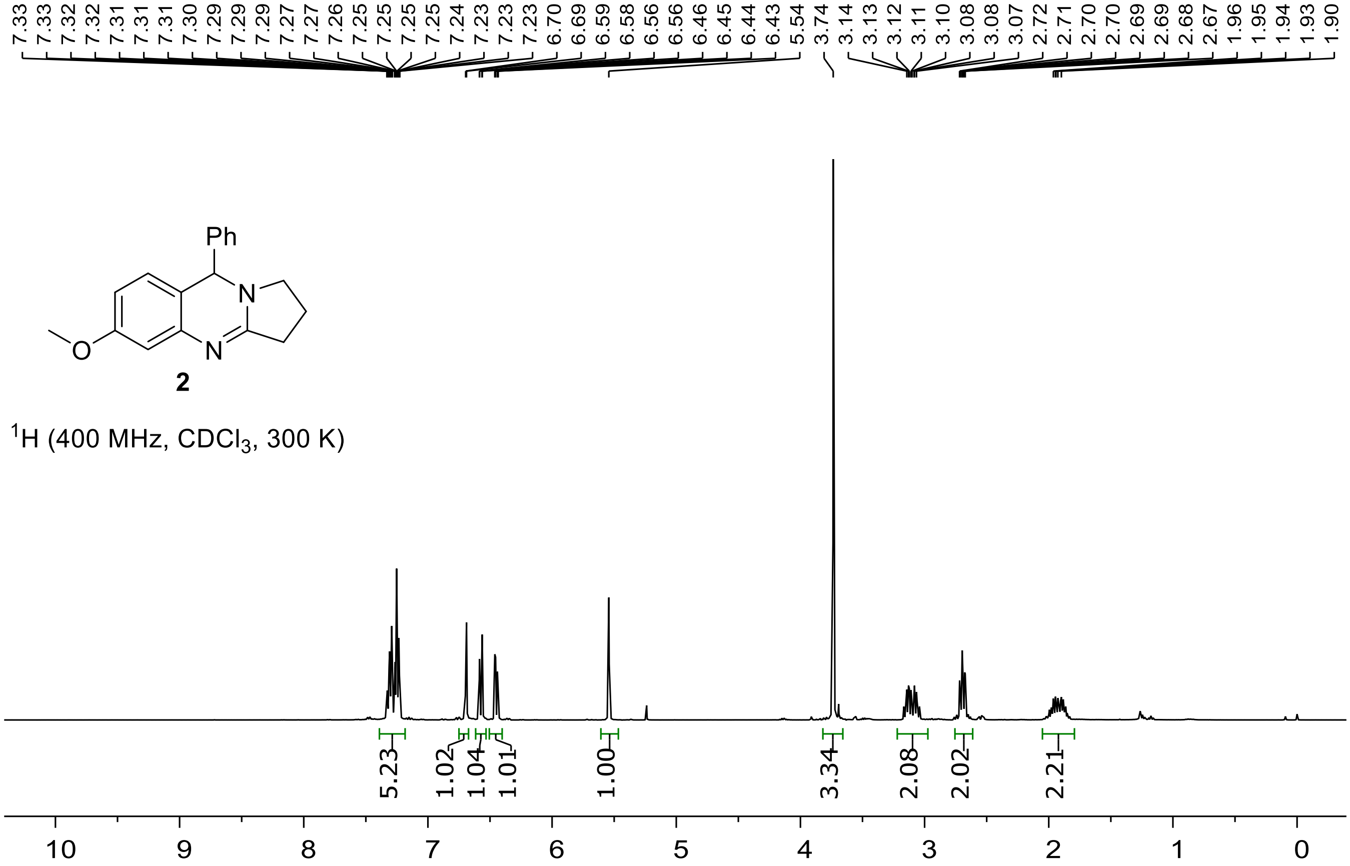
7.33 7.33 7.32 7.32 7.31 7.31 7.31 7.30 7.29 7.29 7.29 7.27 7.26 7.25 7.25 7.25 7.25 7.24 7.23 7.23 7.23 6.70 6.69 6.59 6.58 6.56 6.56 6.46 6.45 6.44 6.43 5.54 3.74 3.14 3.13 3.12 3.11 3.10 3.08 3.08 3.07 2.72 2.71 2.70 2.70 2.69 2.69 2.68 2.67 1.96 1.95 1.94 1.93 1.90
Ph
N
O
N
2
^{1}H (400 MHz, CDCl$_3$, 300 K)
5.23 1.02 1.04 1.01 1.00 3.34 2.08 2.02 2.21
10 9 8 7 6 5 4 3 2 1 0

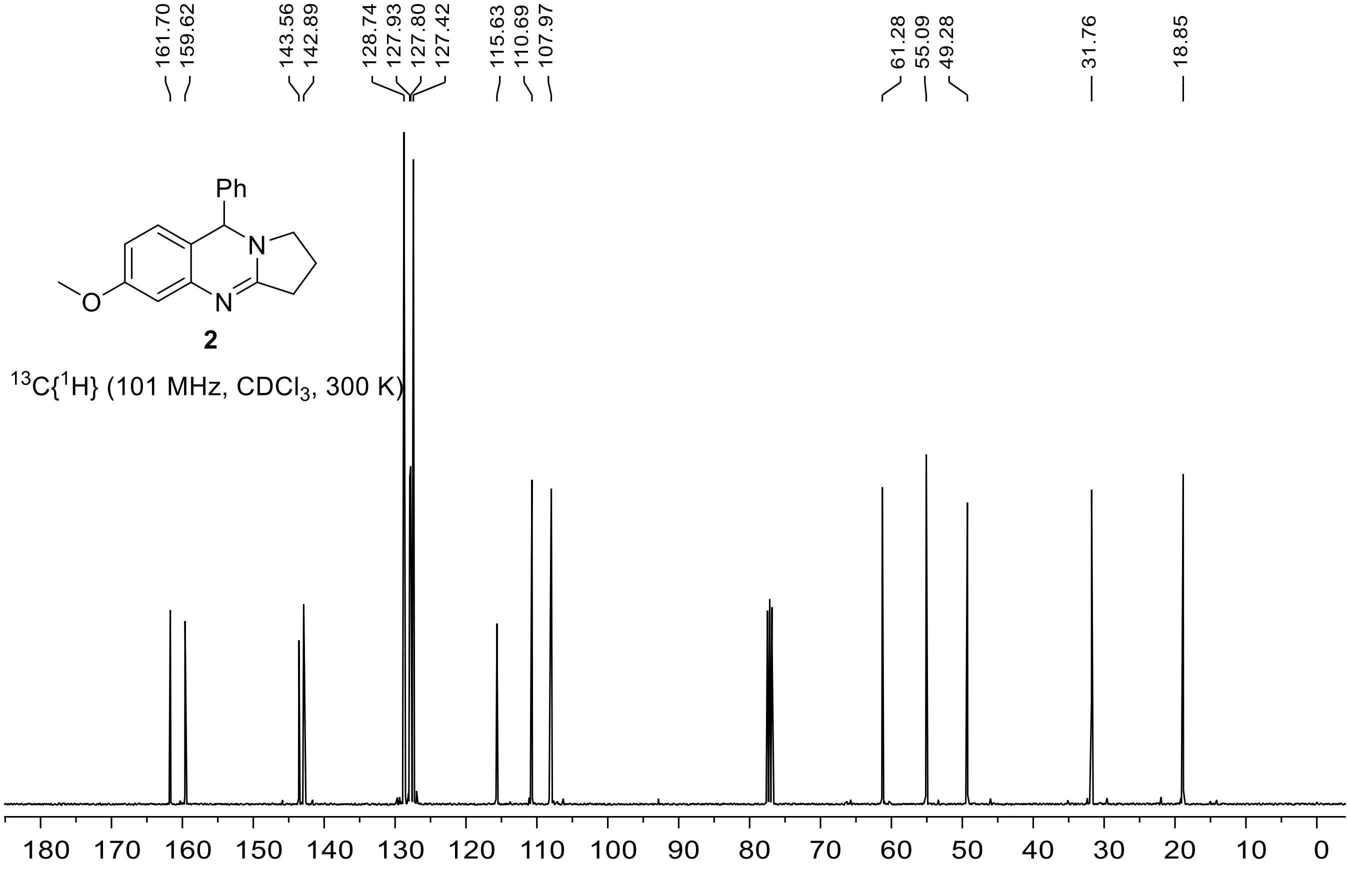
161.70 159.62 143.56 142.89 128.74 127.93 127.80 127.42 115.63 110.69 107.97 61.28 55.09 49.28 31.76 18.85
Ph
N
O
N
2
^{13}C{^{1}H} (101 MHz, CDCl$_3$, 300 K)
180 170 160 150 140 130 120 110 100 90 80 70 60 50 40 30 20 10 0

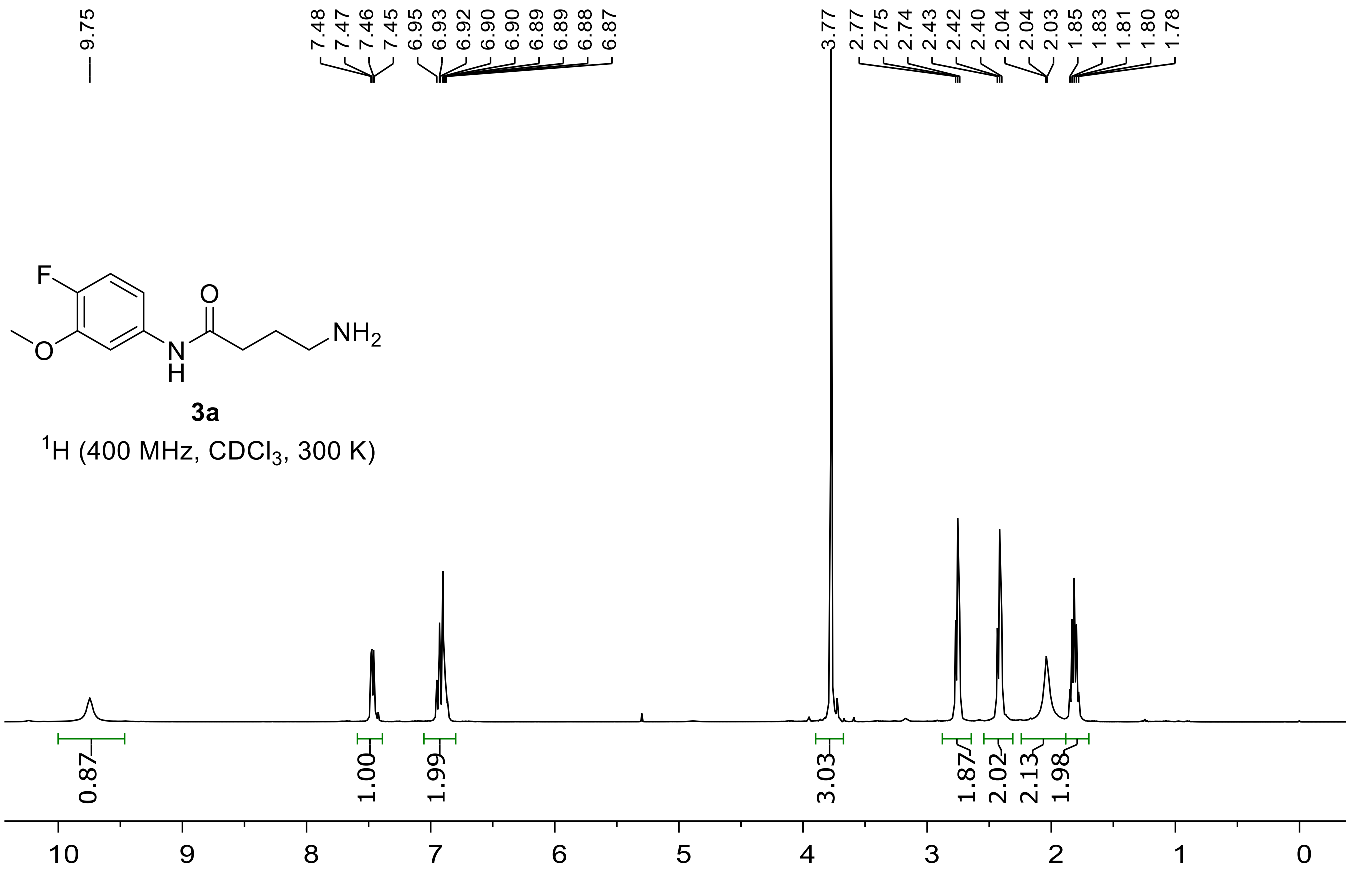
9.75
7.48
7.47
7.46
7.45
6.95
6.93
6.92
6.90
6.90
6.89
6.89
6.88
6.87
3.77
2.77
2.75
2.74
2.43
2.42
2.40
2.04
2.04
2.03
1.85
1.83
1.81
1.80
1.78
F
O
N
H
O
NH2
3a
1H (400 MHz, CDCl3, 300 K)
0.87
1.00
1.99
3.03
1.87
2.02
2.13
1.98
10
9
8
7
6
5
4
3
2
1
0

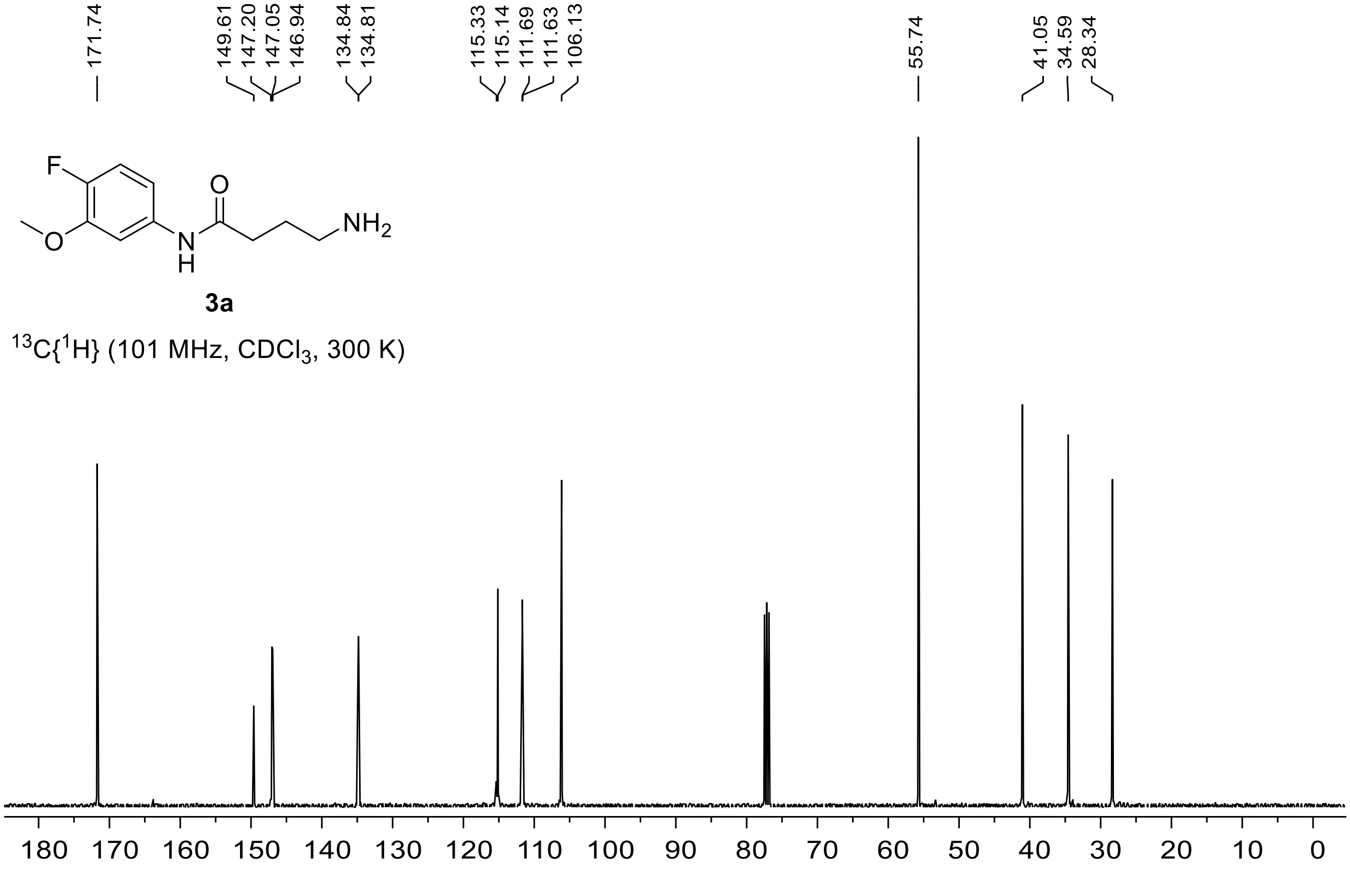
171.74
149.61
147.20
147.05
146.94
134.84
134.81
115.33
115.14
111.69
111.63
106.13
55.74
41.05
34.59
28.34
F
O
N
H
O
NH2
3a
13C{1H} (101 MHz, CDCl3, 300 K)
180
170
160
150
140
130
120
110
100
90
80
70
60
50
40
30
20
10
0

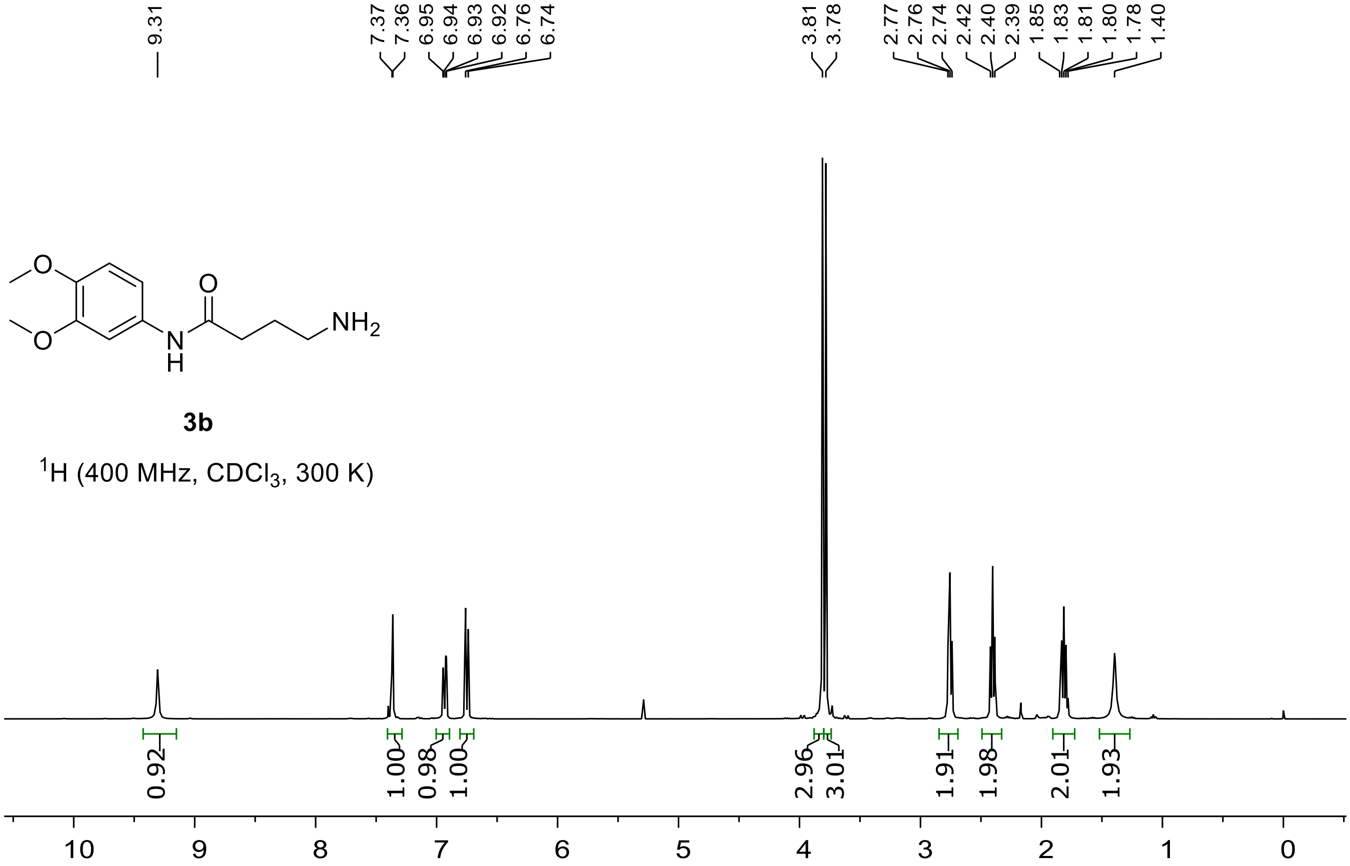
9.31
7.37 7.36 6.95 6.94 6.93 6.92 6.76 6.74
3.81 3.78
2.77 2.76 2.74 2.42 2.40 2.39 1.85 1.83 1.81 1.80 1.78 1.40
O
NH2
N
H
3b
1H (400 MHz, CDCl3, 300 K)
0.92 1.00 0.98 1.00 2.96 3.01 1.91 1.98 2.01 1.93
10 9 8 7 6 5 4 3 2 1 0

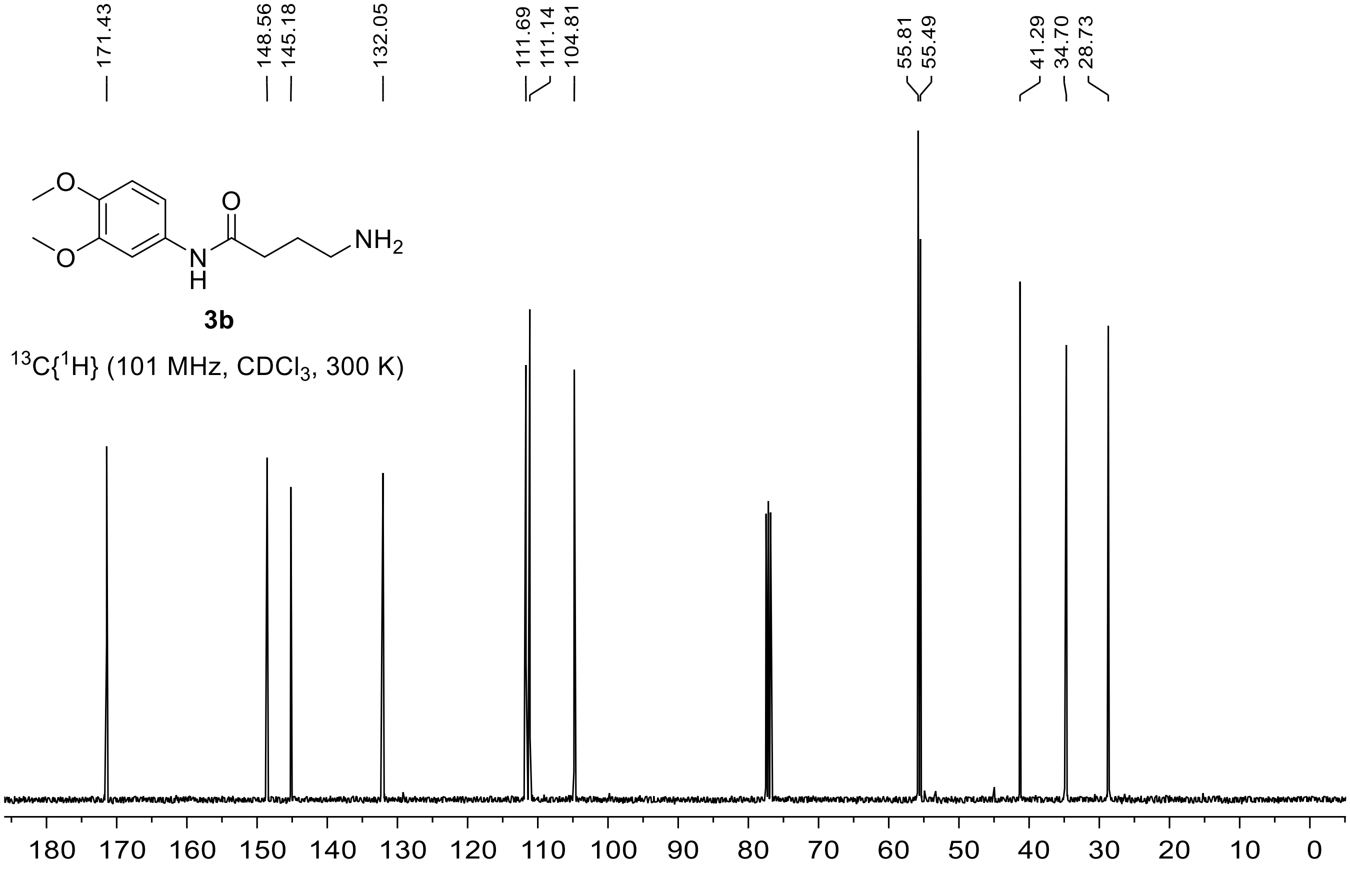
171.43
148.56 145.18
132.05
111.69 111.14 104.81
55.81 55.49
41.29 34.70 28.73
O
NH2
N
H
3b
13C{1H} (101 MHz, CDCl3, 300 K)
180 170 160 150 140 130 120 110 100 90 80 70 60 50 40 30 20 10 0

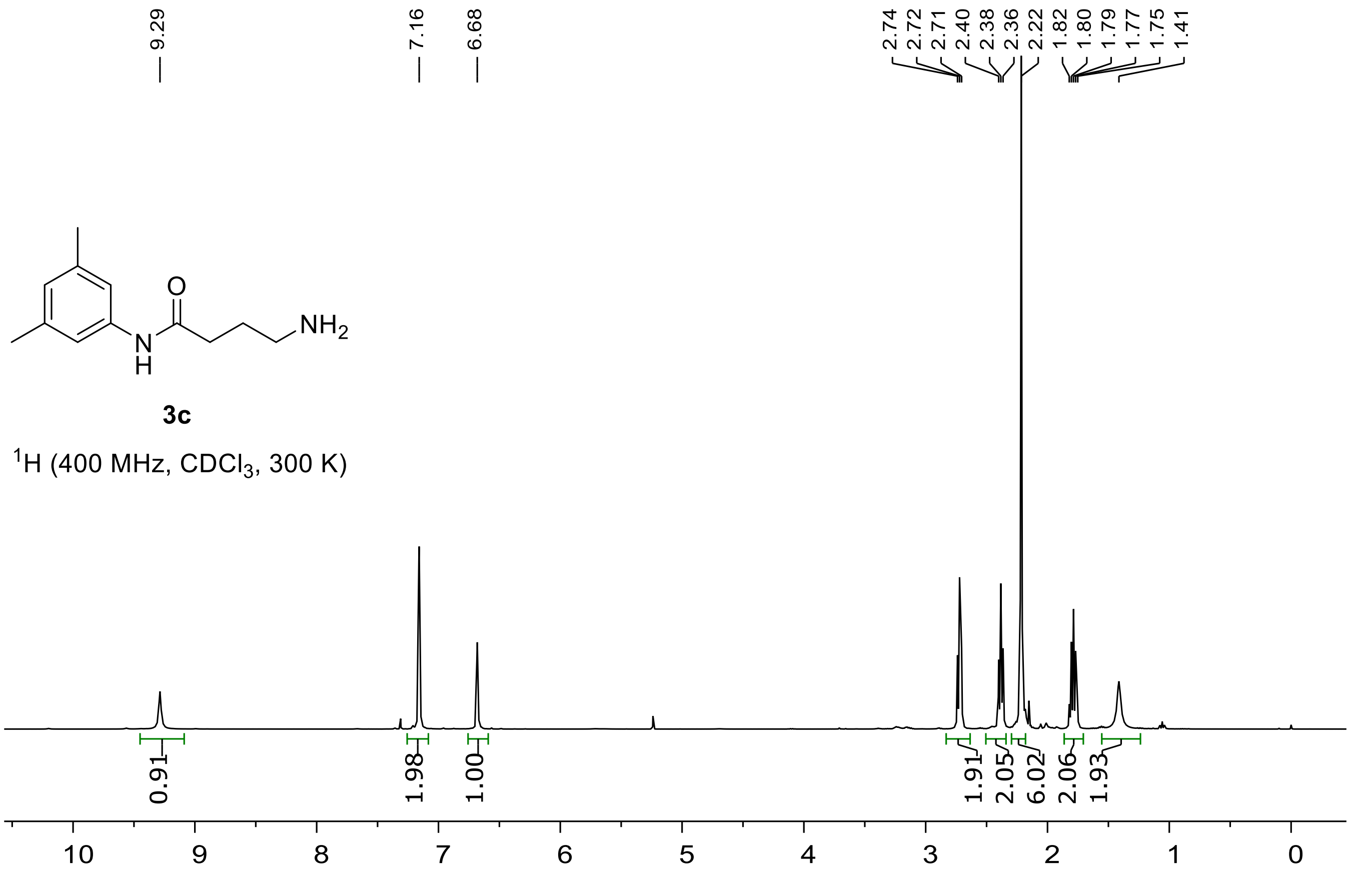
9.29
7.16
6.68
2.74
2.72
2.71
2.40
2.38
2.36
2.22
1.82
1.80
1.79
1.77
1.75
1.41
O
N
H
NH2
3c
1H (400 MHz, CDCl3, 300 K)
0.91
1.98
1.00
1.91
2.05
6.02
2.06
1.93
10
9
8
7
6
5
4
3
2
1
0

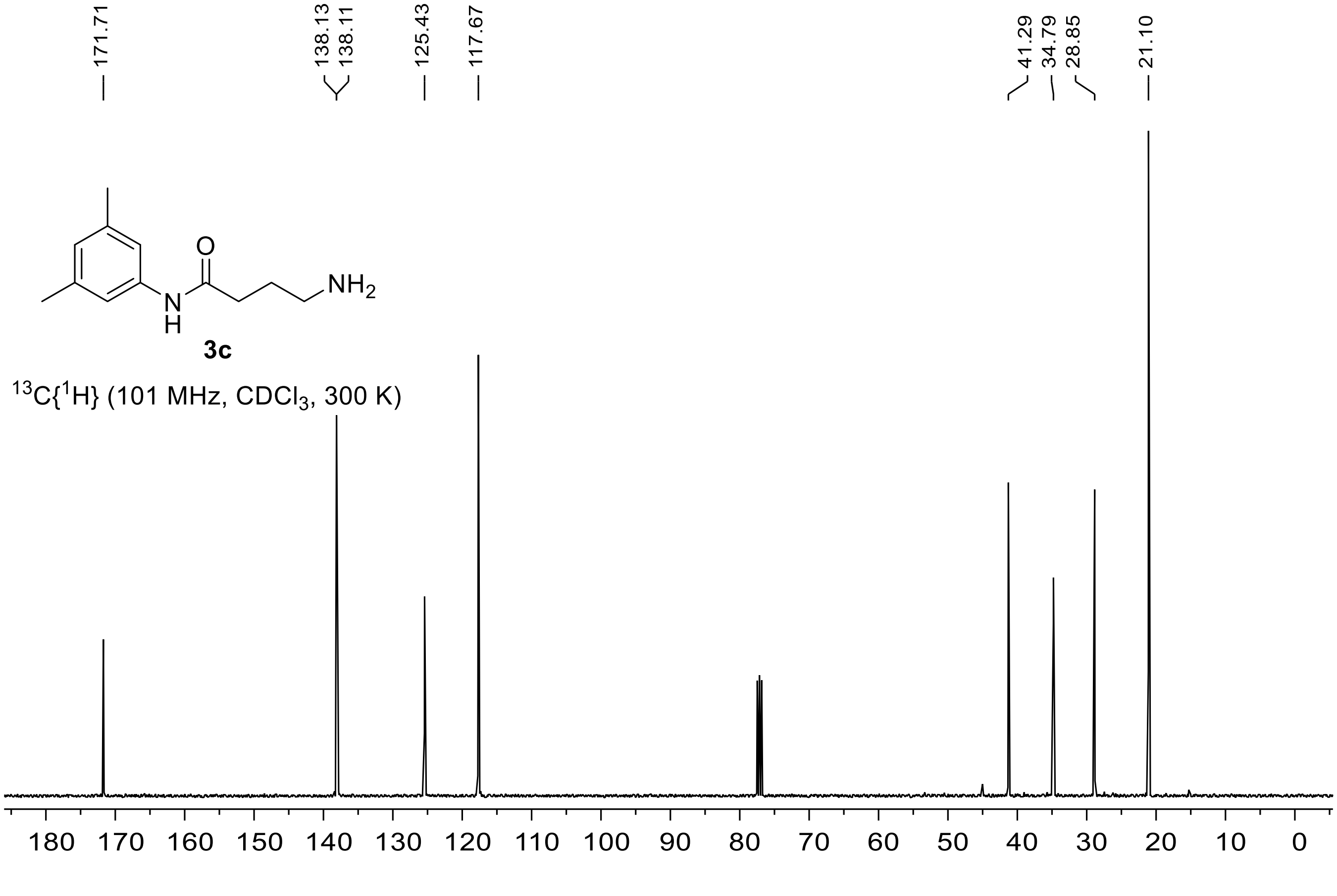
171.71
138.13
138.11
125.43
117.67
41.29
34.79
28.85
21.10
O
N
H
NH2
3c
13C{1H} (101 MHz, CDCl3, 300 K)
180
170
160
150
140
130
120
110
100
90
80
70
60
50
40
30
20
10
0

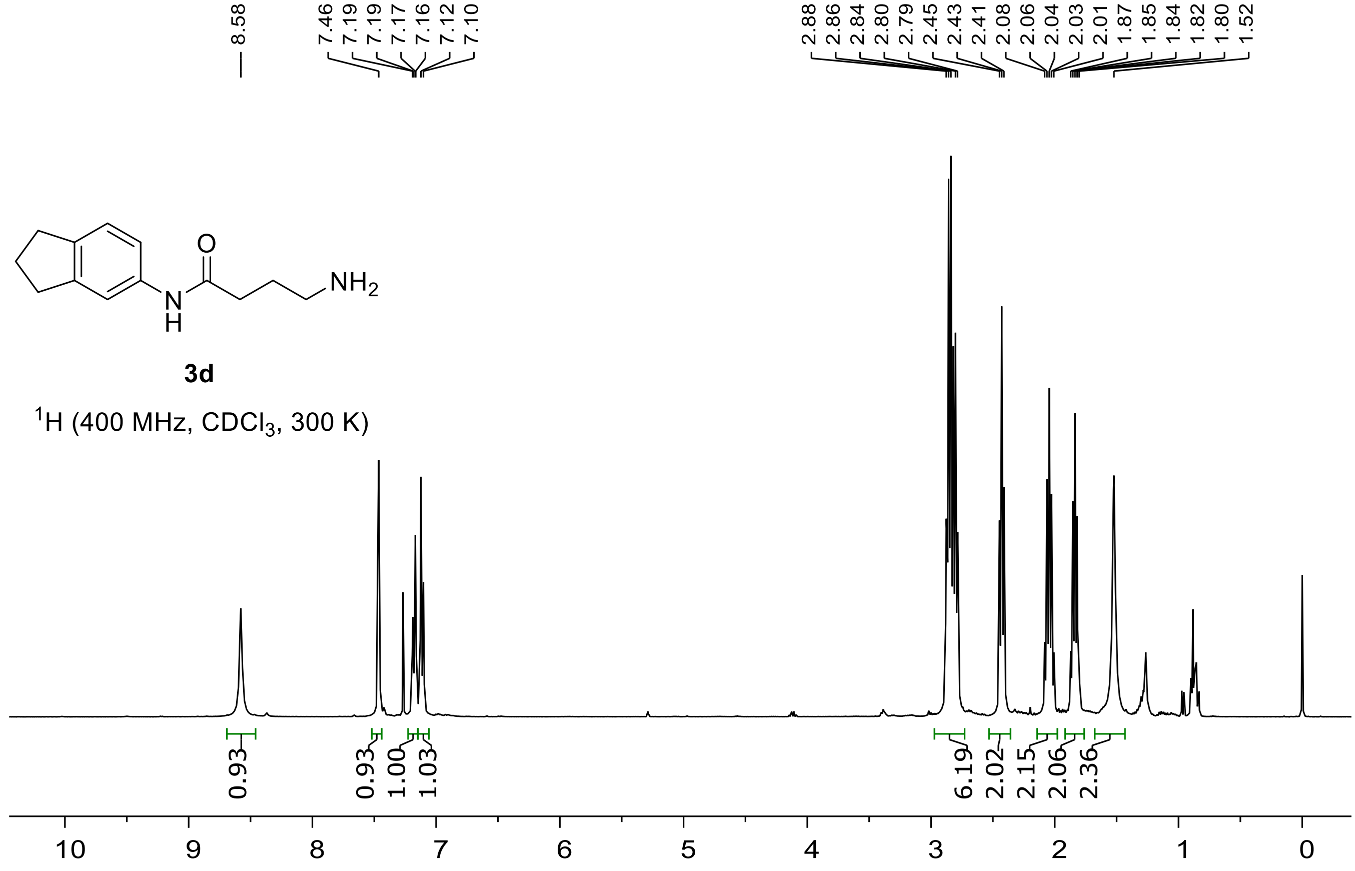

8.58
7.46 7.19 7.19 7.17 7.16 7.12 7.10
2.88 2.86 2.84 2.80 2.79 2.45 2.43 2.41 2.08 2.06 2.04 2.03 2.01 1.87 1.85 1.84 1.82 1.80 1.52
O
N
H
NH2
3d
1H (400 MHz, CDCl3, 300 K)
0.93 0.93 1.00 1.03 6.19 2.02 2.15 2.06 2.36
10 9 8 7 6 5 4 3 2 1 0

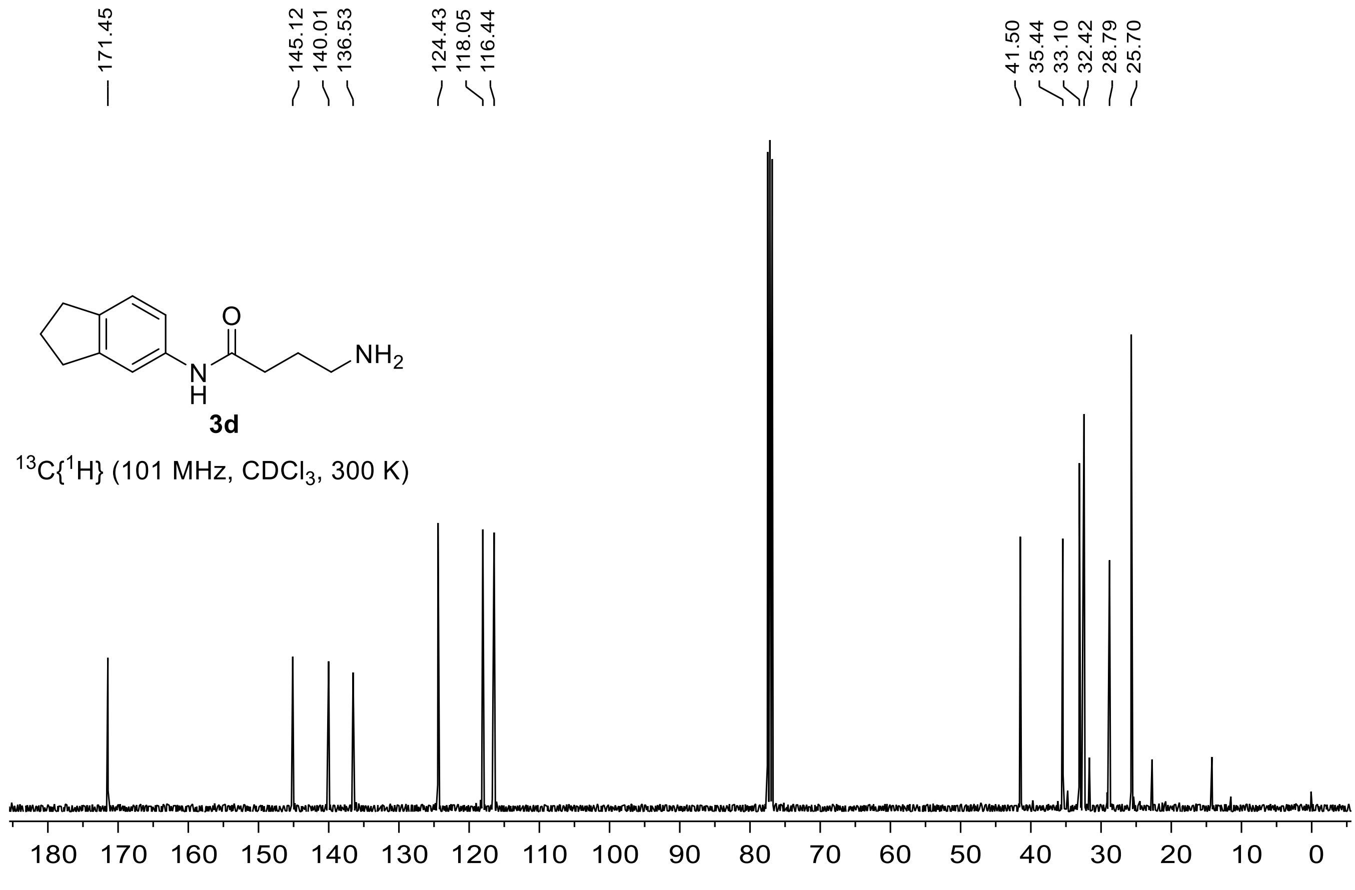

171.45
145.12 140.01 136.53
124.43 118.05 116.44
41.50 35.44 33.10 32.42 28.79 25.70
O
N
H
NH2
3d
13C{1H} (101 MHz, CDCl3, 300 K)
180 170 160 150 140 130 120 110 100 90 80 70 60 50 40 30 20 10 0

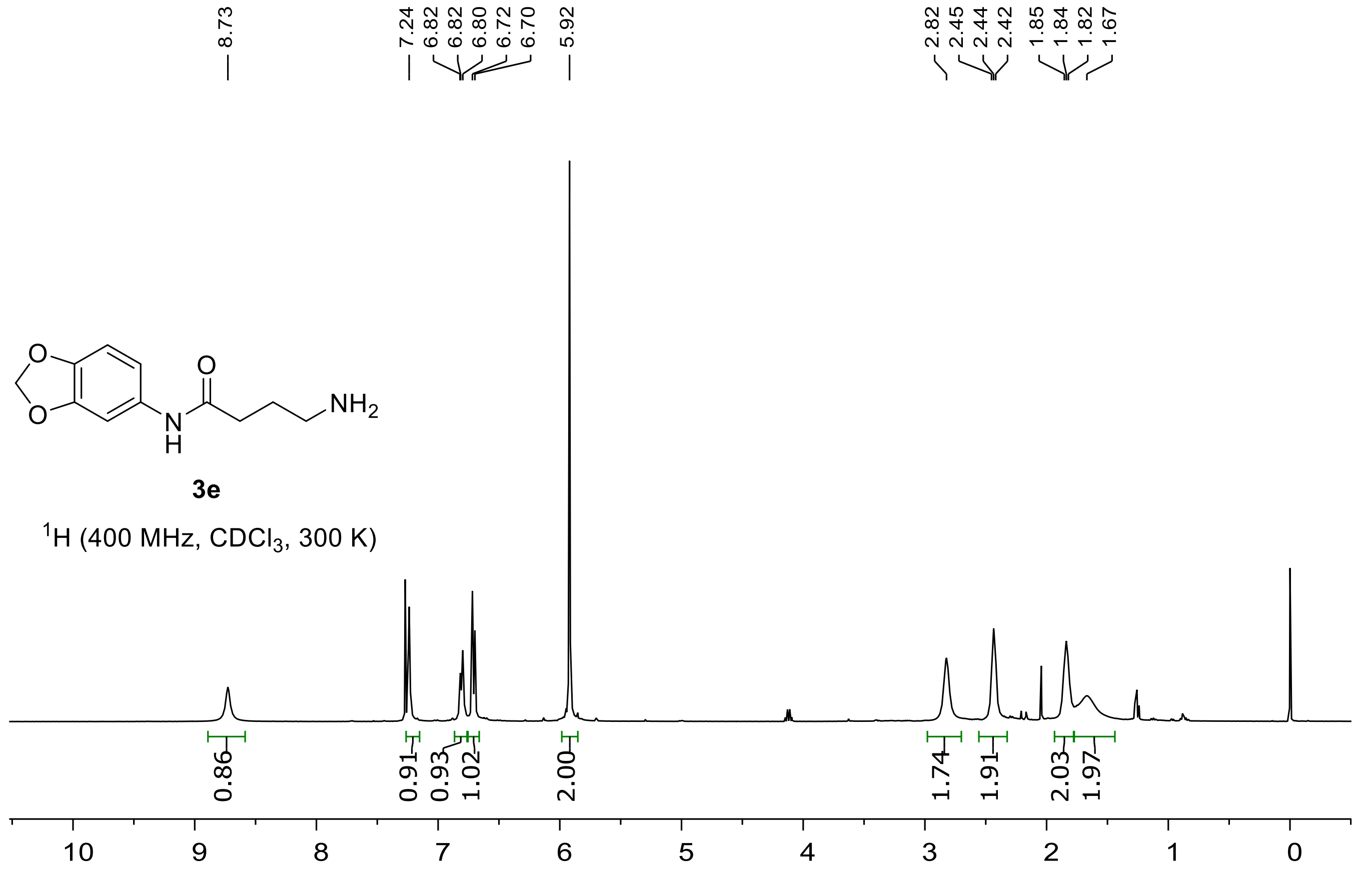
8.73
7.24
6.82
6.82
6.80
6.72
6.70
5.92
2.82
2.45
2.44
2.42
1.85
1.84
1.82
1.67
O
O
O
N
H
NH2
3e
1H (400 MHz, CDCl3, 300 K)
0.86
0.91
0.93
1.02
2.00
1.74
1.91
2.03
1.97
10 9 8 7 6 5 4 3 2 1 0

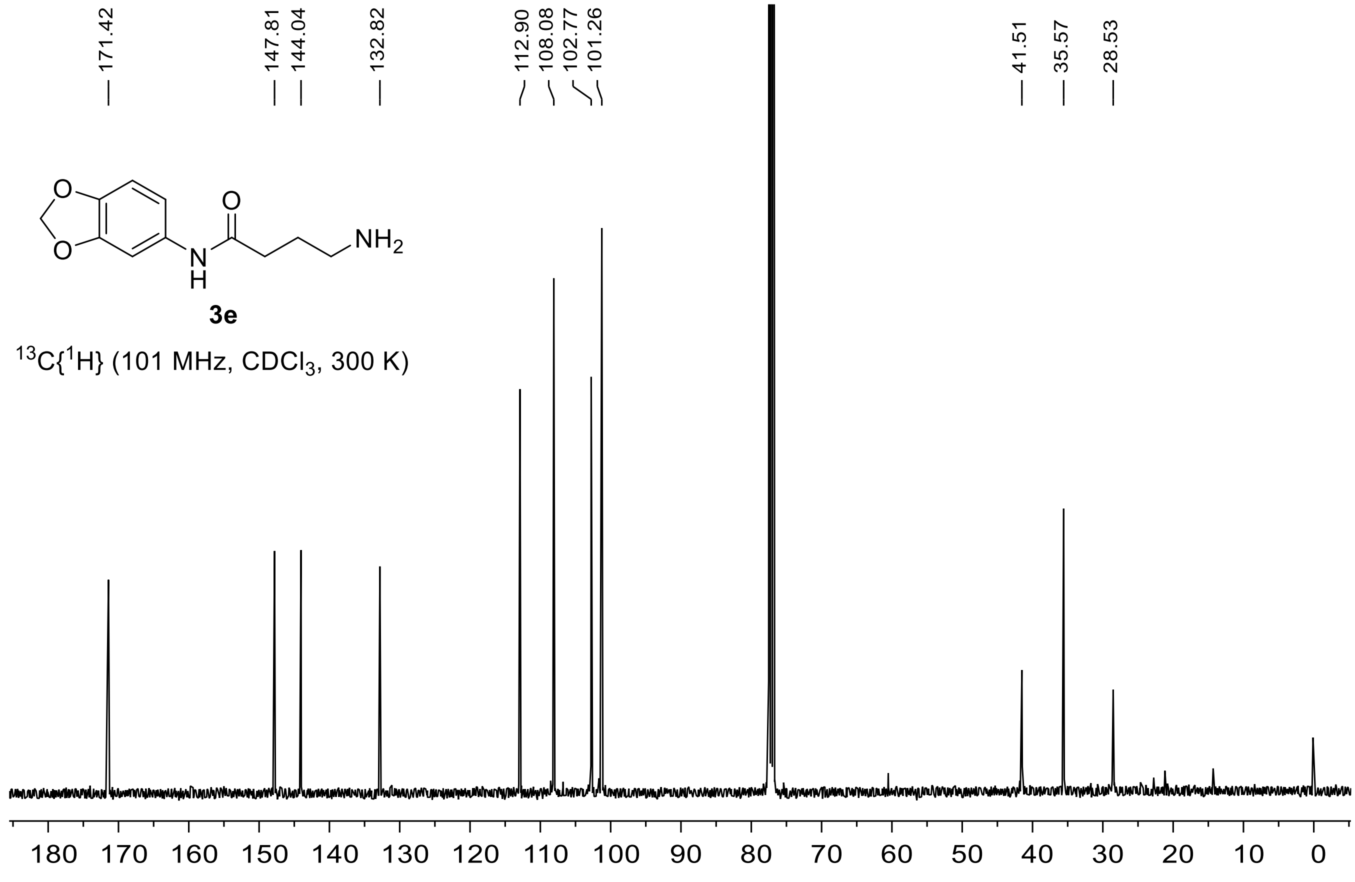
171.42
147.81
144.04
132.82
112.90
108.08
102.77
101.26
41.51
35.57
28.53
O
O
O
N
H
NH2
3e
13C{1H} (101 MHz, CDCl3, 300 K)
180 170 160 150 140 130 120 110 100 90 80 70 60 50 40 30 20 10 0

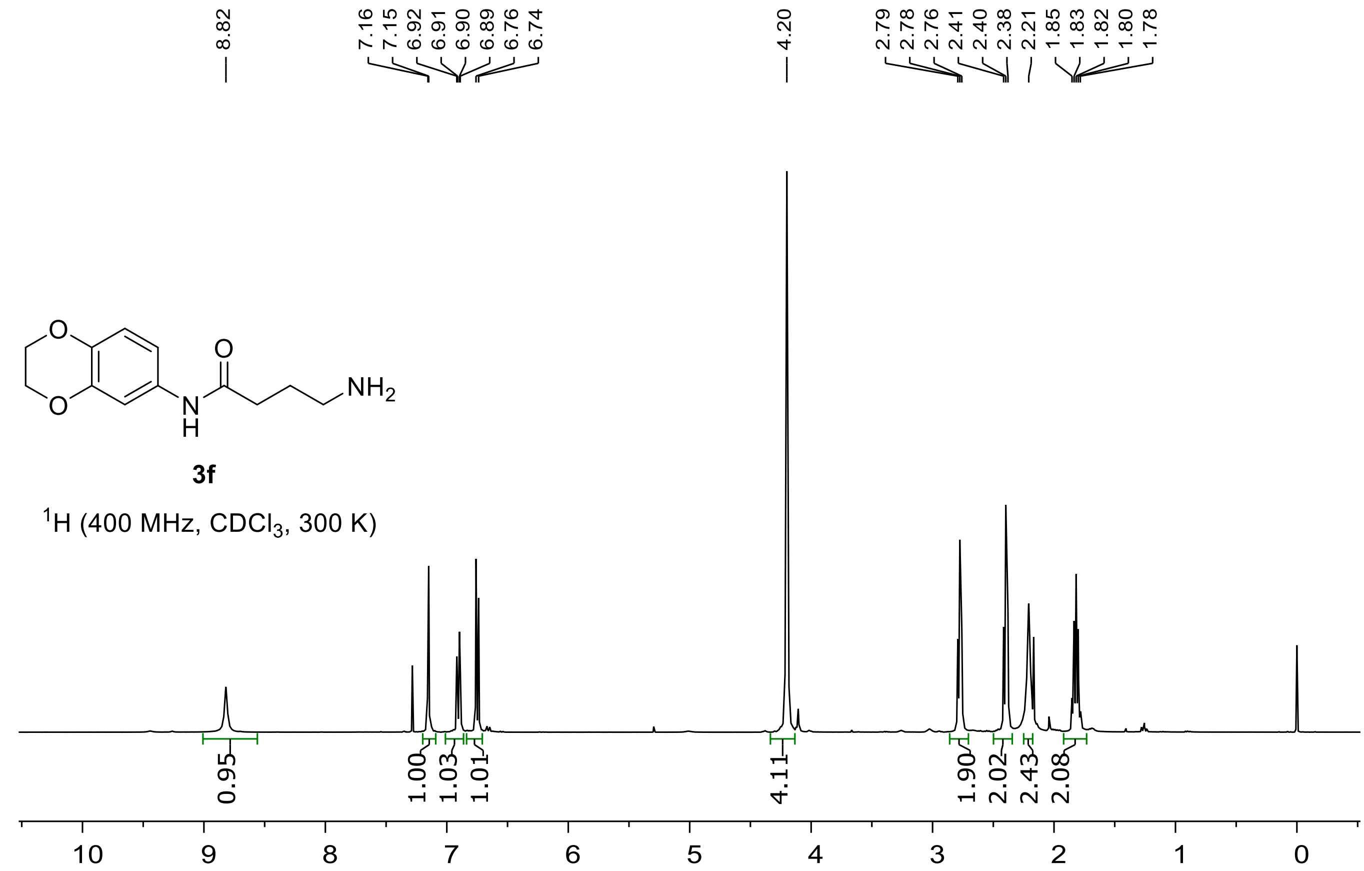
8.82
7.16 7.15 6.92 6.91 6.90 6.89 6.76 6.74
4.20
2.79 2.78 2.76 2.41 2.40 2.38 2.21 1.85 1.83 1.82 1.80 1.78
O
O
O
N
H
NH2
3f
1H (400 MHz, CDCl3, 300 K)
0.95
1.00
1.03
1.01
4.11
1.90
2.02
2.43
2.08
10 9 8 7 6 5 4 3 2 1 0

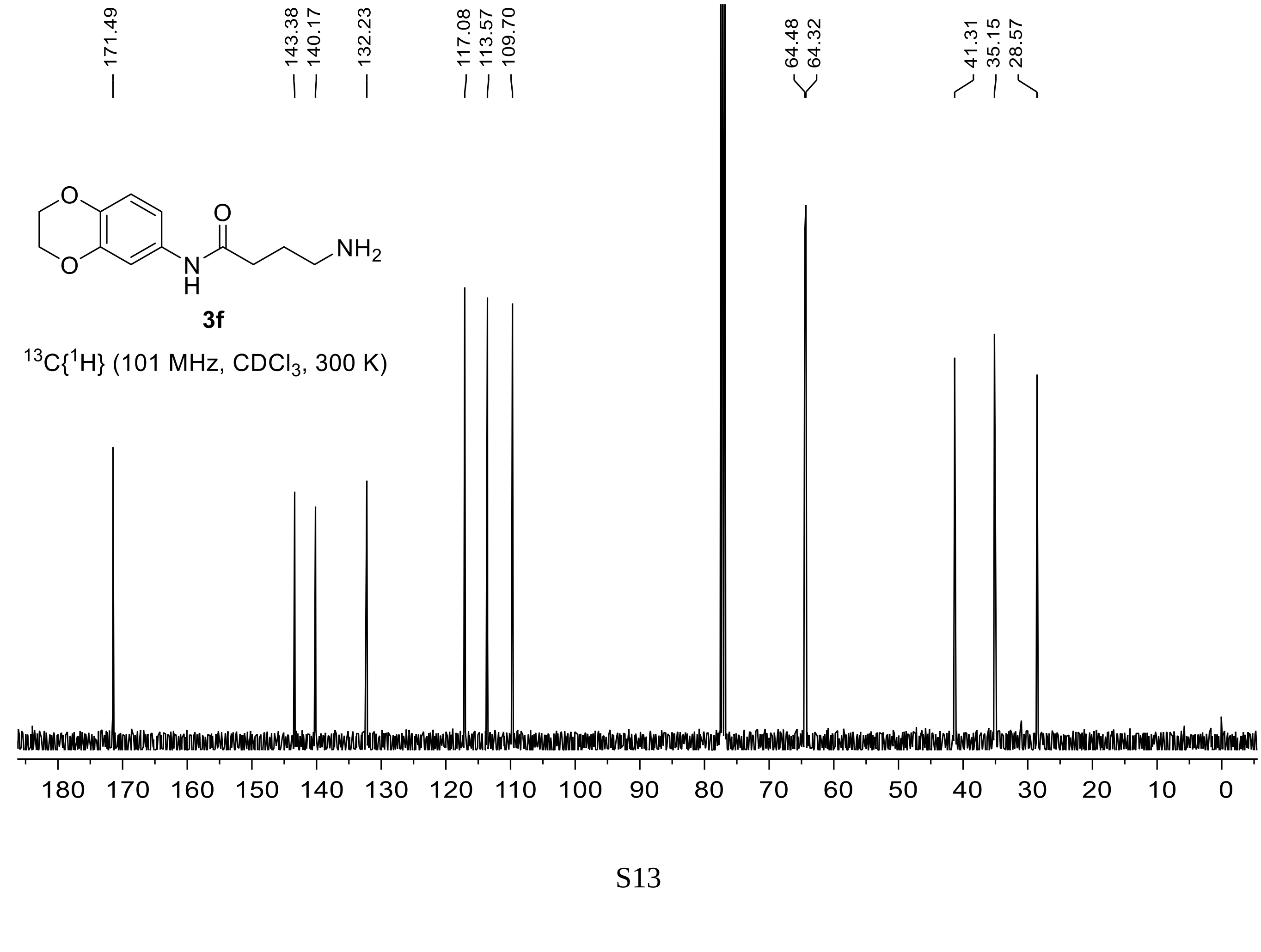
171.49
143.38 140.17
132.23
117.08 113.57 109.70
64.48 64.32
41.31 35.15 28.57
O
O
O
N
H
NH2
3f
13C{1H} (101 MHz, CDCl3, 300 K)
180 170 160 150 140 130 120 110 100 90 80 70 60 50 40 30 20 10 0

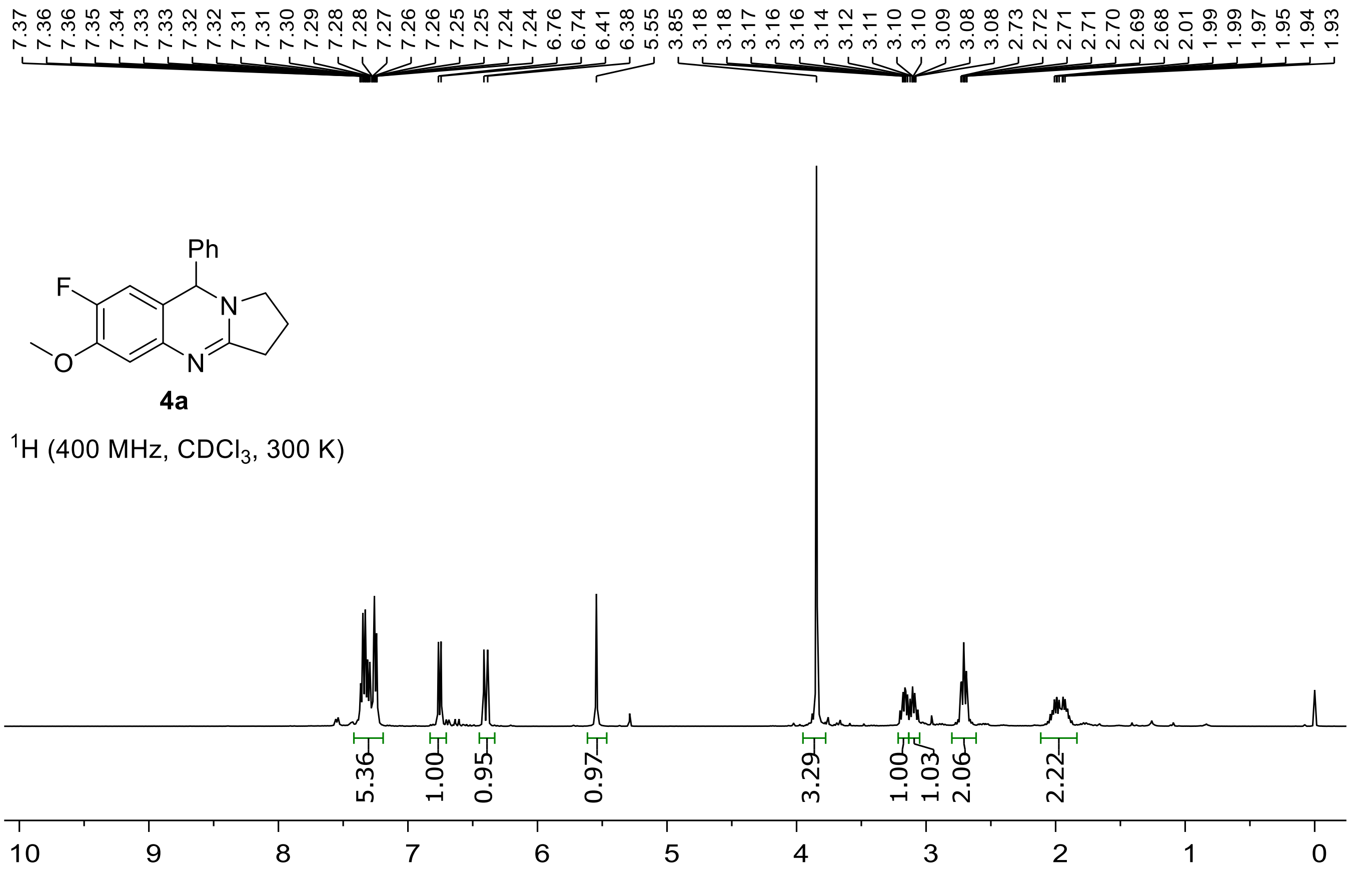
7.37 7.36 7.36 7.35 7.34 7.33 7.33 7.32 7.32 7.31 7.31 7.30 7.29 7.28 7.28 7.27 7.26 7.26 7.25 7.25 7.24 7.24 6.76 6.74 6.41 6.38 5.55 3.85 3.18 3.18 3.17 3.16 3.16 3.14 3.12 3.11 3.10 3.10 3.09 3.08 3.08 2.73 2.72 2.71 2.71 2.70 2.69 2.68 2.01 1.99 1.99 1.97 1.95 1.94 1.93
Ph
F
N
O
N
4a
^{1}H (400 MHz, CDCl$_3$, 300 K)
5.36
1.00
0.95
0.97
3.29
1.00
1.03
2.06
2.22
10 9 8 7 6 5 4 3 2 1 0

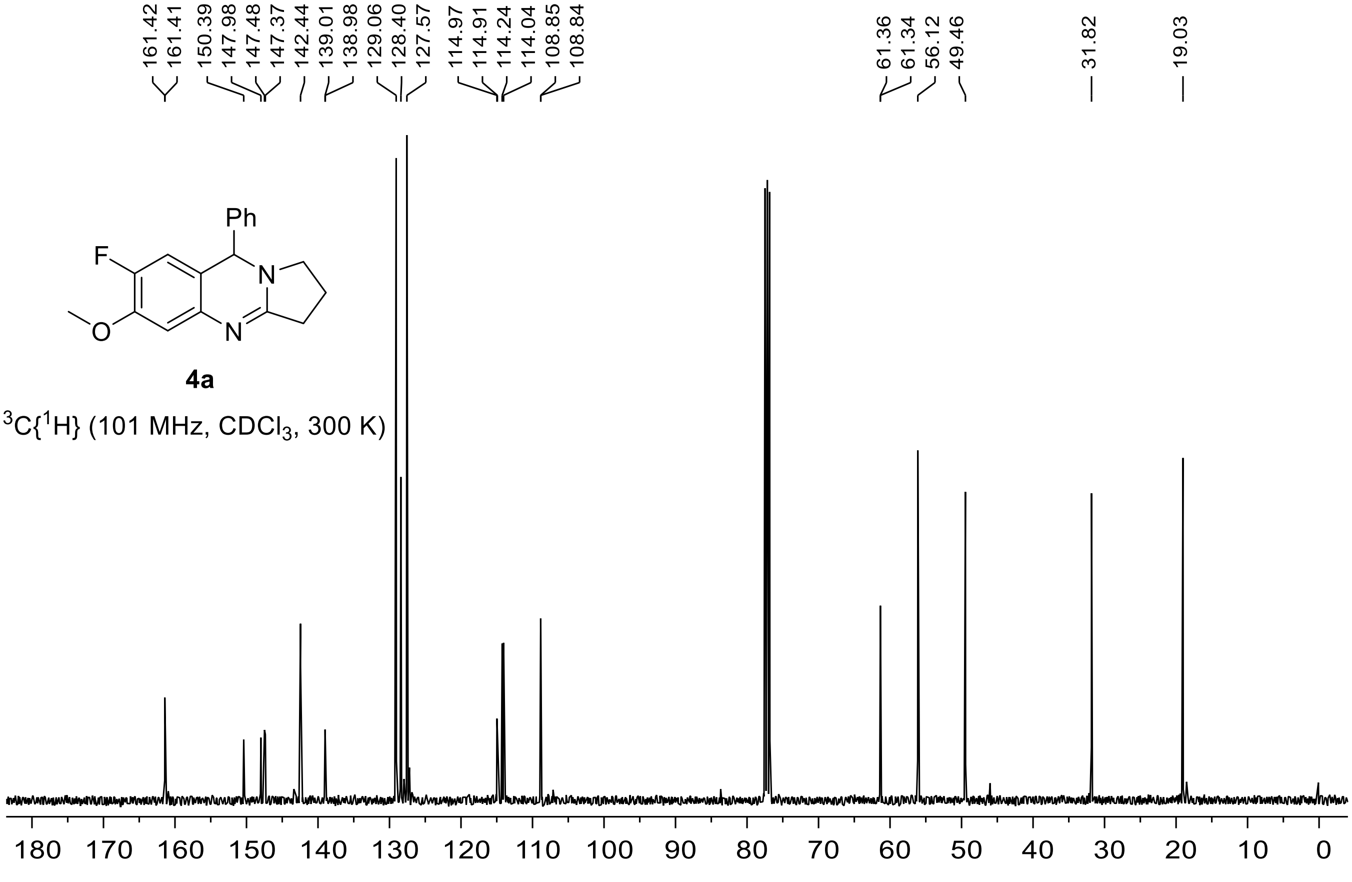
161.42 161.41 150.39 147.98 147.48 147.37 142.44 139.01 138.98 129.06 128.40 127.57 114.97 114.91 114.24 114.04 108.85 108.84
61.36 61.34 56.12 49.46
31.82
19.03
Ph
F
N
O
N
4a
^{13}C{^{1}H} (101 MHz, CDCl$_3$, 300 K)
180 170 160 150 140 130 120 110 100 90 80 70 60 50 40 30 20 10 0

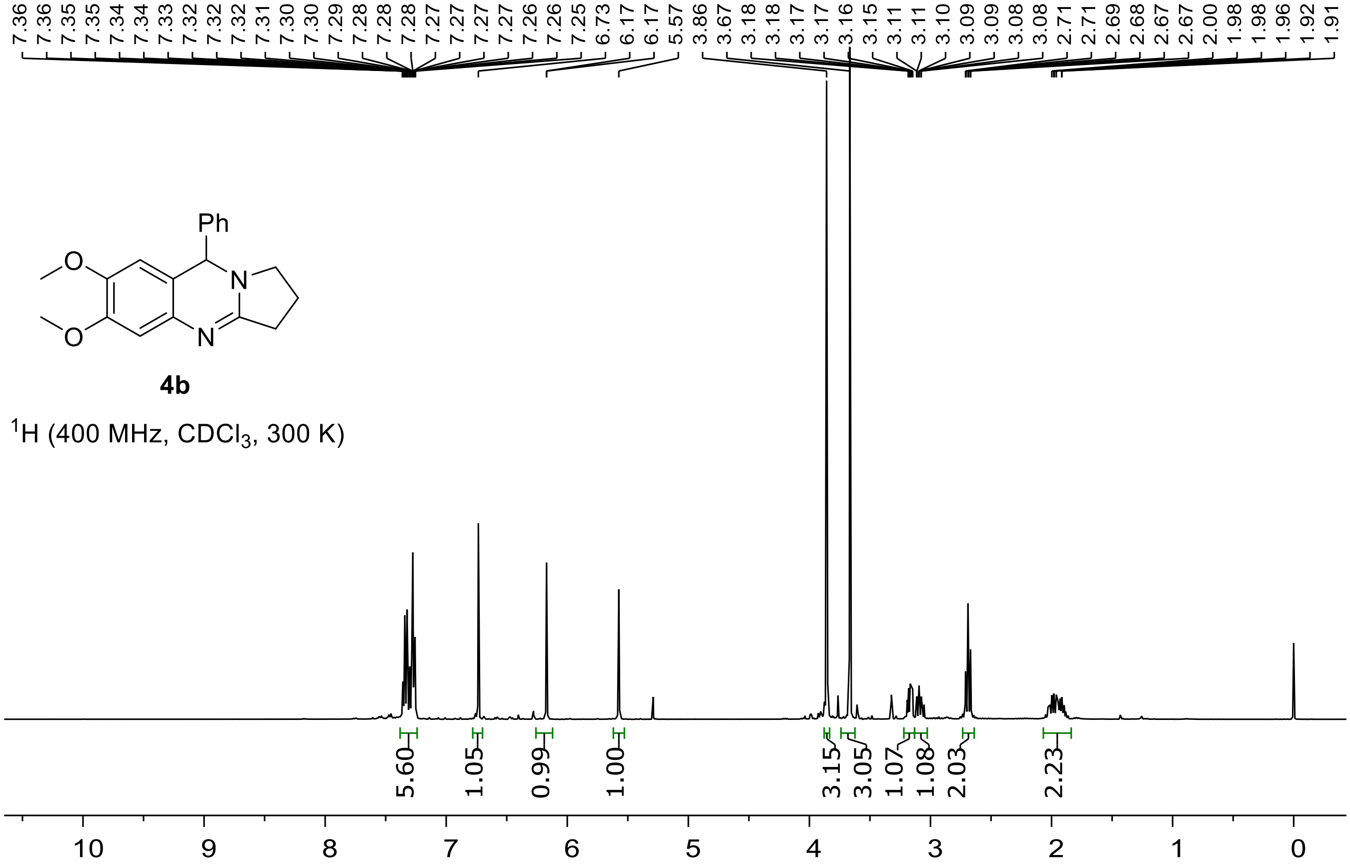
7.36 7.36 7.35 7.34 7.34 7.33 7.32 7.32 7.32 7.31 7.30 7.30 7.29 7.28 7.28 7.28 7.27 7.27 7.27 7.27 7.26 7.25 6.73 6.17 6.17 5.57 3.86 3.67 3.18 3.18 3.17 3.17 3.16 3.15 3.11 3.11 3.10 3.09 3.09 3.08 3.08 2.71 2.71 2.69 2.68 2.67 2.67 2.00 1.98 1.98 1.96 1.92 1.91
Ph
O
O
N
N
4b
^{1}H (400 MHz, $CDCl_3$, 300 K)
5.60 1.05 0.99 1.00 3.15 3.05 1.07 1.08 2.03 2.23
10 9 8 7 6 5 4 3 2 1 0

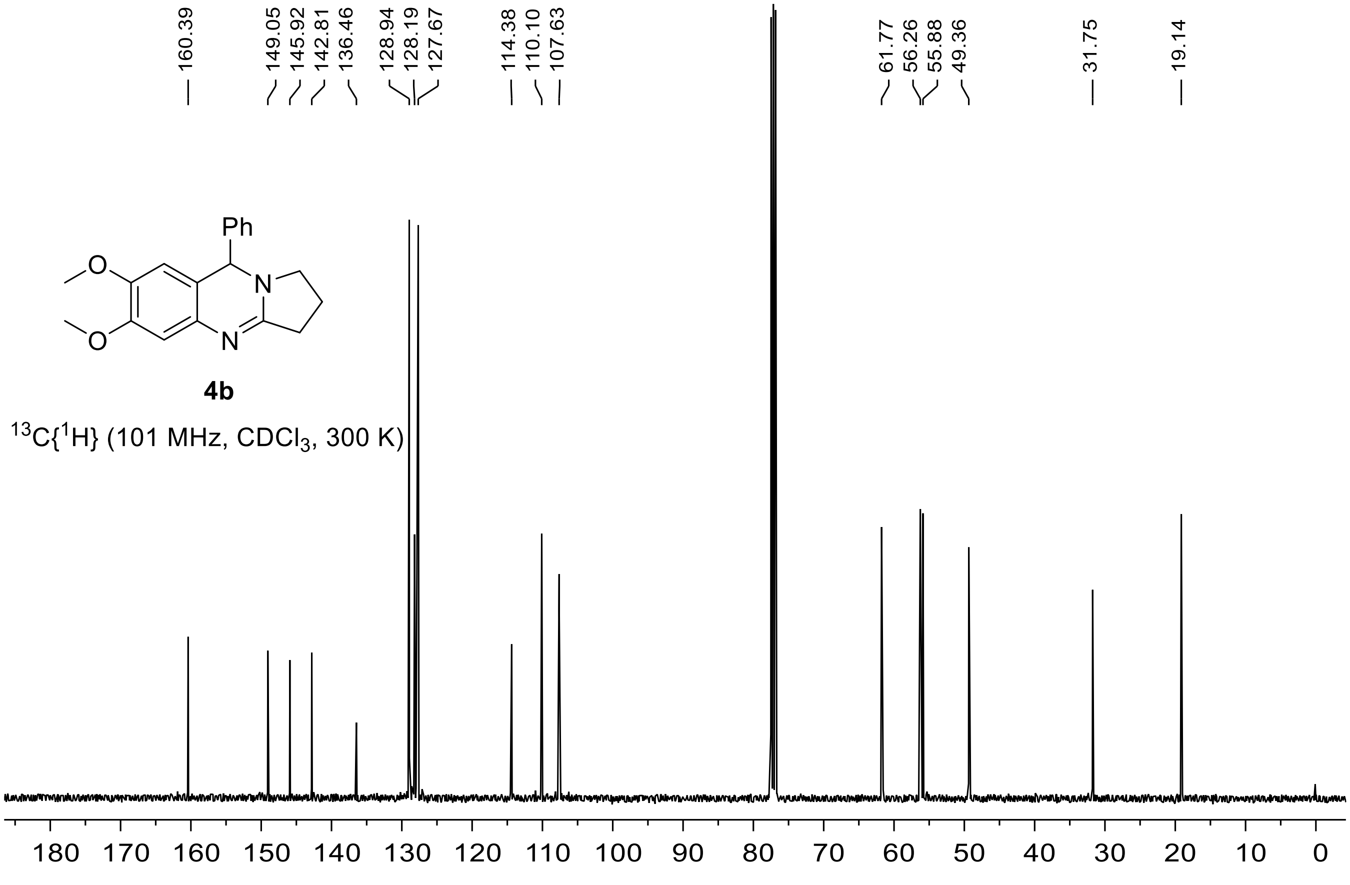
160.39 149.05 145.92 142.81 136.46 128.94 128.19 127.67 114.38 110.10 107.63 61.77 56.26 55.88 49.36 31.75 19.14
Ph
O
O
N
N
4b
$^{13}C\{^{1}H\}$ (101 MHz, $CDCl_3$, 300 K)
180 170 160 150 140 130 120 110 100 90 80 70 60 50 40 30 20 10 0

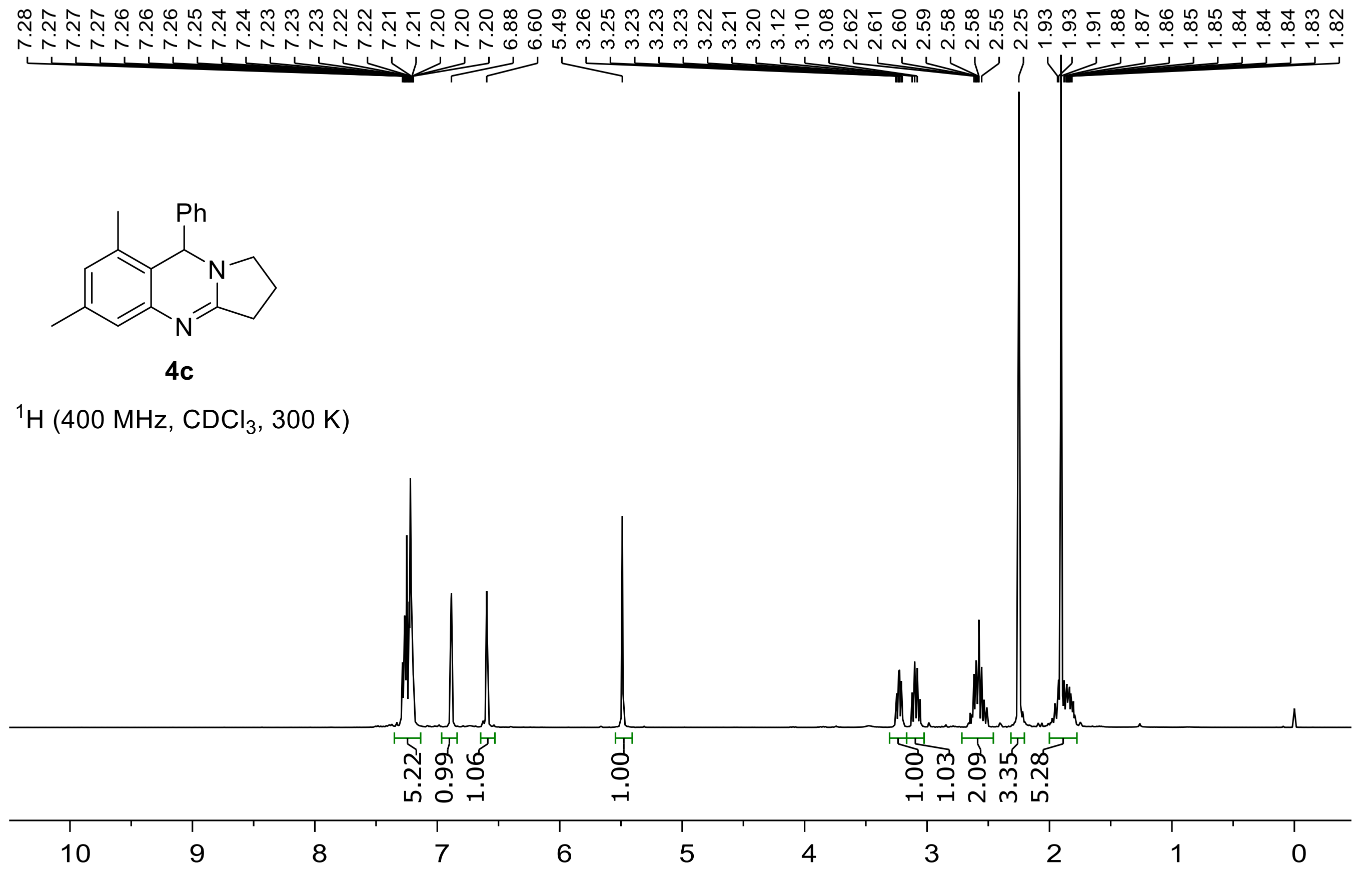
7.28 7.27 7.27 7.27 7.26 7.26 7.26 7.25 7.24 7.24 7.23 7.23 7.23 7.22 7.22 7.21 7.21 7.20 7.20 7.20 6.88 6.60 5.49 3.26 3.25 3.23 3.23 3.23 3.22 3.21 3.20 3.12 3.10 3.08 2.62 2.61 2.60 2.59 2.58 2.58 2.55 2.25 1.93 1.93 1.91 1.88 1.87 1.86 1.85 1.85 1.84 1.84 1.84 1.83 1.82
Ph
N
N
4c
1H (400 MHz, CDCl3, 300 K)
5.22 0.99 1.06 1.00 1.00 1.03 2.09 3.35 5.28
10 9 8 7 6 5 4 3 2 1 0

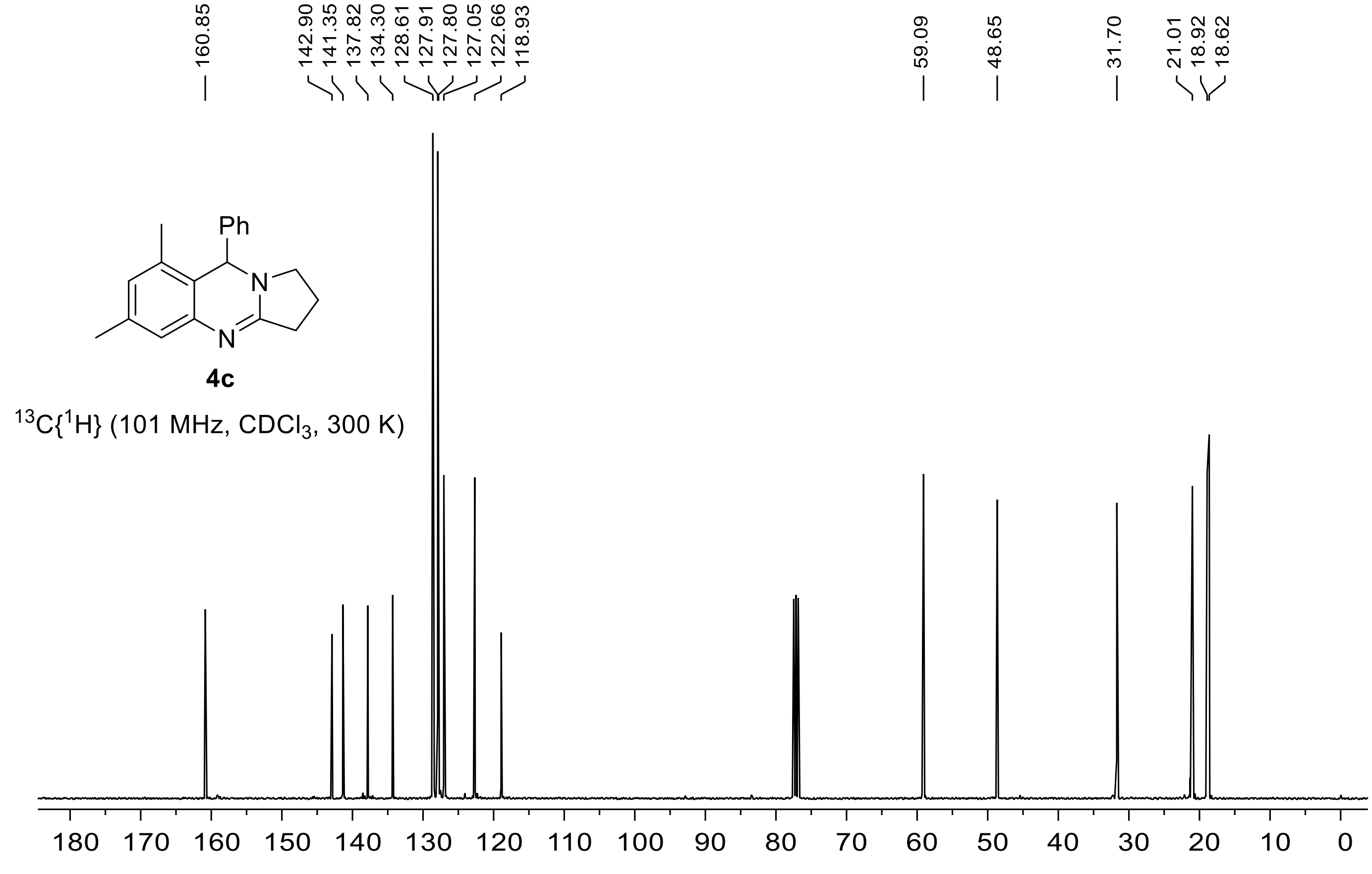
160.85
142.90 141.35 137.82 134.30 128.61 127.91 127.80 127.05 122.66 118.93
59.09
48.65
31.70
21.01 18.92 18.62
Ph
N
N
4c
13C{1H} (101 MHz, CDCl3, 300 K)
180 170 160 150 140 130 120 110 100 90 80 70 60 50 40 30 20 10 0

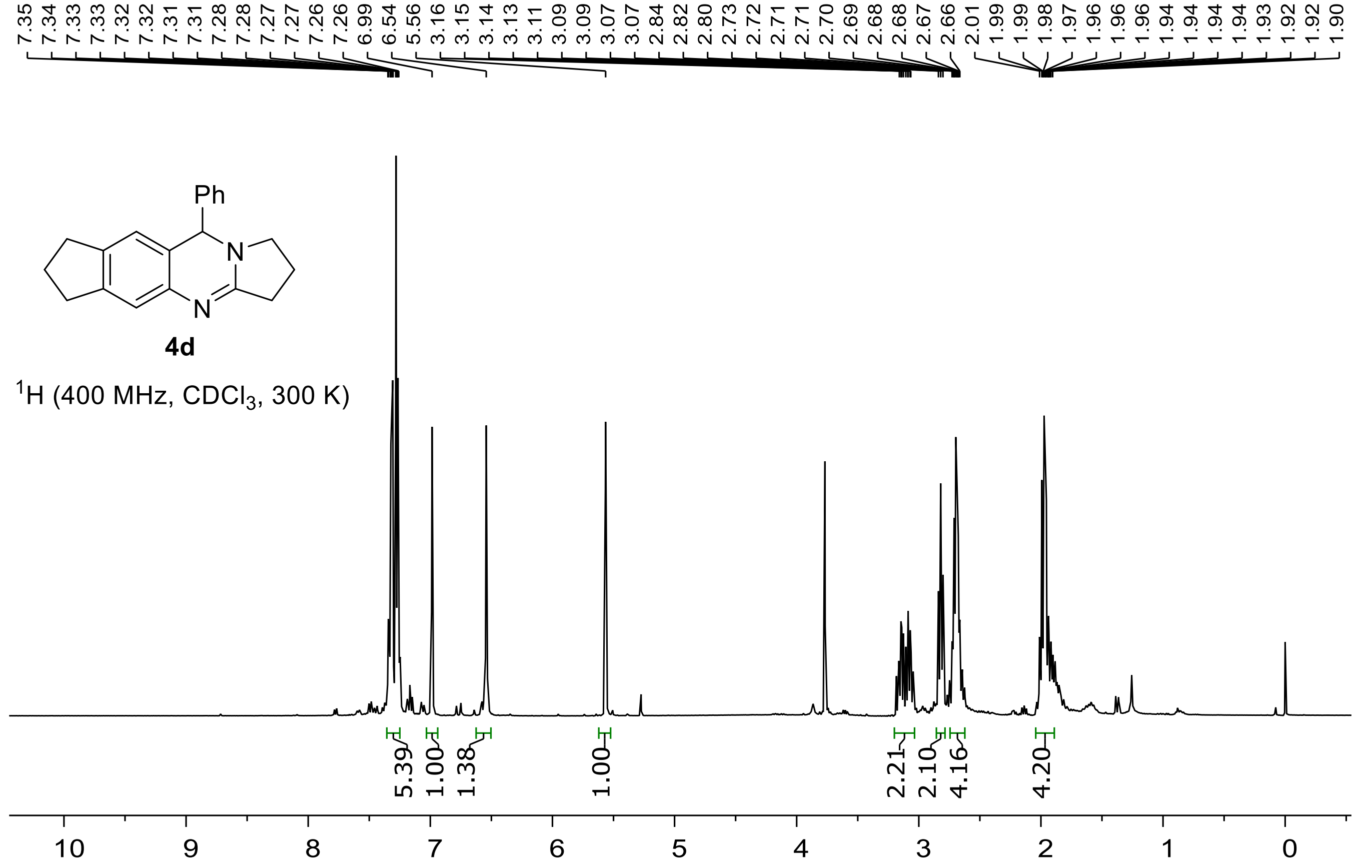
7.35 7.34 7.33 7.33 7.32 7.32 7.31 7.31 7.28 7.28 7.27 7.27 7.26 7.26 6.99 6.54 5.56 3.16 3.15 3.14 3.13 3.11 3.09 3.09 3.07 3.07 2.84 2.82 2.80 2.73 2.72 2.71 2.71 2.70 2.69 2.68 2.68 2.67 2.66 2.01 1.99 1.99 1.98 1.97 1.96 1.96 1.96 1.94 1.94 1.94 1.94 1.93 1.92 1.92 1.90
Ph
N
N
4d
^{1}H (400 MHz, $CDCl_3$, 300 K)
5.39 1.00 1.38 1.00 2.21 2.10 4.16 4.20
10 9 8 7 6 5 4 3 2 1 0

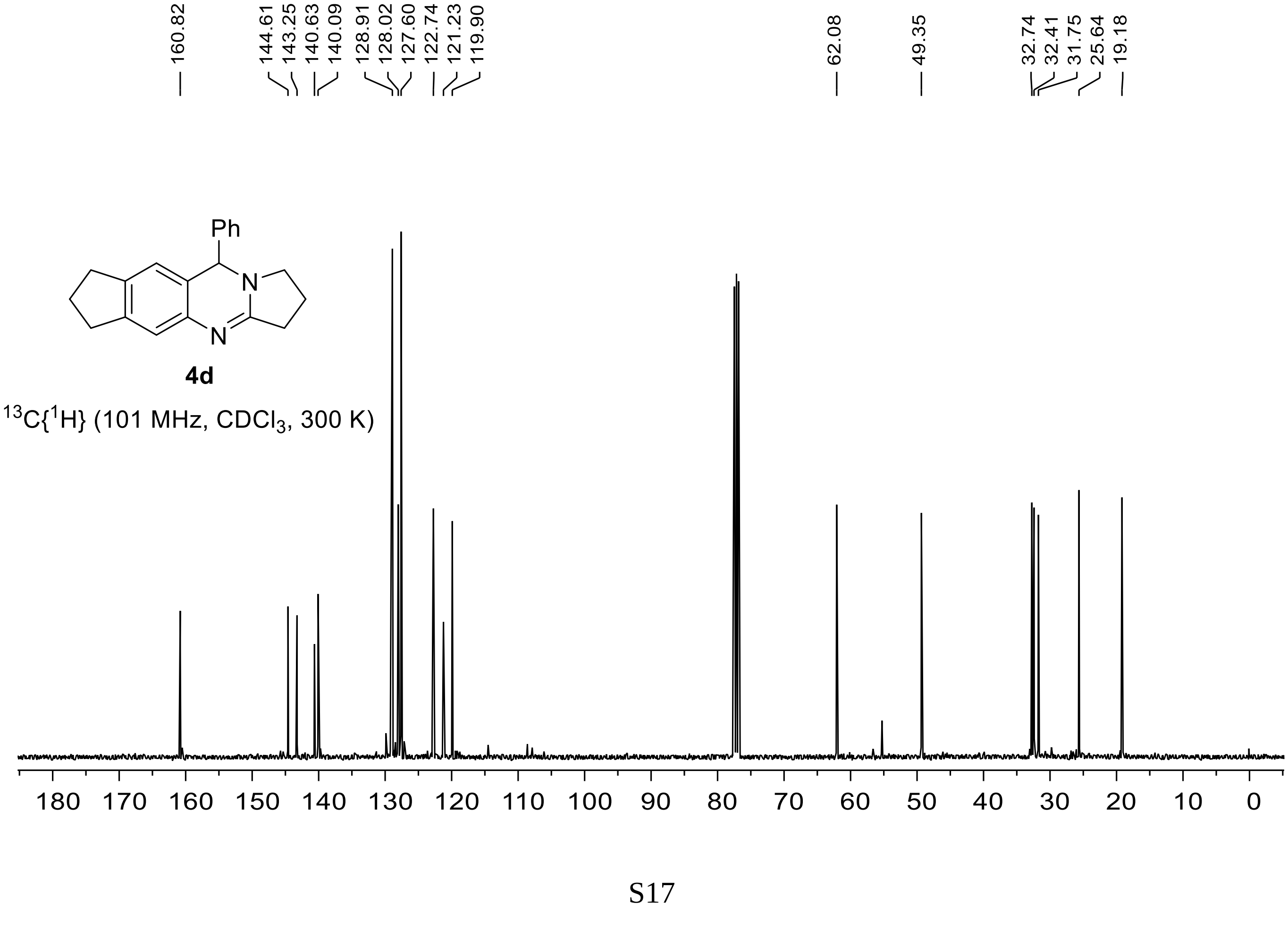
160.82
144.61 143.25 140.63 140.09 128.91 128.02 127.60 122.74 121.23 119.90
62.08
49.35
32.74 32.41 31.75 25.64 19.18
Ph
N
N
4d
$^{13}C\{^{1}H\}$ (101 MHz, $CDCl_3$, 300 K)
180 170 160 150 140 130 120 110 100 90 80 70 60 50 40 30 20 10 0

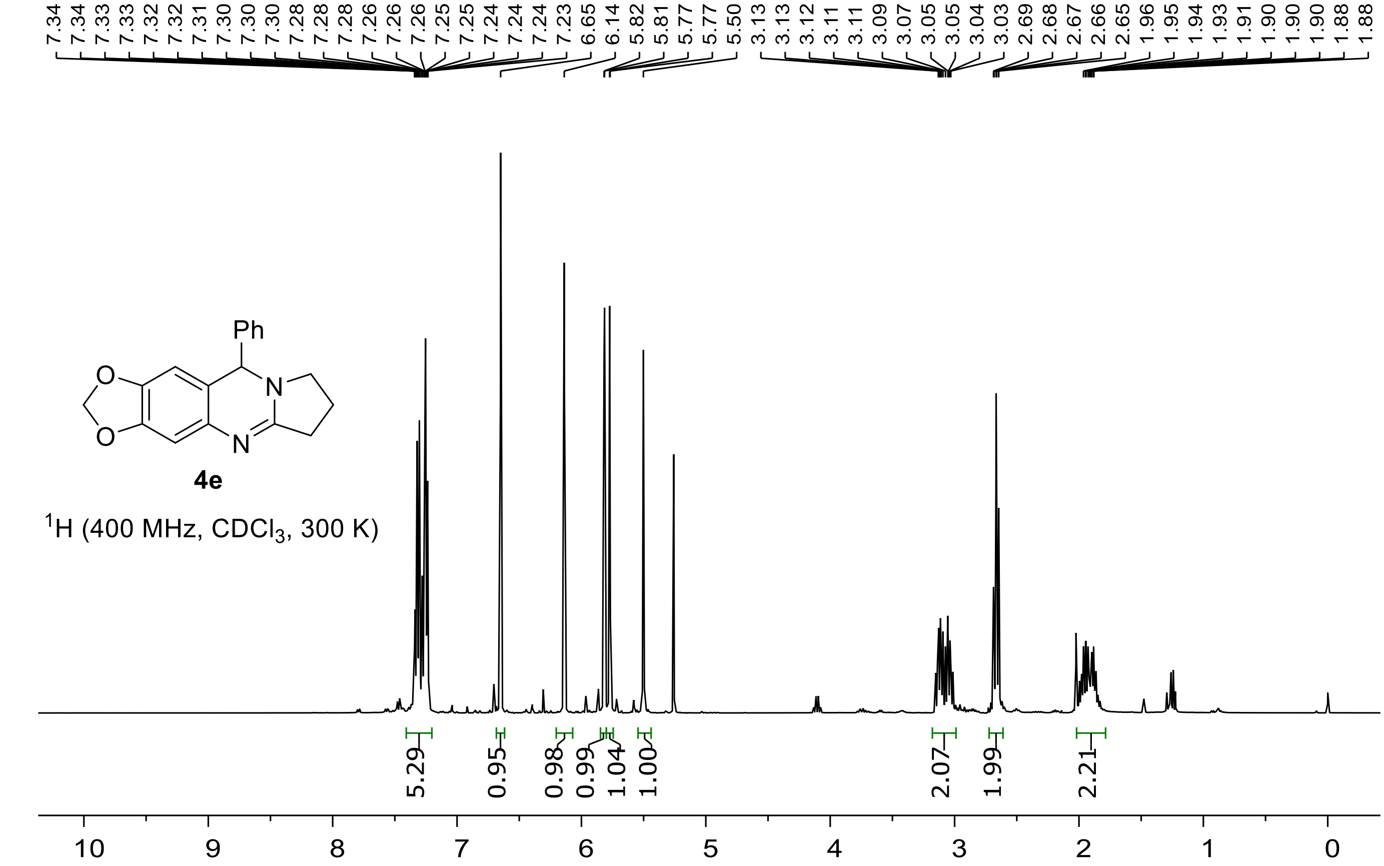
7.34
7.34
7.33
7.33
7.32
7.32
7.31
7.30
7.30
7.30
7.28
7.28
7.26
7.26
7.26
7.25
7.25
7.24
7.24
7.24
7.23
6.65
6.14
5.82
5.81
5.77
5.77
5.50
3.13
3.13
3.12
3.11
3.11
3.09
3.07
3.05
3.05
3.04
3.03
2.69
2.68
2.67
2.66
2.65
1.96
1.95
1.94
1.93
1.91
1.90
1.90
1.90
1.88
1.88
Ph
O
O
N
N
4e
^{1}H (400 MHz, CDCl$_3$, 300 K)
5.29
0.95
0.98
0.99
1.04
1.00
2.07
1.99
2.21
10
9
8
7
6
5
4
3
2
1
0

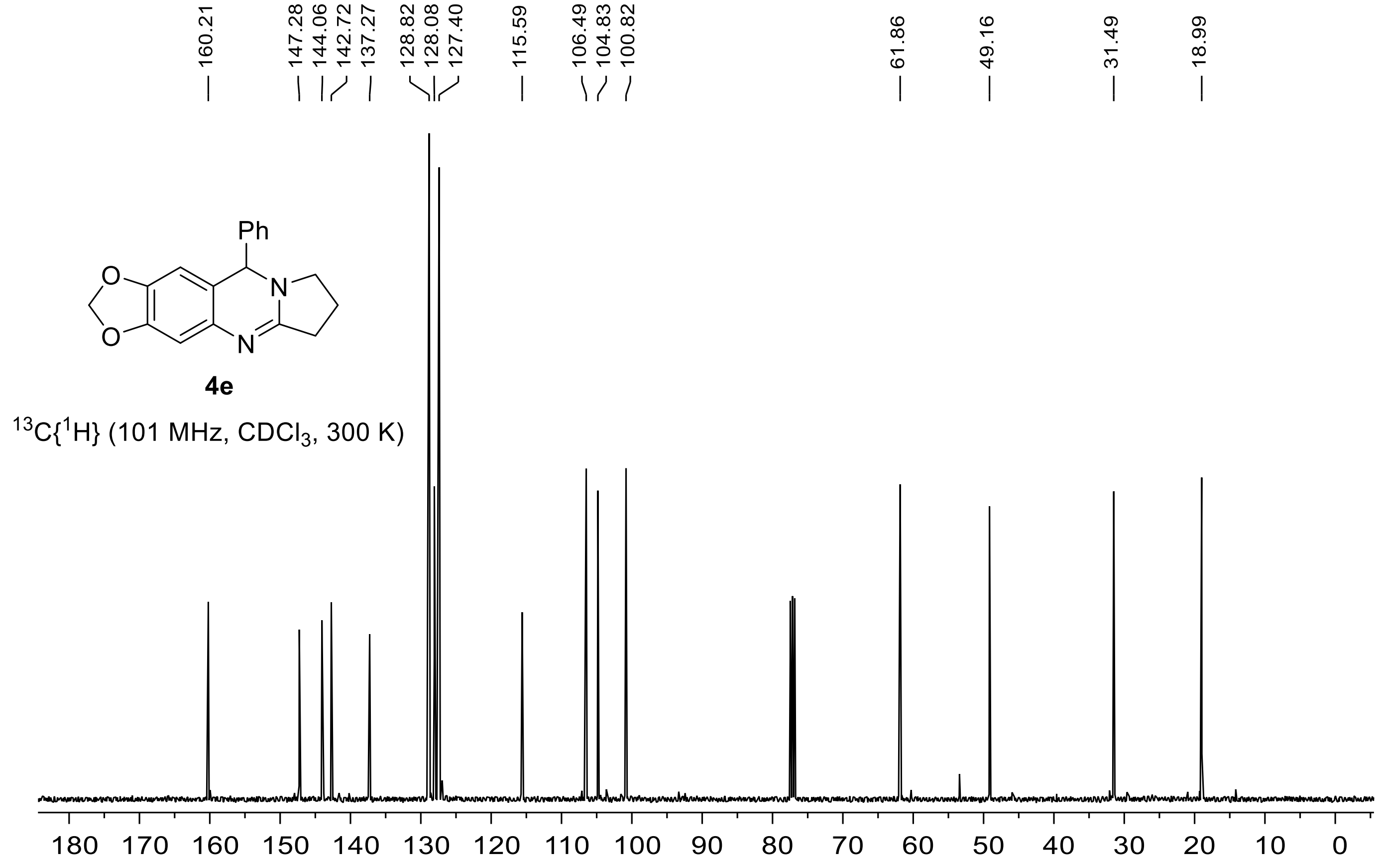
160.21
147.28
144.06
142.72
137.27
128.82
128.08
127.40
115.59
106.49
104.83
100.82
61.86
49.16
31.49
18.99
Ph
O
O
N
N
4e
^{13}C{^{1}H} (101 MHz, CDCl$_3$, 300 K)
180
170
160
150
140
130
120
110
100
90
80
70
60
50
40
30
20
10
0

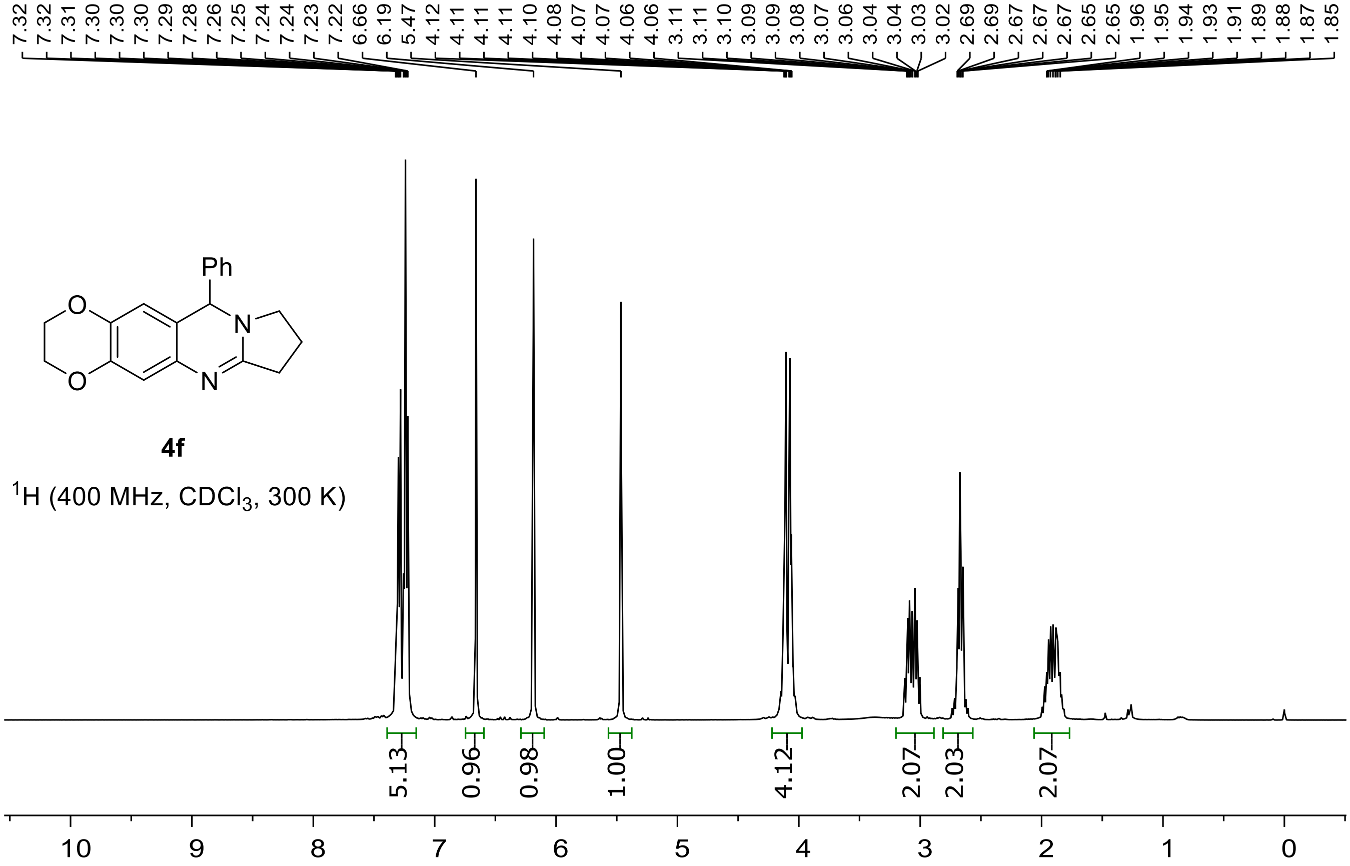

7.32 7.32 7.31 7.30 7.30 7.30 7.29 7.28 7.26 7.25 7.24 7.24 7.23 7.22 6.66 6.19 5.47 4.12 4.11 4.11 4.11 4.10 4.08 4.07 4.07 4.06 4.06 3.11 3.11 3.10 3.09 3.09 3.08 3.07 3.06 3.04 3.04 3.03 3.02 2.69 2.69 2.67 2.67 2.67 2.65 2.65 1.96 1.95 1.94 1.93 1.91 1.89 1.88 1.87 1.85
Ph
O
O
N
N
4f
^{1}H (400 MHz, CDCl$_3$, 300 K)
5.13
0.96
0.98
1.00
4.12
2.07
2.03
2.07
10 9 8 7 6 5 4 3 2 1 0

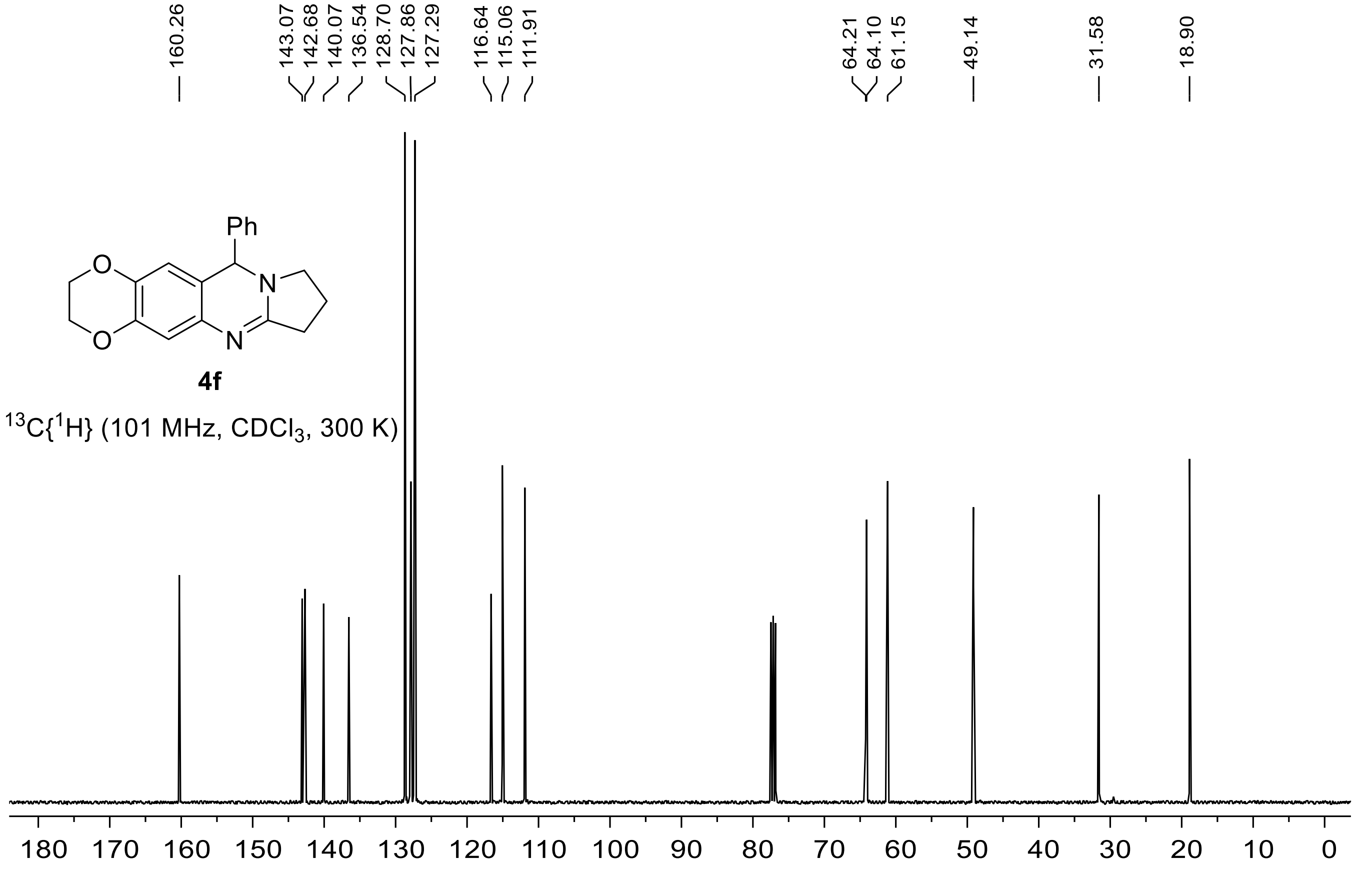

160.26
143.07 142.68 140.07 136.54 128.70 127.86 127.29
116.64 115.06 111.91
64.21 64.10 61.15
49.14
31.58
18.90
Ph
O
O
N
N
4f
^{13}C{^{1}H} (101 MHz, CDCl$_3$, 300 K)
180 170 160 150 140 130 120 110 100 90 80 70 60 50 40 30 20 10 0

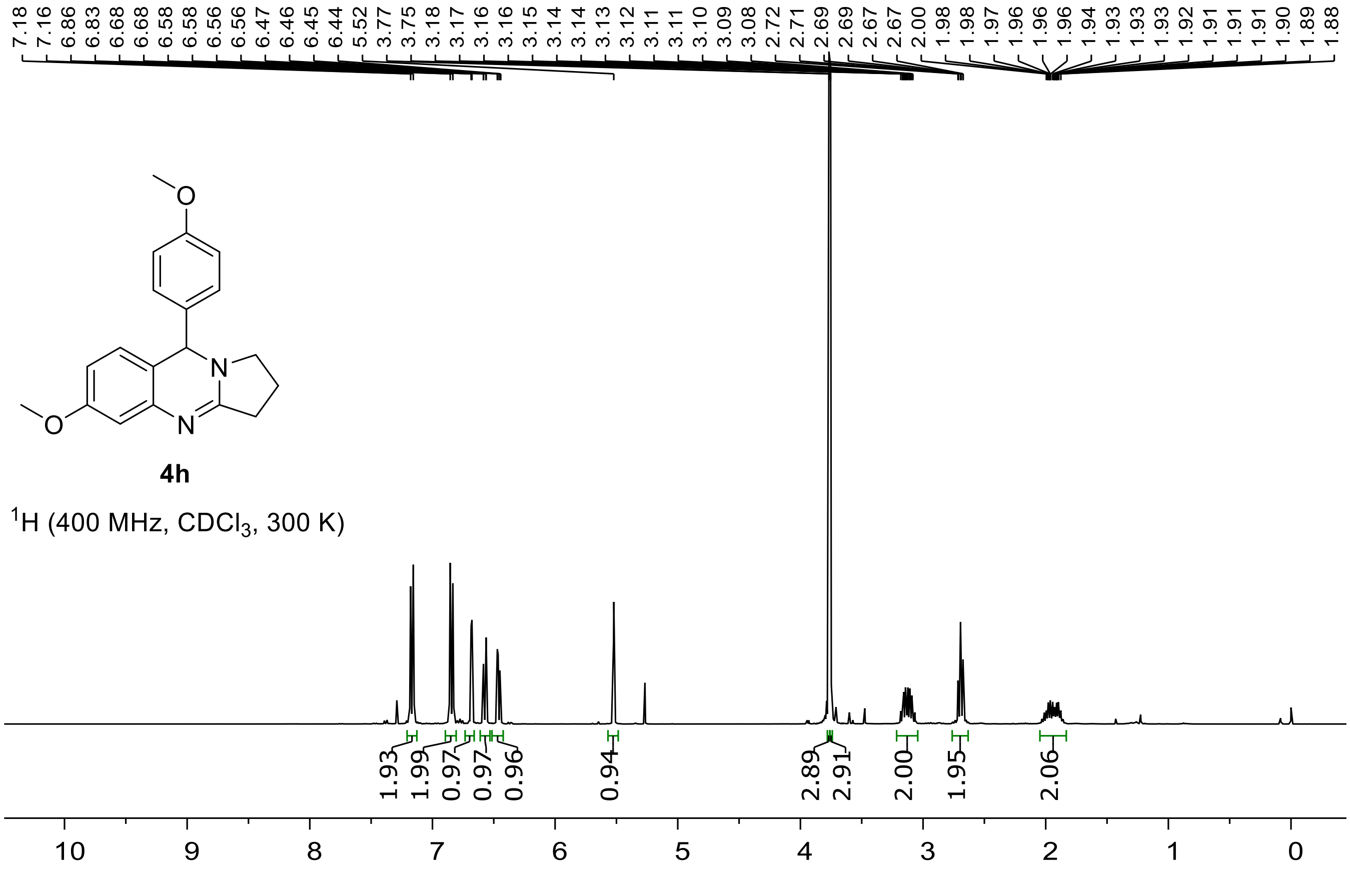
7.18 7.16 6.86 6.83 6.68 6.68 6.58 6.58 6.56 6.56 6.47 6.46 6.45 6.44 5.52 3.77 3.75 3.18 3.17 3.16 3.16 3.15 3.14 3.14 3.13 3.12 3.11 3.11 3.10 3.09 3.08 2.72 2.71 2.69 2.69 2.67 2.67 2.00 1.98 1.98 1.97 1.96 1.96 1.96 1.94 1.93 1.93 1.93 1.92 1.91 1.91 1.91 1.90 1.89 1.88
N
O
4h
^{1}H (400 MHz, CDCl$_3$, 300 K)
1.93 1.99 0.97 0.97 0.96 0.94 2.89 2.91 2.00 1.95 2.06
10 9 8 7 6 5 4 3 2 1 0

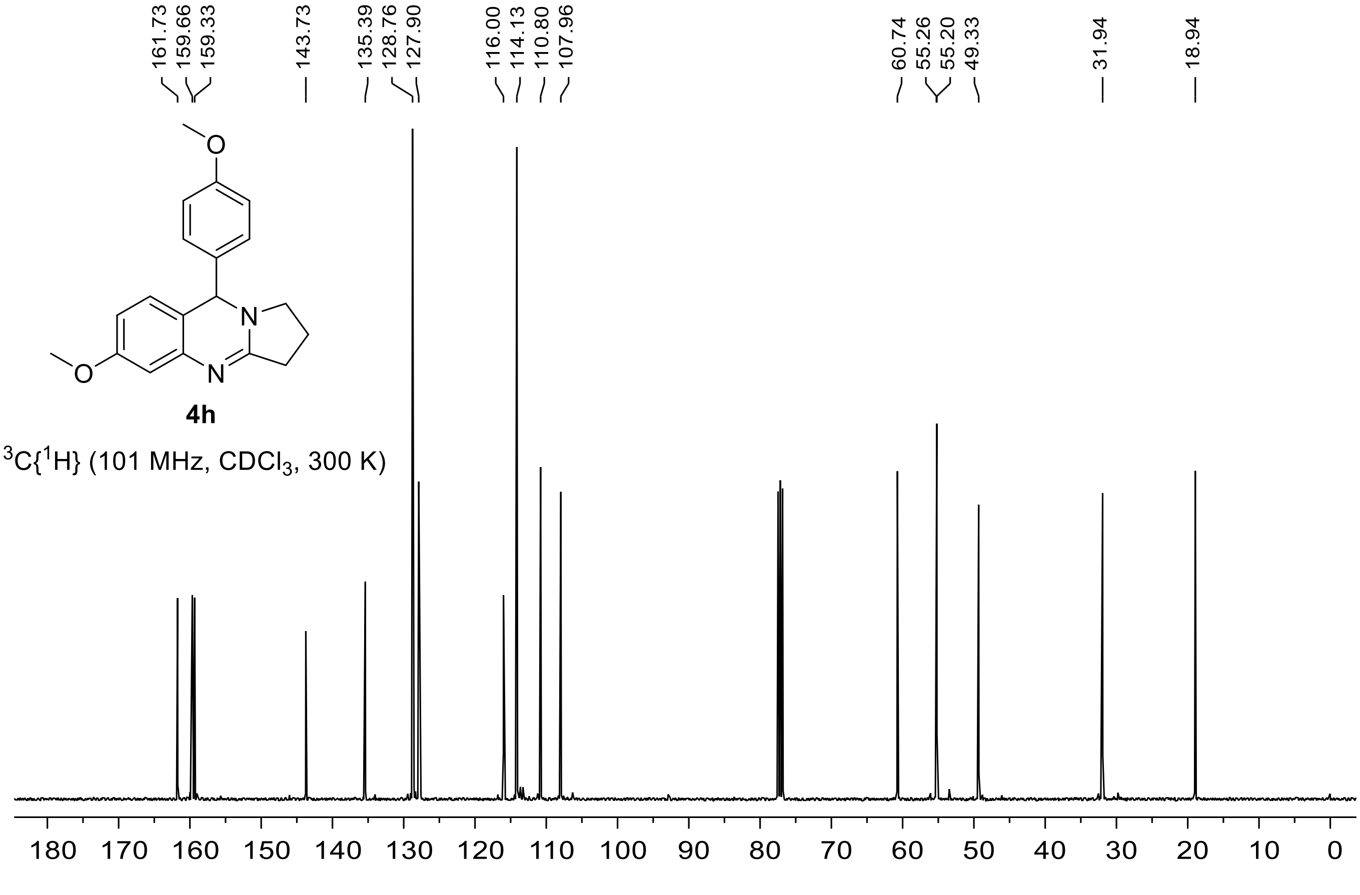
161.73 159.66 159.33 143.73 135.39 128.76 127.90 116.00 114.13 110.80 107.96 60.74 55.26 55.20 49.33 31.94 18.94
N
O
4h
^{13}C{^{1}H} (101 MHz, CDCl$_3$, 300 K)
180 170 160 150 140 130 120 110 100 90 80 70 60 50 40 30 20 10 0

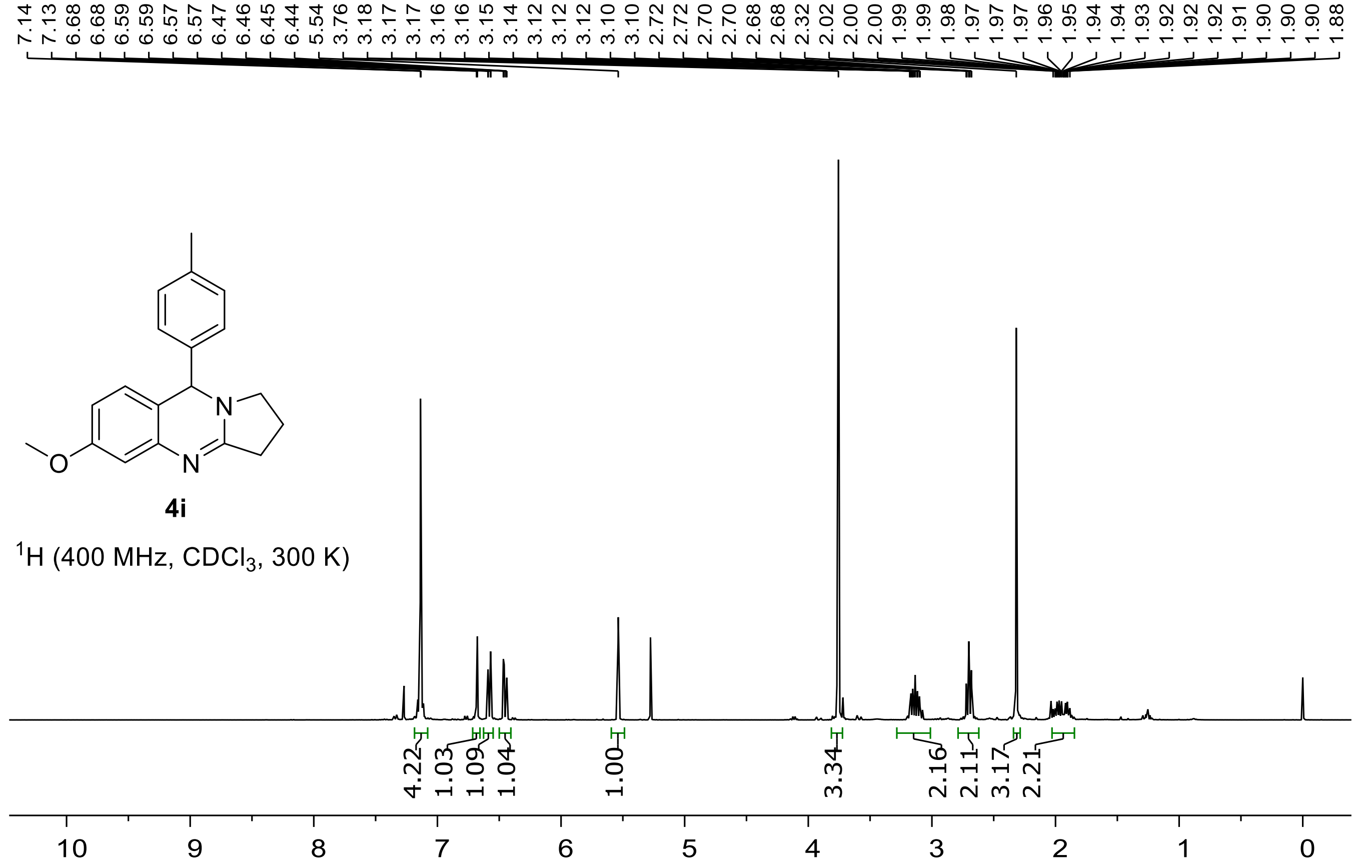

4i

^{1}H (400 MHz, $CDCl_3$, 300 K)

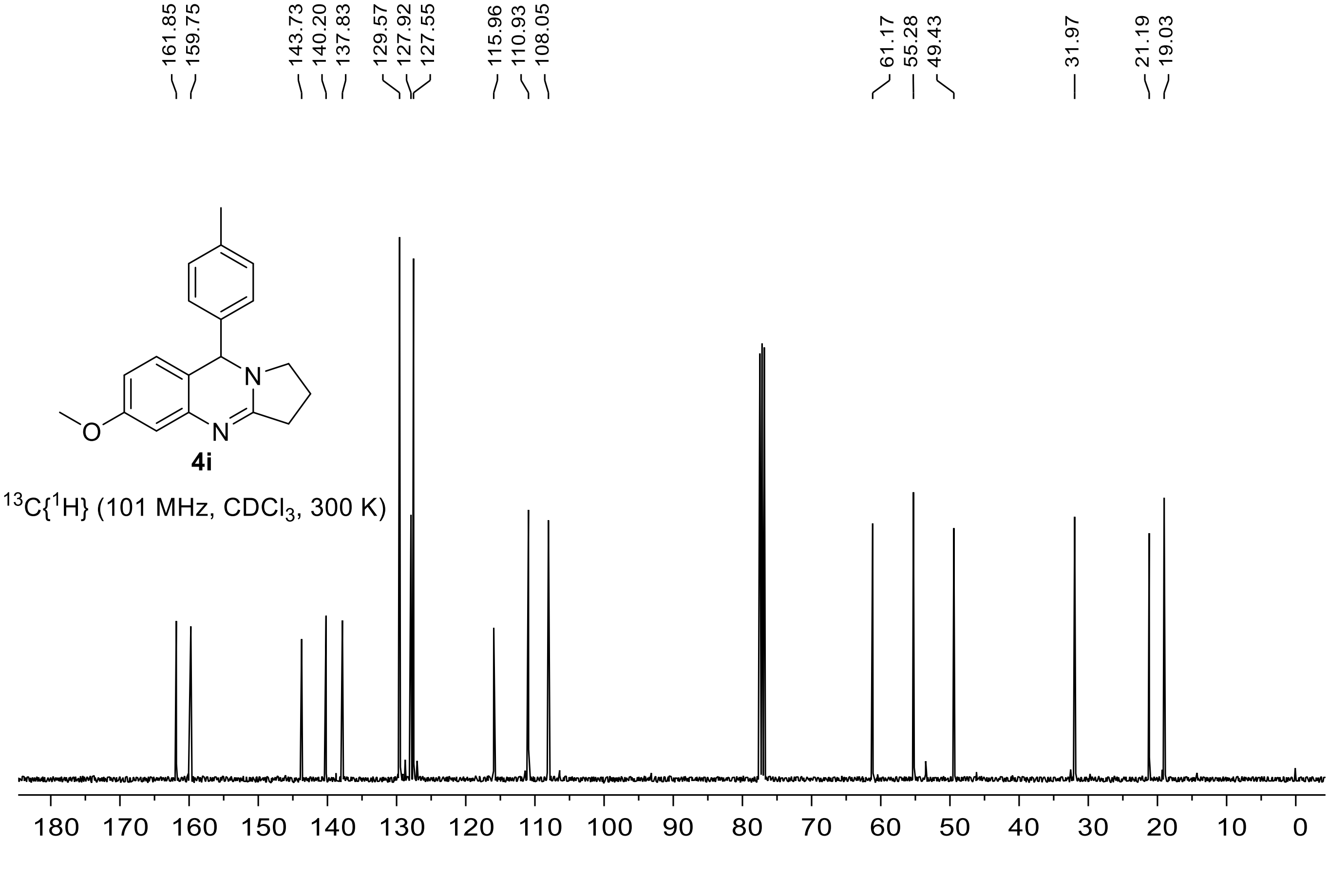

4i

^{13}C{^{1}H} (101 MHz, $CDCl_3$, 300 K)

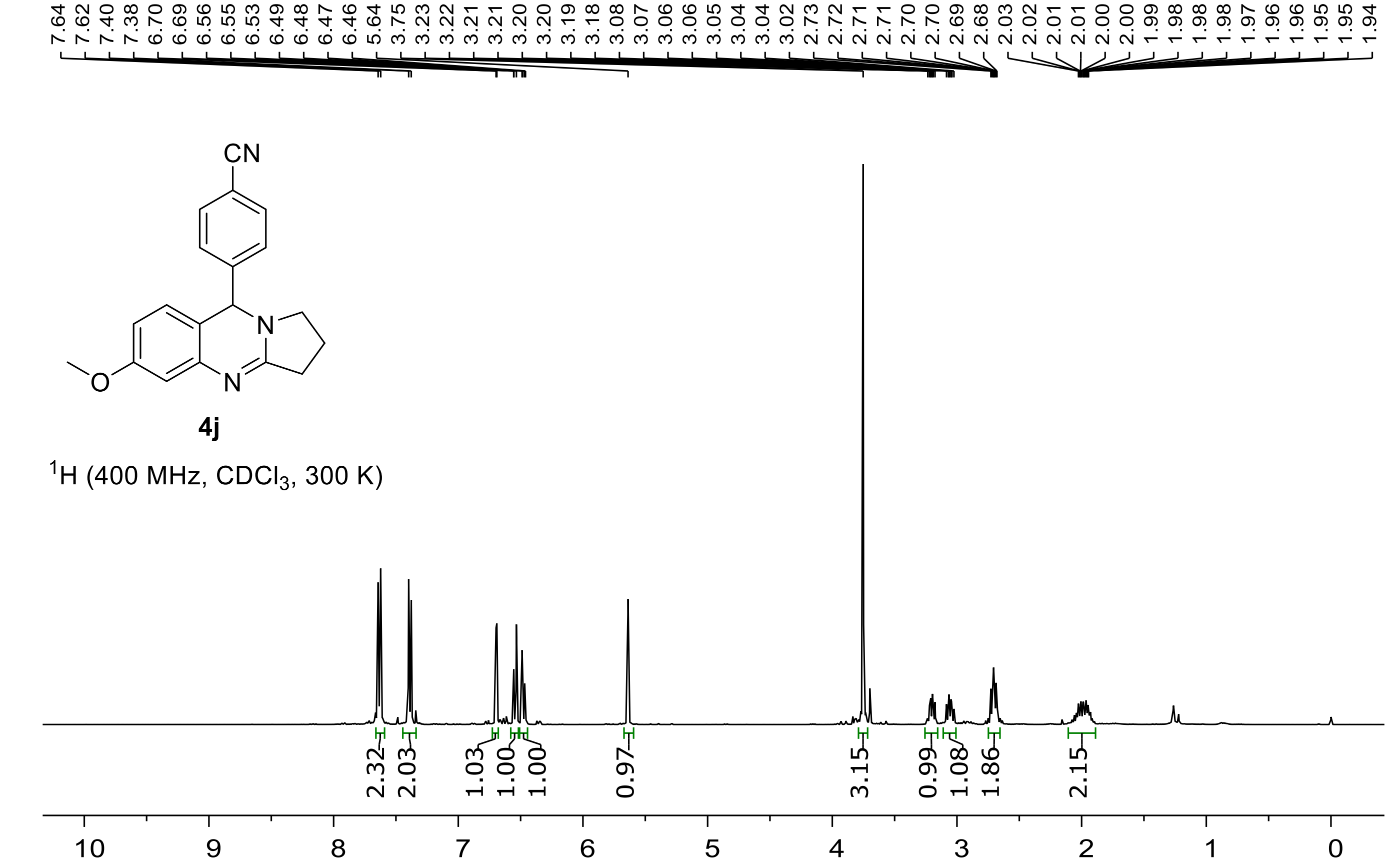
7.64 7.62 7.40 7.38 6.70 6.69 6.56 6.55 6.53 6.49 6.48 6.47 6.46 5.64 3.75 3.23 3.22 3.21 3.21 3.20 3.20 3.19 3.18 3.08 3.07 3.06 3.06 3.05 3.04 3.04 3.02 2.73 2.72 2.71 2.71 2.70 2.70 2.69 2.68 2.03 2.02 2.01 2.01 2.00 2.00 1.99 1.98 1.98 1.98 1.97 1.97 1.96 1.95 1.95 1.94
CN
O
N
N
4j
1H (400 MHz, CDCl3, 300 K)
2.32 2.03 1.03 1.00 1.00 0.97 3.15 0.99 1.08 1.86 2.15
10 9 8 7 6 5 4 3 2 1 0

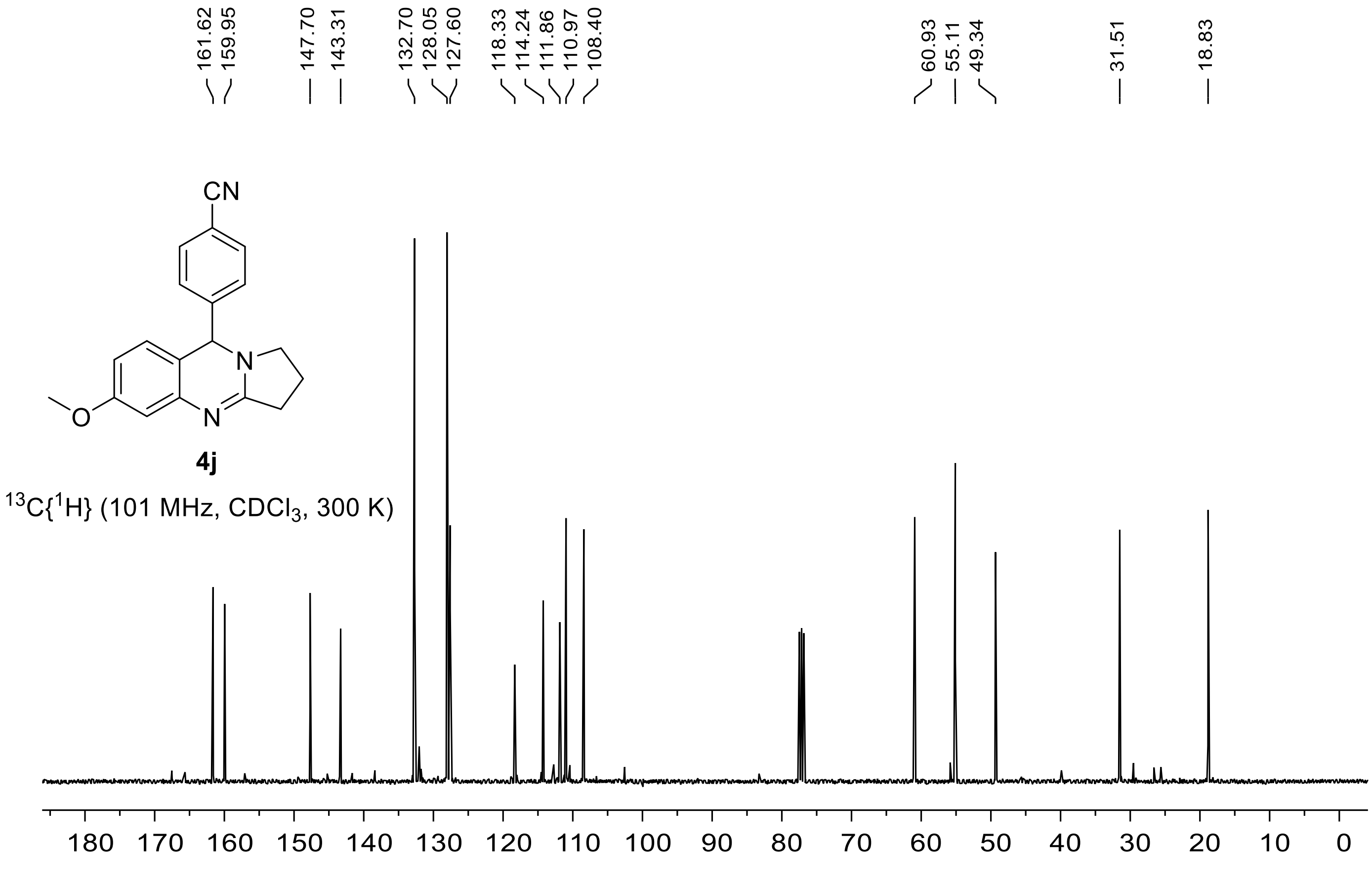
161.62 159.95 147.70 143.31 132.70 128.05 127.60 118.33 114.24 111.86 110.97 108.40 60.93 55.11 49.34 31.51 18.83
CN
O
N
N
4j
13C{1H} (101 MHz, CDCl3, 300 K)
180 170 160 150 140 130 120 110 100 90 80 70 60 50 40 30 20 10 0

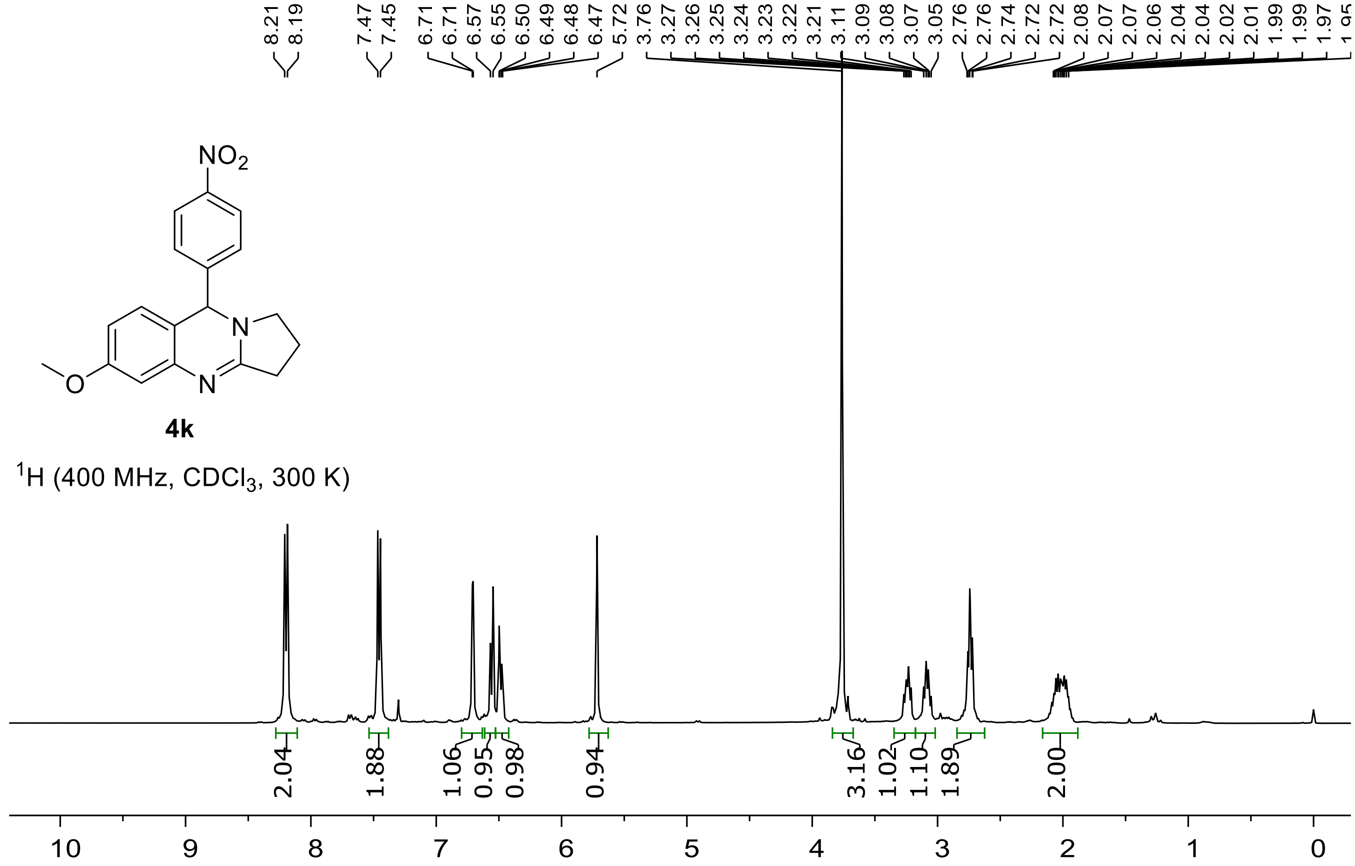

8.21
8.19
7.47
7.45
6.71
6.71
6.57
6.55
6.50
6.49
6.48
6.47
5.72
3.76
3.27
3.26
3.25
3.24
3.23
3.22
3.21
3.11
3.09
3.08
3.07
3.05
2.76
2.76
2.74
2.72
2.72
2.08
2.07
2.07
2.06
2.04
2.04
2.02
2.01
1.99
1.99
1.97
NO2
N
O
N
4k
1H (400 MHz, CDCl3, 300 K)
2.04
1.88
1.06
0.95
0.98
0.94
3.16
1.02
1.10
1.89
2.00
10
9
8
7
6
5
4
3
2
1
0

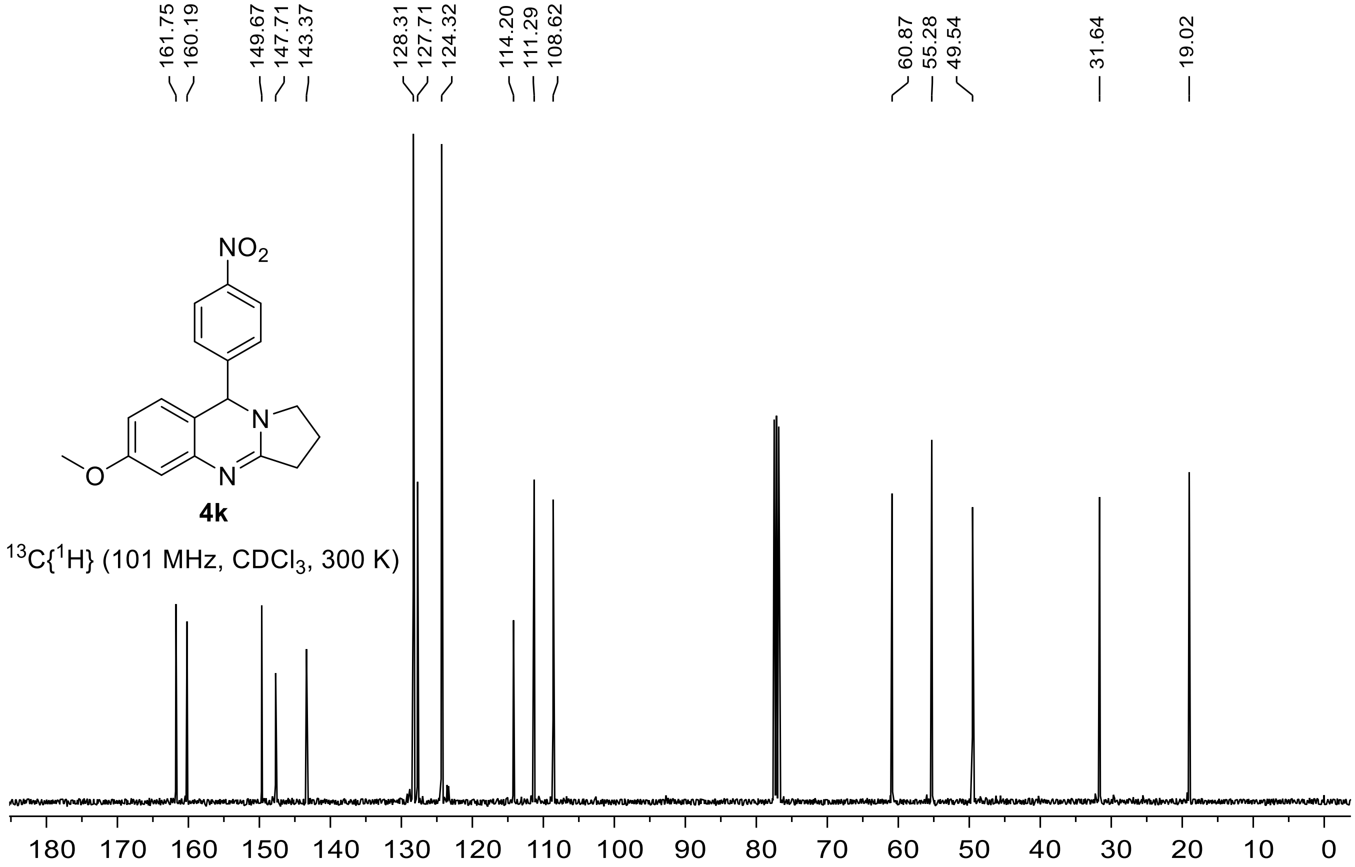

161.75
160.19
149.67
147.71
143.37
128.31
127.71
124.32
114.20
111.29
108.62
60.87
55.28
49.54
31.64
19.02
NO2
N
O
N
4k
13C{1H} (101 MHz, CDCl3, 300 K)
180
170
160
150
140
130
120
110
100
90
80
70
60
50
40
30
20
10
0

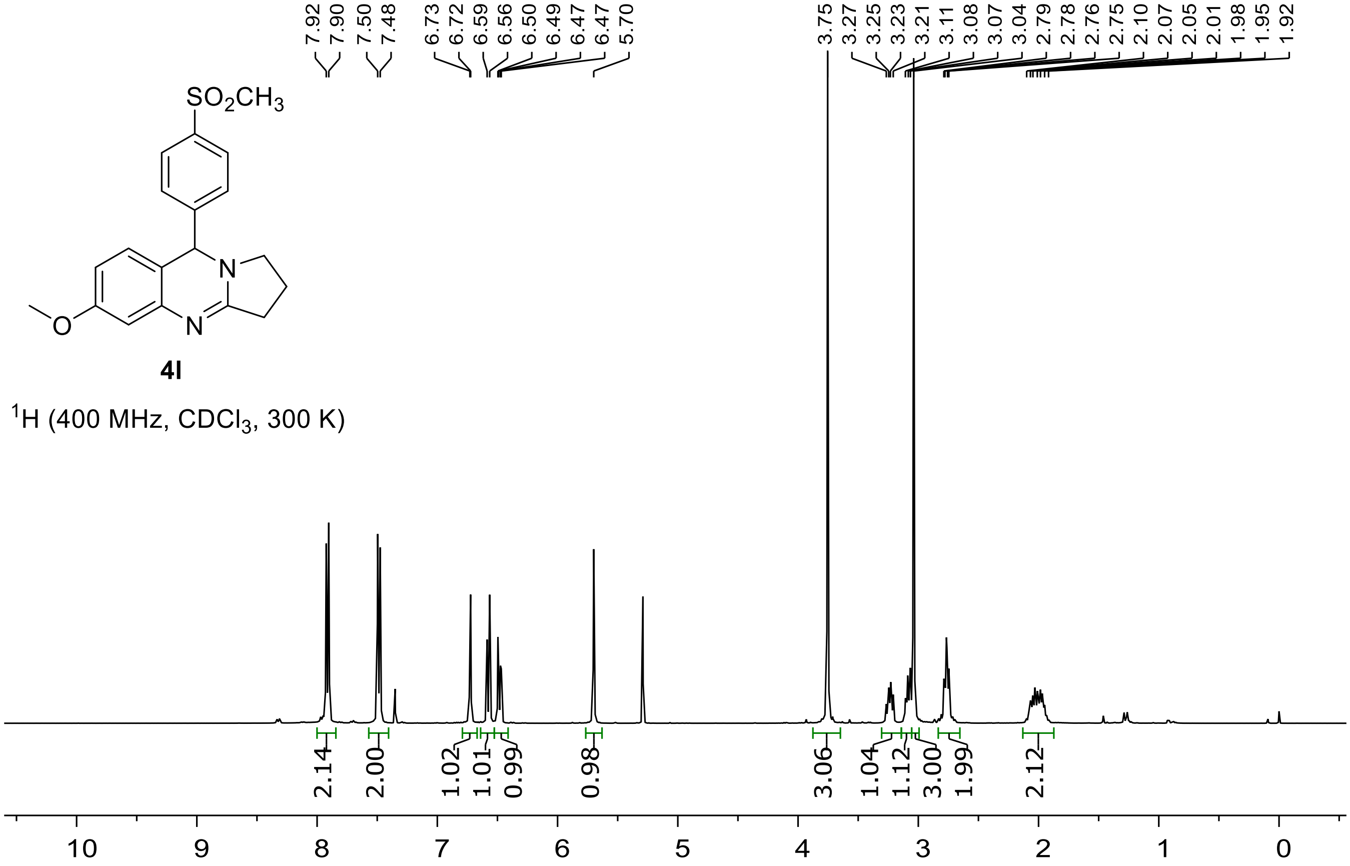
7.92
7.90
7.50
7.48
6.73
6.72
6.59
6.56
6.50
6.49
6.47
6.47
5.70
3.75
3.27
3.25
3.23
3.21
3.11
3.08
3.07
3.04
2.79
2.78
2.76
2.75
2.10
2.07
2.05
2.01
1.98
1.95
1.92
SO_2CH_3
N
N
O
4l
^{1}H (400 MHz, $CDCl_3$, 300 K)
2.14
2.00
1.02
1.01
0.99
0.98
3.06
1.04
1.12
3.00
1.99
2.12
10
9
8
7
6
5
4
3
2
1
0

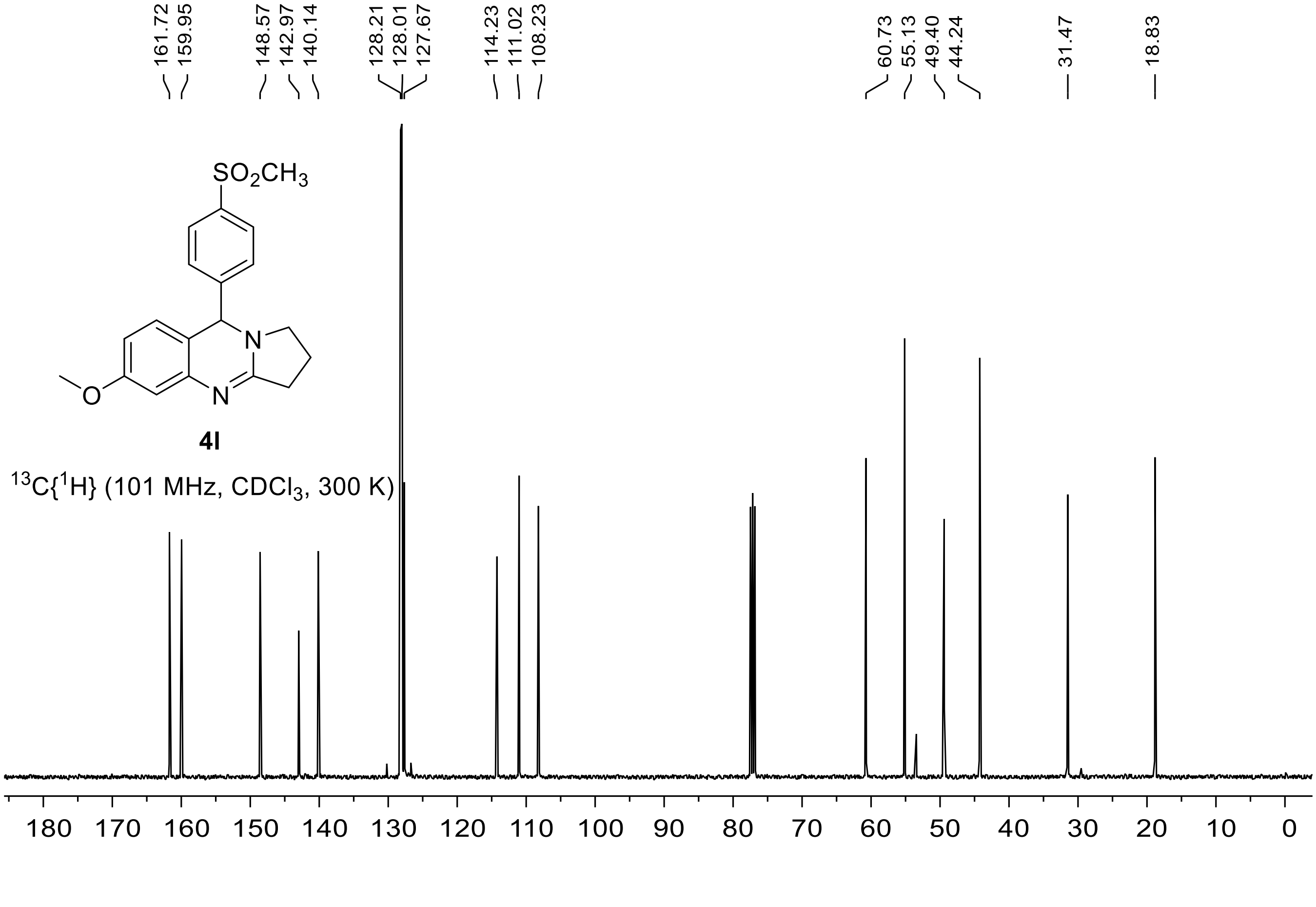
161.72
159.95
148.57
142.97
140.14
128.21
128.01
127.67
114.23
111.02
108.23
60.73
55.13
49.40
44.24
31.47
18.83
SO_2CH_3
N
N
O
4l
^{13}C{^{1}H} (101 MHz, $CDCl_3$, 300 K)
180
170
160
150
140
130
120
110
100
90
80
70
60
50
40
30
20
10
0

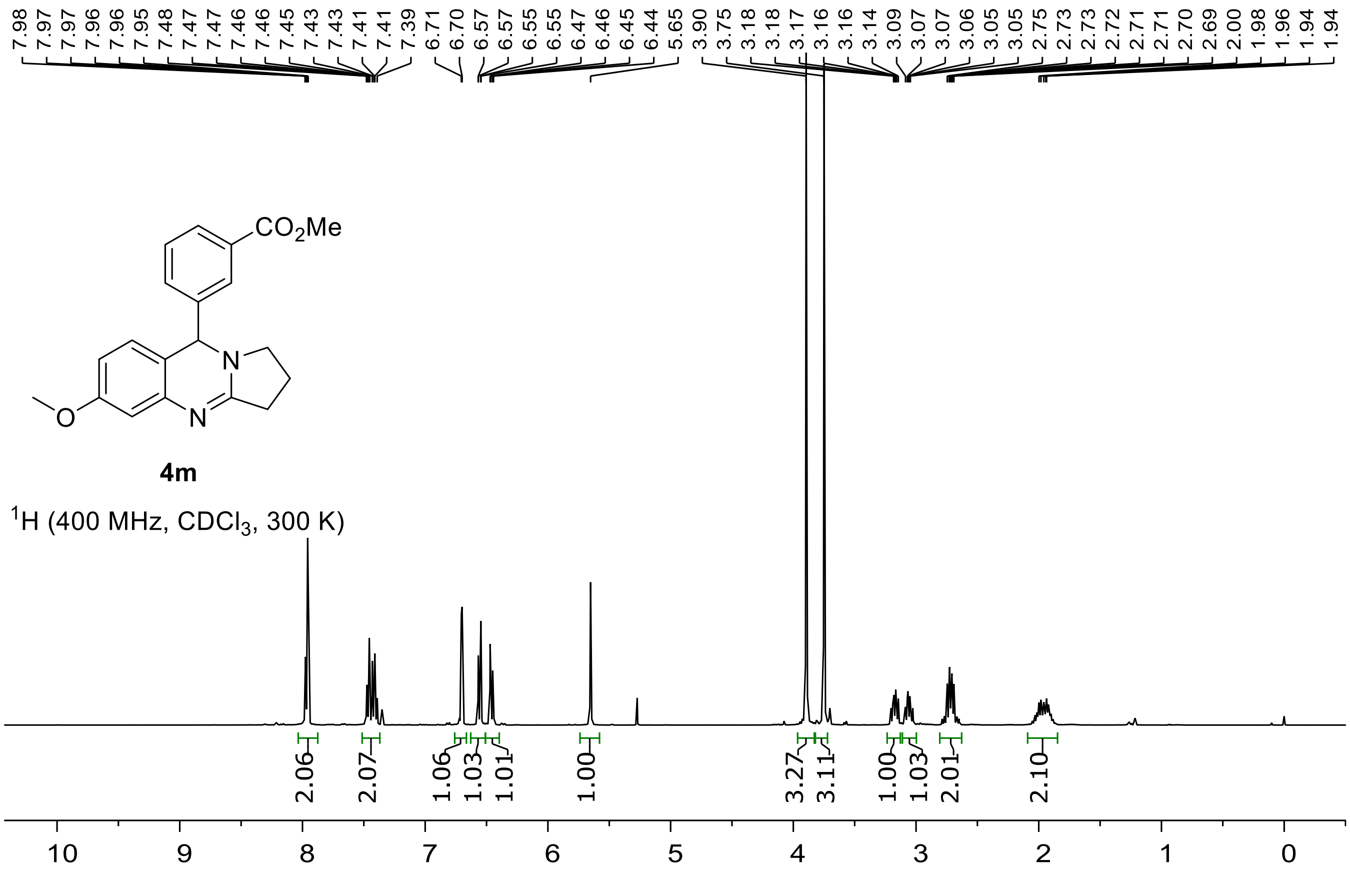
7.98 7.97 7.97 7.96 7.96 7.95 7.48 7.47 7.47 7.46 7.46 7.45 7.43 7.43 7.41 7.41 7.39 6.71 6.70 6.57 6.57 6.55 6.55 6.47 6.46 6.45 6.44 5.65 3.90 3.75 3.18 3.18 3.17 3.16 3.16 3.14 3.09 3.07 3.07 3.06 3.05 3.05 2.75 2.73 2.73 2.72 2.71 2.71 2.70 2.69 2.00 1.98 1.96 1.94 1.94
CO2Me
N
N
O
4m
1H (400 MHz, CDCl3, 300 K)
2.06 2.07 1.06 1.03 1.01 1.00 3.27 3.11 1.00 1.03 2.01 2.10
10 9 8 7 6 5 4 3 2 1 0

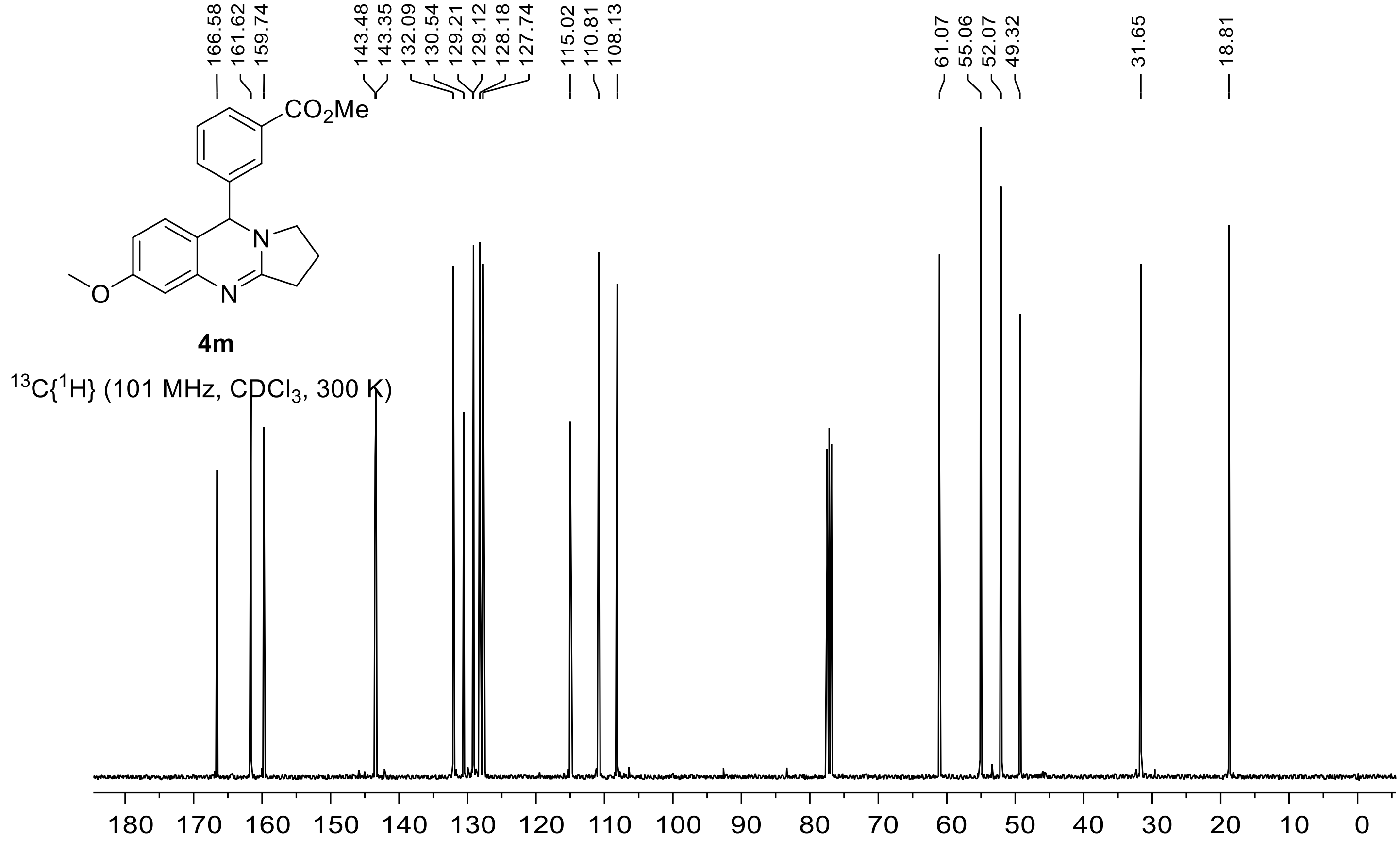
166.58 161.62 159.74 143.48 143.35 132.09 130.54 129.21 129.12 128.18 127.74 115.02 110.81 108.13 61.07 55.06 52.07 49.32 31.65 18.81
CO2Me
N
N
O
4m
13C{1H} (101 MHz, CDCl3, 300 K)
180 170 160 150 140 130 120 110 100 90 80 70 60 50 40 30 20 10 0

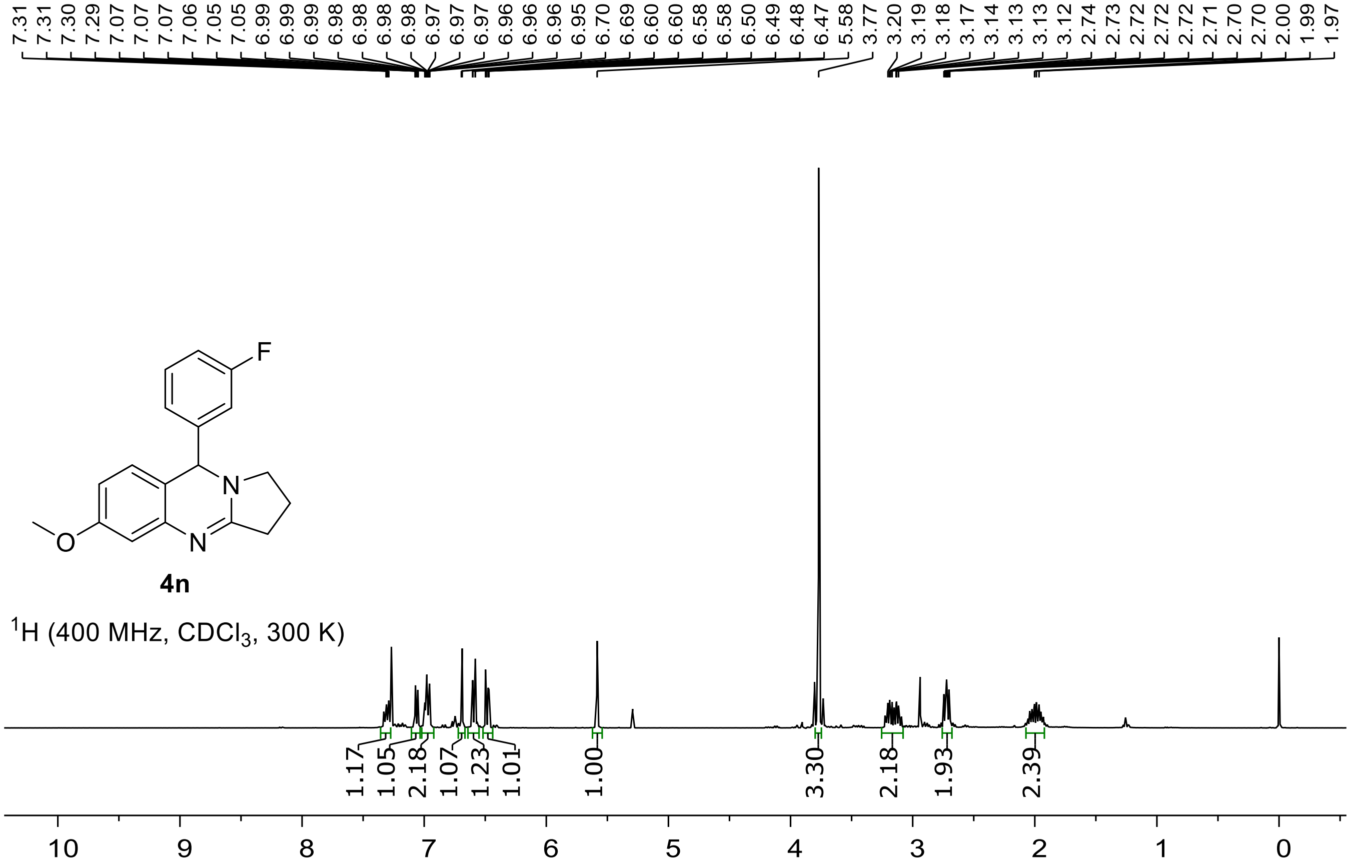
F
N
O
N
4n
1H (400 MHz, CDCl3, 300 K)

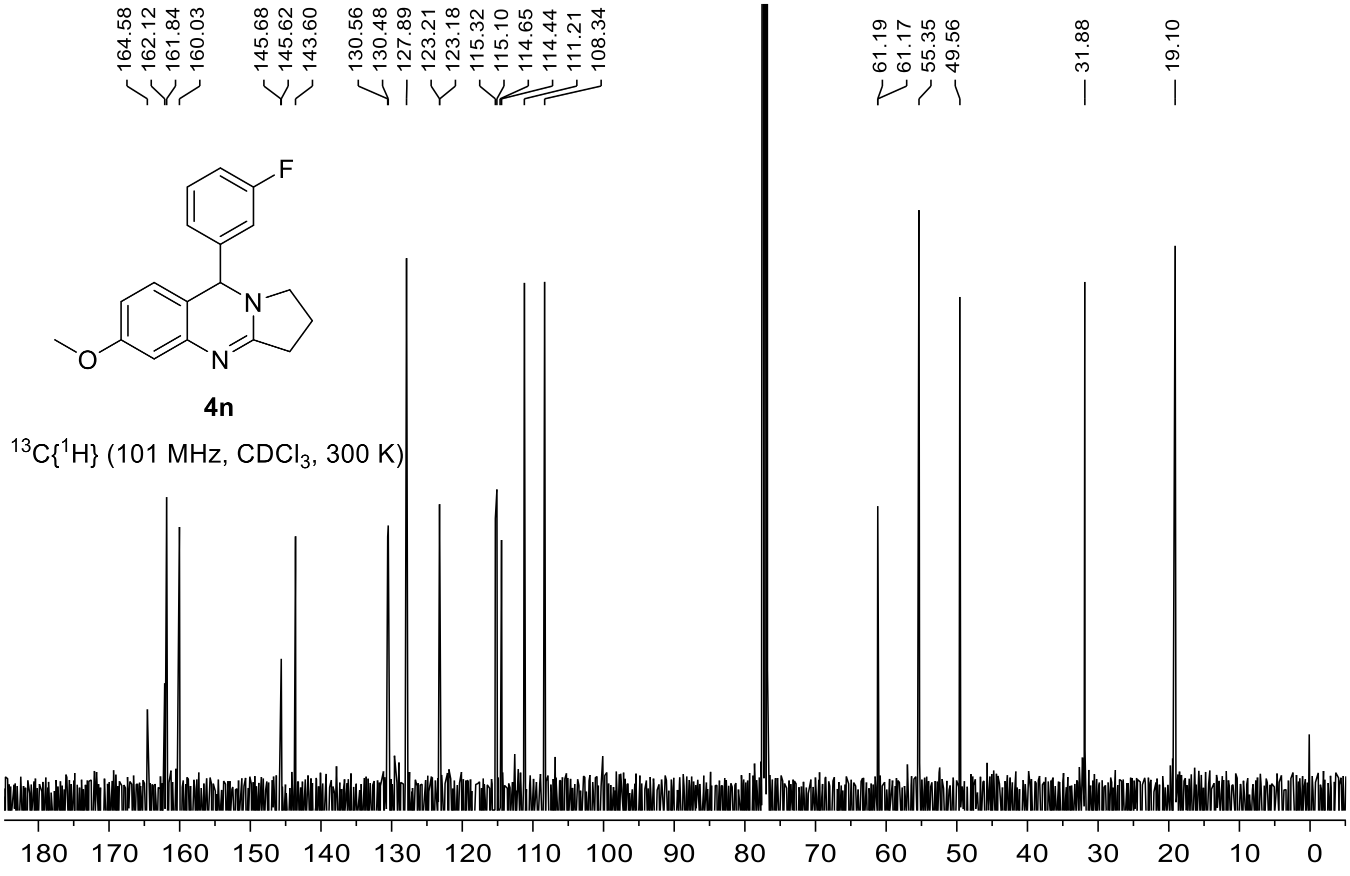
F
N
O
N
4n
13C{1H} (101 MHz, CDCl3, 300 K)

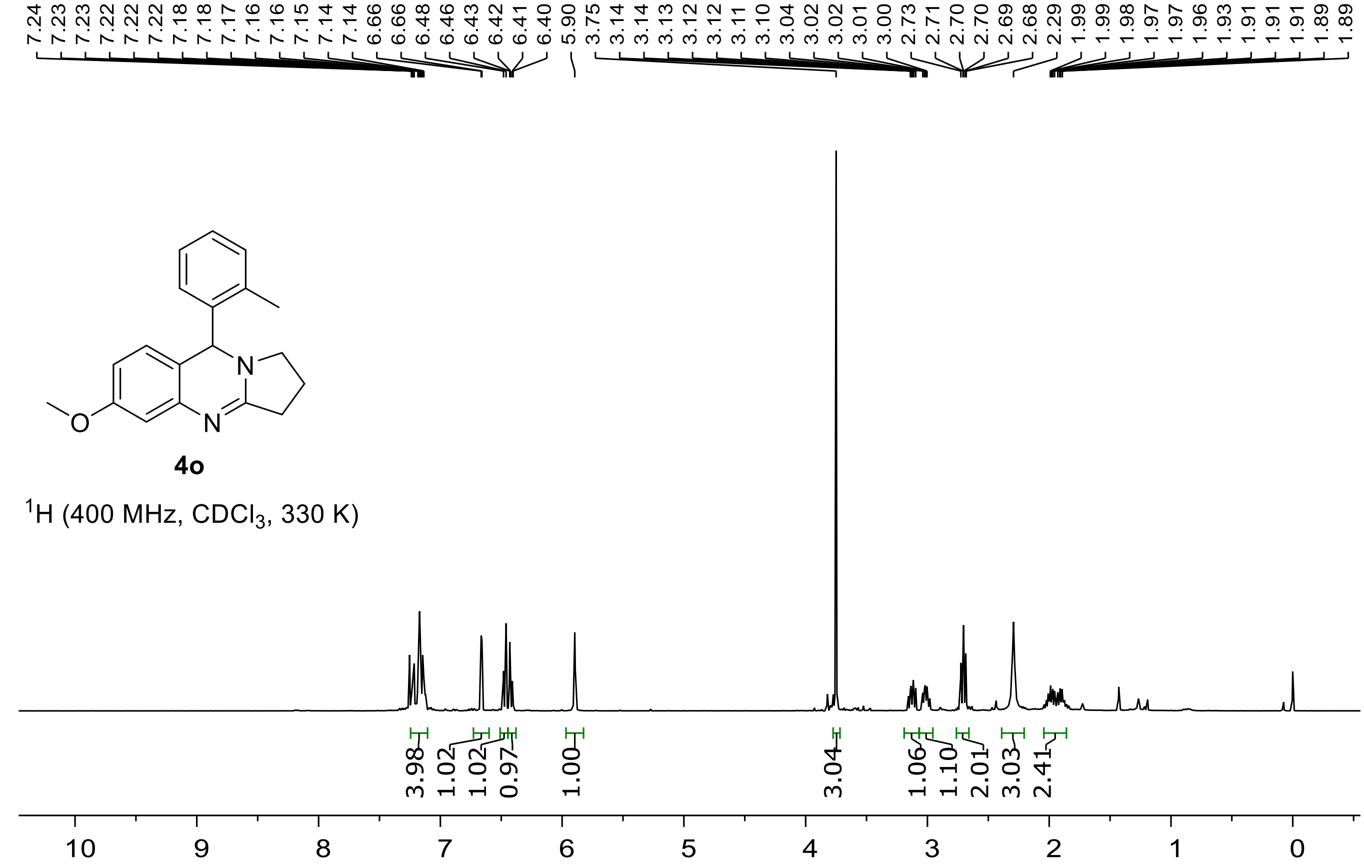

7.24 7.23 7.23 7.22 7.22 7.22 7.18 7.18 7.17 7.16 7.16 7.15 7.14 7.14 6.66 6.66 6.48 6.46 6.43 6.42 6.41 6.40 5.90 3.75 3.14 3.14 3.13 3.12 3.12 3.11 3.10 3.04 3.02 3.02 3.01 3.00 2.73 2.71 2.70 2.70 2.69 2.68 2.29 1.99 1.99 1.98 1.97 1.97 1.96 1.93 1.91 1.91 1.91 1.89 1.89
N
O
N
4o
^{1}H (400 MHz, CDCl$_3$, 330 K)
3.98 1.02 1.02 0.97 1.00 3.04 1.06 1.10 2.01 3.03 2.41
10 9 8 7 6 5 4 3 2 1 0

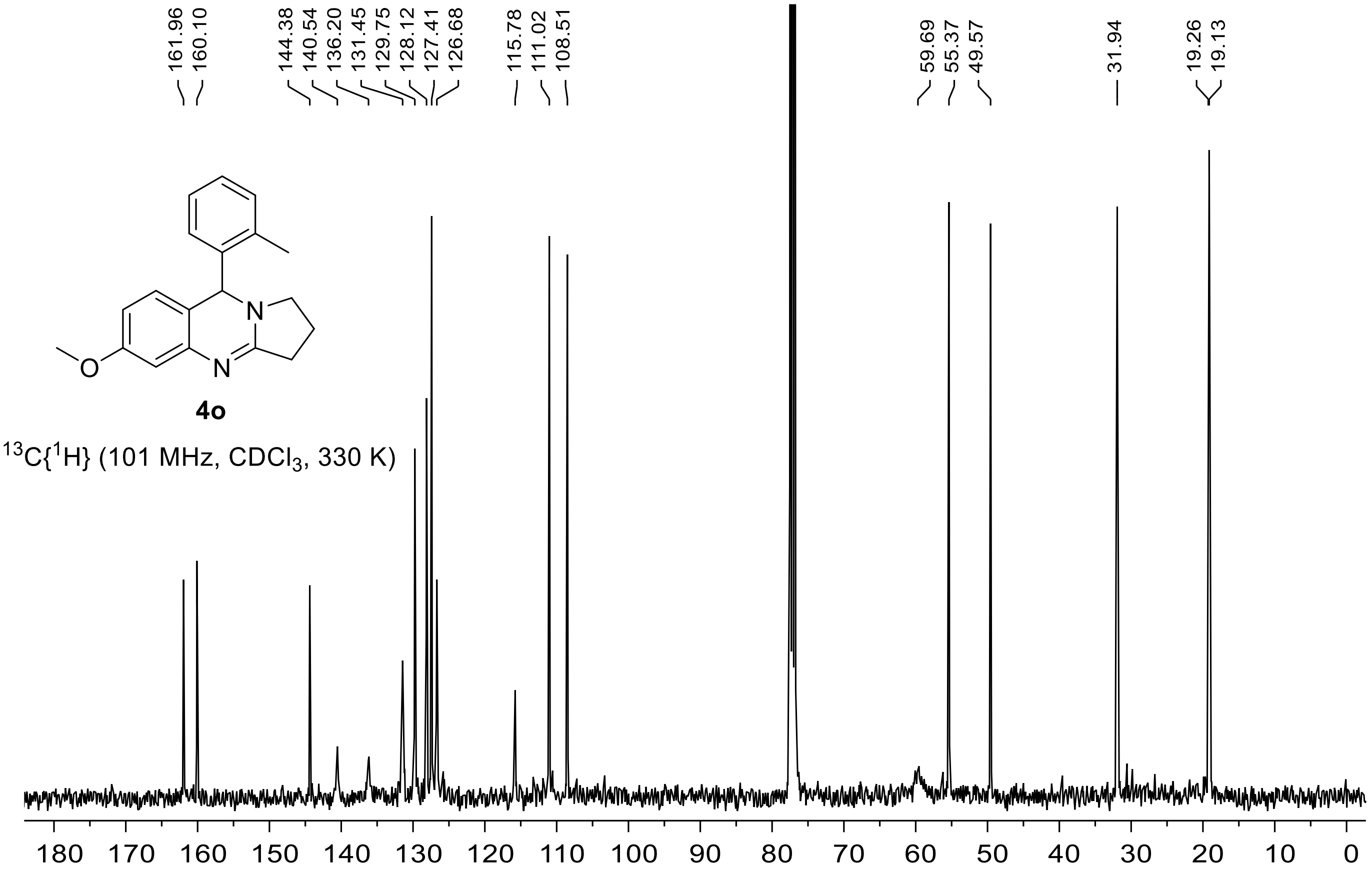

161.96 160.10 144.38 140.54 136.20 131.45 129.75 128.12 127.41 126.68 115.78 111.02 108.51 59.69 55.37 49.57 31.94 19.26 19.13
N
O
N
4o
^{13}C{^{1}H} (101 MHz, CDCl$_3$, 330 K)
180 170 160 150 140 130 120 110 100 90 80 70 60 50 40 30 20 10 0

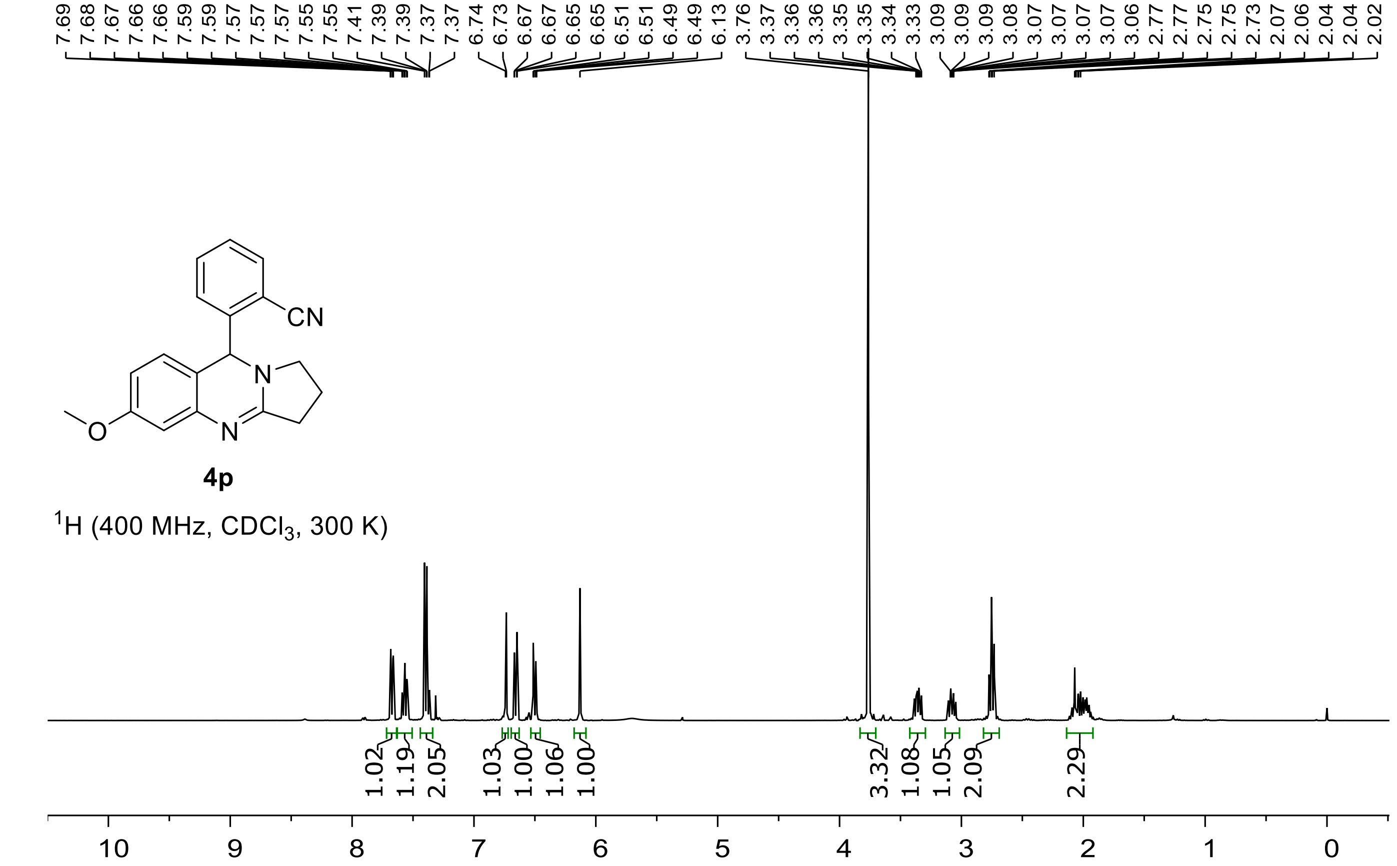
7.69 7.68 7.67 7.66 7.66 7.59 7.59 7.57 7.57 7.57 7.55 7.55 7.41 7.39 7.39 7.37 7.37 6.74 6.73 6.67 6.67 6.65 6.65 6.51 6.51 6.49 6.49 6.13 3.76 3.37 3.36 3.36 3.35 3.35 3.34 3.33 3.09 3.09 3.09 3.08 3.07 3.07 3.07 3.07 3.06 2.77 2.77 2.75 2.75 2.73 2.07 2.06 2.04 2.04 2.02
CN
N
N
O
4p
^{1}H (400 MHz, CDCl$_3$, 300 K)
1.02 1.19 2.05 1.03 1.00 1.06 1.00 3.32 1.08 1.05 2.09 2.29
10 9 8 7 6 5 4 3 2 1 0

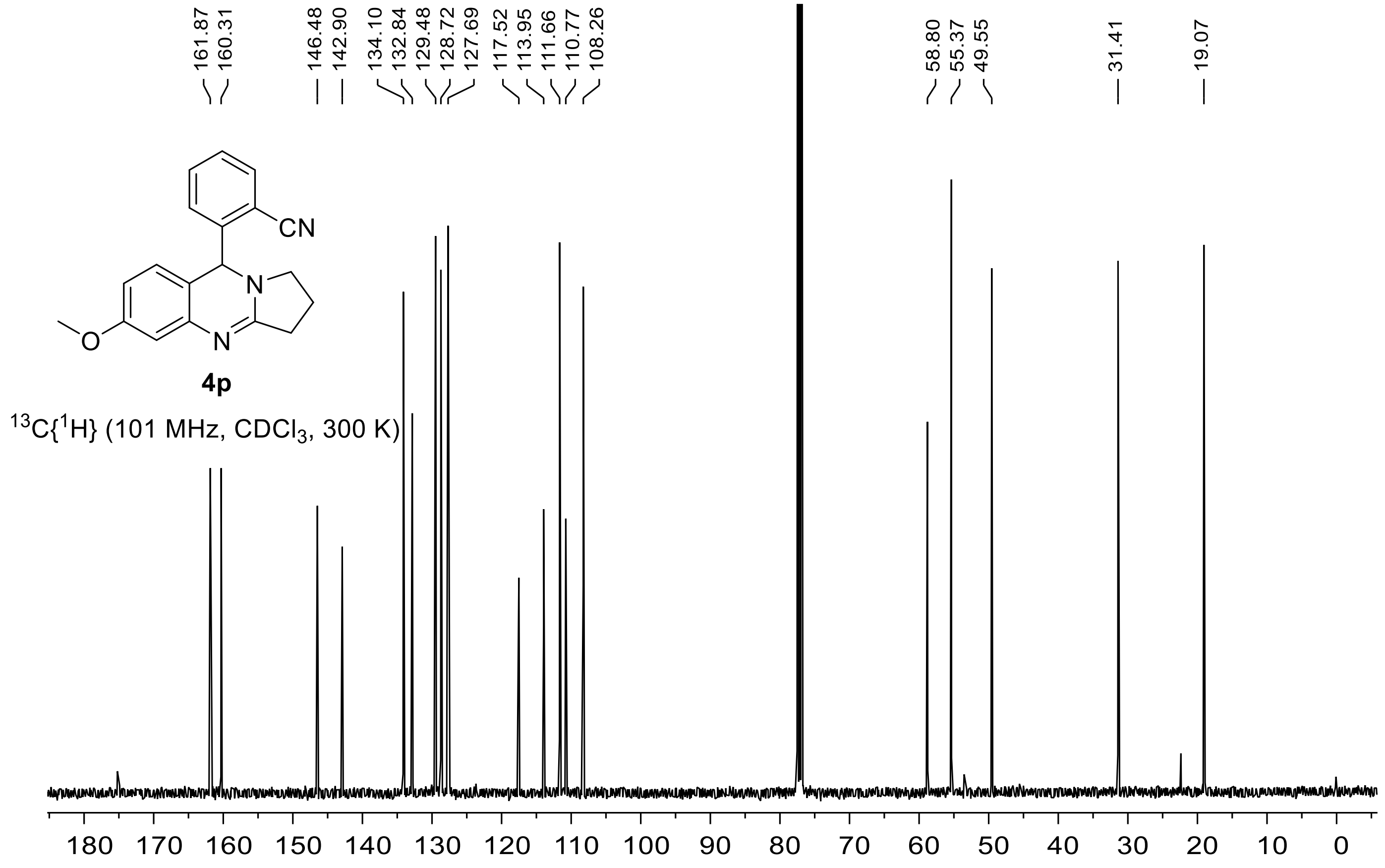
161.87 160.31 146.48 142.90 134.10 132.84 129.48 128.72 127.69 117.52 113.95 111.66 110.77 108.26 58.80 55.37 49.55 31.41 19.07
CN
N
N
O
4p
^{13}C{^{1}H} (101 MHz, CDCl$_3$, 300 K)
180 170 160 150 140 130 120 110 100 90 80 70 60 50 40 30 20 10 0

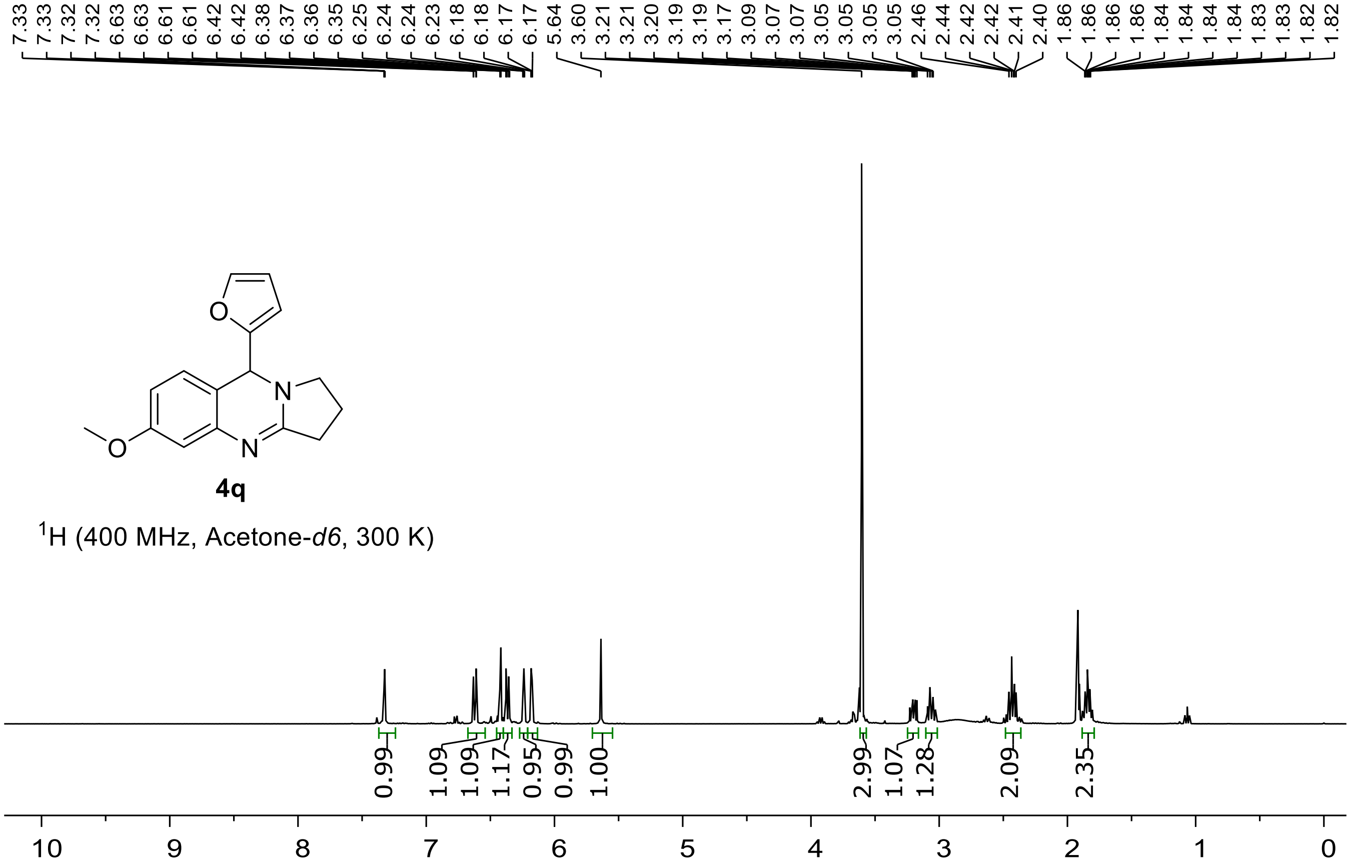
7.33 7.33 7.32 7.32 6.63 6.63 6.61 6.61 6.42 6.42 6.38 6.37 6.36 6.35 6.25 6.24 6.24 6.23 6.18 6.18 6.17 6.17 5.64 3.60 3.21 3.21 3.20 3.19 3.19 3.17 3.09 3.07 3.07 3.05 3.05 3.05 3.05 2.46 2.44 2.42 2.42 2.41 2.40 1.86 1.86 1.86 1.86 1.84 1.84 1.84 1.84 1.83 1.83 1.82 1.82
O
N
N
O
4q
1H (400 MHz, Acetone-d6, 300 K)
0.99 1.09 1.09 1.17 0.95 0.99 1.00 2.99 1.07 1.28 2.09 2.35
10 9 8 7 6 5 4 3 2 1 0

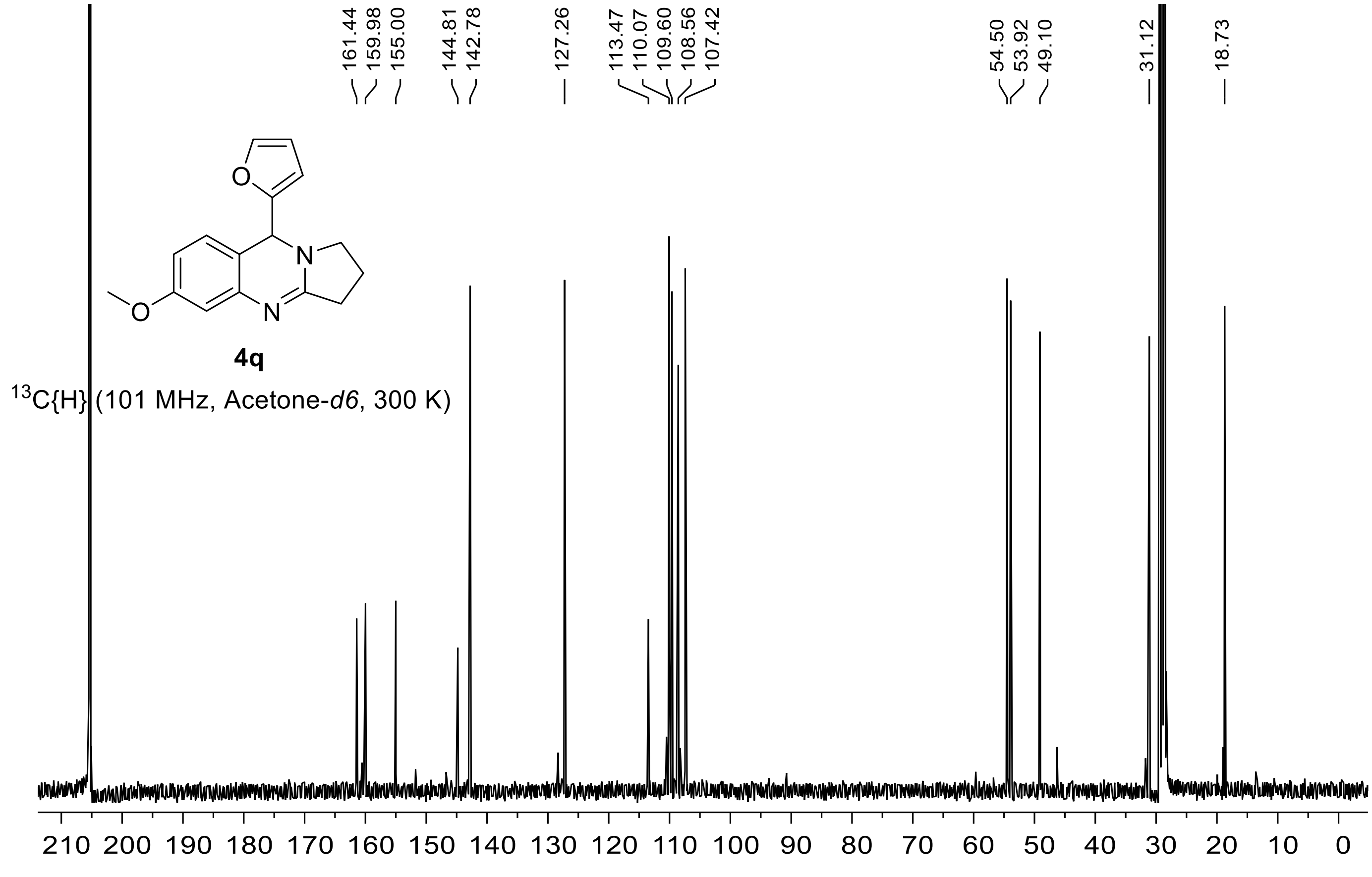
161.44 159.98 155.00 144.81 142.78 127.26 113.47 110.07 109.60 108.56 107.42 54.50 53.92 49.10 31.12 18.73
O
N
N
O
4q
13C{H} (101 MHz, Acetone-d6, 300 K)
210 200 190 180 170 160 150 140 130 120 110 100 90 80 70 60 50 40 30 20 10 0

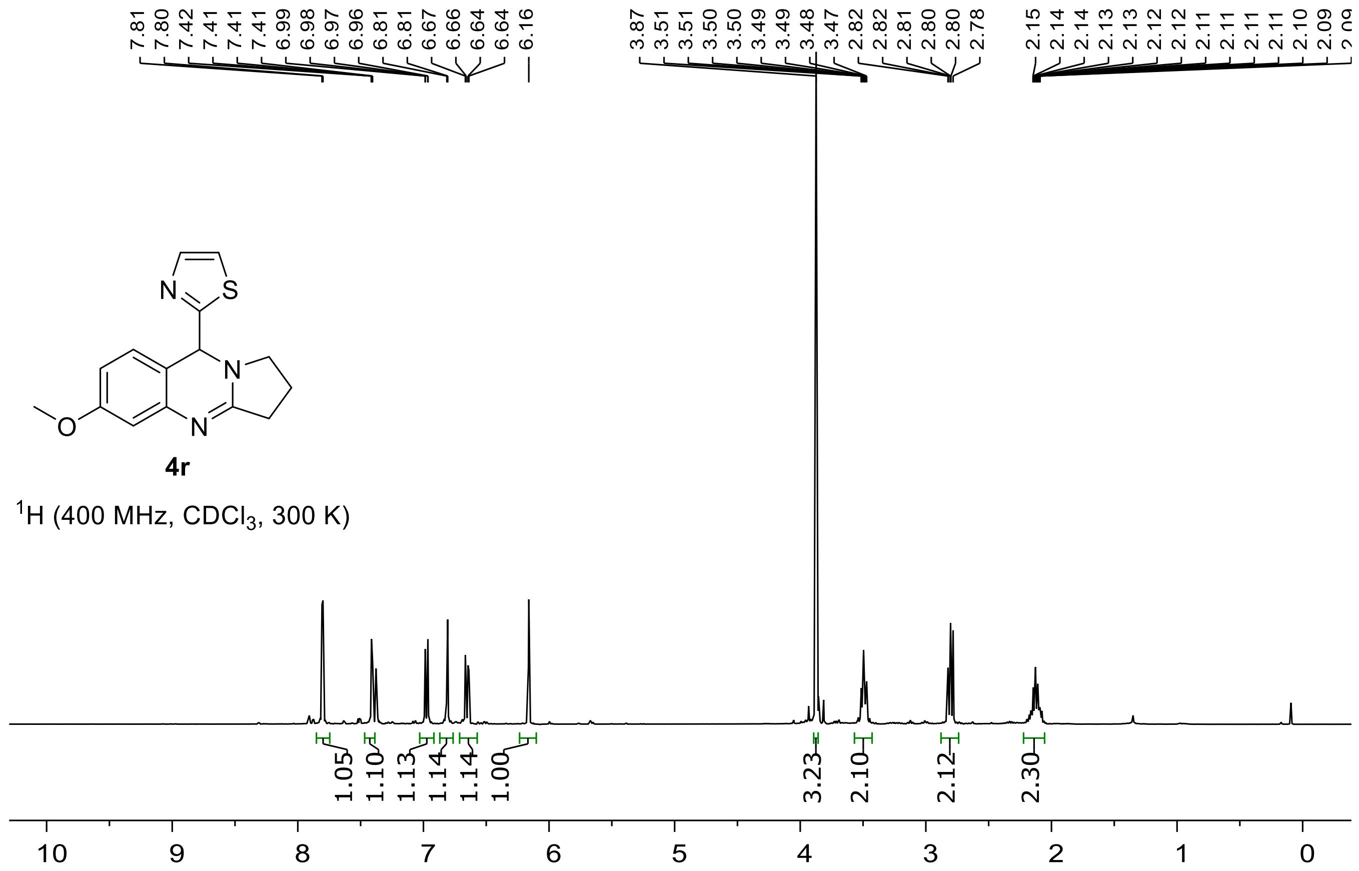
7.81
7.80
7.42
7.41
7.41
7.41
6.99
6.98
6.97
6.96
6.81
6.81
6.67
6.66
6.64
6.64
6.16
3.87
3.51
3.51
3.50
3.50
3.49
3.49
3.48
3.47
2.82
2.82
2.81
2.80
2.80
2.78
2.15
2.14
2.14
2.13
2.13
2.12
2.12
2.11
2.11
2.11
2.11
2.10
2.09
N
S
N
N
O
4r
^{1}H (400 MHz, CDCl$_3$, 300 K)
1.05
1.10
1.13
1.14
1.14
1.00
3.23
2.10
2.12
2.30
10
9
8
7
6
5
4
3
2
1
0

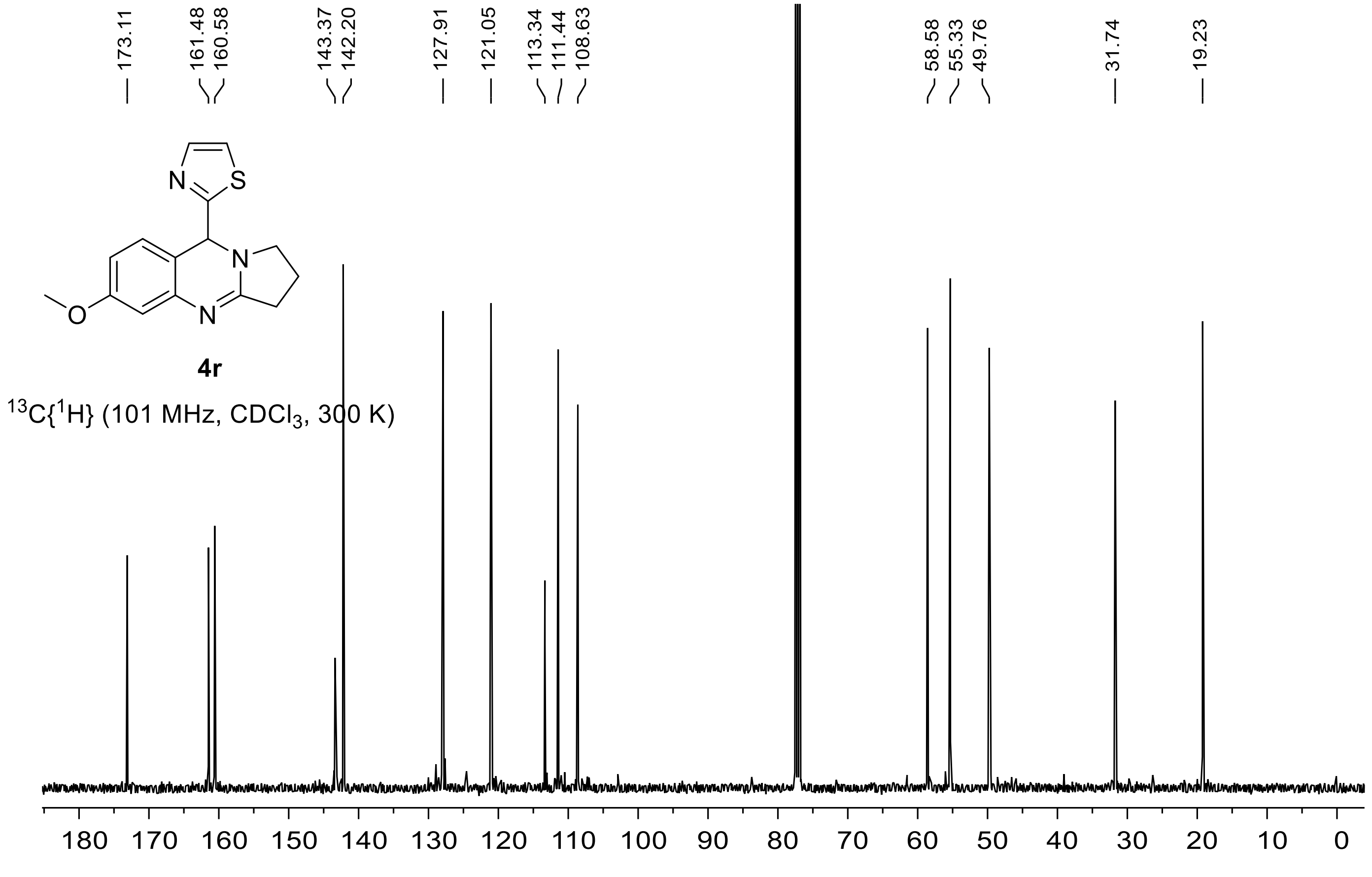
173.11
161.48
160.58
143.37
142.20
127.91
121.05
113.34
111.44
108.63
58.58
55.33
49.76
31.74
19.23
N
S
N
N
O
4r
^{13}C{^{1}H} (101 MHz, CDCl$_3$, 300 K)
180
170
160
150
140
130
120
110
100
90
80
70
60
50
40
30
20
10
0

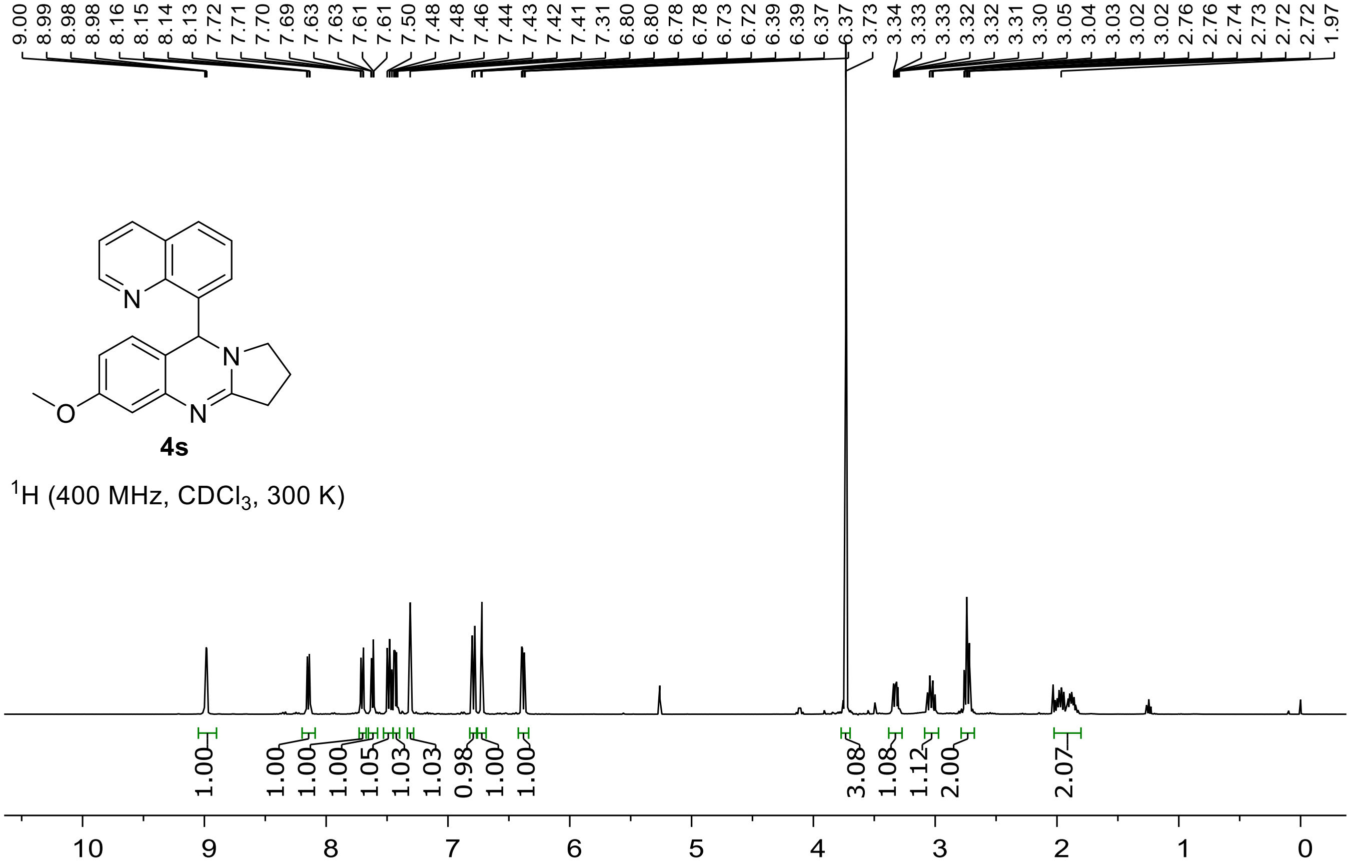
9.00 8.99 8.98 8.98 8.16 8.15 8.14 8.13 7.72 7.71 7.70 7.69 7.63 7.63 7.61 7.61 7.50 7.48 7.48 7.46 7.44 7.43 7.42 7.41 7.31 6.80 6.80 6.78 6.78 6.73 6.72 6.39 6.39 6.37 6.37 3.73 3.34 3.33 3.33 3.32 3.32 3.31 3.30 3.05 3.04 3.03 3.02 3.02 2.76 2.74 2.73 2.72 2.72 1.97
N
N
N
O
4s
^{1}H (400 MHz, $CDCl_3$, 300 K)
1.00 1.00 1.00 1.00 1.05 1.03 1.03 0.98 1.00 1.00 3.08 1.08 1.12 2.00 2.07
10 9 8 7 6 5 4 3 2 1 0

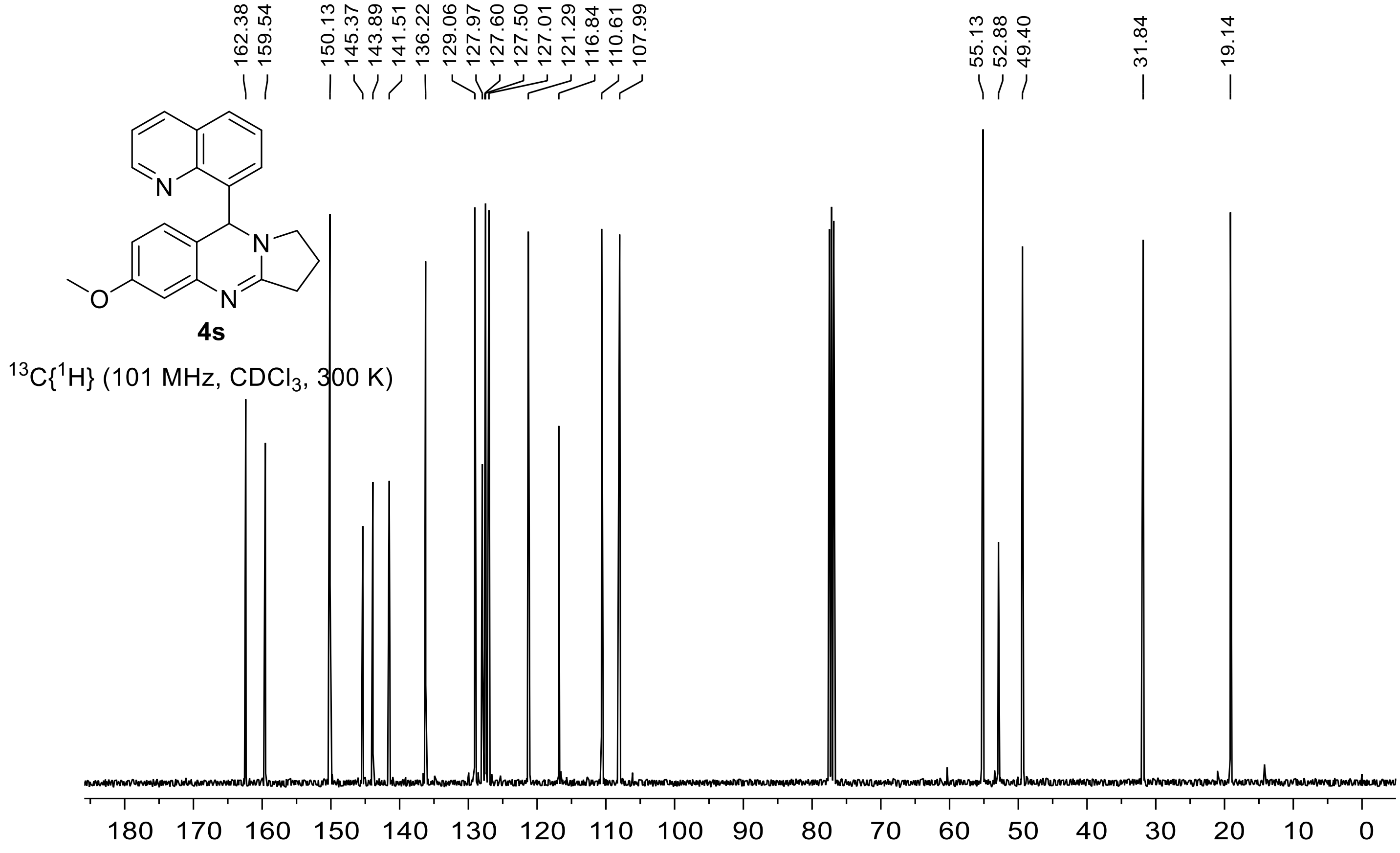
162.38 159.54 150.13 145.37 143.89 141.51 136.22 129.06 127.97 127.60 127.50 127.01 121.29 116.84 110.61 107.99 55.13 52.88 49.40 31.84 19.14
N
N
N
O
4s
$^{13}C\{^{1}H\}$ (101 MHz, $CDCl_3$, 300 K)
180 170 160 150 140 130 120 110 100 90 80 70 60 50 40 30 20 10 0

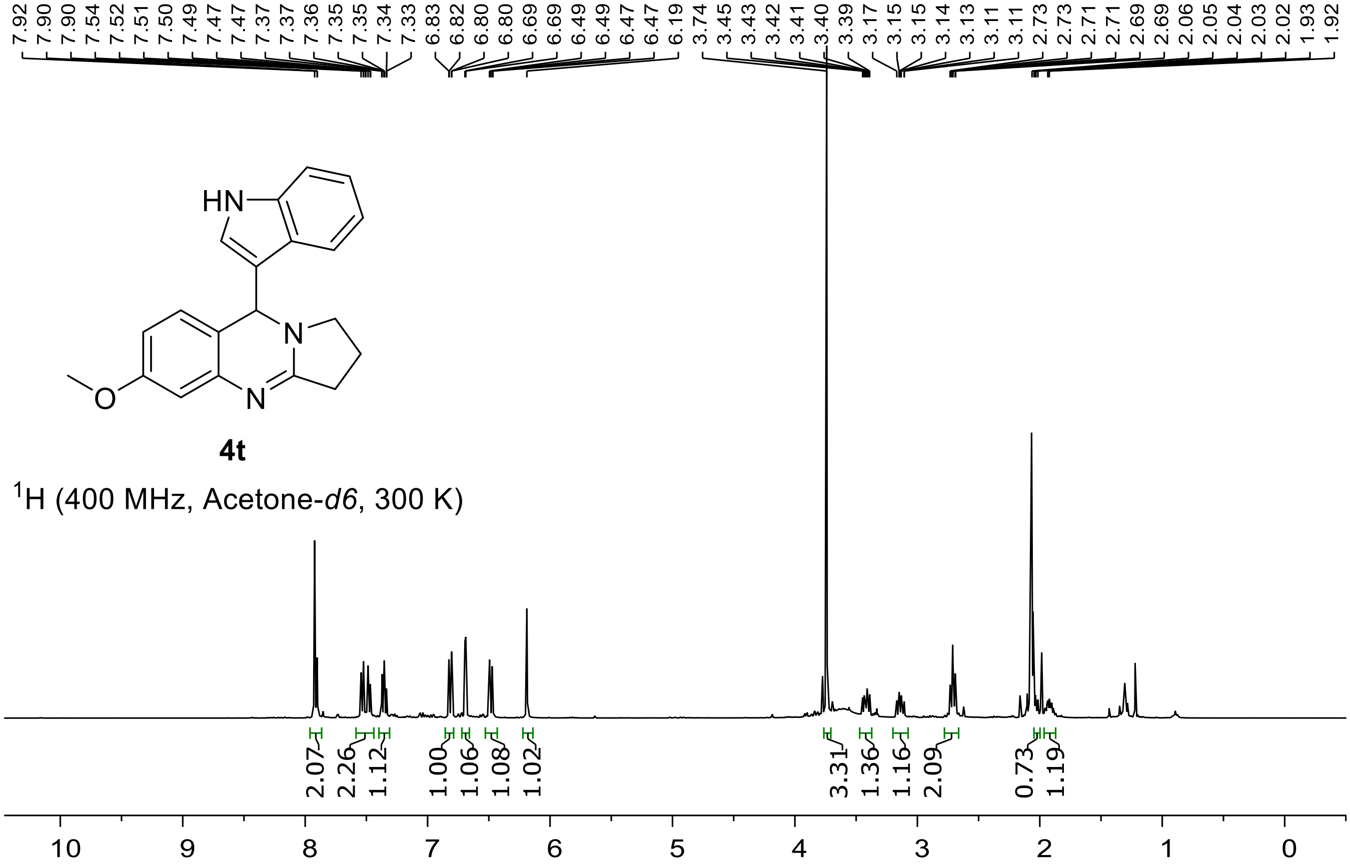
7.92 7.90 7.90 7.54 7.52 7.51 7.50 7.49 7.47 7.47 7.37 7.37 7.36 7.35 7.35 7.34 7.33 6.83 6.82 6.80 6.80 6.69 6.69 6.49 6.49 6.47 6.47 6.19 3.74 3.45 3.43 3.42 3.41 3.40 3.39 3.17 3.15 3.15 3.14 3.13 3.11 3.11 2.73 2.73 2.71 2.71 2.69 2.69 2.06 2.05 2.04 2.03 2.02 1.93 1.92
HN
N
O
N
4t
^{1}H (400 MHz, Acetone-*d6*, 300 K)
2.07 2.26 1.12 1.00 1.06 1.08 1.02 3.31 1.36 1.16 2.09 0.73 1.19
10 9 8 7 6 5 4 3 2 1 0

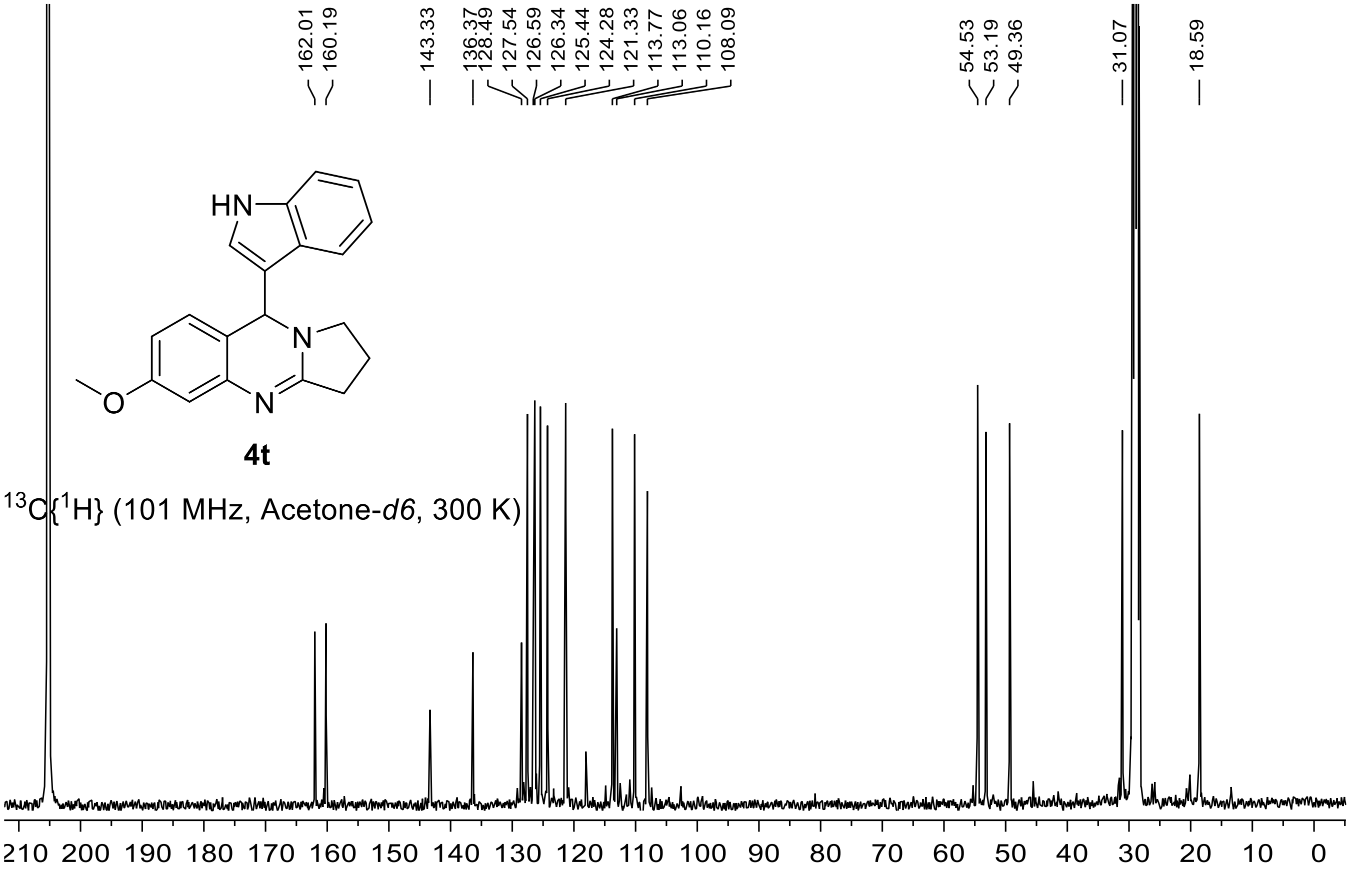
162.01 160.19 143.33 136.37 128.49 127.54 126.59 126.34 125.44 124.28 121.33 113.77 113.06 110.16 108.09 54.53 53.19 49.36 31.07 18.59
HN
N
O
N
4t
^{13}C{^{1}H} (101 MHz, Acetone-*d6*, 300 K)
210 200 190 180 170 160 150 140 130 120 110 100 90 80 70 60 50 40 30 20 10 0

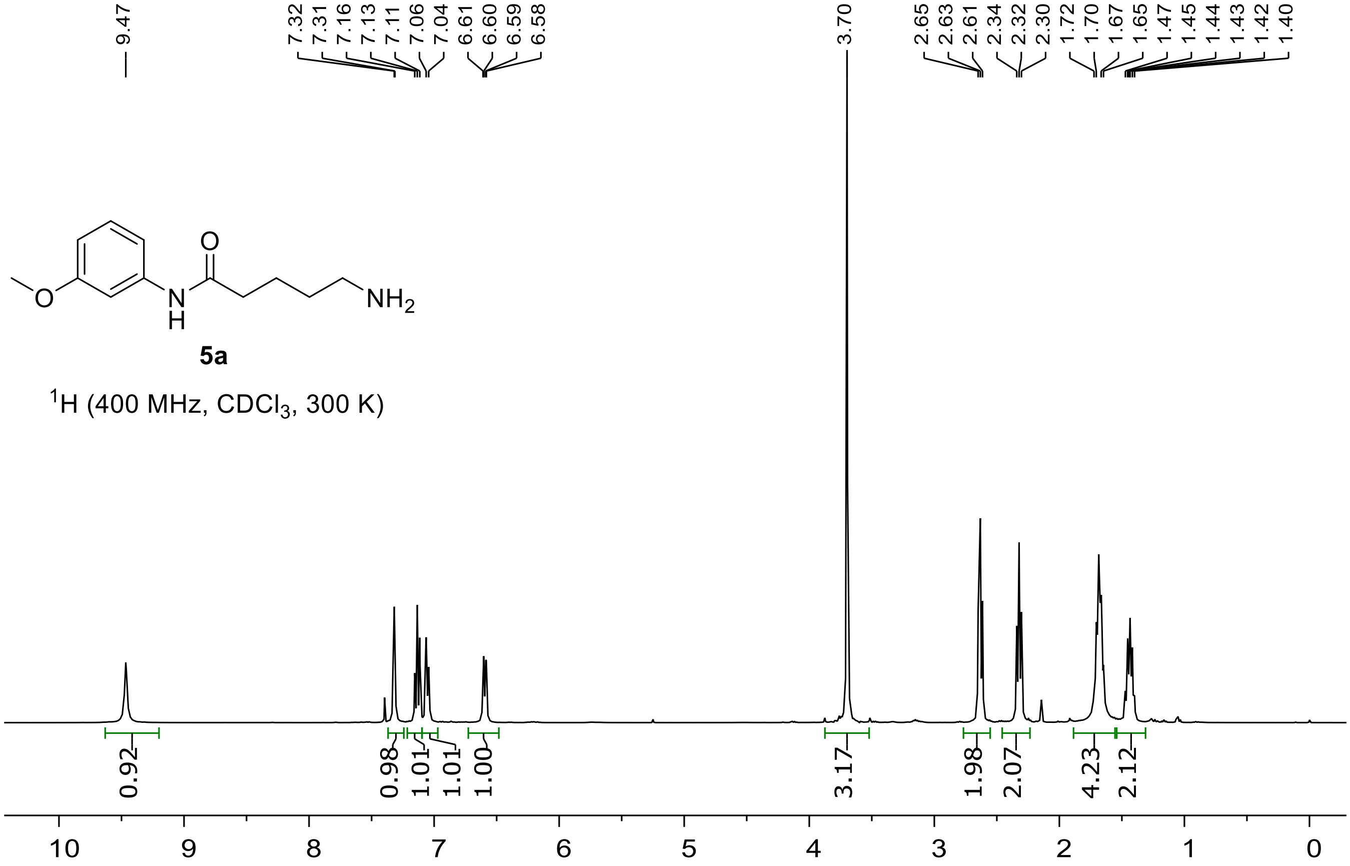
9.47
7.32 7.31 7.16 7.13 7.11 7.06 7.04 6.61 6.60 6.59 6.58
3.70
2.65 2.63 2.61 2.34 2.32 2.30 1.72 1.70 1.67 1.65 1.47 1.45 1.44 1.43 1.42 1.40
O
N
H
NH2
5a
1H (400 MHz, CDCl3, 300 K)
0.92
0.98 1.01 1.01 1.00
3.17
1.98 2.07 4.23 2.12
10 9 8 7 6 5 4 3 2 1 0

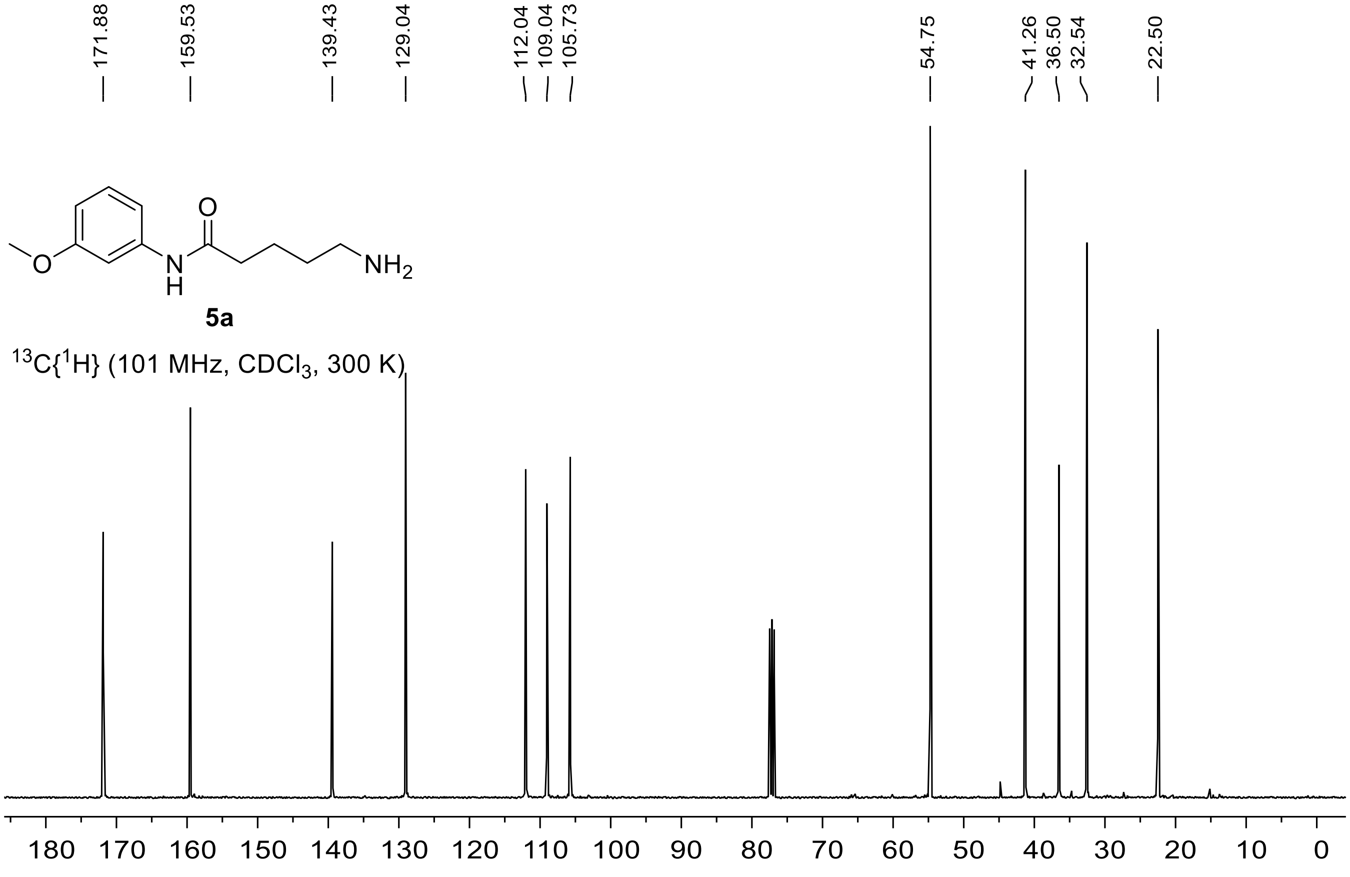
171.88
159.53
139.43
129.04
112.04 109.04 105.73
54.75
41.26 36.50 32.54
22.50
O
N
H
NH2
5a
13C{1H} (101 MHz, CDCl3, 300 K)
180 170 160 150 140 130 120 110 100 90 80 70 60 50 40 30 20 10 0

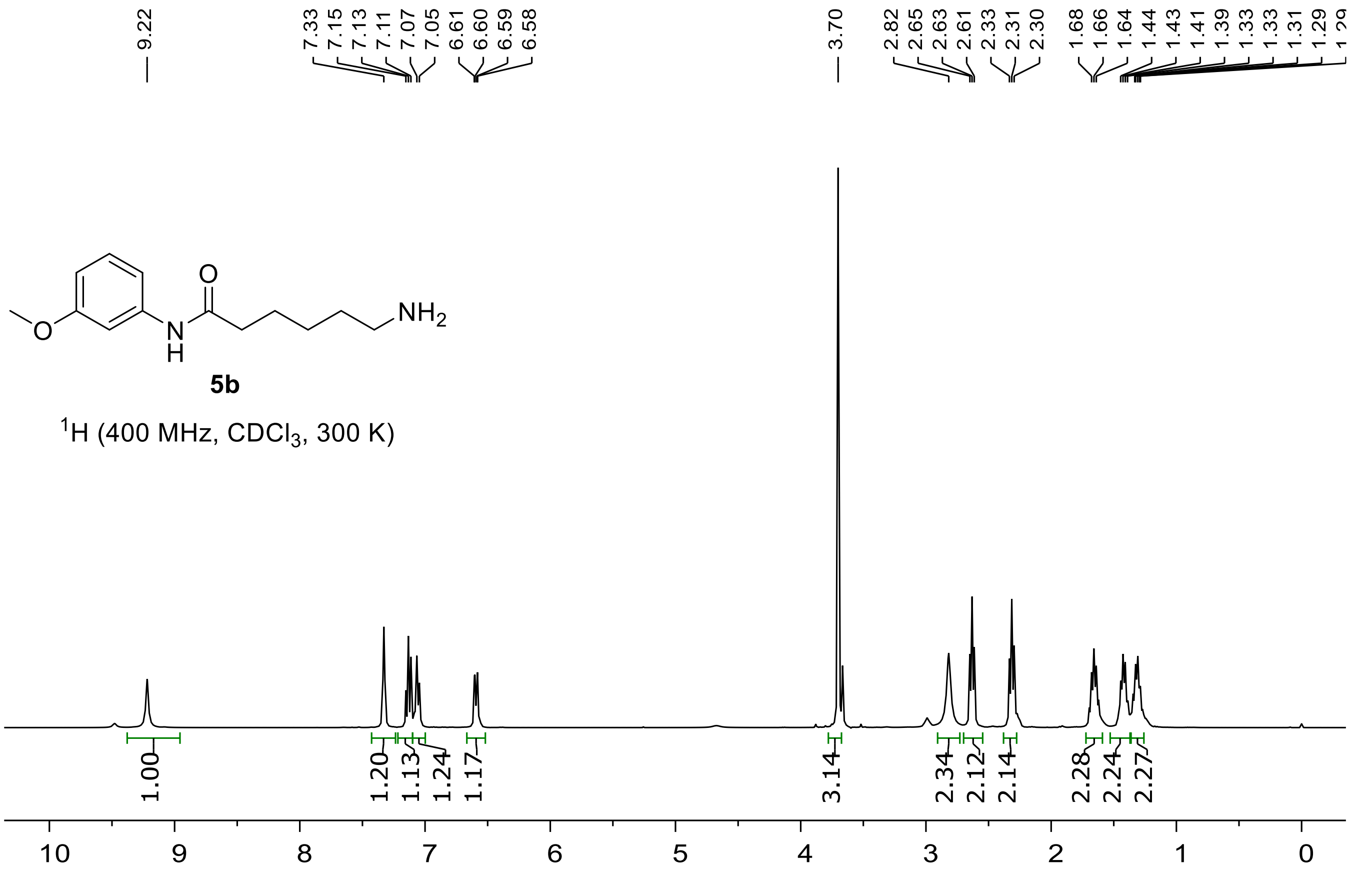
9.22
7.33
7.15
7.13
7.11
7.07
7.05
6.61
6.60
6.59
6.58
3.70
2.82
2.65
2.63
2.61
2.33
2.31
2.30
1.68
1.66
1.64
1.44
1.43
1.41
1.39
1.33
1.33
1.31
1.29
O
O
N
H
NH2
5b
^{1}H (400 MHz, CDCl$_3$, 300 K)
1.00
1.20
1.13
1.24
1.17
3.14
2.34
2.12
2.14
2.28
2.24
2.27
10 9 8 7 6 5 4 3 2 1 0

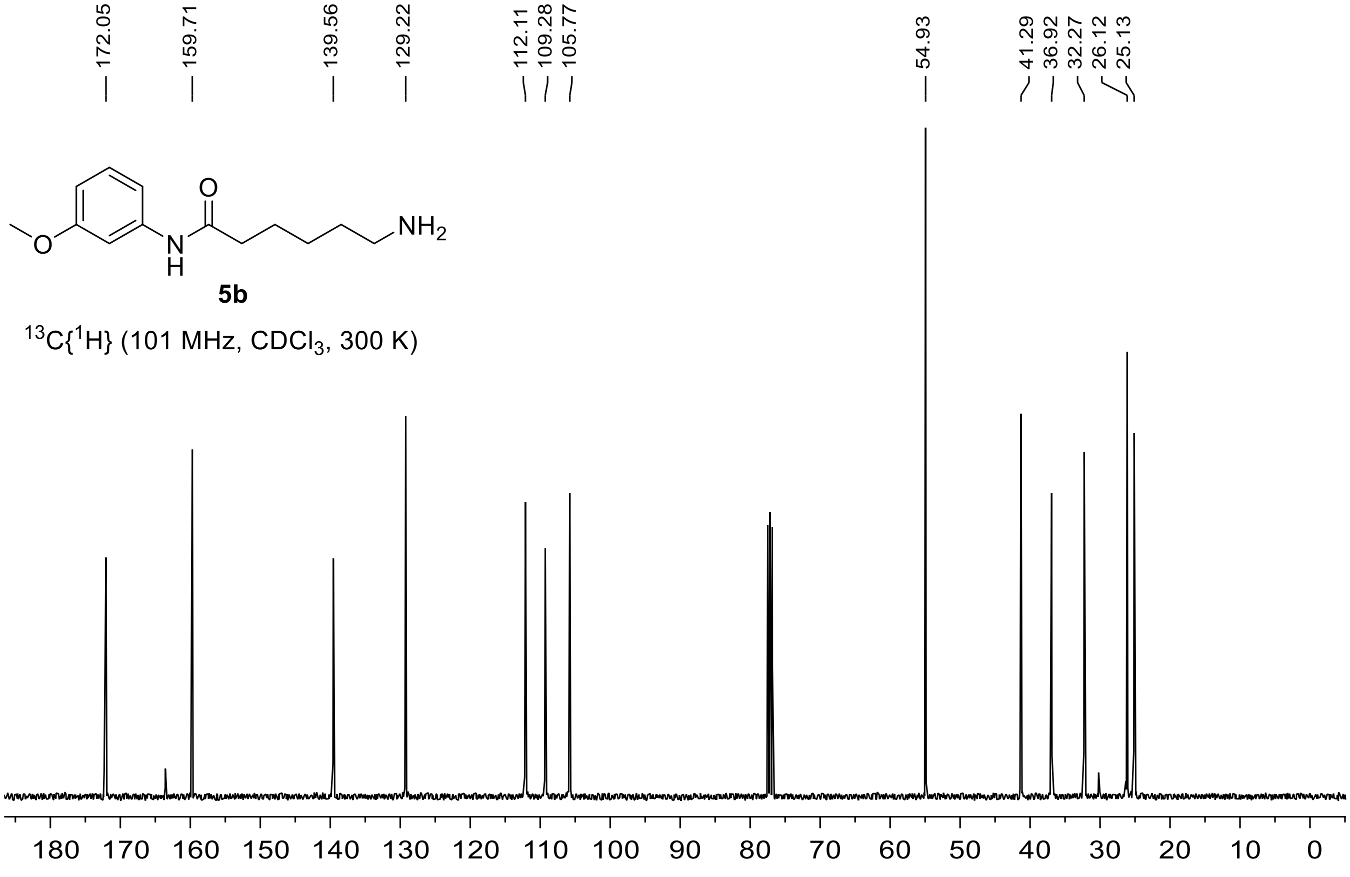
172.05
159.71
139.56
129.22
112.11
109.28
105.77
54.93
41.29
36.92
32.27
26.12
25.13
O
O
N
H
NH2
5b
^{13}C{^{1}H} (101 MHz, CDCl$_3$, 300 K)
180 170 160 150 140 130 120 110 100 90 80 70 60 50 40 30 20 10 0

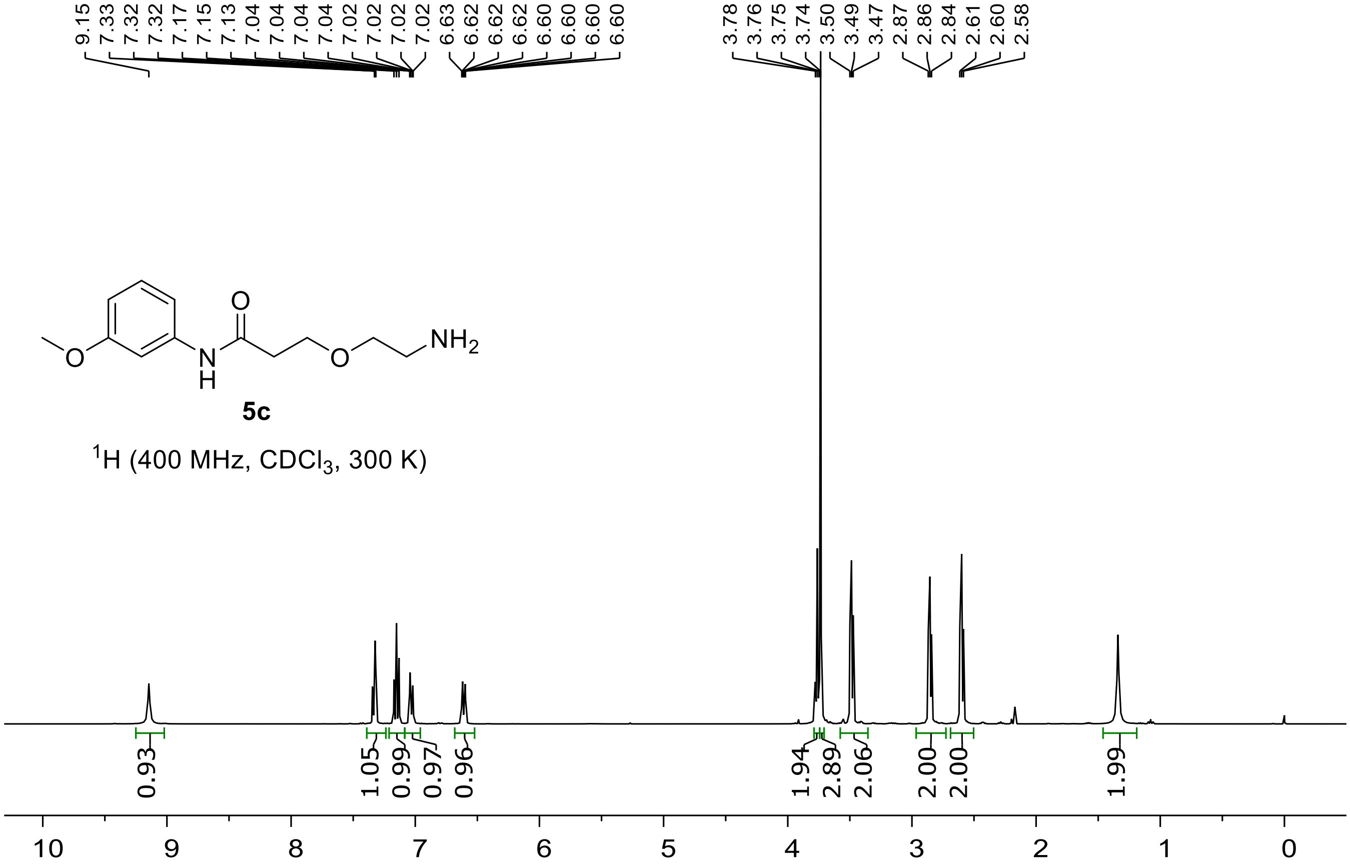

9.15 7.33 7.32 7.32 7.17 7.15 7.13 7.04 7.04 7.04 7.04 7.02 7.02 7.02 7.02 6.63 6.62 6.62 6.62 6.60 6.60 6.60 6.60
3.78 3.76 3.75 3.74 3.50 3.49 3.47 2.87 2.86 2.84 2.61 2.60 2.58
O
N
H
O
NH2
5c
1H (400 MHz, CDCl3, 300 K)
0.93 1.05 0.99 0.97 0.96 1.94 2.89 2.06 2.00 2.00 1.99
10 9 8 7 6 5 4 3 2 1 0

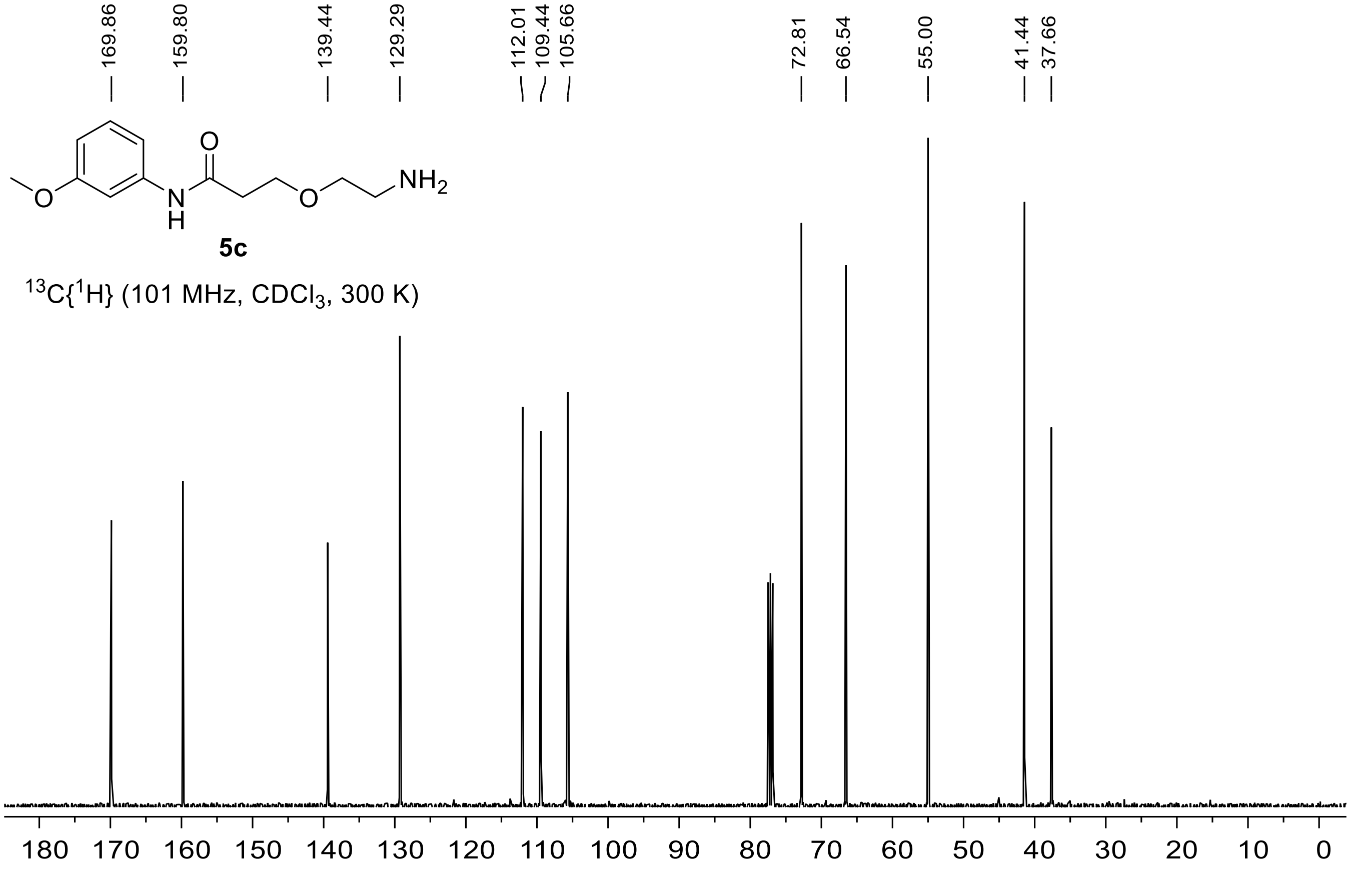

169.86 159.80 139.44 129.29 112.01 109.44 105.66 72.81 66.54 55.00 41.44 37.66
O
N
H
O
NH2
5c
13C{1H} (101 MHz, CDCl3, 300 K)
180 170 160 150 140 130 120 110 100 90 80 70 60 50 40 30 20 10 0

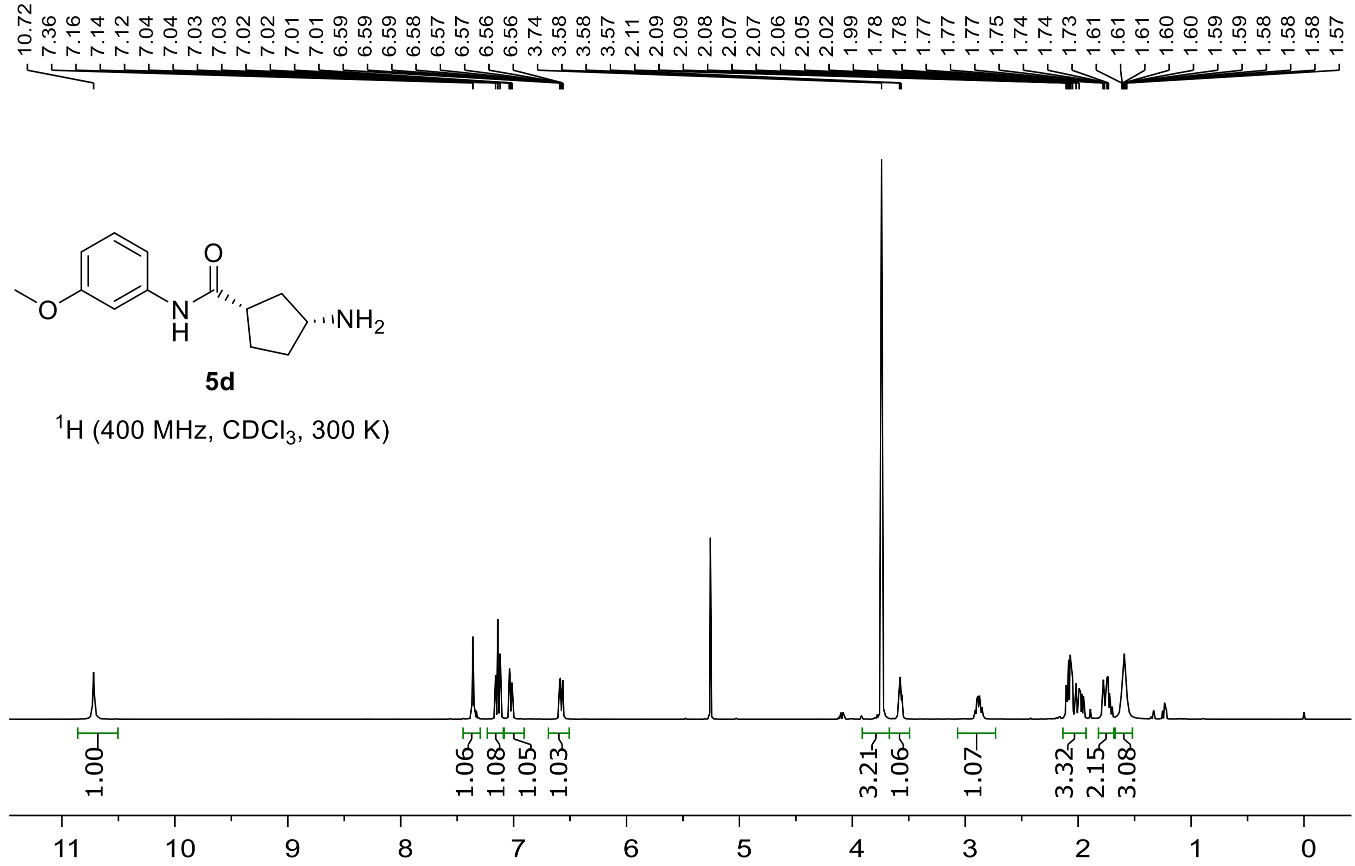
10.72 7.36 7.16 7.14 7.12 7.04 7.04 7.03 7.03 7.02 7.02 7.01 7.01 6.59 6.59 6.59 6.58 6.57 6.57 6.56 6.56 3.74 3.58 3.58 3.57 2.11 2.09 2.09 2.08 2.07 2.07 2.06 2.05 2.02 1.99 1.78 1.78 1.77 1.77 1.77 1.75 1.74 1.74 1.73 1.61 1.61 1.61 1.60 1.60 1.59 1.59 1.58 1.58 1.58 1.57
O
N
H
NH2
5d
1H (400 MHz, CDCl3, 300 K)
1.00 1.06 1.08 1.05 1.03 3.21 1.06 1.07 3.32 2.15 3.08
11 10 9 8 7 6 5 4 3 2 1 0

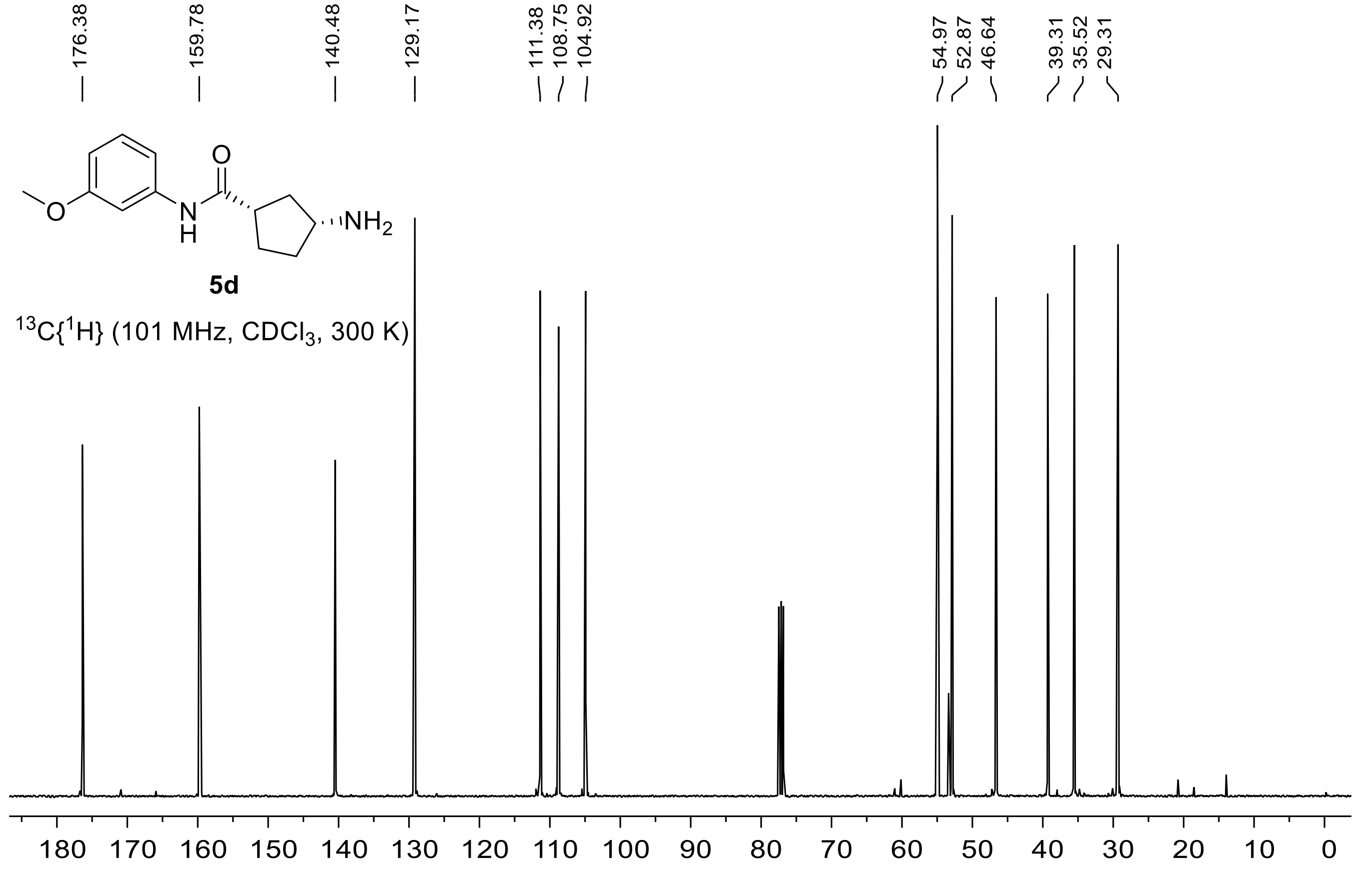
176.38 159.78 140.48 129.17 111.38 108.75 104.92 54.97 52.87 46.64 39.31 35.52 29.31
O
N
H
NH2
5d
13C{1H} (101 MHz, CDCl3, 300 K)
180 170 160 150 140 130 120 110 100 90 80 70 60 50 40 30 20 10 0

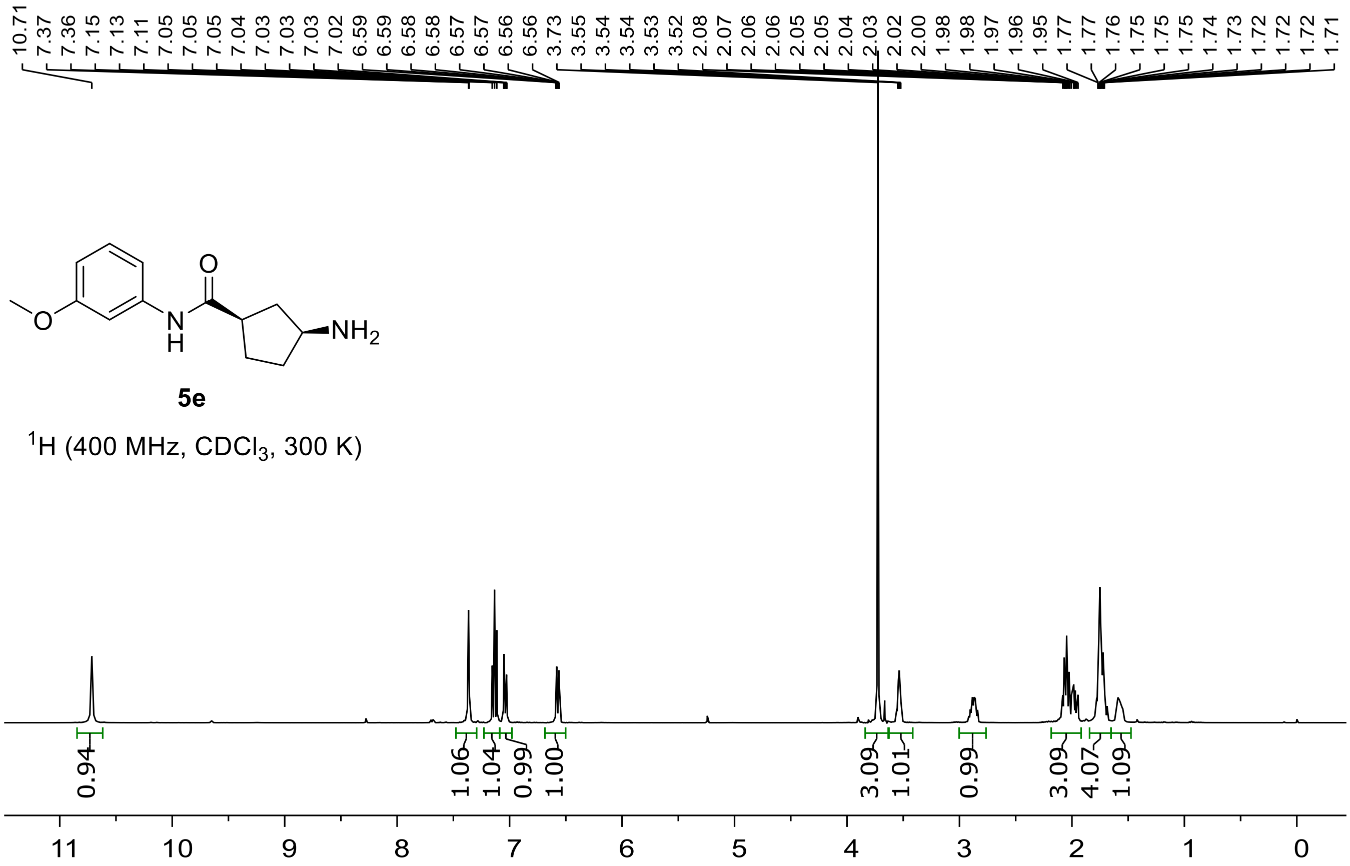

10.71
7.37
7.36
7.15
7.13
7.11
7.05
7.05
7.05
7.04
7.03
7.03
7.03
7.02
6.59
6.59
6.58
6.58
6.57
6.57
6.56
6.56
3.73
3.55
3.54
3.54
3.53
3.52
2.08
2.07
2.06
2.06
2.05
2.05
2.04
2.03
2.02
2.00
1.98
1.98
1.97
1.96
1.95
1.77
1.77
1.76
1.75
1.75
1.75
1.74
1.73
1.72
1.72
1.72
1.71
O
N
H
NH2
O
5e
1H (400 MHz, CDCl3, 300 K)
0.94
1.06
1.04
0.99
1.00
3.09
1.01
0.99
3.09
4.07
1.09
11
10
9
8
7
6
5
4
3
2
1
0

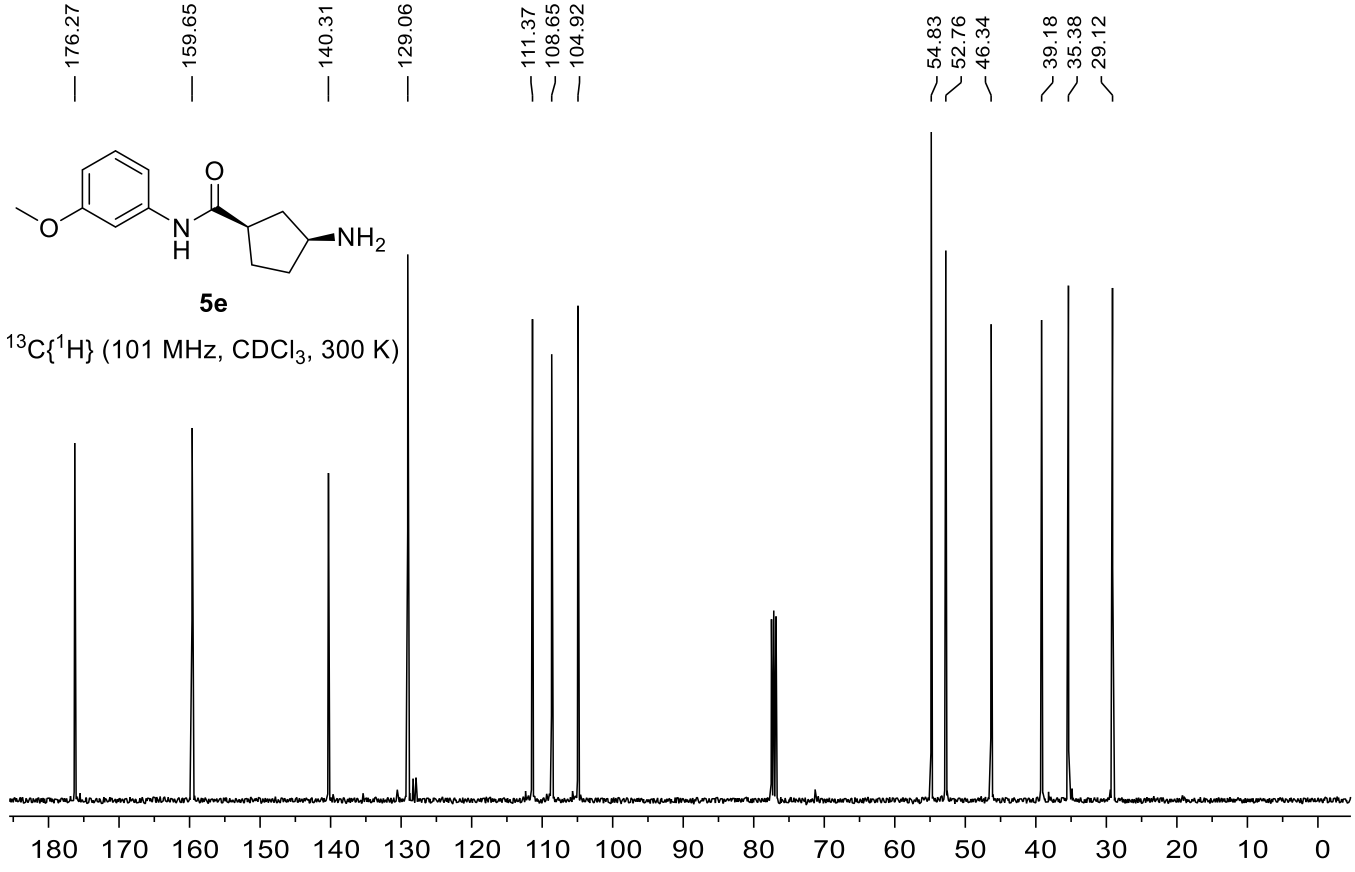

176.27
159.65
140.31
129.06
111.37
108.65
104.92
54.83
52.76
46.34
39.18
35.38
29.12
O
N
H
NH2
O
5e
13C{1H} (101 MHz, CDCl3, 300 K)
180
170
160
150
140
130
120
110
100
90
80
70
60
50
40
30
20
10
0

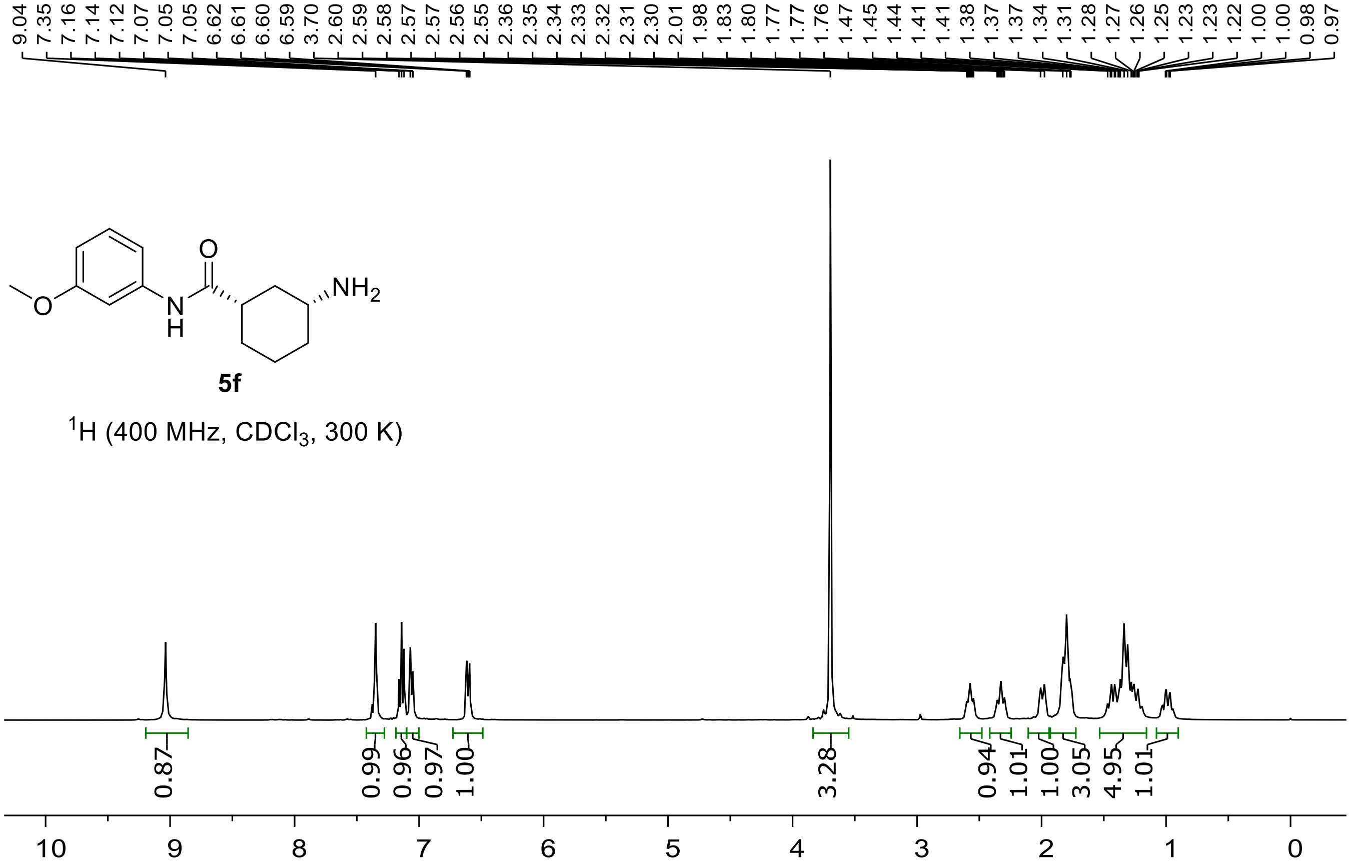
9.04
7.35
7.16
7.14
7.12
7.07
7.05
6.62
6.61
6.60
6.59
3.70
2.60
2.59
2.58
2.57
2.57
2.56
2.55
2.36
2.35
2.34
2.33
2.32
2.31
2.30
2.01
1.98
1.83
1.80
1.77
1.77
1.76
1.47
1.45
1.44
1.41
1.41
1.38
1.37
1.37
1.34
1.31
1.28
1.27
1.26
1.25
1.23
1.22
1.00
1.00
0.98
0.97
O
NH$_2$
N
H
O
5f
^{1}H (400 MHz, CDCl$_3$, 300 K)
0.87
0.99
0.96
0.97
1.00
3.28
0.94
1.01
1.00
3.05
4.95
1.01
10
9
8
7
6
5
4
3
2
1
0

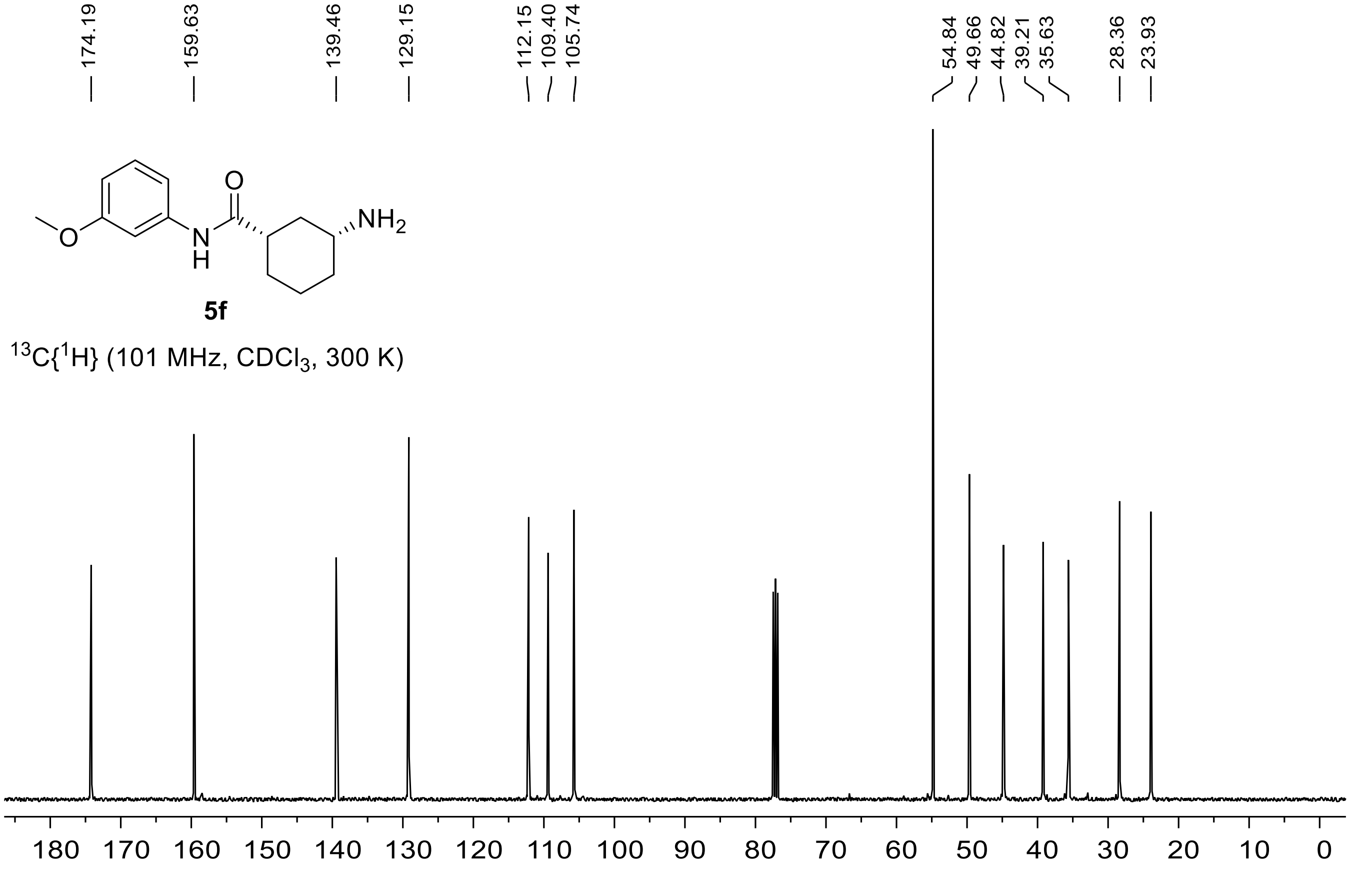
174.19
159.63
139.46
129.15
112.15
109.40
105.74
54.84
49.66
44.82
39.21
35.63
28.36
23.93
O
NH$_2$
N
H
O
5f
^{13}C{^{1}H} (101 MHz, CDCl$_3$, 300 K)
180
170
160
150
140
130
120
110
100
90
80
70
60
50
40
30
20
10
0

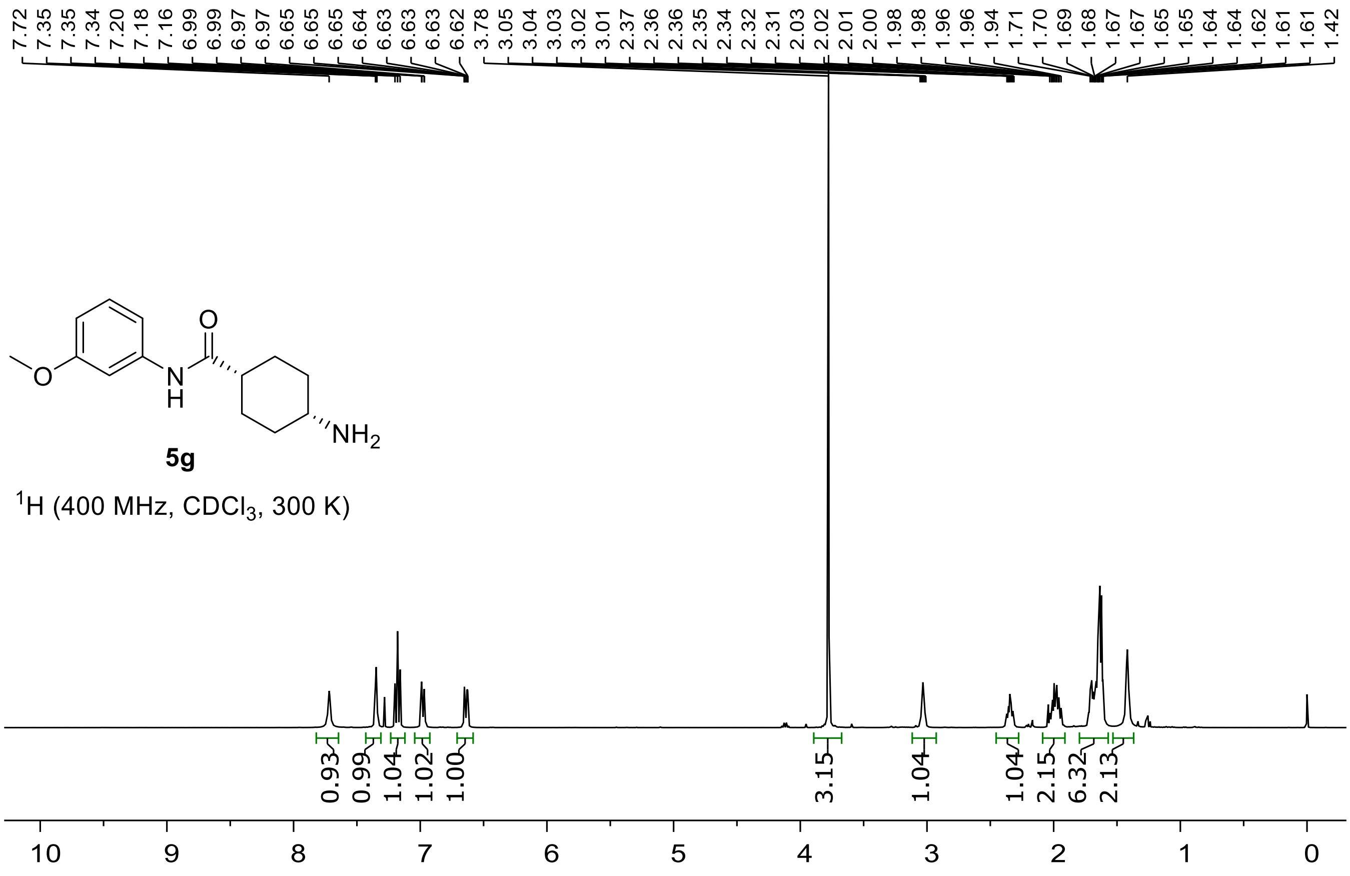

5g
^{1}H (400 MHz, CDCl$_3$, 300 K)

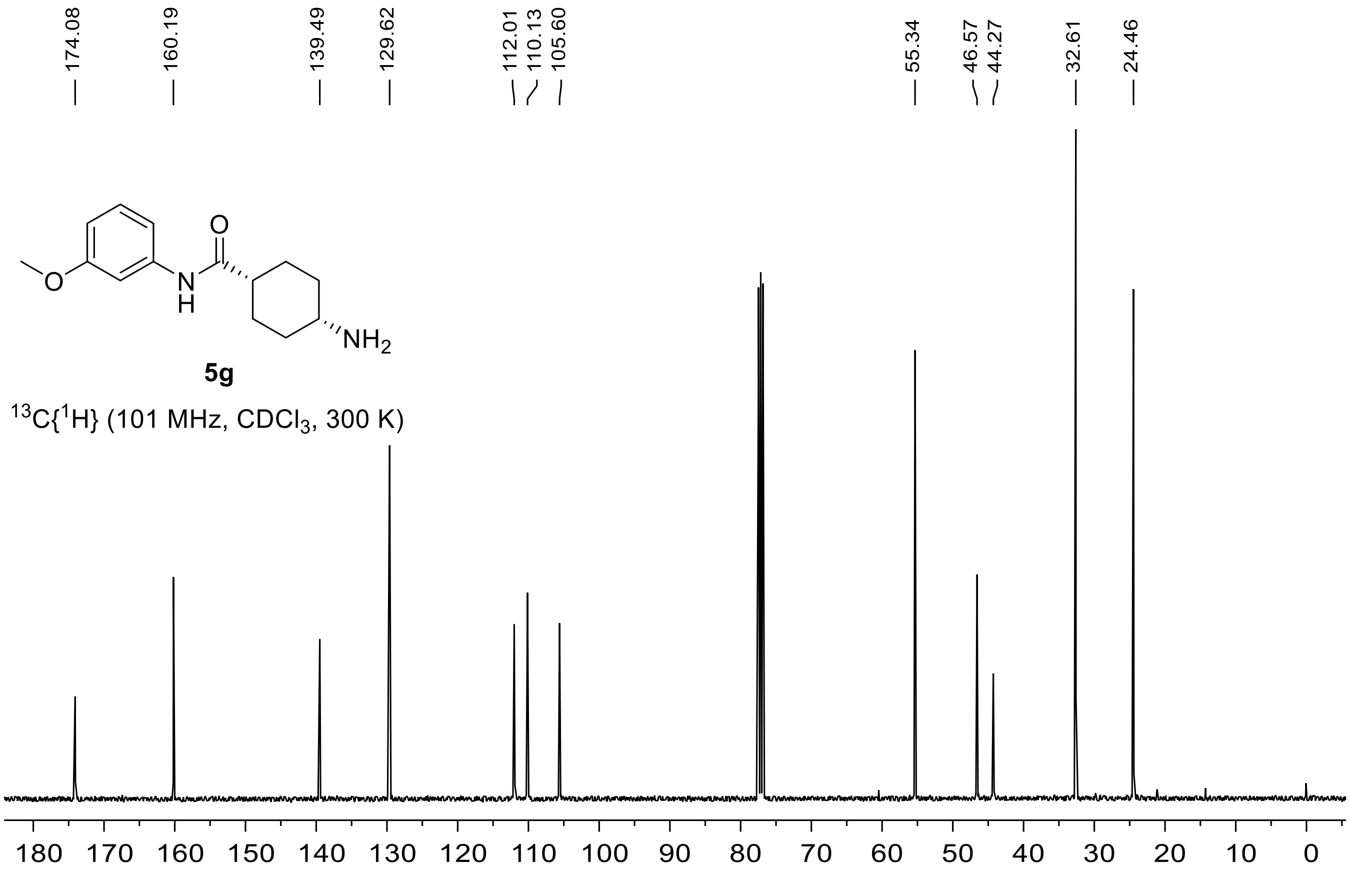

5g
^{13}C{^{1}H} (101 MHz, CDCl$_3$, 300 K)

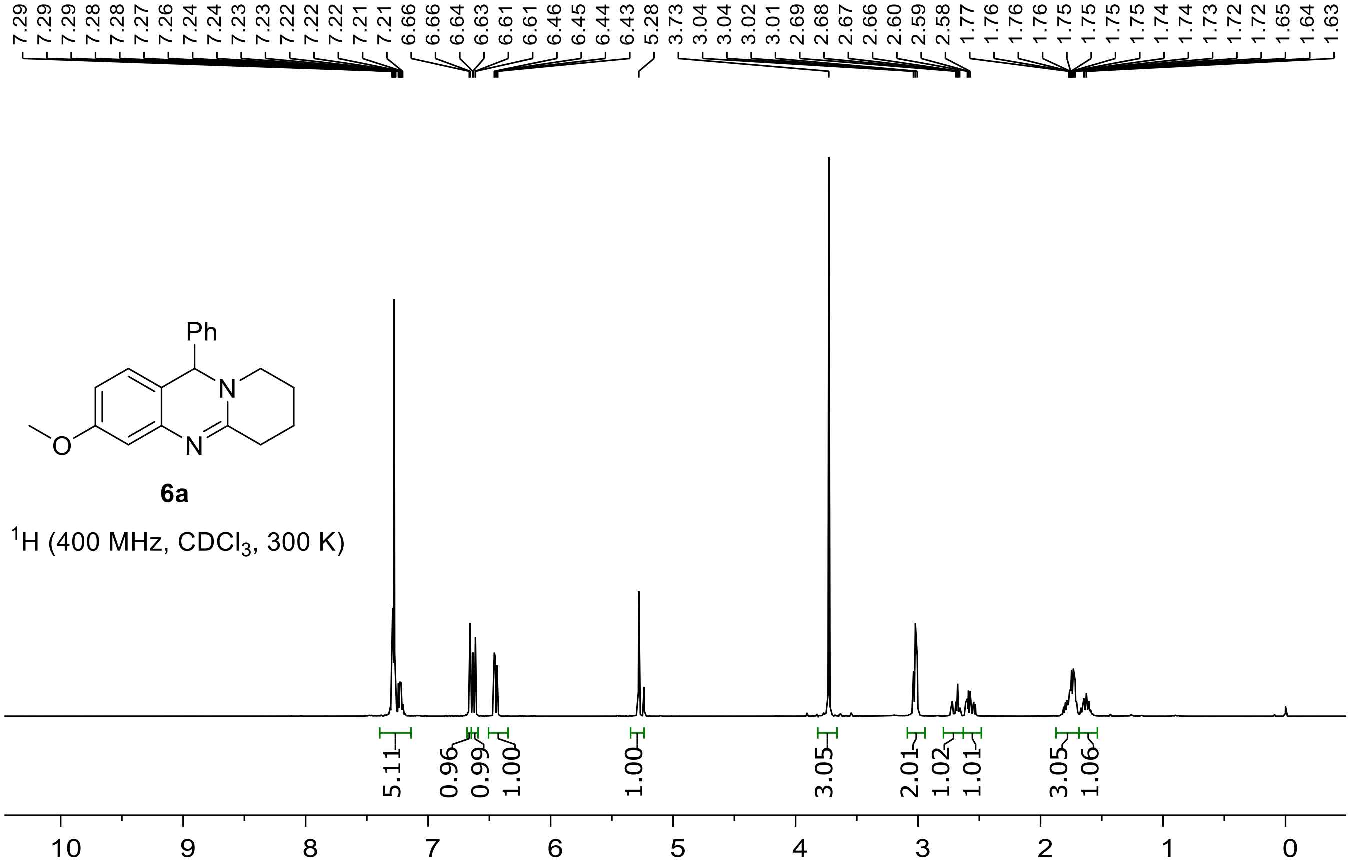
7.29 7.29 7.29 7.28 7.28 7.27 7.26 7.24 7.24 7.23 7.23 7.22 7.22 7.22 7.21 7.21 6.66 6.66 6.64 6.63 6.61 6.61 6.46 6.45 6.44 6.43 5.28 3.73 3.04 3.04 3.02 3.01 2.69 2.68 2.67 2.66 2.60 2.59 2.58 1.77 1.76 1.76 1.75 1.75 1.75 1.75 1.74 1.74 1.73 1.72 1.72 1.65 1.64 1.63
Ph
N
N
O
6a
1H (400 MHz, CDCl3, 300 K)
5.11 0.96 0.99 1.00 1.00 3.05 2.01 1.02 1.01 3.05 1.06
10 9 8 7 6 5 4 3 2 1 0

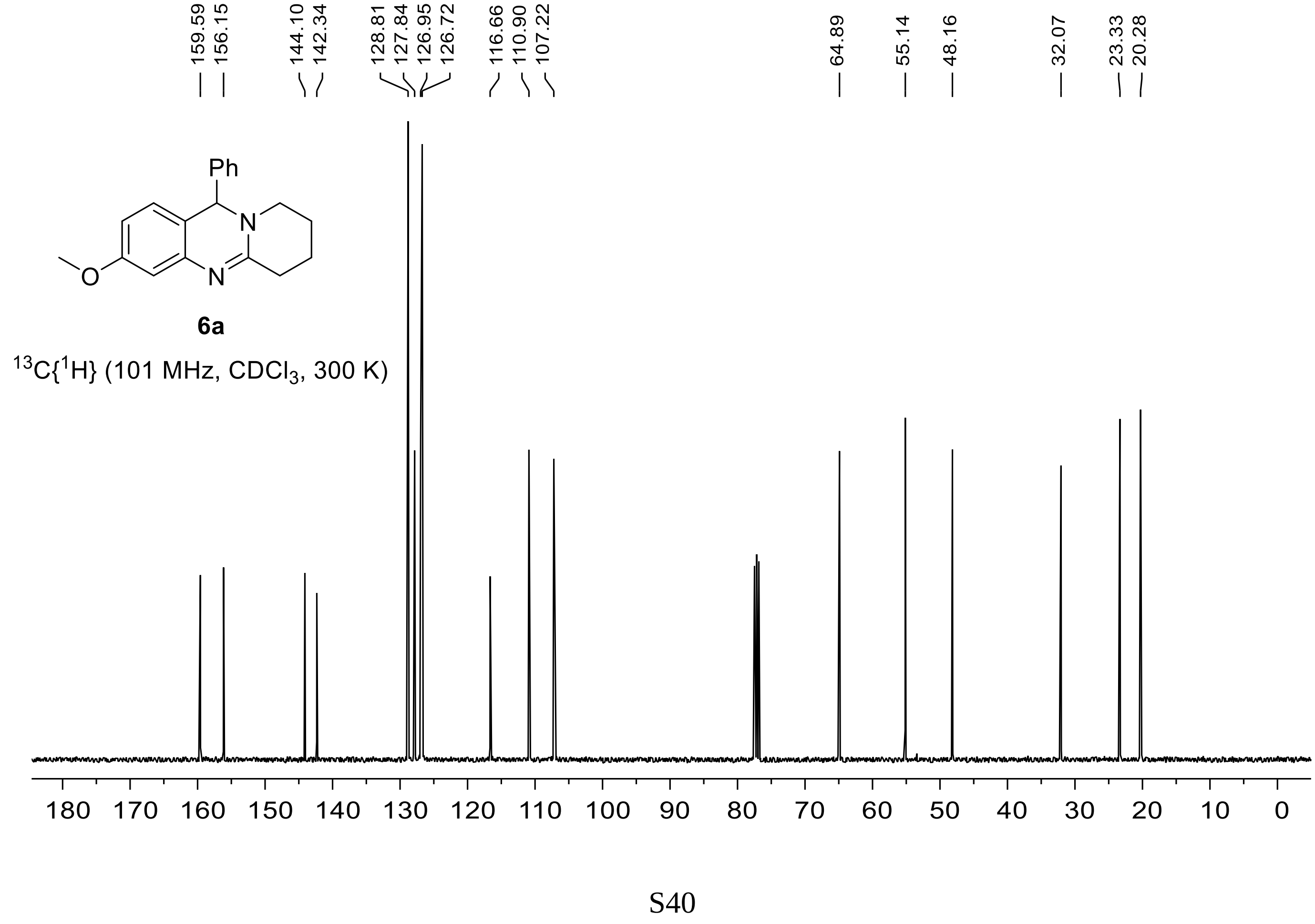
159.59 156.15 144.10 142.34 128.81 127.84 126.95 126.72 116.66 110.90 107.22 64.89 55.14 48.16 32.07 23.33 20.28
Ph
N
N
O
6a
13C{1H} (101 MHz, CDCl3, 300 K)
180 170 160 150 140 130 120 110 100 90 80 70 60 50 40 30 20 10 0

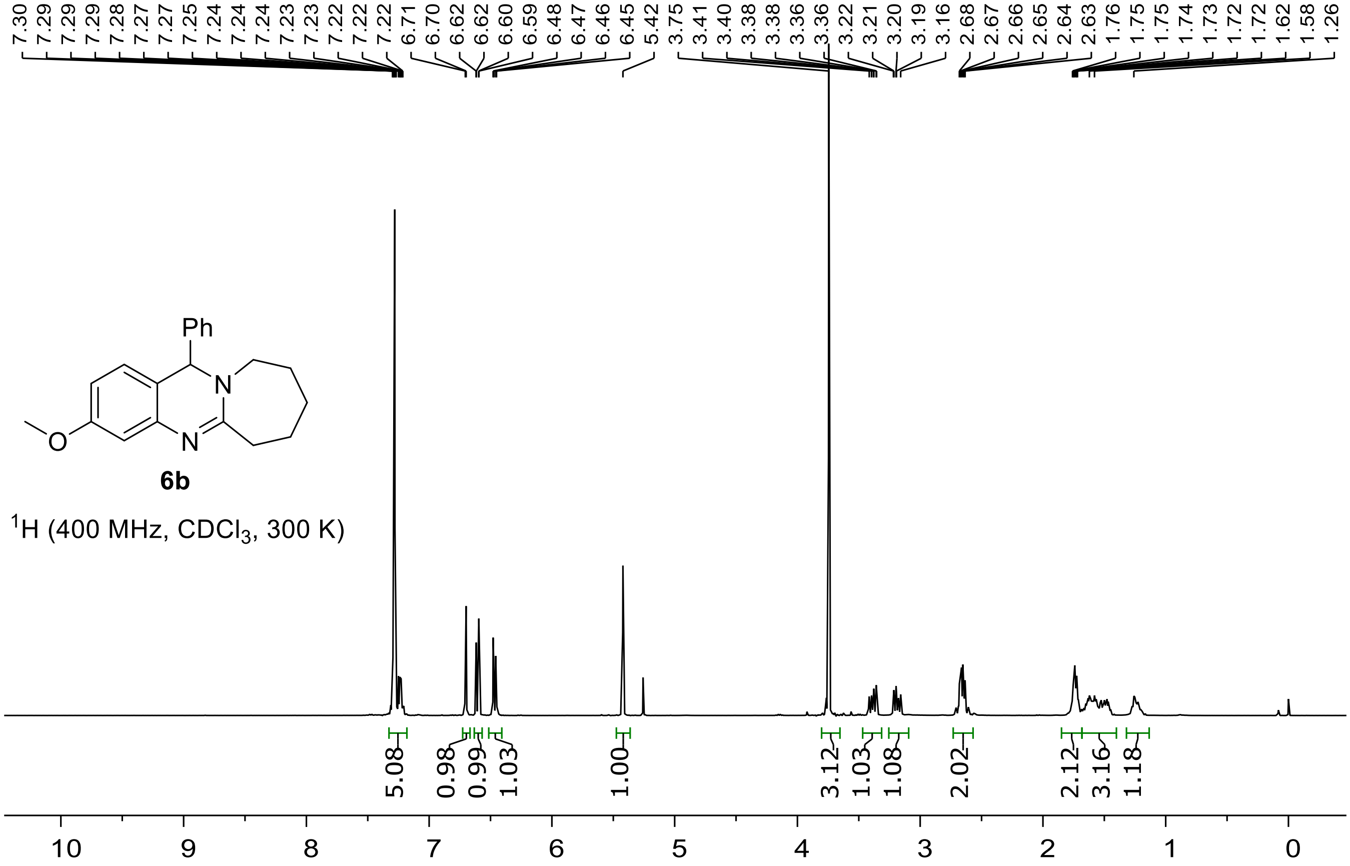
7.30 7.29 7.29 7.29 7.28 7.27 7.27 7.25 7.24 7.24 7.24 7.23 7.23 7.22 7.22 7.22 6.71 6.70 6.62 6.62 6.60 6.59 6.48 6.47 6.46 6.45 5.42 3.75 3.41 3.40 3.38 3.38 3.36 3.36 3.22 3.21 3.20 3.19 3.16 2.68 2.67 2.66 2.65 2.64 2.63 1.76 1.75 1.75 1.74 1.73 1.72 1.72 1.62 1.58 1.26
Ph
N
O
N
6b
1H (400 MHz, CDCl3, 300 K)
5.08 0.98 0.99 1.03 1.00 3.12 1.03 1.08 2.02 2.12 3.16 1.18
10 9 8 7 6 5 4 3 2 1 0

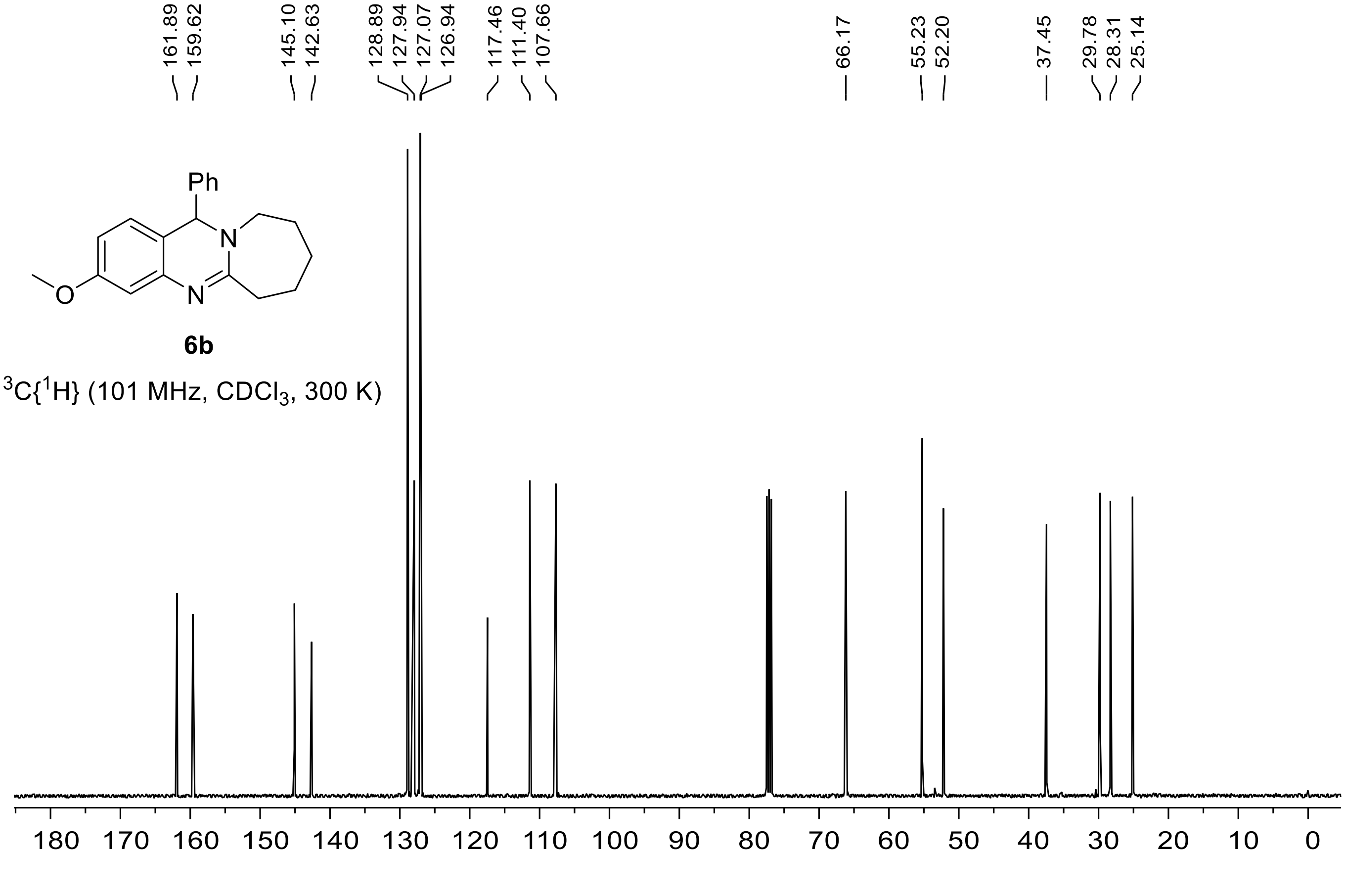
161.89 159.62 145.10 142.63 128.89 127.94 127.07 126.94 117.46 111.40 107.66 66.17 55.23 52.20 37.45 29.78 28.31 25.14
Ph
N
O
N
6b
13C{1H} (101 MHz, CDCl3, 300 K)
180 170 160 150 140 130 120 110 100 90 80 70 60 50 40 30 20 10 0

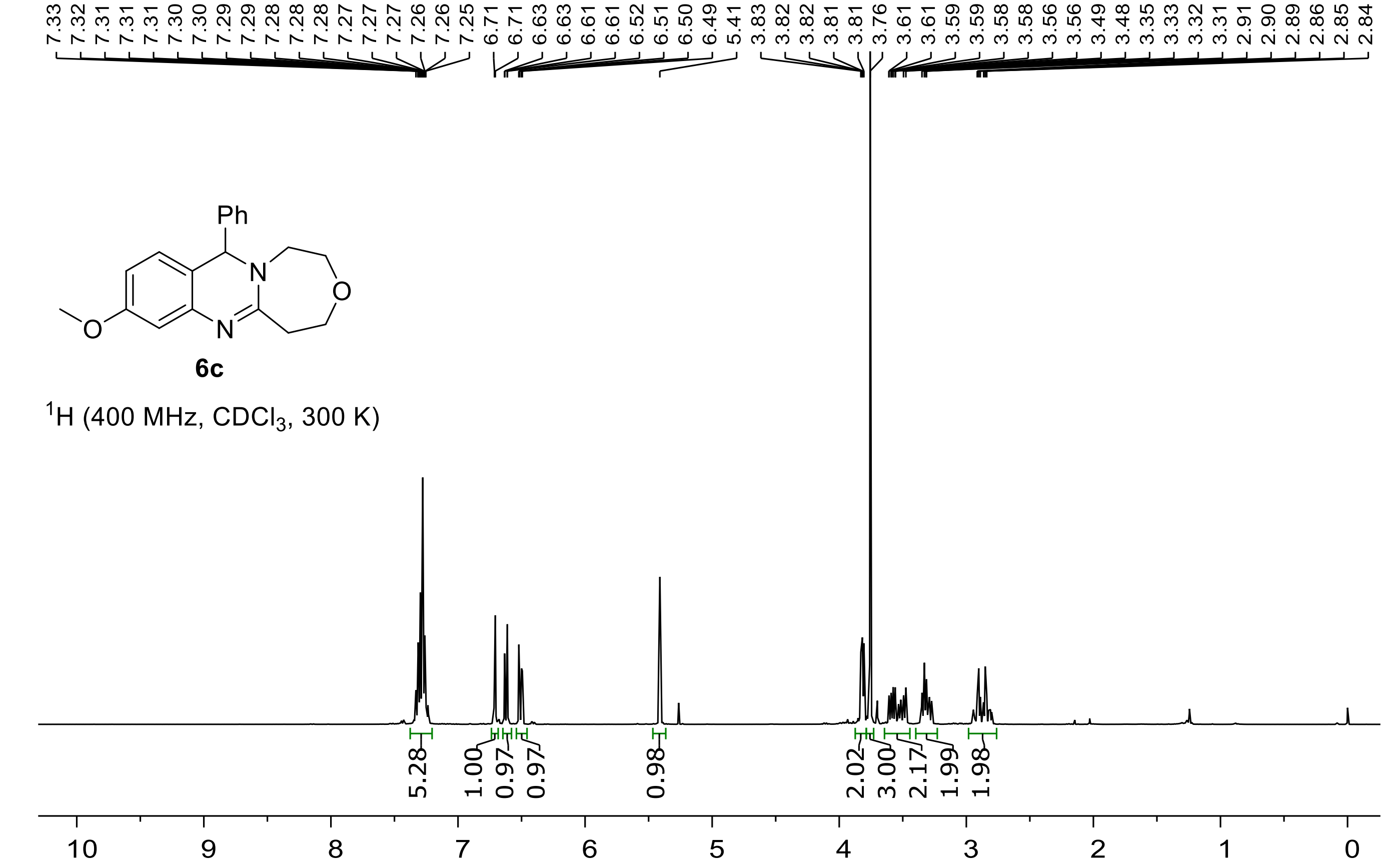
7.33 7.32 7.31 7.31 7.31 7.30 7.30 7.29 7.29 7.28 7.28 7.28 7.27 7.27 7.27 7.26 7.26 7.25 6.71 6.71 6.63 6.63 6.61 6.61 6.61 6.52 6.51 6.50 6.49 5.41 3.83 3.82 3.82 3.81 3.81 3.76 3.61 3.61 3.59 3.59 3.58 3.58 3.56 3.56 3.49 3.48 3.35 3.33 3.32 3.31 2.91 2.90 2.89 2.86 2.85 2.84
Ph
N
O
N
6c
¹H (400 MHz, CDCl₃, 300 K)
5.28 1.00 0.97 0.97 0.98 2.02 3.00 2.17 1.99 1.98
10 9 8 7 6 5 4 3 2 1 0

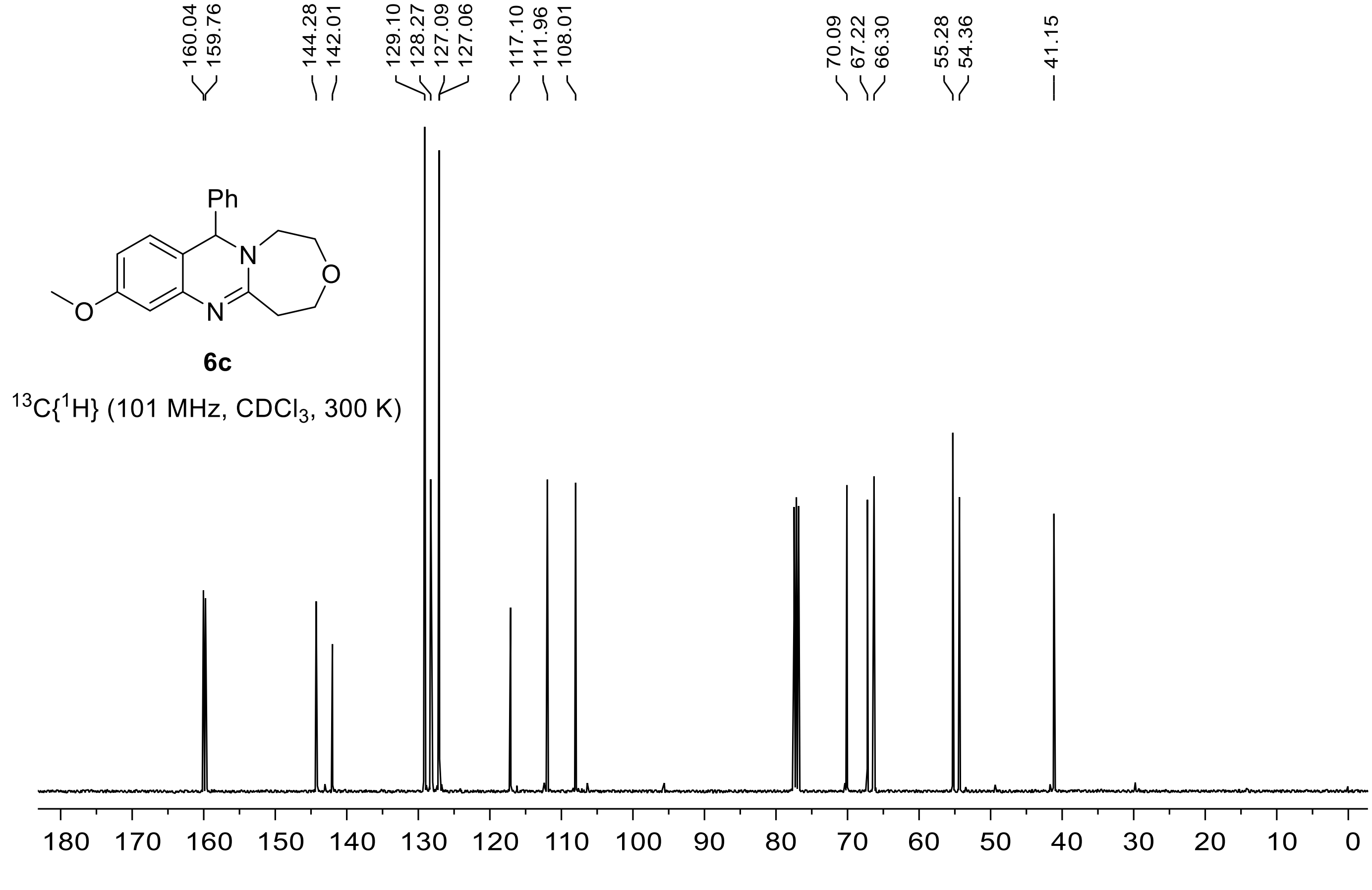
160.04 159.76 144.28 142.01 129.10 128.27 127.09 127.06 117.10 111.96 108.01 70.09 67.22 66.30 55.28 54.36 41.15
Ph
N
O
N
6c
¹³C{¹H} (101 MHz, CDCl₃, 300 K)
180 170 160 150 140 130 120 110 100 90 80 70 60 50 40 30 20 10 0

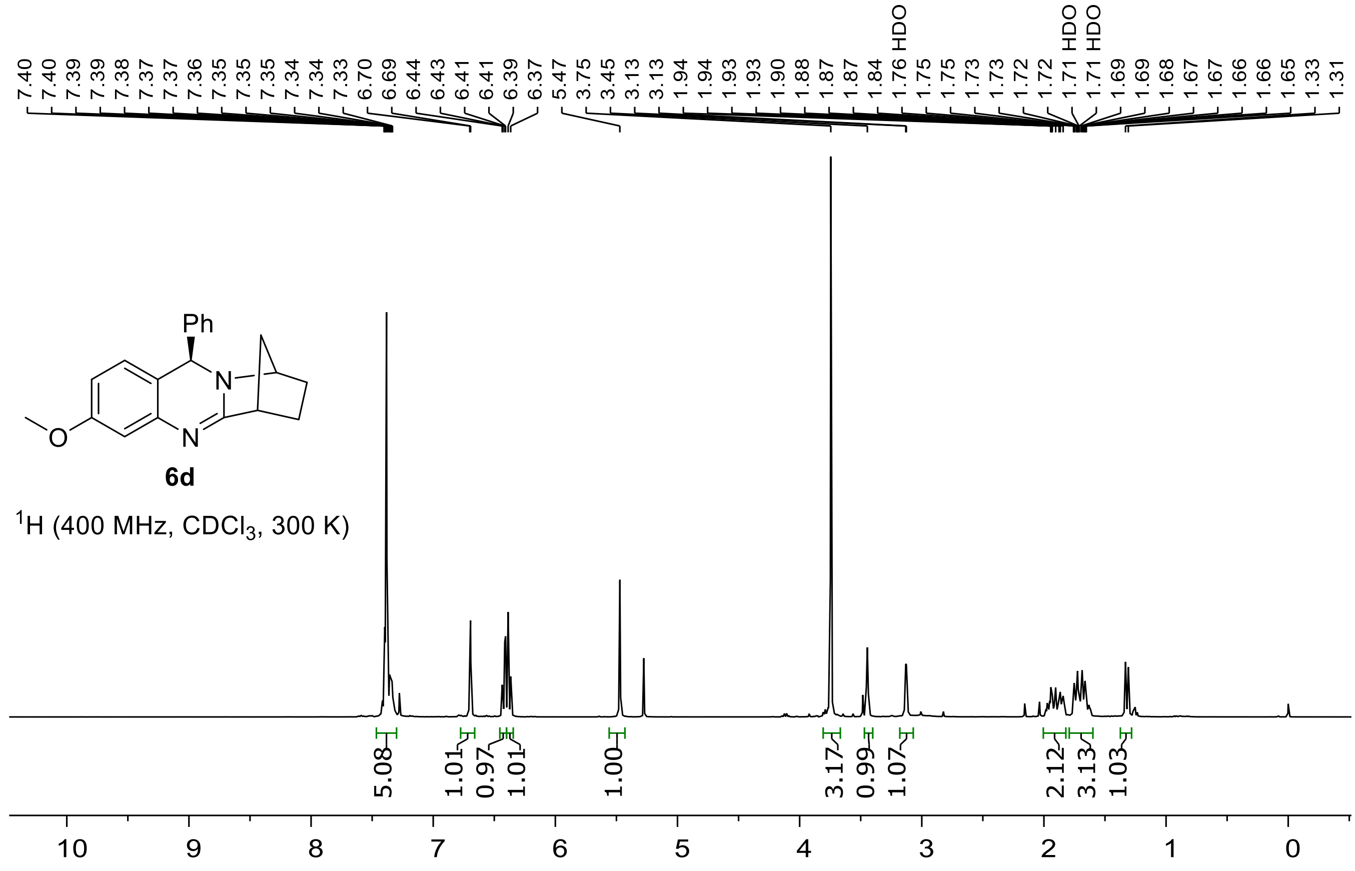
Ph
N
N
O
6d
^{1}H (400 MHz, CDCl3, 300 K)
7.40 7.40 7.39 7.39 7.38 7.37 7.37 7.36 7.35 7.35 7.35 7.34 7.34 7.33 6.70 6.69 6.44 6.43 6.41 6.41 6.39 6.37 5.47 3.75 3.45 3.13 3.13 1.94 1.94 1.93 1.93 1.90 1.88 1.87 1.87 1.84 1.76 HDO 1.75 1.75 1.73 1.73 1.72 1.72 1.71 HDO 1.71 HDO 1.69 1.69 1.68 1.67 1.67 1.66 1.66 1.65 1.33 1.31
5.08 1.01 0.97 1.01 1.00 3.17 0.99 1.07 2.12 3.13 1.03
10 9 8 7 6 5 4 3 2 1 0
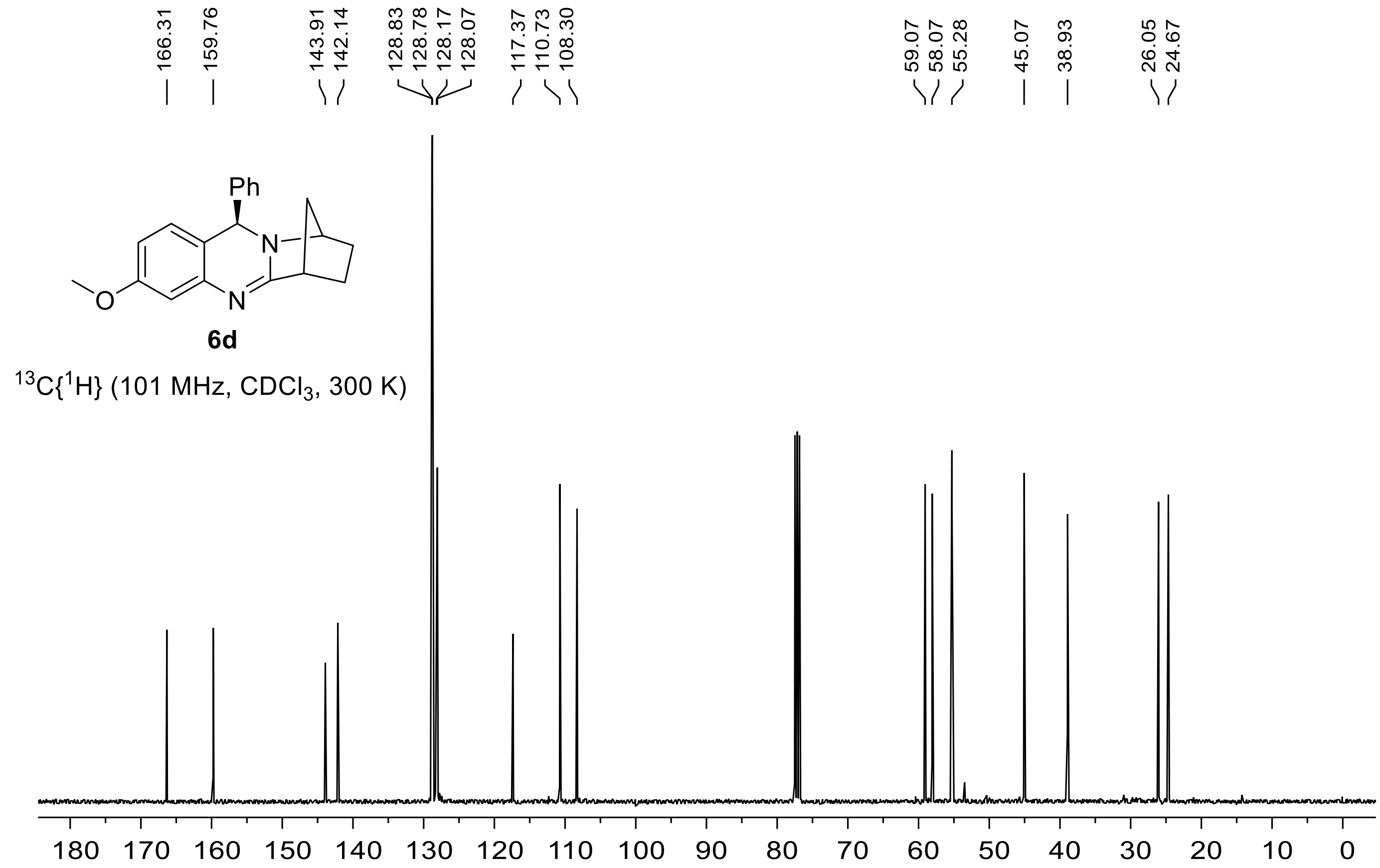
Ph
N
N
O
6d
^{13}C{^{1}H} (101 MHz, CDCl3, 300 K)
166.31 159.76 143.91 142.14 128.83 128.78 128.17 128.07 117.37 110.73 108.30 59.07 58.07 55.28 45.07 38.93 26.05 24.67
180 170 160 150 140 130 120 110 100 90 80 70 60 50 40 30 20 10 0

NOESY Spectrum of **6d**

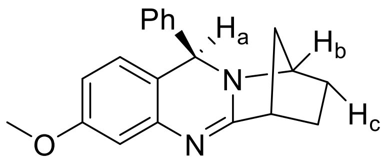

6d

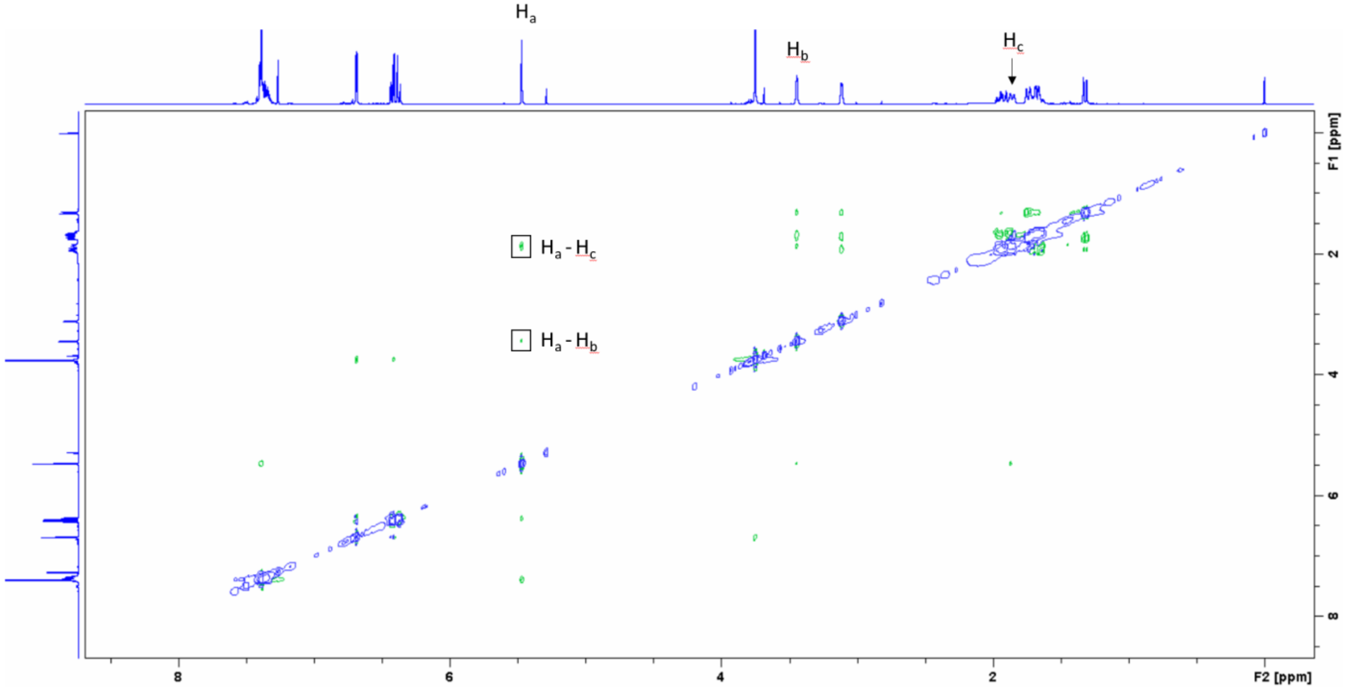

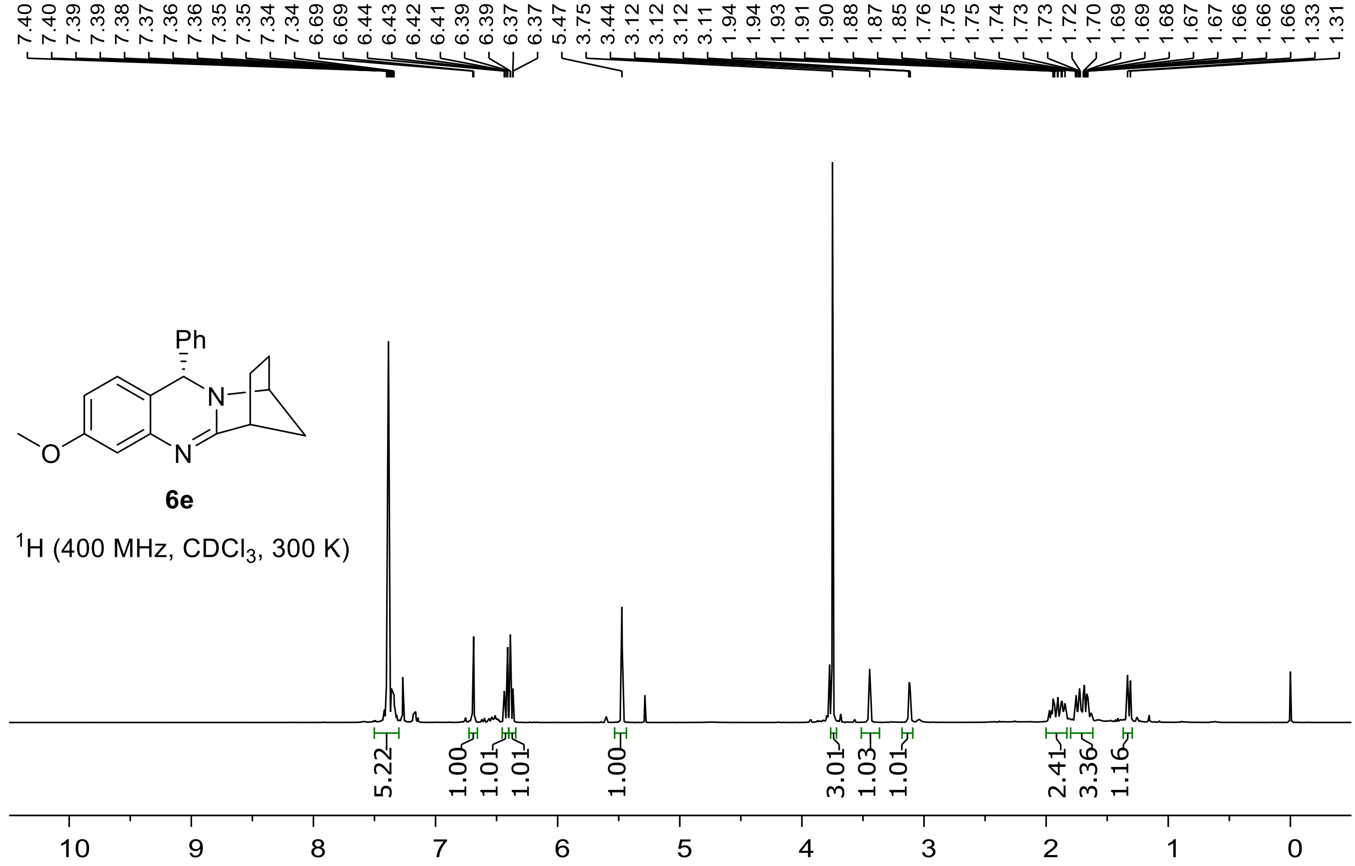
7.40 7.40 7.39 7.39 7.38 7.37 7.36 7.36 7.35 7.35 7.34 7.34 6.69 6.69 6.44 6.43 6.42 6.41 6.39 6.39 6.37 6.37 5.47 3.75 3.44 3.12 3.12 3.12 3.11 1.94 1.94 1.93 1.91 1.90 1.88 1.87 1.85 1.76 1.75 1.75 1.74 1.73 1.73 1.72 1.70 1.69 1.69 1.68 1.67 1.67 1.66 1.66 1.66 1.33 1.31
Ph
N
N
O
6e
^{1}H (400 MHz, $CDCl_3$, 300 K)
5.22 1.00 1.01 1.01 1.00 3.01 1.03 1.01 2.41 3.36 1.16
10 9 8 7 6 5 4 3 2 1 0

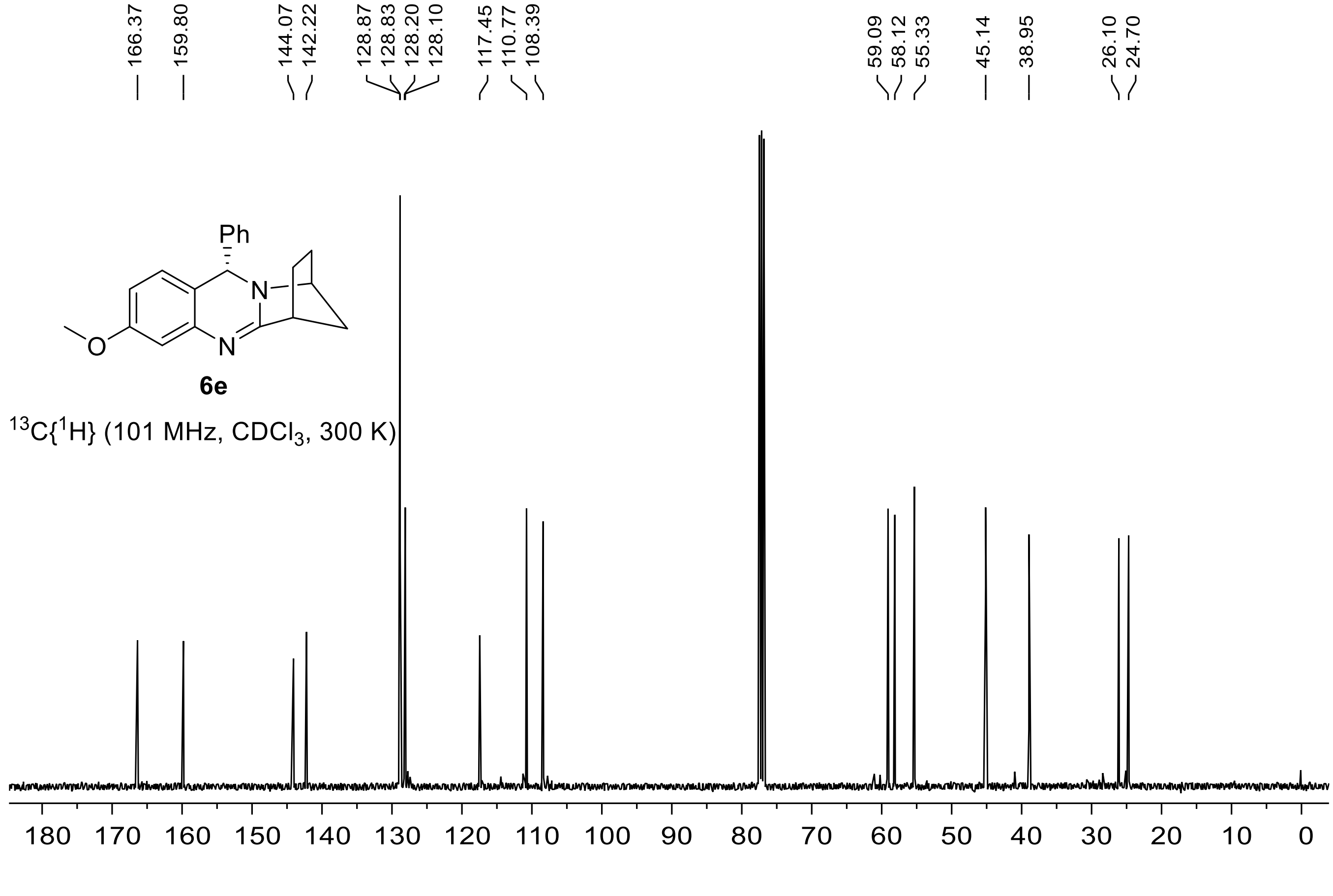
166.37 159.80 144.07 142.22 128.87 128.83 128.20 128.10 117.45 110.77 108.39 59.09 58.12 55.33 45.14 38.95 26.10 24.70
Ph
N
N
O
6e
$^{13}C\{^{1}H\}$ (101 MHz, $CDCl_3$, 300 K)
180 170 160 150 140 130 120 110 100 90 80 70 60 50 40 30 20 10 0

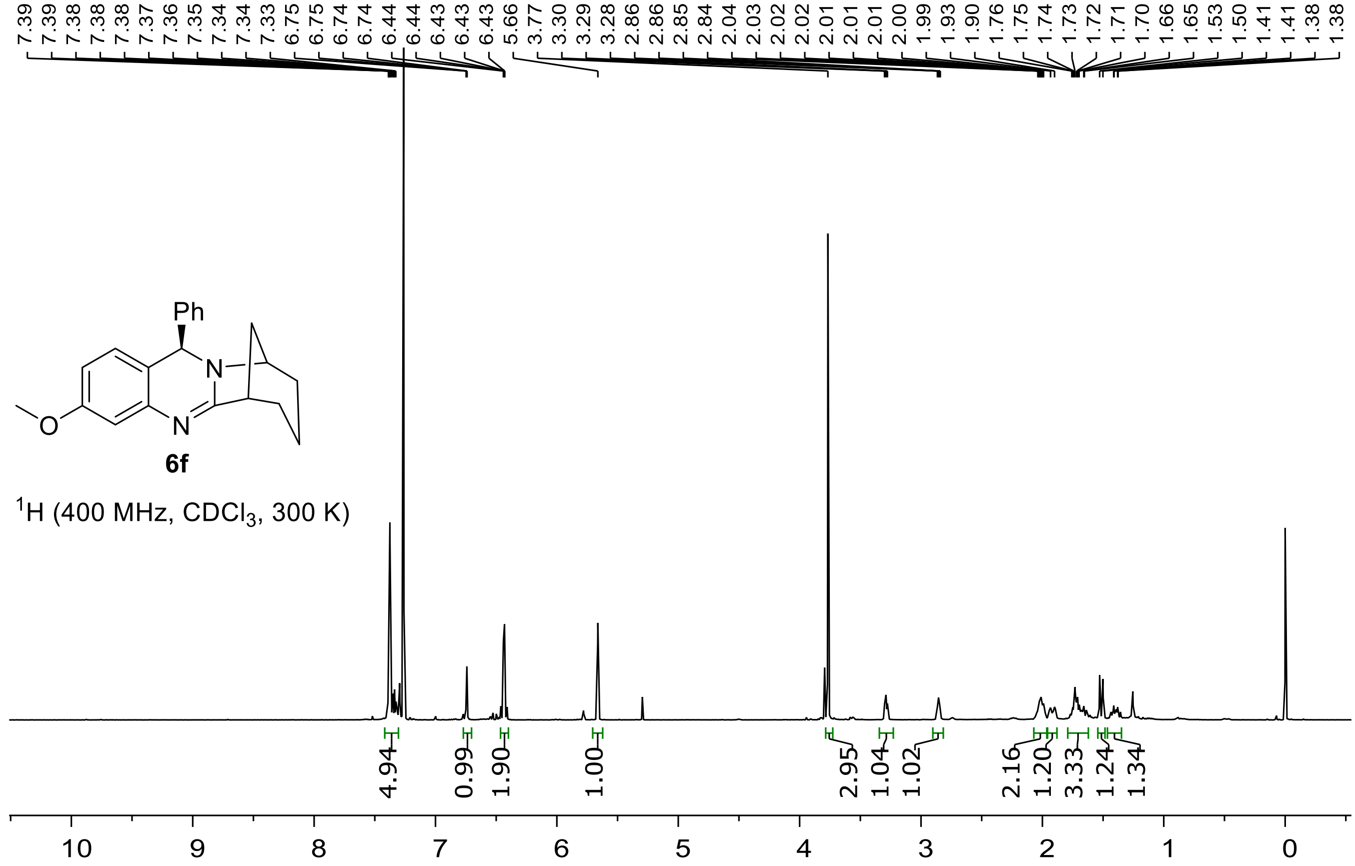
7.39 7.39 7.38 7.38 7.38 7.37 7.36 7.35 7.34 7.34 7.33 6.75 6.75 6.74 6.74 6.44 6.44 6.43 6.43 6.43 5.66 3.77 3.30 3.29 3.28 2.86 2.86 2.85 2.84 2.04 2.03 2.02 2.02 2.01 2.01 2.01 2.00 1.99 1.93 1.90 1.76 1.75 1.74 1.73 1.72 1.71 1.70 1.66 1.65 1.53 1.50 1.41 1.41 1.38 1.38
Ph
N
N
O
6f
^{1}H (400 MHz, CDCl3, 300 K)
4.94 0.99 1.90 1.00 2.95 1.04 1.02 2.16 1.20 3.33 1.24 1.34
10 9 8 7 6 5 4 3 2 1 0

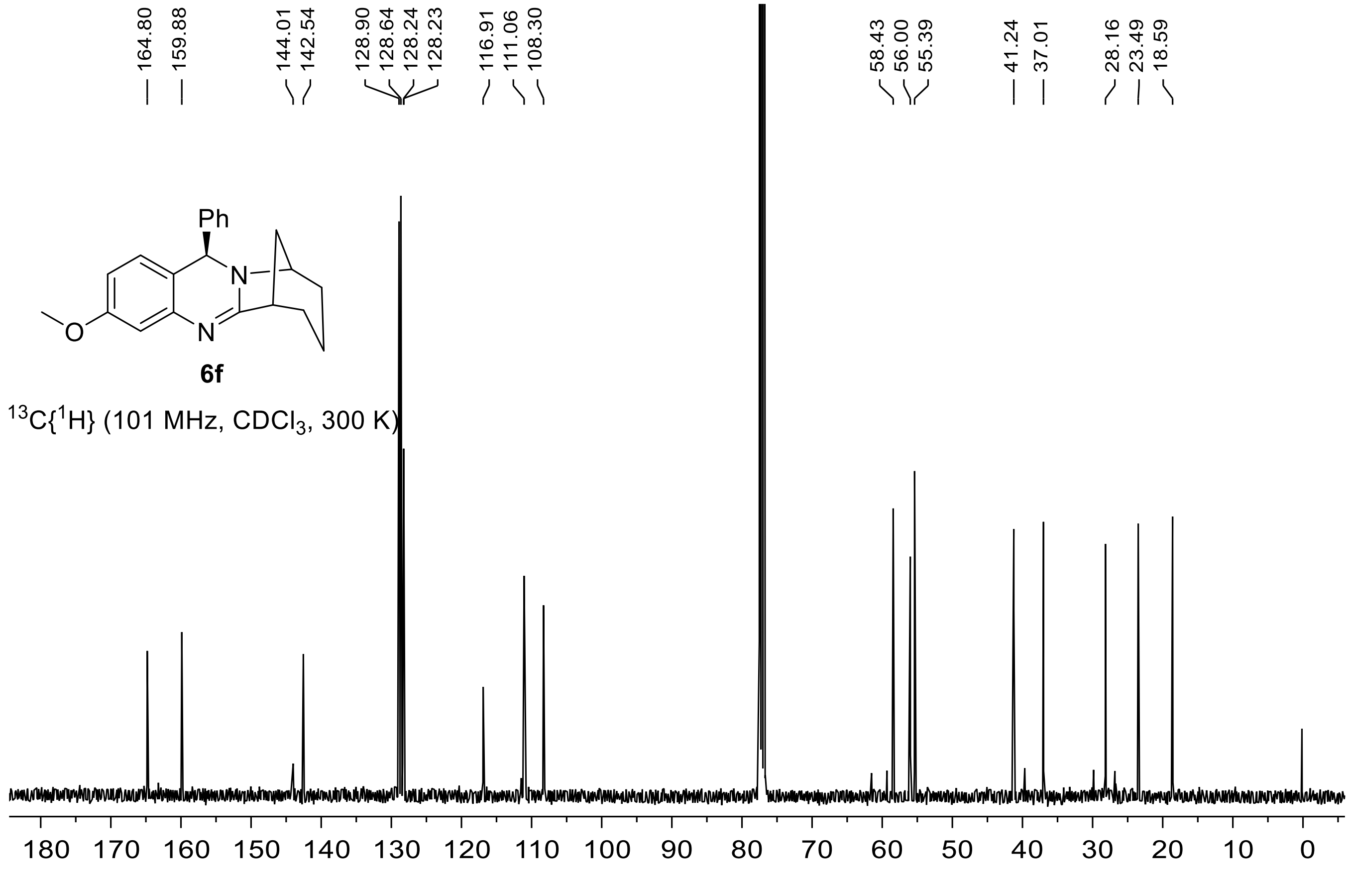
164.80 159.88 144.01 142.54 128.90 128.64 128.24 128.23 116.91 111.06 108.30 58.43 56.00 55.39 41.24 37.01 28.16 23.49 18.59
Ph
N
N
O
6f
^{13}C{^{1}H} (101 MHz, CDCl3, 300 K)
180 170 160 150 140 130 120 110 100 90 80 70 60 50 40 30 20 10 0

NOESY Spectrum of **6f**

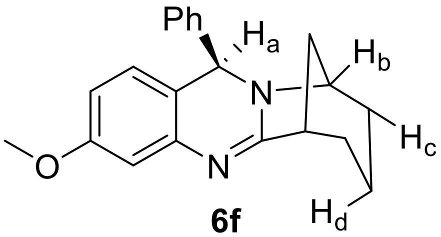

6f

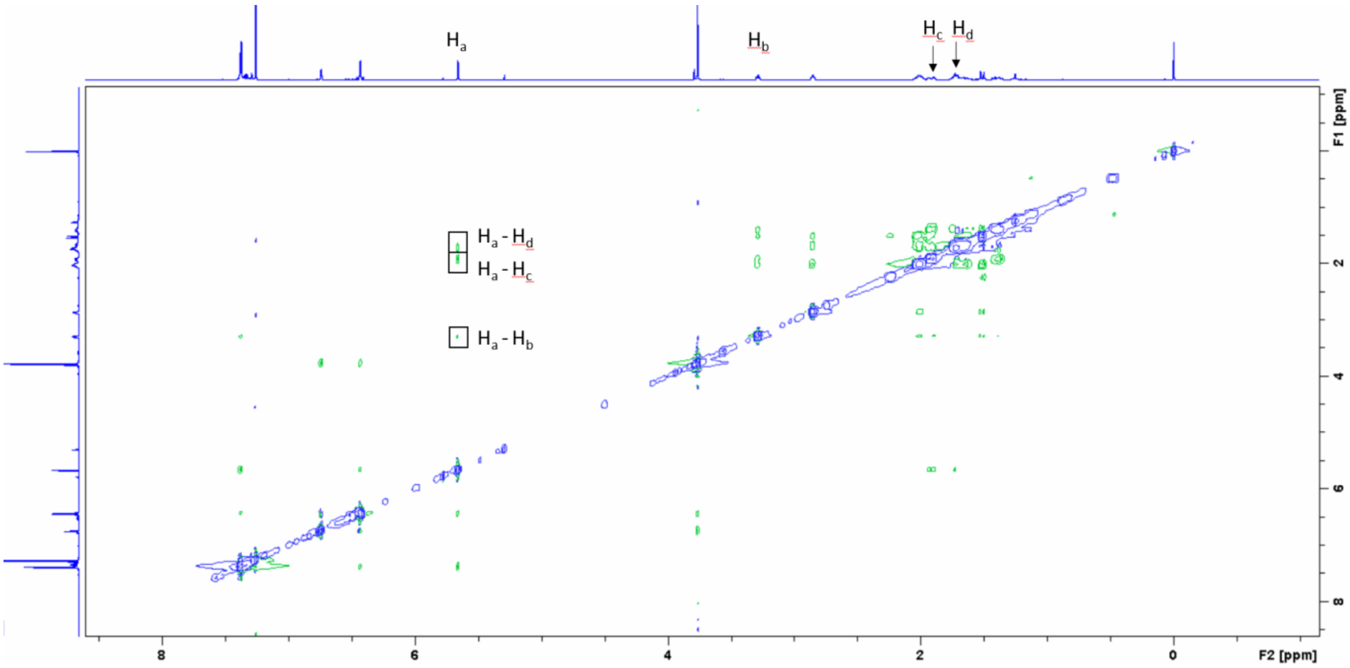

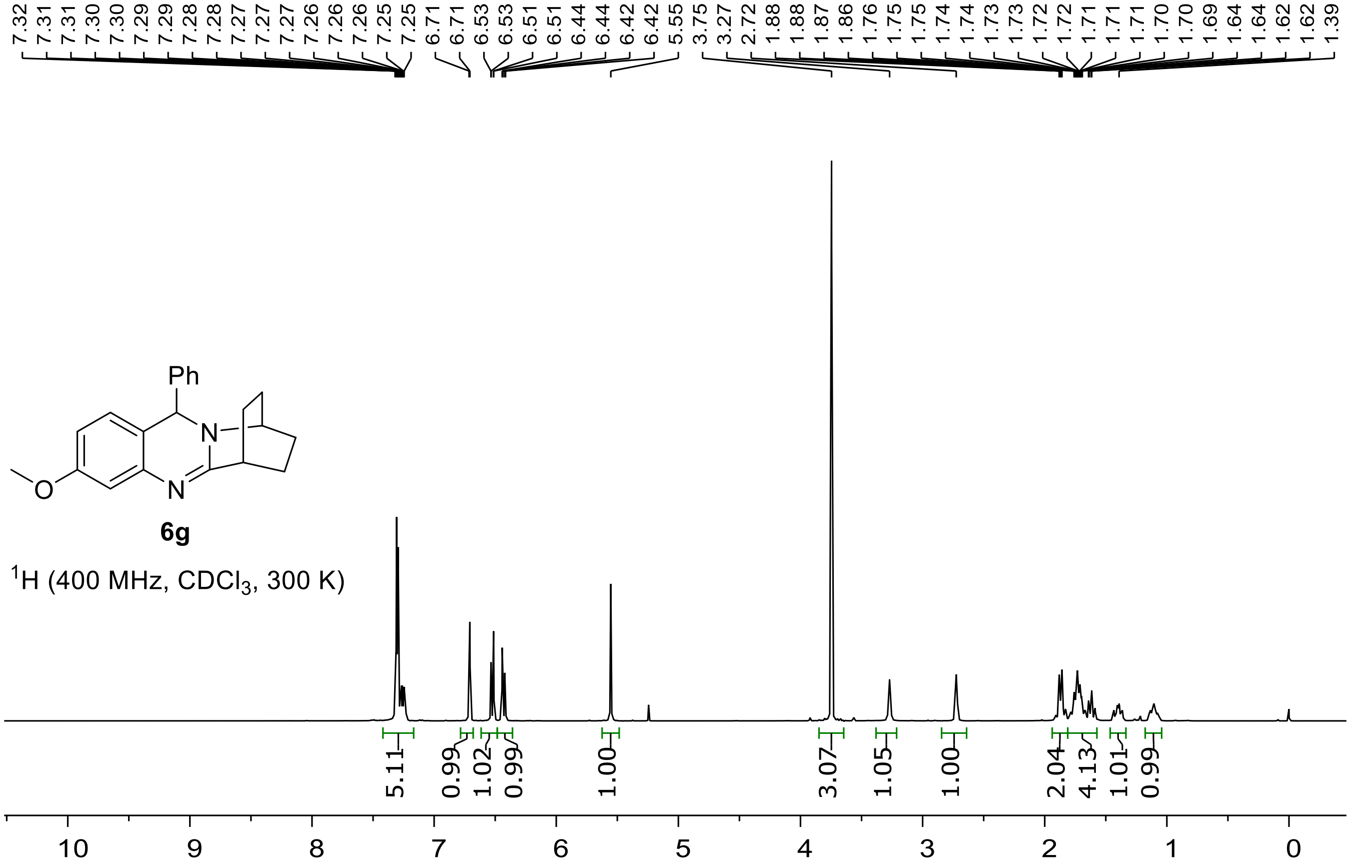
7.32 7.31 7.31 7.30 7.30 7.29 7.29 7.28 7.28 7.27 7.27 7.27 7.26 7.26 7.26 7.25 7.25 6.71 6.71 6.53 6.53 6.51 6.51 6.44 6.44 6.42 6.42 5.55 3.75 3.27 2.72 1.88 1.88 1.87 1.86 1.76 1.75 1.75 1.74 1.74 1.73 1.73 1.72 1.72 1.71 1.71 1.71 1.70 1.70 1.69 1.64 1.64 1.62 1.62 1.39
Ph
N
N
O
6g
1H (400 MHz, CDCl3, 300 K)
5.11 0.99 1.02 0.99 1.00 3.07 1.05 1.00 2.04 4.13 1.01 0.99
10 9 8 7 6 5 4 3 2 1 0

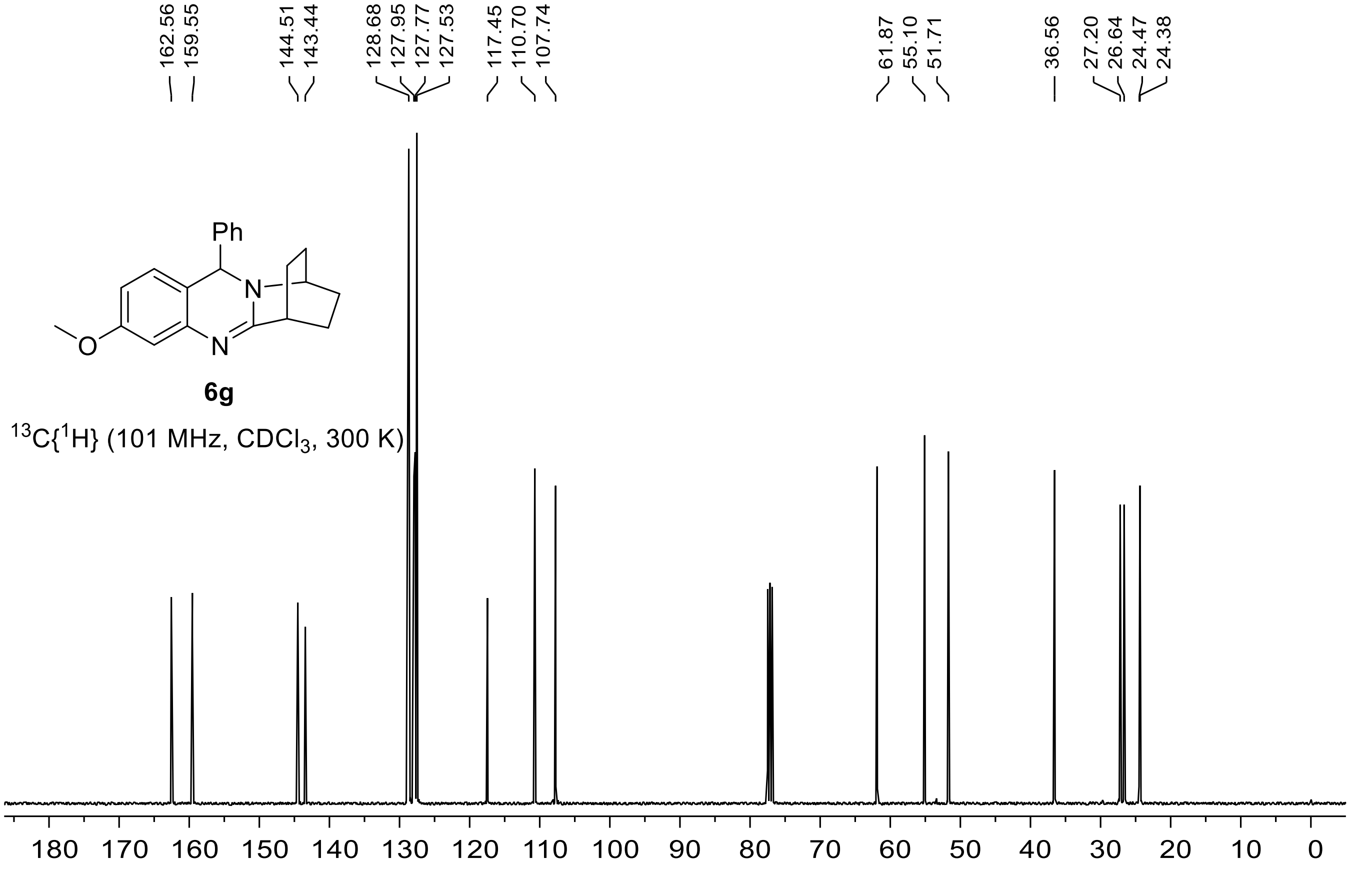
162.56 159.55 144.51 143.44 128.68 127.95 127.77 127.53 117.45 110.70 107.74 61.87 55.10 51.71 36.56 27.20 26.64 24.47 24.38
Ph
N
N
O
6g
13C{1H} (101 MHz, CDCl3, 300 K)
180 170 160 150 140 130 120 110 100 90 80 70 60 50 40 30 20 10 0

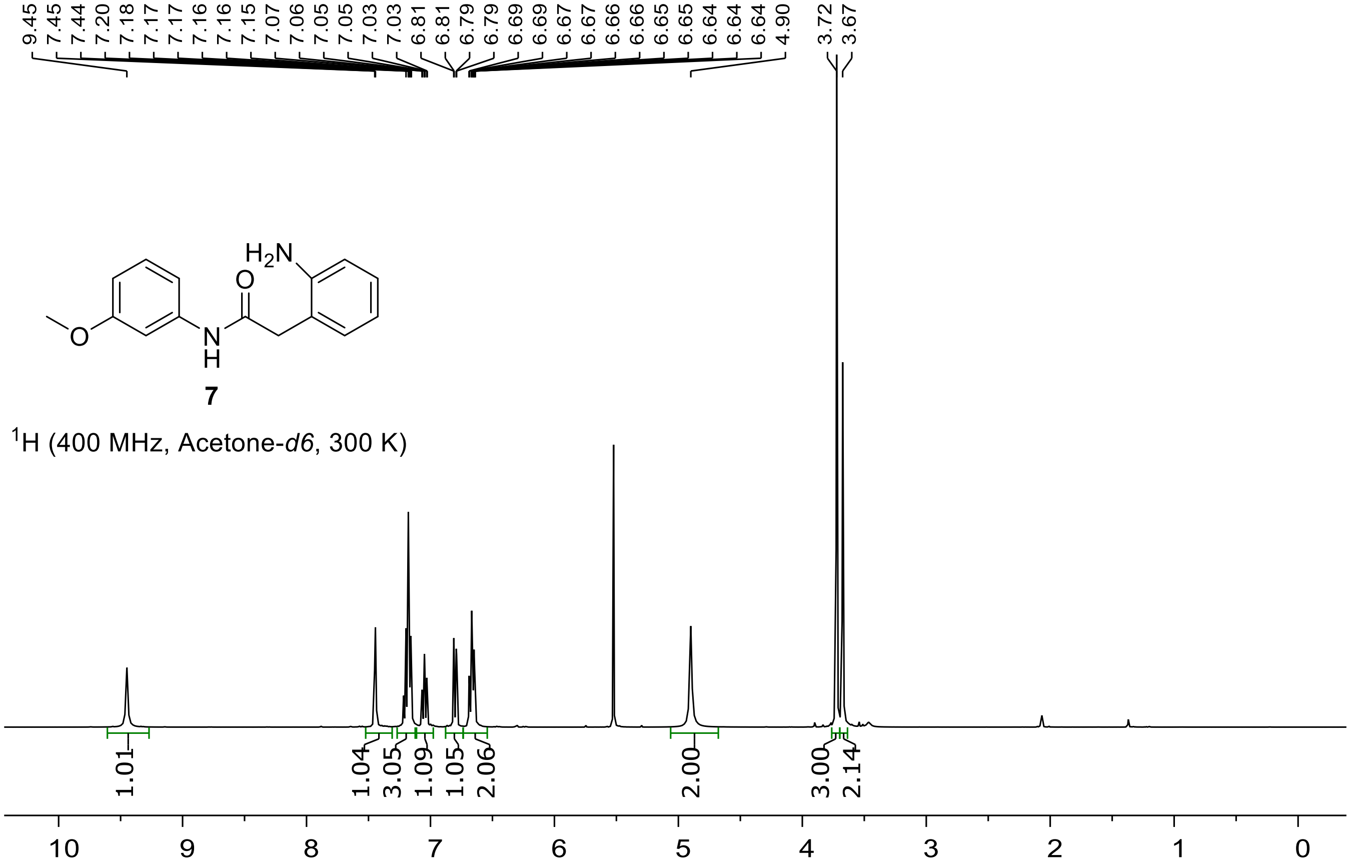
9.45 7.45 7.44 7.20 7.18 7.17 7.17 7.16 7.16 7.15 7.07 7.06 7.05 7.05 7.03 7.03 6.81 6.81 6.79 6.79 6.69 6.69 6.67 6.67 6.66 6.66 6.65 6.65 6.64 6.64 6.64 4.90 3.72 3.67
H2N
O
O
N
H
7
1H (400 MHz, Acetone-d6, 300 K)
1.01 1.04 3.05 1.09 1.05 2.06 2.00 3.00 2.14
10 9 8 7 6 5 4 3 2 1 0

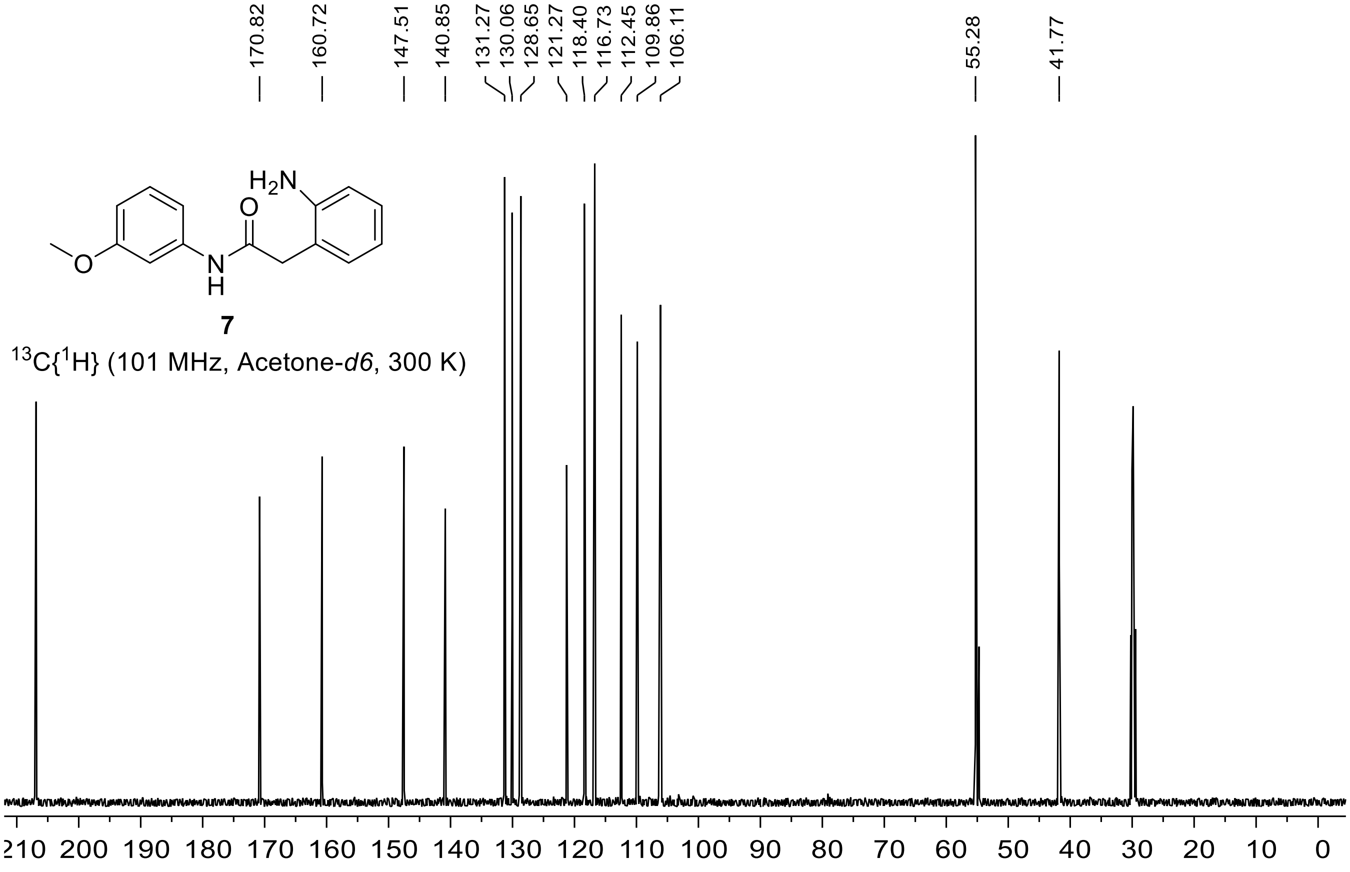
170.82 160.72 147.51 140.85 131.27 130.06 128.65 121.27 118.40 116.73 112.45 109.86 106.11 55.28 41.77
H2N
O
O
N
H
7
13C{1H} (101 MHz, Acetone-d6, 300 K)
210 200 190 180 170 160 150 140 130 120 110 100 90 80 70 60 50 40 30 20 10 0

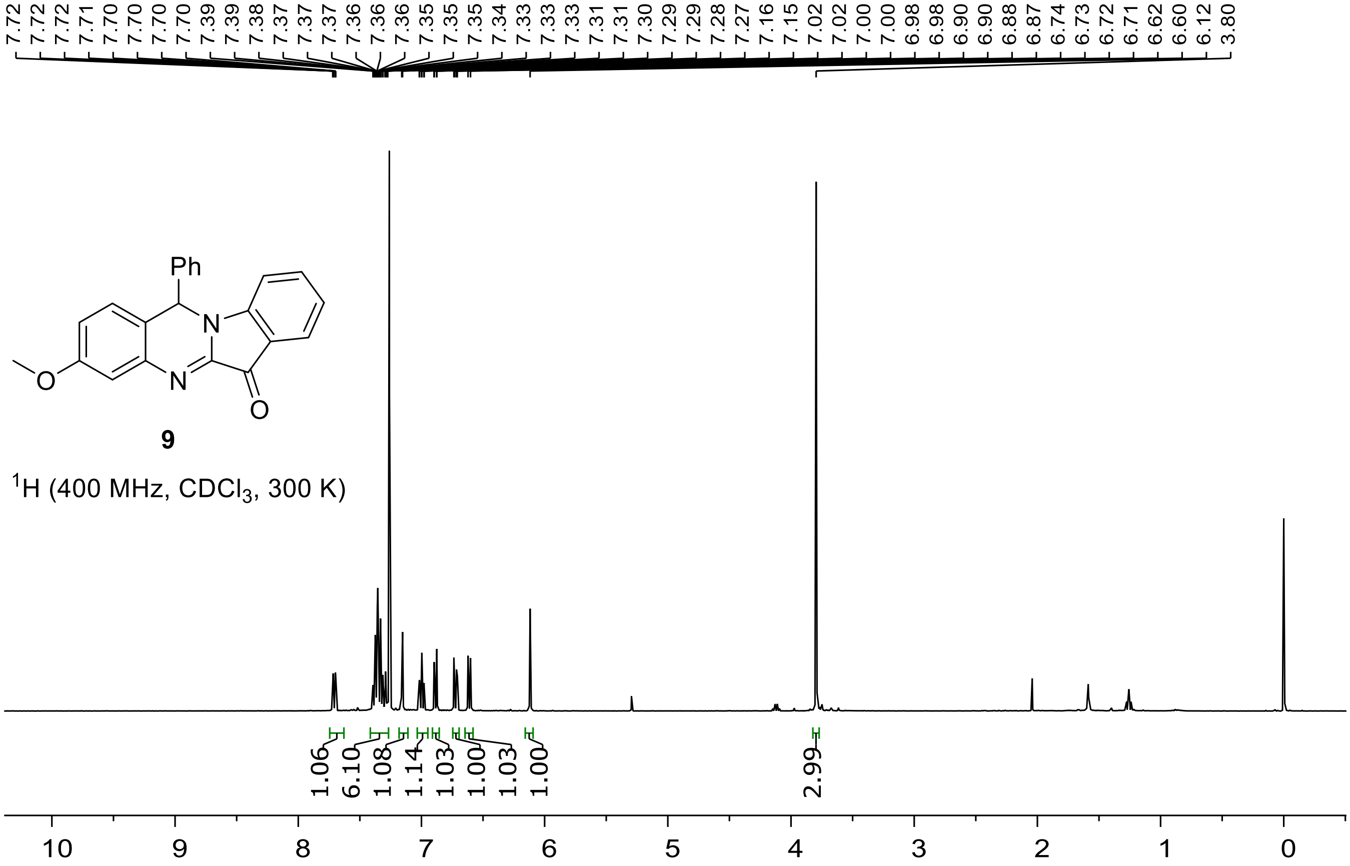
7.72 7.72 7.72 7.71 7.70 7.70 7.70 7.39 7.39 7.38 7.37 7.37 7.37 7.36 7.36 7.36 7.35 7.35 7.35 7.34 7.33 7.33 7.33 7.31 7.31 7.30 7.29 7.29 7.28 7.27 7.16 7.15 7.02 7.02 7.00 7.00 6.98 6.98 6.90 6.90 6.88 6.87 6.74 6.73 6.72 6.71 6.62 6.60 6.12 3.80
Ph
N
N
O
O
9
^{1}H (400 MHz, CDCl$_3$, 300 K)
1.06 6.10 1.08 1.14 1.03 1.00 1.03 1.00
2.99
10 9 8 7 6 5 4 3 2 1 0

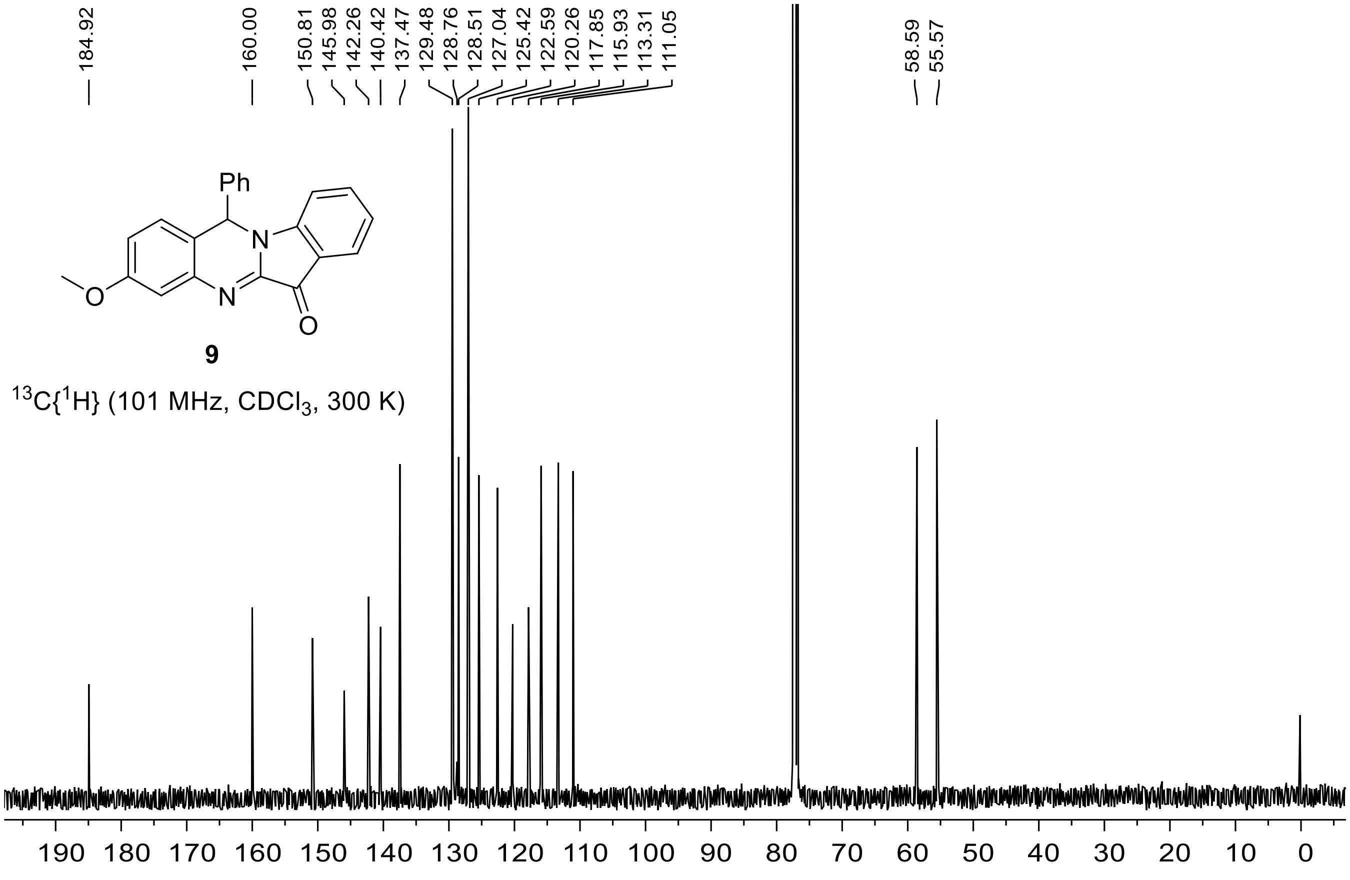
184.92
160.00
150.81 145.98 142.26 140.42 137.47 129.48 128.76 128.51 127.04 125.42 122.59 120.26 117.85 115.93 113.31 111.05
58.59 55.57
Ph
N
N
O
O
9
^{13}C{^{1}H} (101 MHz, CDCl$_3$, 300 K)
190 180 170 160 150 140 130 120 110 100 90 80 70 60 50 40 30 20 10 0
